# Supplementary material for: Assessing population structure and body condition to inform conservation strategies for a small isolated Asian elephant (Elephas maximus) population in southwest China
Source: PLoS One. 2021 Mar 9;16(3):e0248210. doi: 10.1371/journal.pone.0248210 (PMC7942997; doi:10.1371/journal.pone.0248210)
Supplement: S1 Table — (DOCX) [file pone.0248210.s001.docx]

**S1 Table. Historical elephant population in NNNR estimated by different methods.**

| Years | Methods | Effort | Number | Population description | Data resource |
| --- | --- | --- | --- | --- | --- |
| 1981 | - | - | 12 | Two small herds and a solitary, unknown sex and age | [1] |
| 1981 | - | - | 16 | One family with 10 and one with 4, 2 solitary, unknown sex and age | [2] |
| 1982 | Wild track and questionnaires | Several days | 14 | One family with 4 (two mothers and two cubs), one family with 9, one solitary, unknown sex and age | [3] |
| 1984 | - | - | 18-21 | Four families, the biggest family has 12, unknown sex and age | [4] |
| 1986 | Questionnaires | - | 14-16 | Two families, unknown sex and age | [5] |
| 1987 | Dung counting | 7 days | About 12 | Observed one family with 5 and one with 2, 2 solitary, unknown sex and age | [6] |
| 1992 | Questionnaire and sign survey | 5 months | 18 | No description on population number and unknown sex and age | [7] |
| 1992 | Observed and dung counting | 2 months | Observed 11 | No description on population number and unknown sex and age | [8] |
| 2004 | Dung DNA | - | 18-23 | No description on population number and unknown sex and age | [9] |
| 2007 | Wild track and dung counting | 5 months | At least 14 | 4 juveniles, 6 subadults and 4 adults, unknown sex and age | [10] |
| 2009 | Dung DNA | 94 samples | 18-23 | No description on population number and unknown sex and age | [11] |
| 2014 | Transect survey and dung DNA | - | 20-23 | No description on population number and unknown sex and age | [12] |
| 2018 | Camera traps and SECR | 4 months | 12 | One family with 8 and 4 solitary individuals, 3 female adults, 3 male adults, 1 male subadult, 2 female juveniles, 2 male juveniles, 1 male calf | This study |
| 2018 | Camera traps and drones | 6 months | 12 | 2 adult males,1 sub-adult male, 3 adult females and 6 baby elephants | [13] |
| 2019 | - | - | 12 | - | [14] |

1. Peiyan L, Jianhao W. WIld elephant population in China. Chinese Journal of Wildlife. 1983:27-29.

2. Institute YFIaP. Nature Reserve in Yunnan: China Forestry Press; 1989.

3. Yuming Y, Genmin W, Zhisheng Z, Zhongli W. Wild elephant population in Nangunhe reserve. Forest Inventory & Planning. 1982.

4. Yang DH, Zhang J, Li C. Preliminary survey on the population and distribution of gibbons in yunnan province. Primates. 1987;28(4):547-549.

5. Tang J, Wang J, Huang X. Summary of Nangunhe nature reserve. Forest Inventory & Planning. 1986.

6. Guo B. Investigation and protection advice of wild elephant in Nangunhe reserve. YUnnan Forestry. 1989.

7. Ning L, Yongjie L. The History and Present Situation and Prospect of Zoology Research in Nangun River National Nature Reserve. Yunnan Forestry Technology. 1997;(1):66-70.

8. Guo B, Lan D. Status of wildlife resources in Nangunhe reserve. Chinese Journal of Wildlife. 1999;(4):46-47.

9. Ma L. Research on structure of Asian elephant（*Elephas maximus*）community by using conservation genetics: Beijing Normal University; 2004.

10. Ling Q. Habitat Selection by Asian Elephant (*Elephas maximus*) in Nangunhe Nature Reserve, Yunnan, China [Masters]: Northwest University; 2007.

11. Fan Y, Li Z. Population genetic structure and population genetic diversity analysis based on mitochondrial DNA of Asian elephant Elephas maximus in Chhina. Acta Theriologica Sinica. 2012;32(2):90-100. <https://doi.org/10.16829/j.slxb.2012.02.002>.

12. Zhang L, Dong L, Lin L, Feng LM, Yan F, Wang LX, et al. Asian Elephants in China: Estimating Population Size and Evaluating Habitat Suitability. Plos One. 2015;10(5):e0124834. <https://doi.org/10.1371/journal.pone.0124834>. PMID: 25992617.

13. Yong-jing T, Zhi-sheng W, Gui-lian J, Xuan Z, Yuan H, Zheng-ling L, et al. Population dynamics analysis of Asian elephant in Nangunhe river basin. Forestry Construction. 2019;6:101-106.

14. Li W, Yu Y, Liu P, Tang R, Dai Y, Li L, et al. Identifying climate refugia and its potential impact on small population of Asian elephant (Elephas maximus) in China. Global Ecology and Conservation. 2019;19:e00664. <https://doi.org/10.1016/j.gecco.2019.e00664>.
