# Supplementary material for: Assessing population structure and body condition to inform conservation strategies for a small isolated Asian elephant (Elephas maximus) population in southwest China
Source: PLoS One. 2021 Mar 9;16(3):e0248210. doi: 10.1371/journal.pone.0248210 (PMC7942997; doi:10.1371/journal.pone.0248210)

S1 Dataset: A simplified database of Asian elephant in NNNR

Herd individuals

AE01: Juvenile male with c. 15-20 cm short straight tusks (estimated based on photographs and videos). Left lope is longer than right's, and ear upper folded slightly. Both-sides tail brush but not both-continuous in the end. BCS =4. See the following pictures.

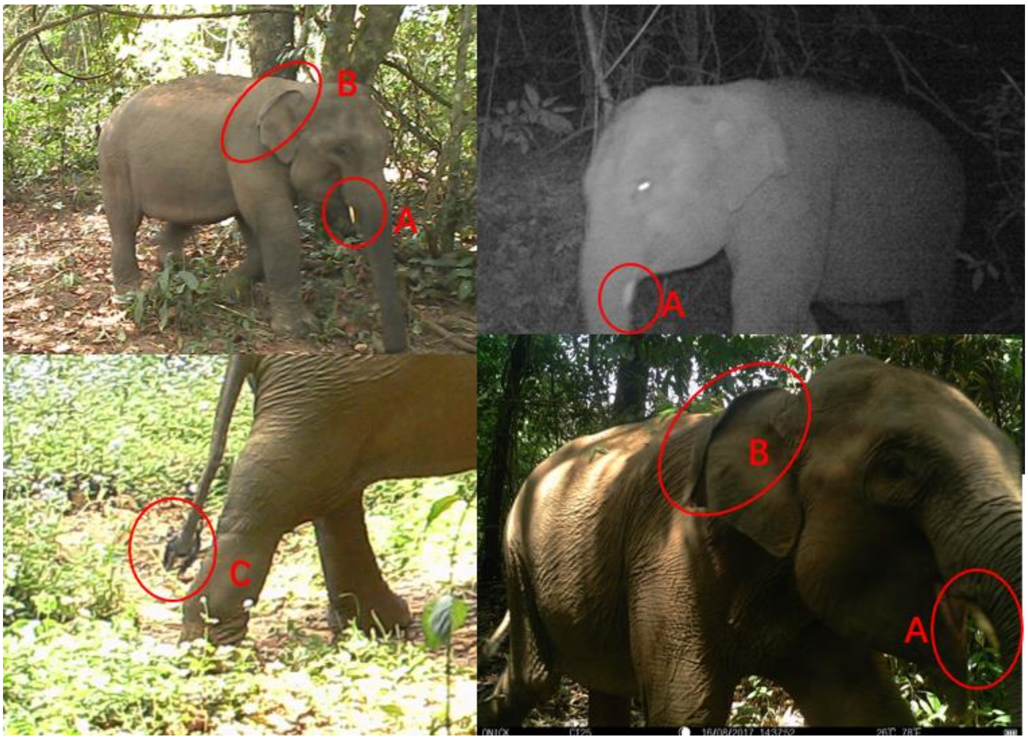

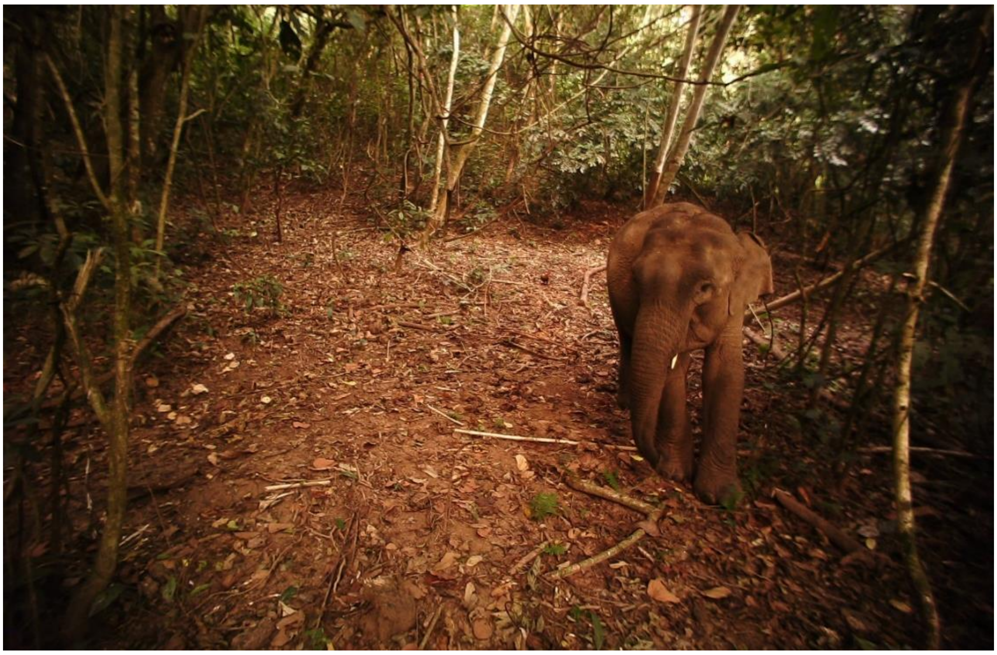

AE02: Adult female with c. 5 cm tusks which hard to see. There has a small hole down margin of the right ear, and there is no tear in left ear lobe, but both ears have serious fold up forward. A small black prominent obviously seen in the medium forehead. Standard tail brush with both sides and both-continuous. BCS =6. See the following pictures.

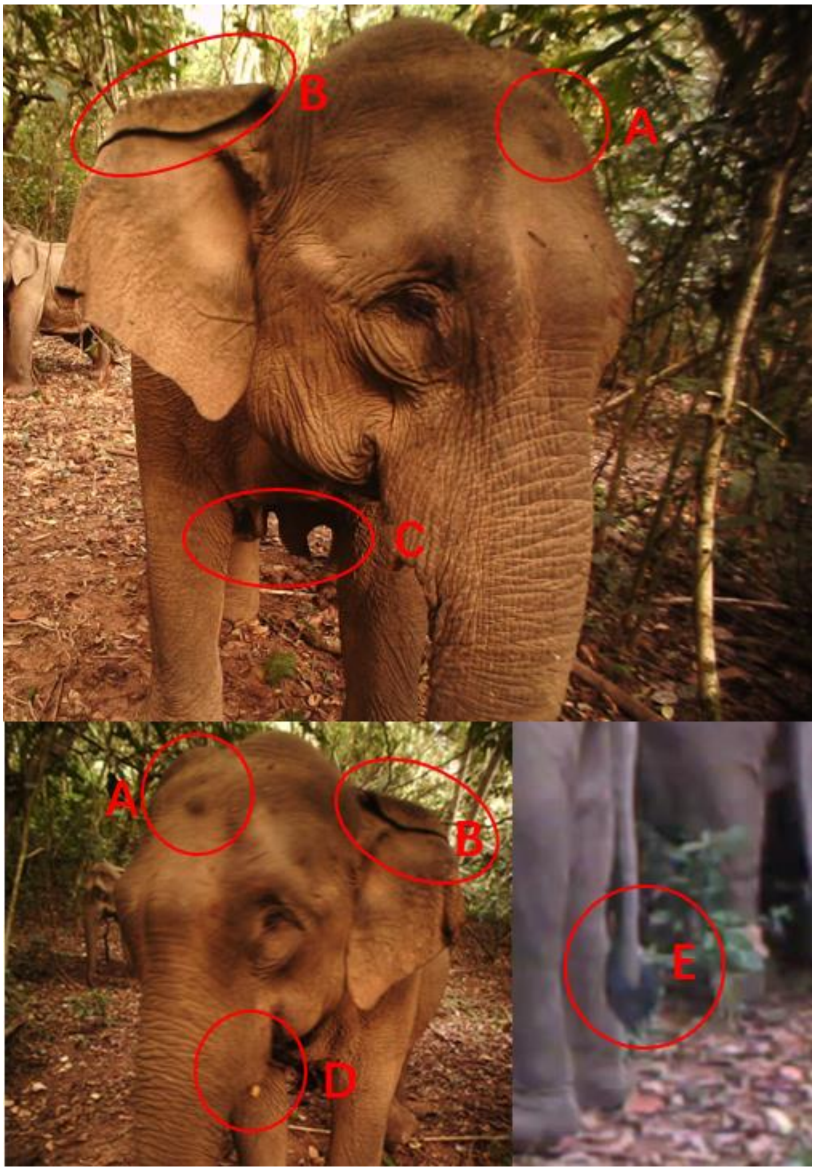

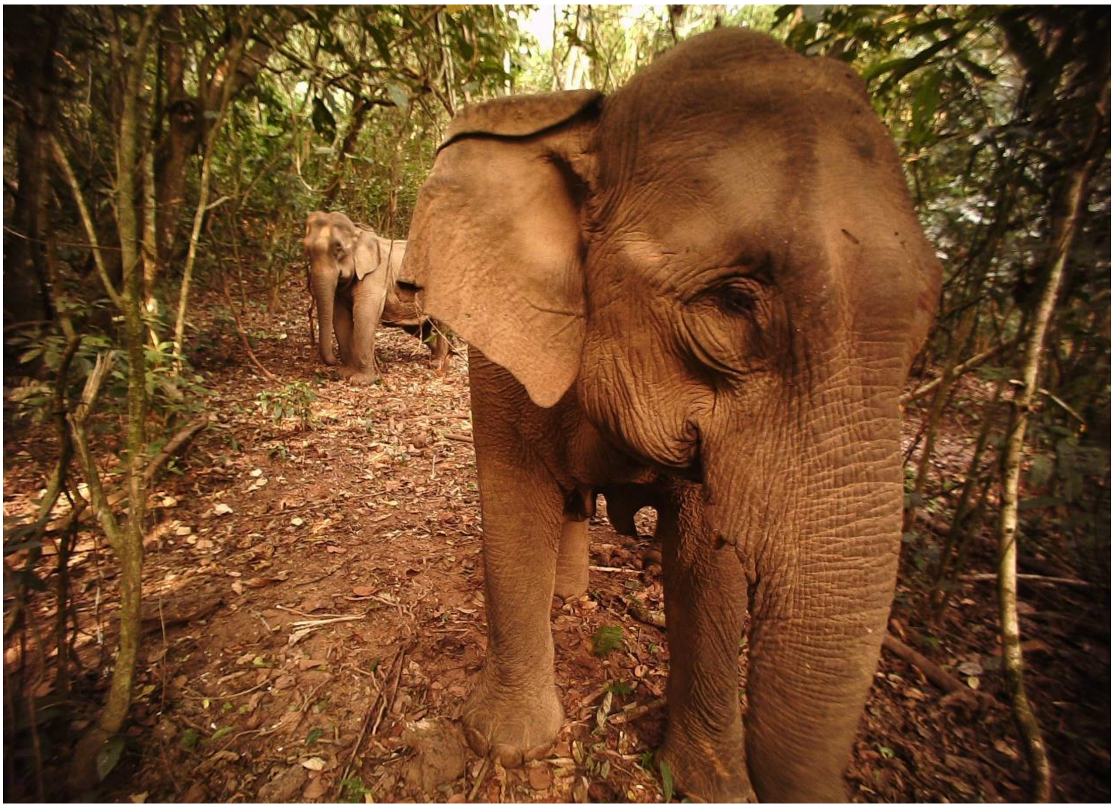

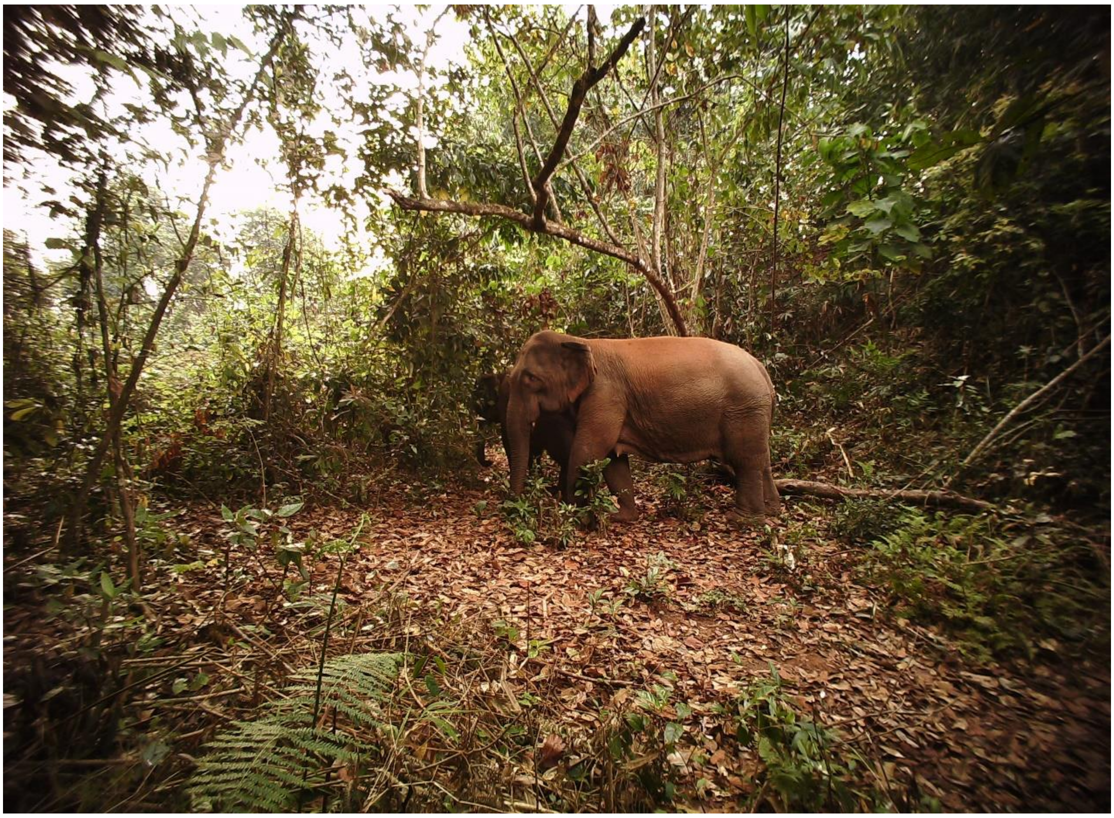

AE03: Adult female only with right tusk, there are lots of tears in right lobe, while left lobe is complete. Both ears upper fold seriously. The forehead can be seen with deeply concaved frontal ridges and they form a crater-like deep depression around the temporal region. Standard tail brush with both sides and both-continuous. BCS=3. See the following pictures.

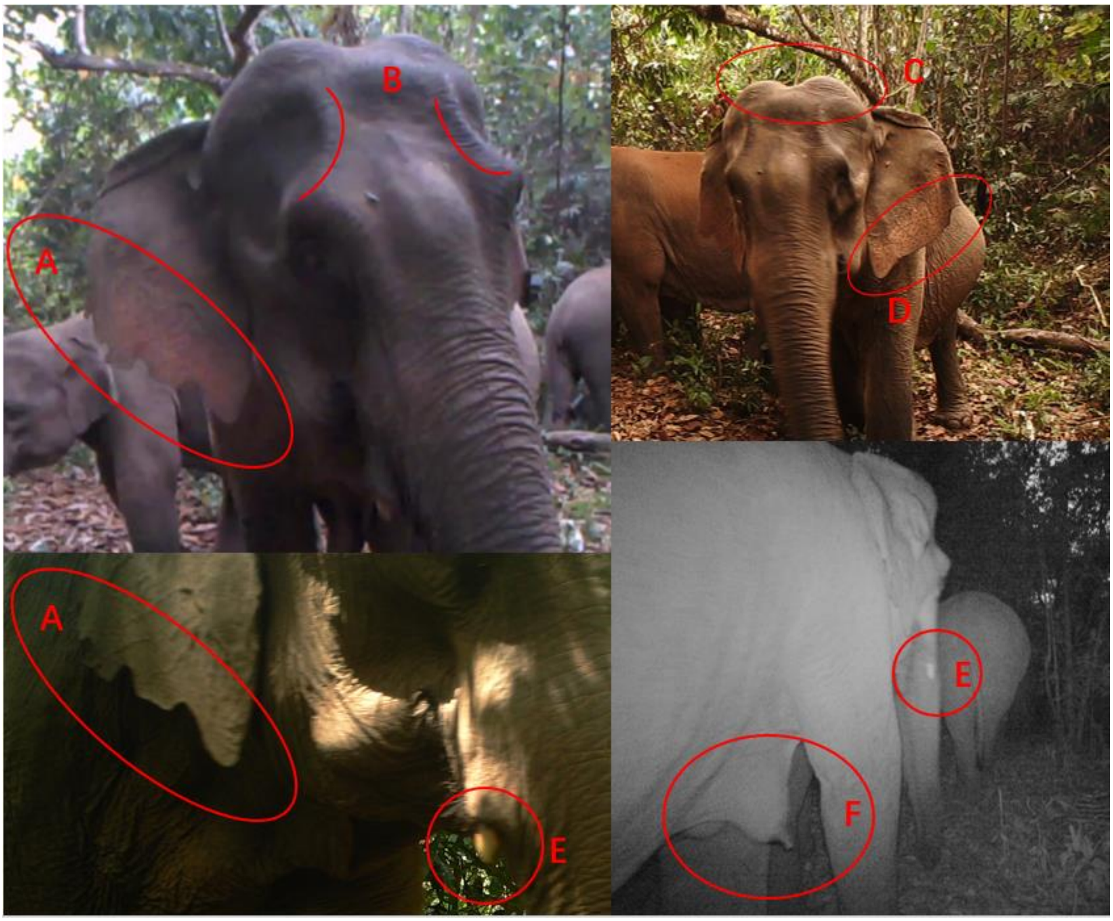

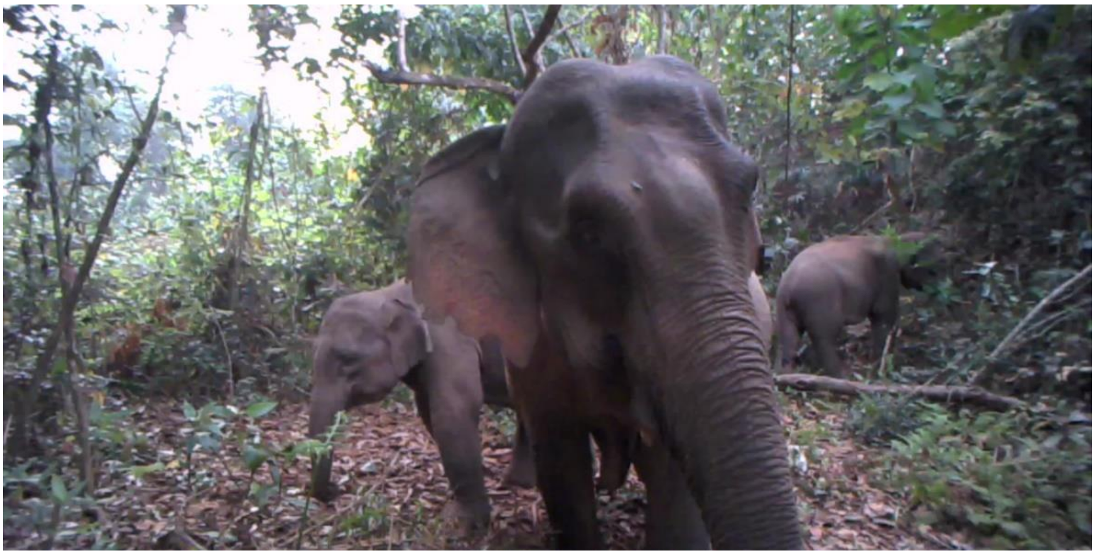

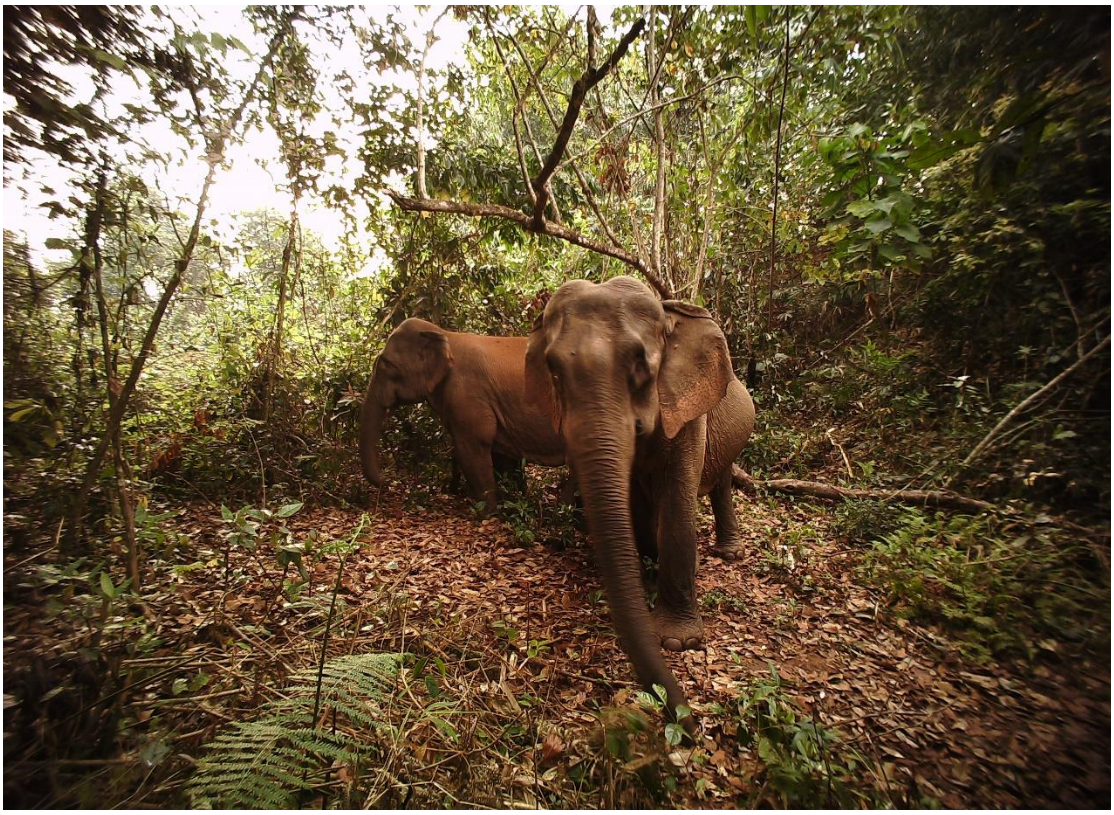

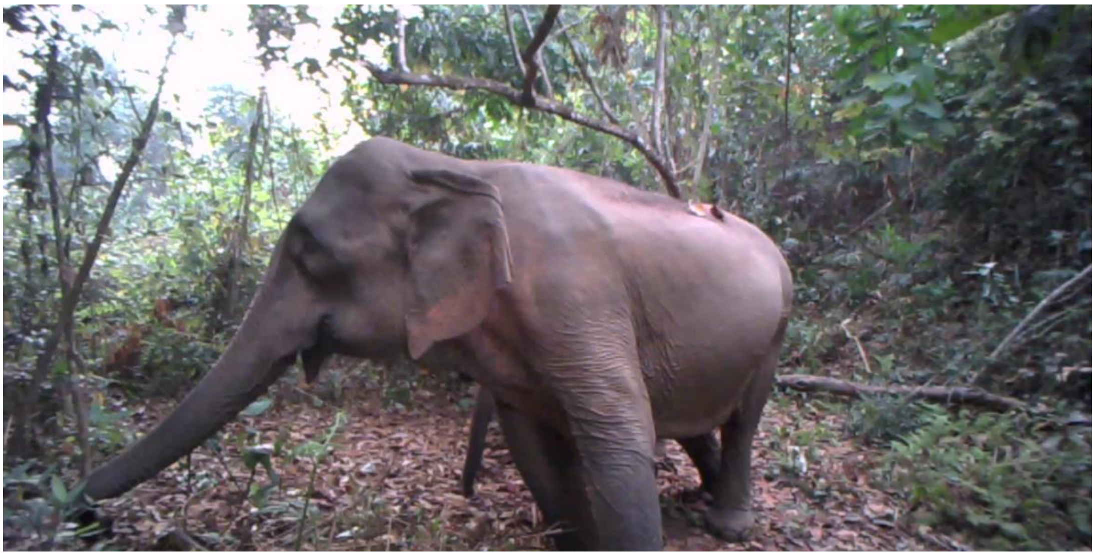

AE04: Juvenile female without apparent tusks, only can see a little white tip form left side. There are no tears on both sides ear, the upper ear has a waved-fold. Tail brush has not both-continuous. BCS=5. See the following pictures.

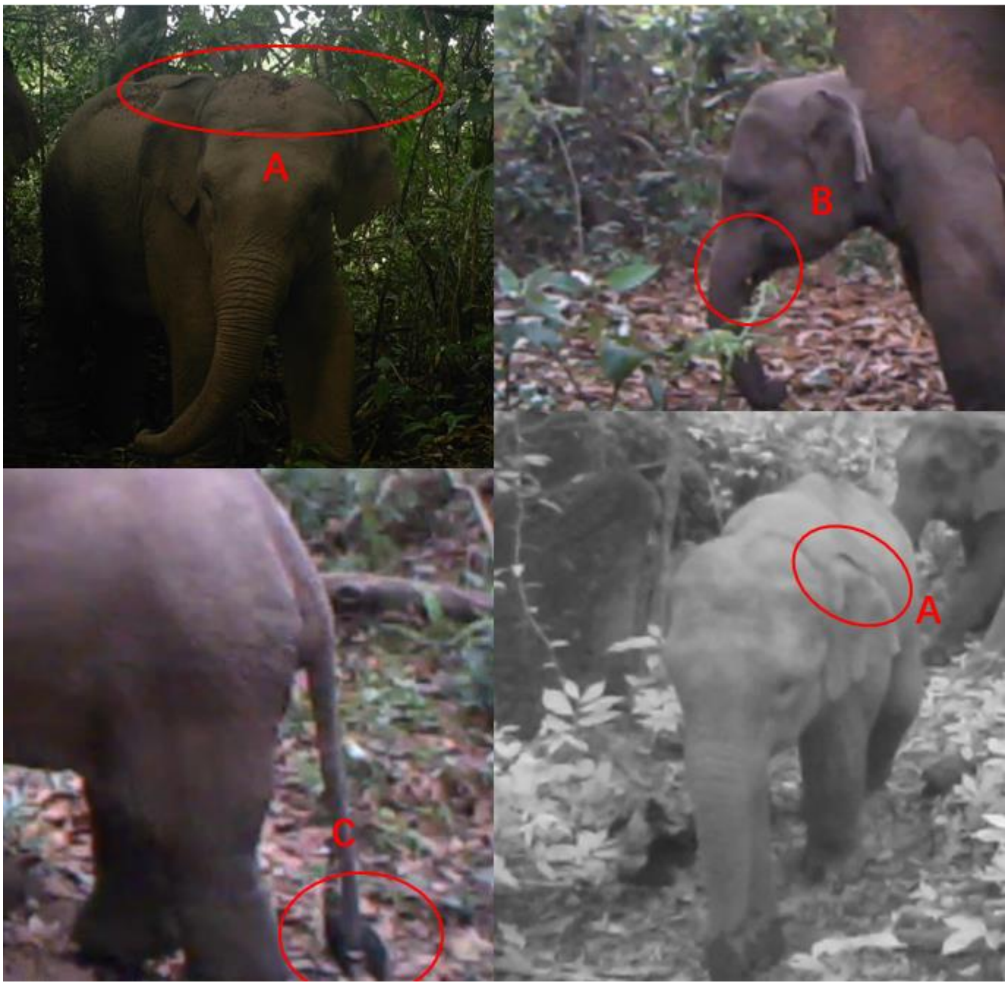

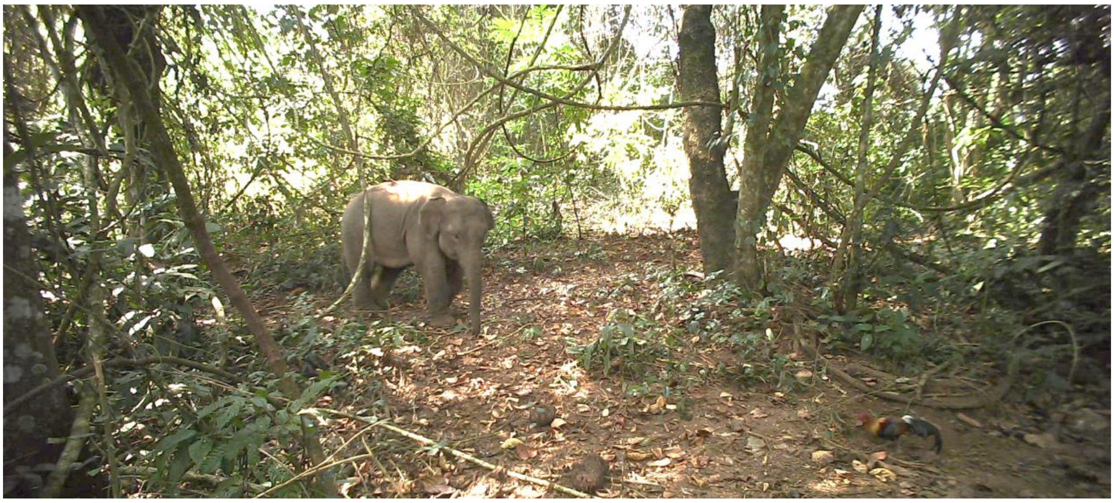

AE05: A calf male, whose mother is individual AE06. Penis was seen from a scene when it climbing. The brown hair was visible in head, both ear lobes were V-acuted, very little tail brush. BCS=8. See the following pictures.

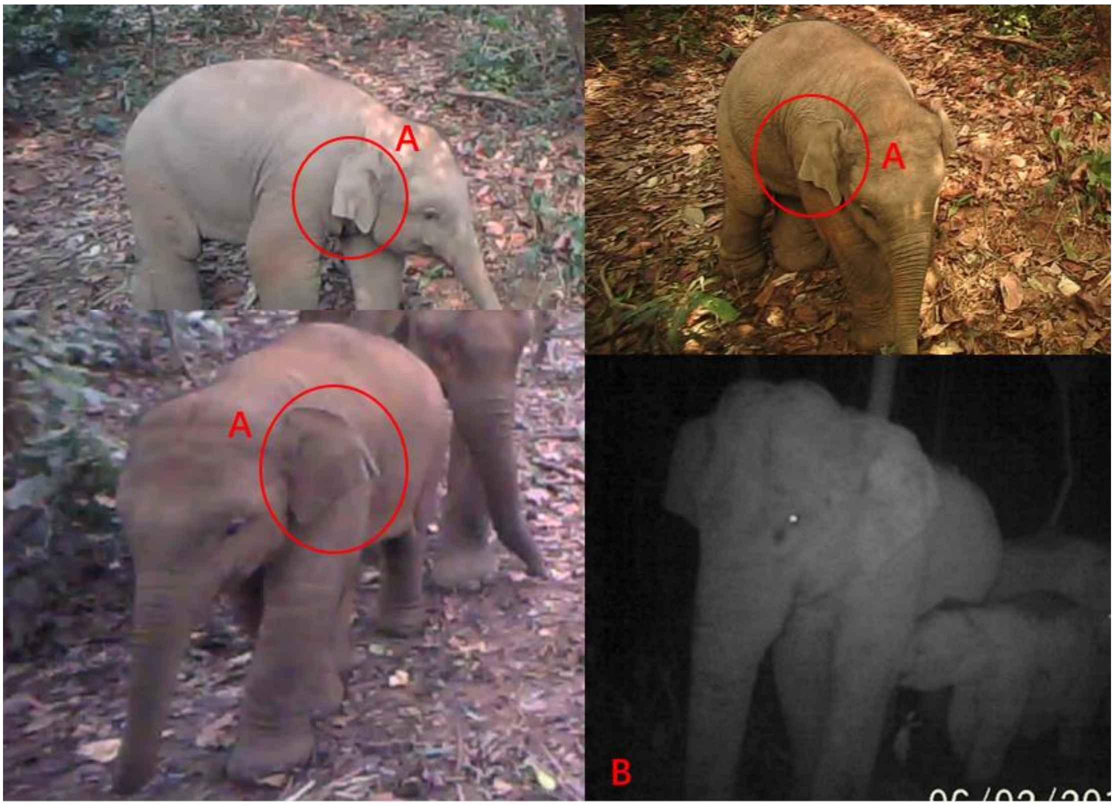

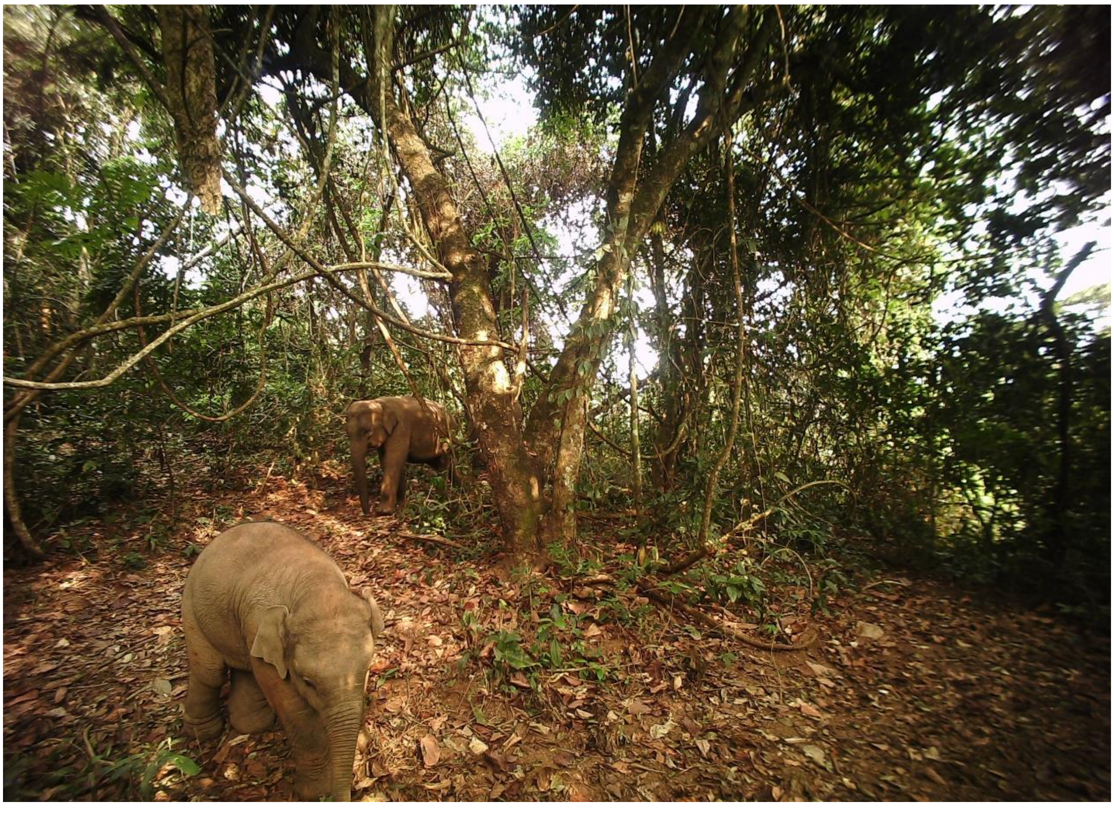

AE06: Adult female, with left-side tusk. Both ears have slightly fold at upper edge without tear in lobes, and right ear lobe shaped like V-actued, longer than left side. Little tail hair in both sides but dis-continuous. BCS =2. See the following pictures.

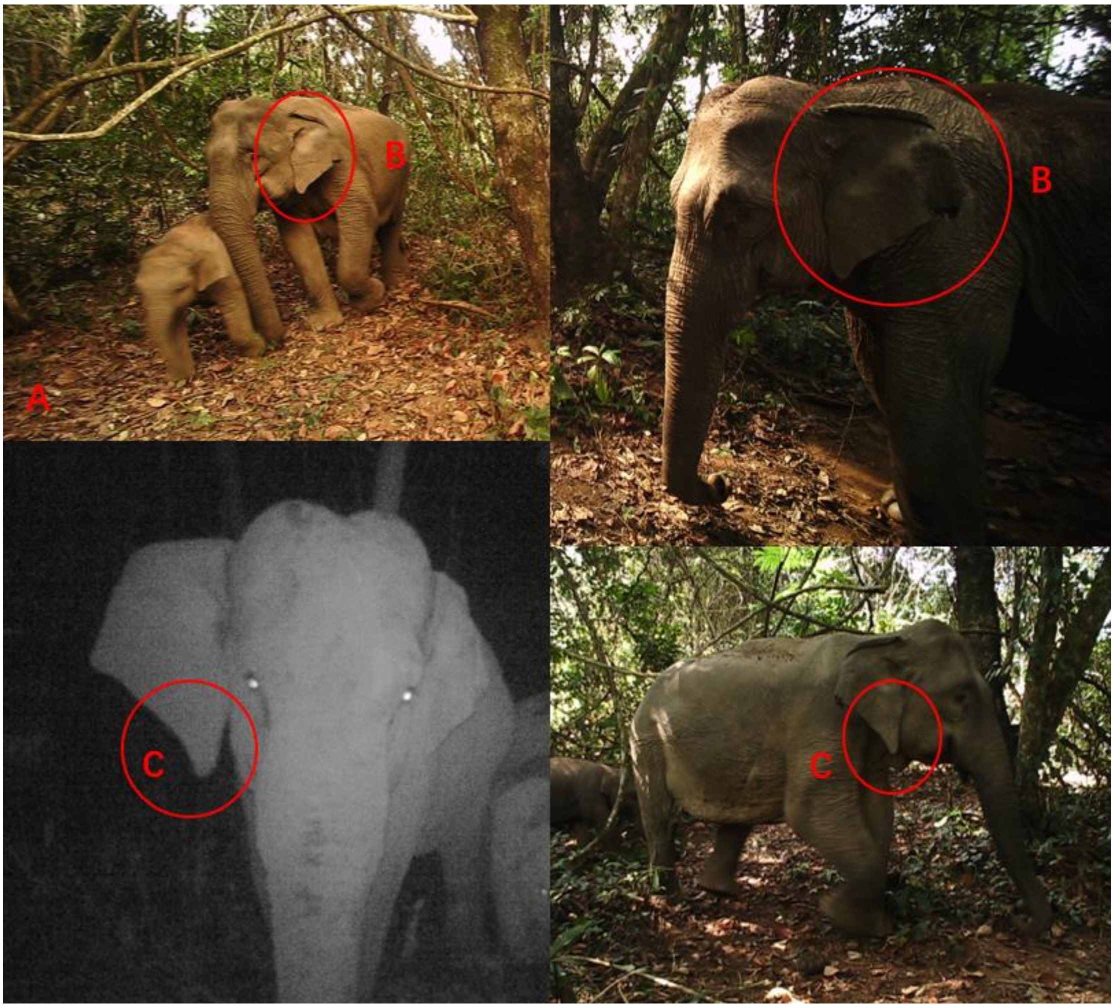

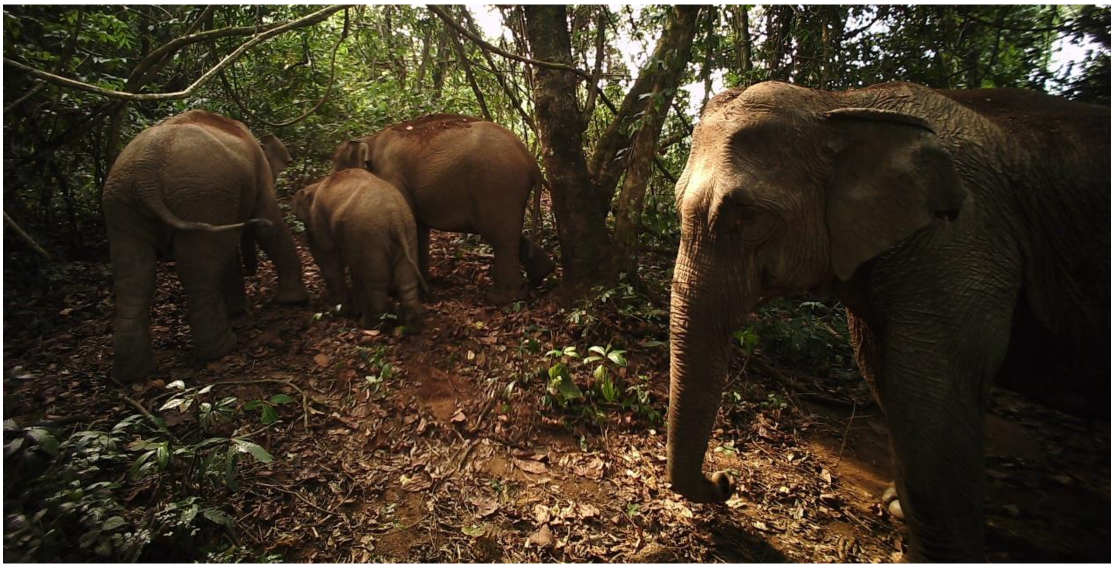

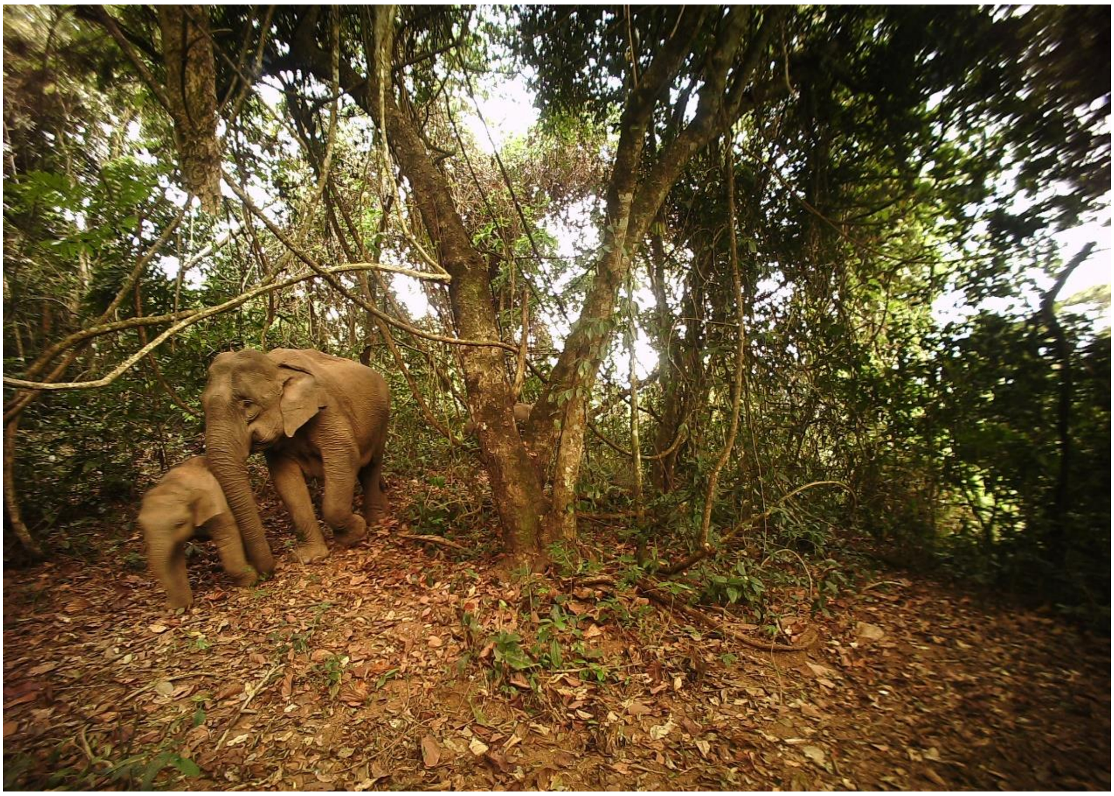

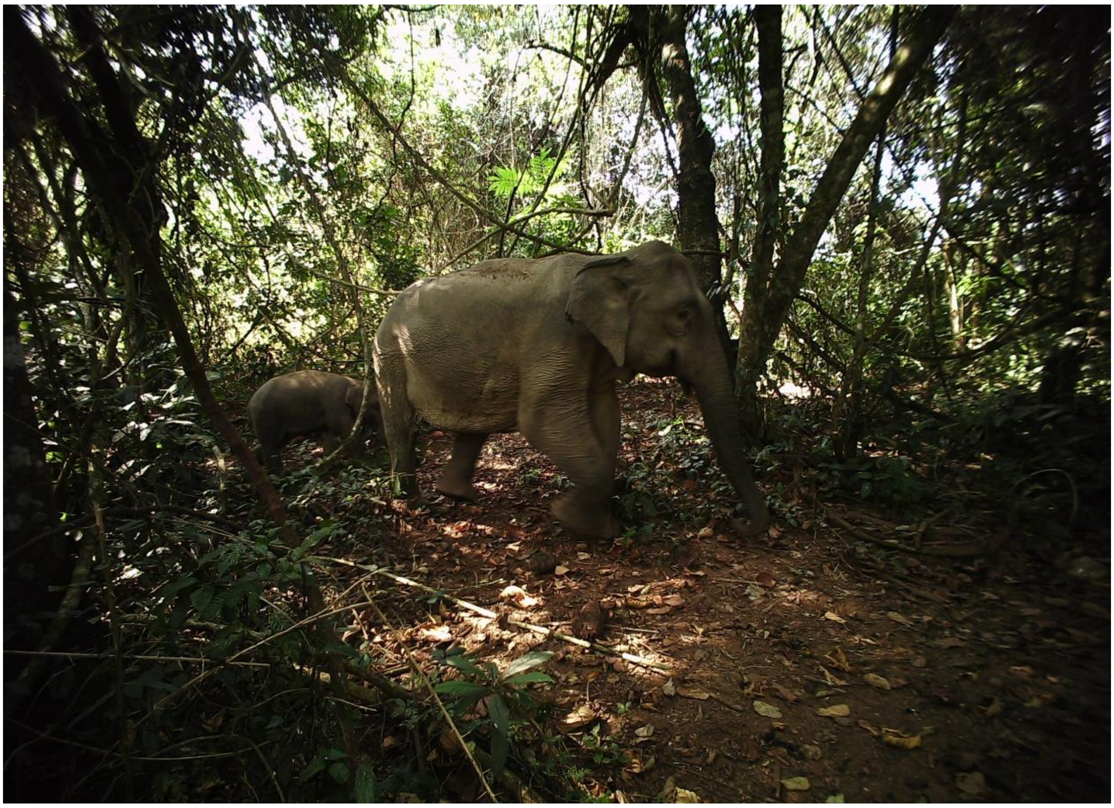

AE07: Juvenile female without both side tusks, has a similar body size with AE04. Unfold on both ears, little tail brush. BCS =7. See the following pictures.

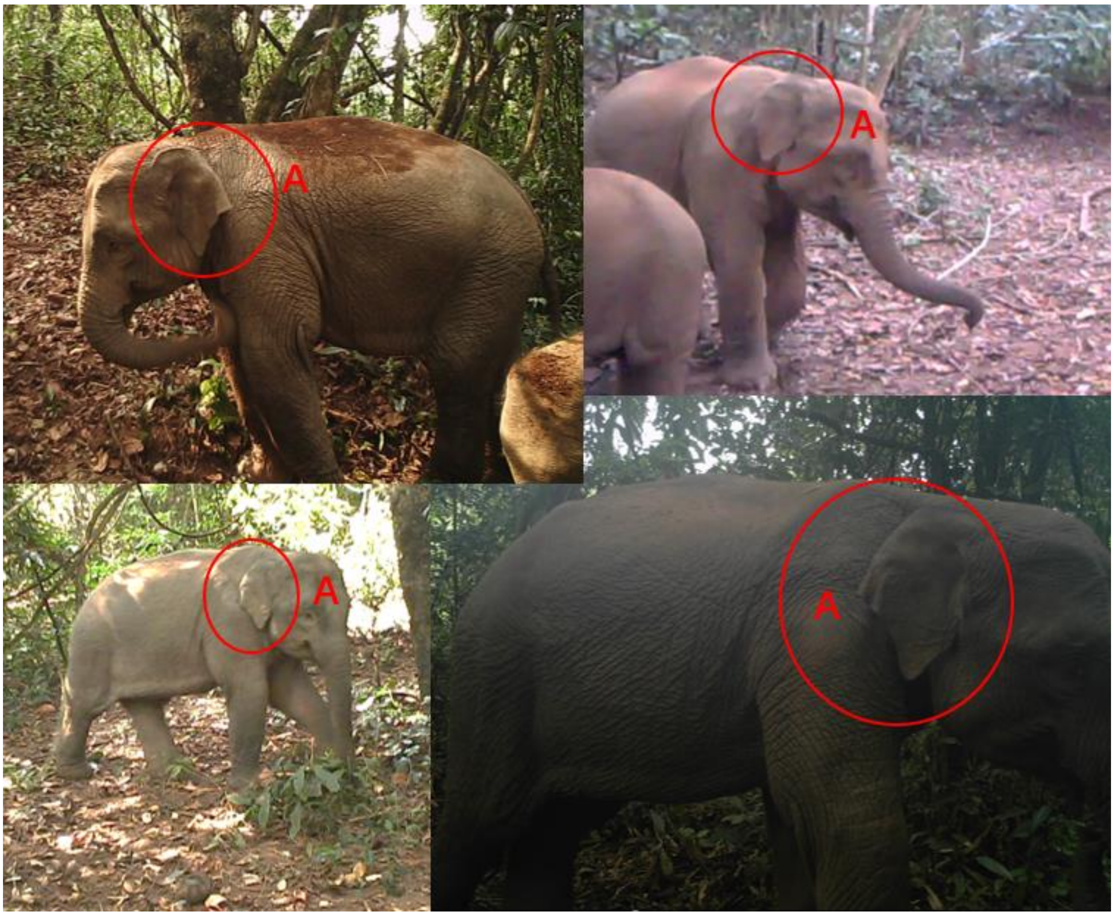

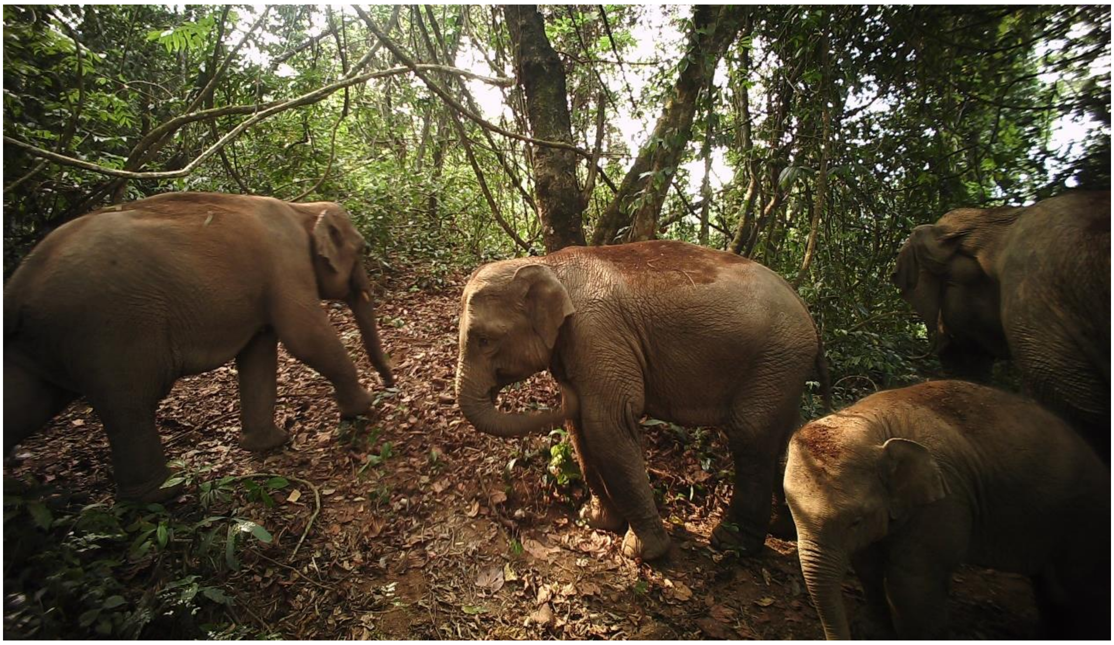

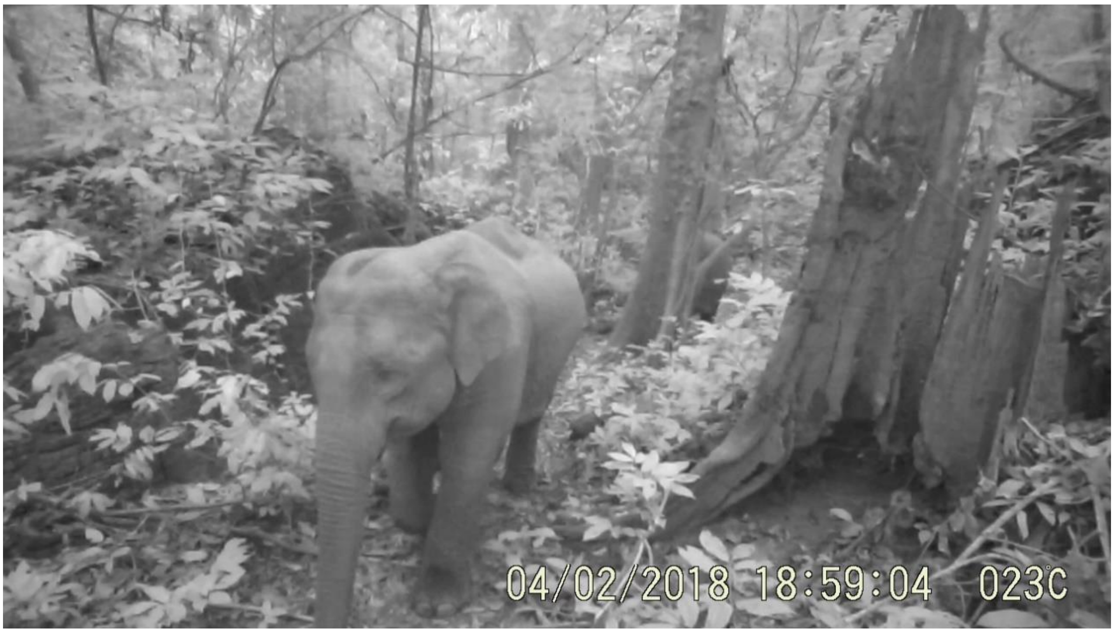
04/02/2018 18:59:04 023C

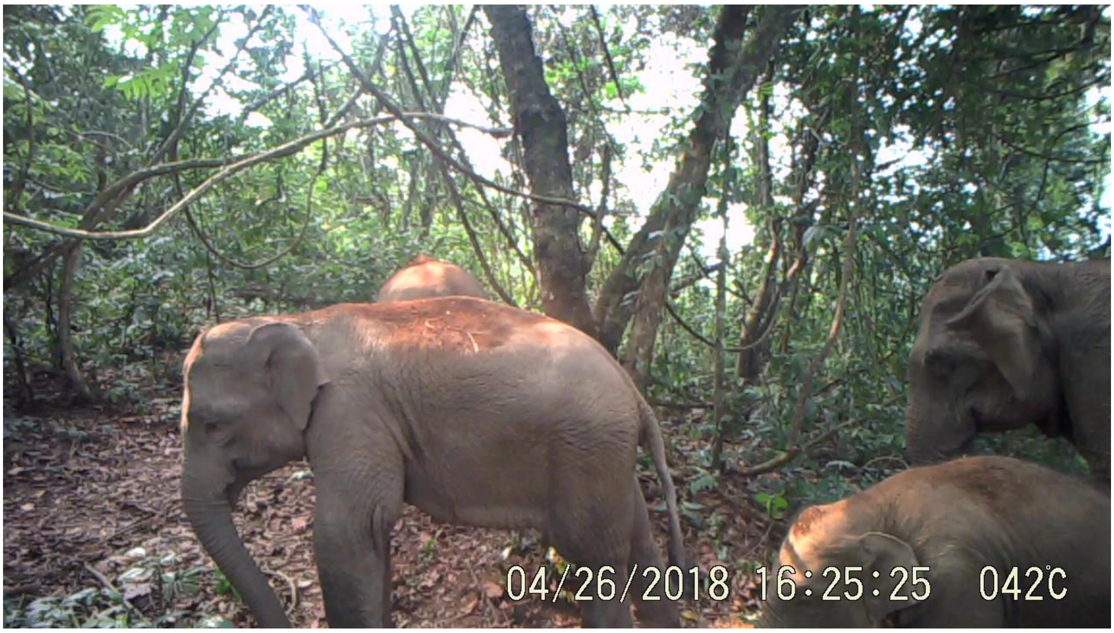
04/26/2018 16:25:25 042C

AE08: Juvenile male with c.30-40cm thin long curve tusks. The body size is similar with AE01. Both sides ear has wave-shaped fold and little tail brush. BCS=4. See the following pictures.

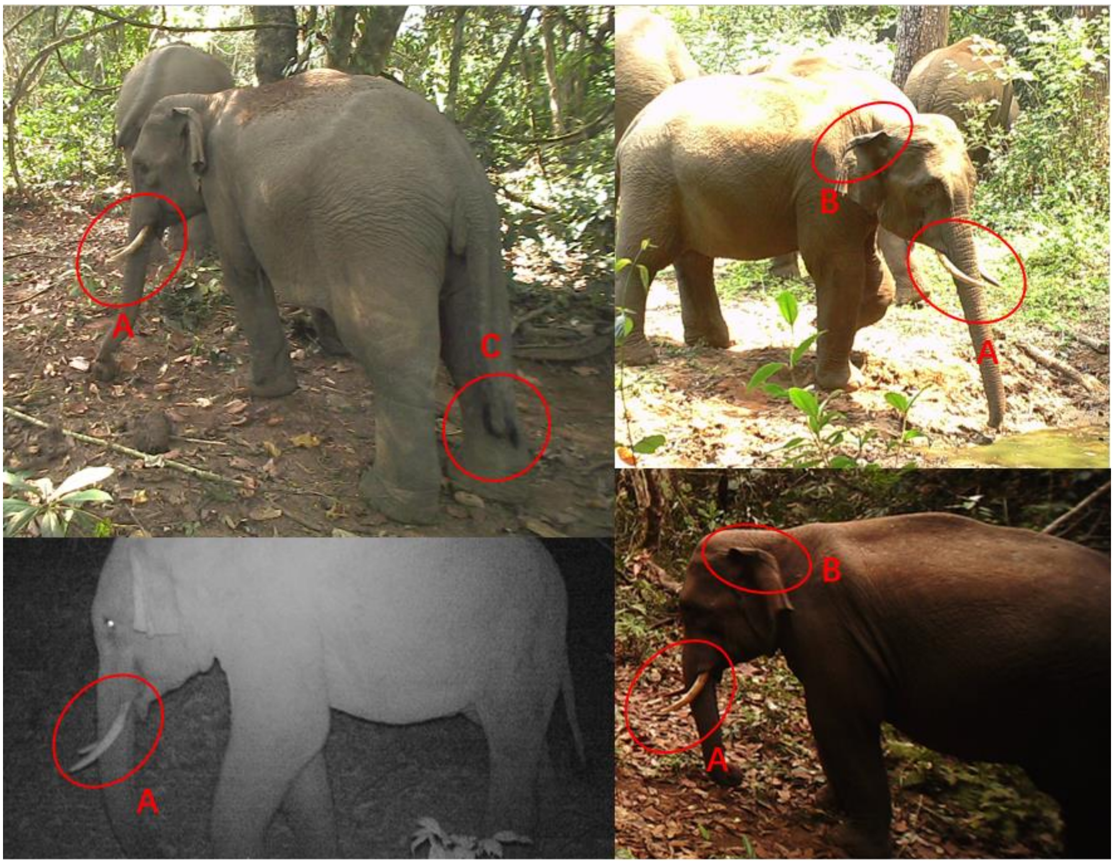

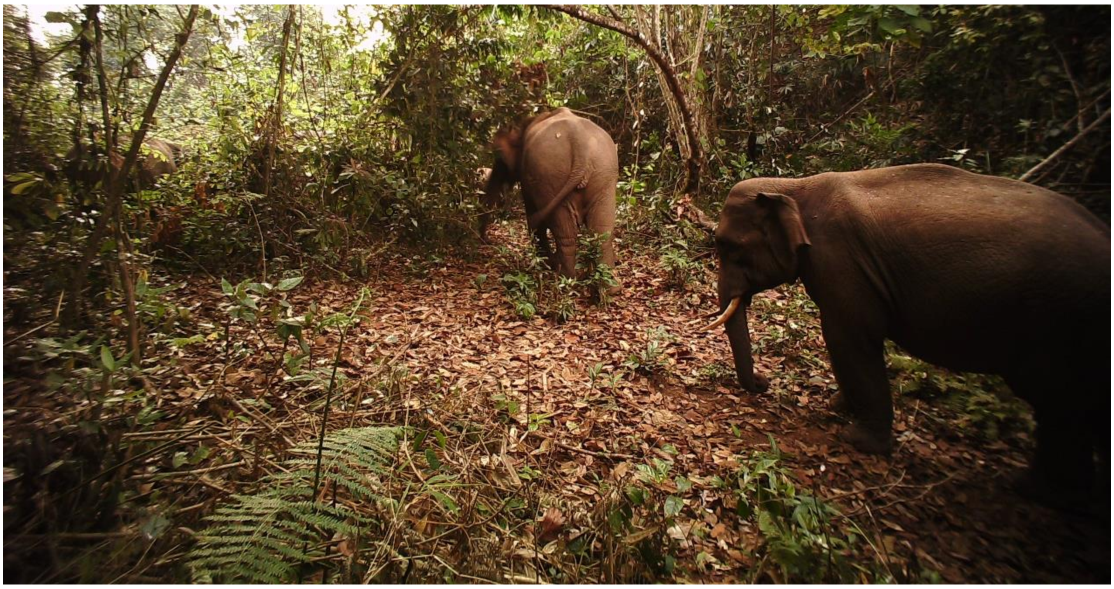

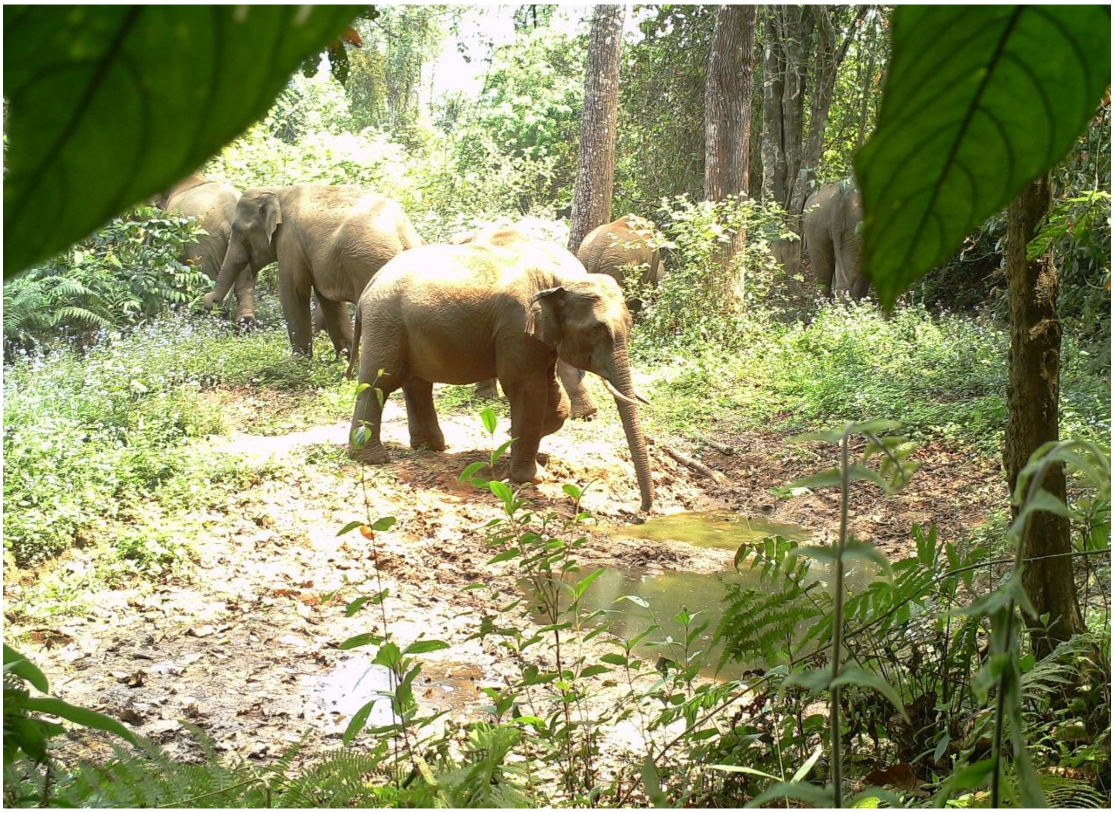

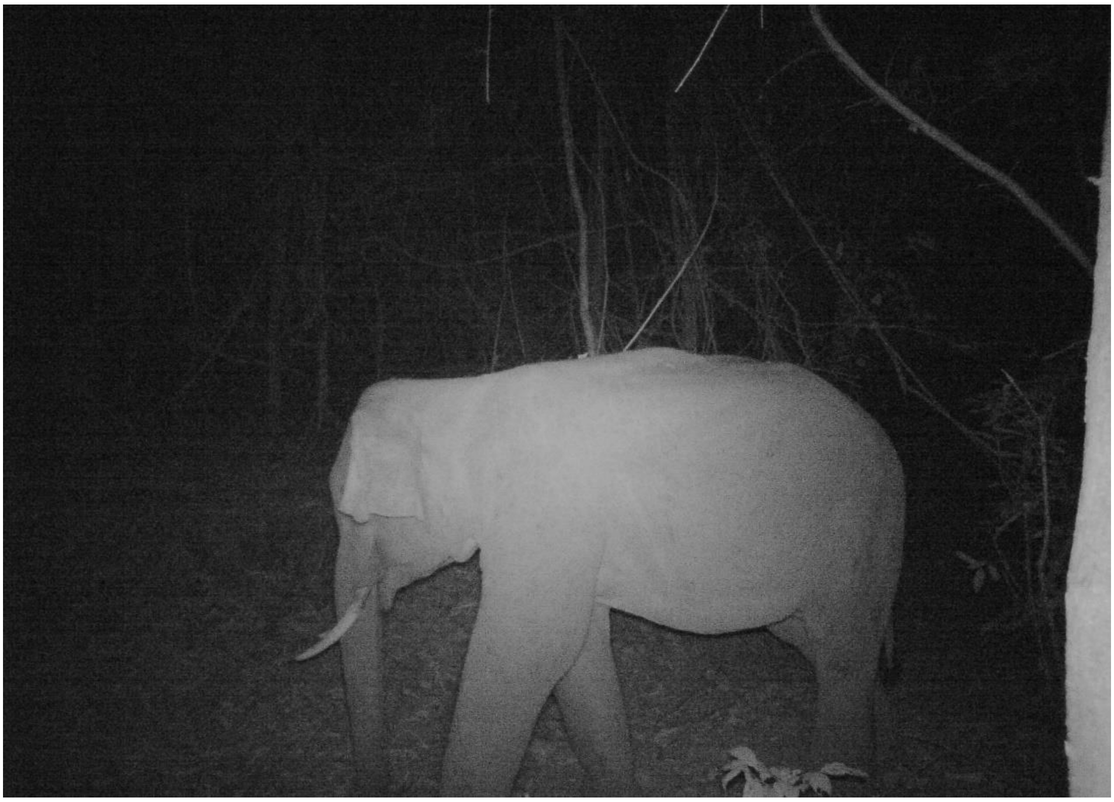

Solitary individuals

AE09: Subadult male, the body size is between juvenile and adult, without any tusks. No fold on both ears, and both sides lobe shaped like v-acuted. Little tail brush and dis continuous. BCS=4.

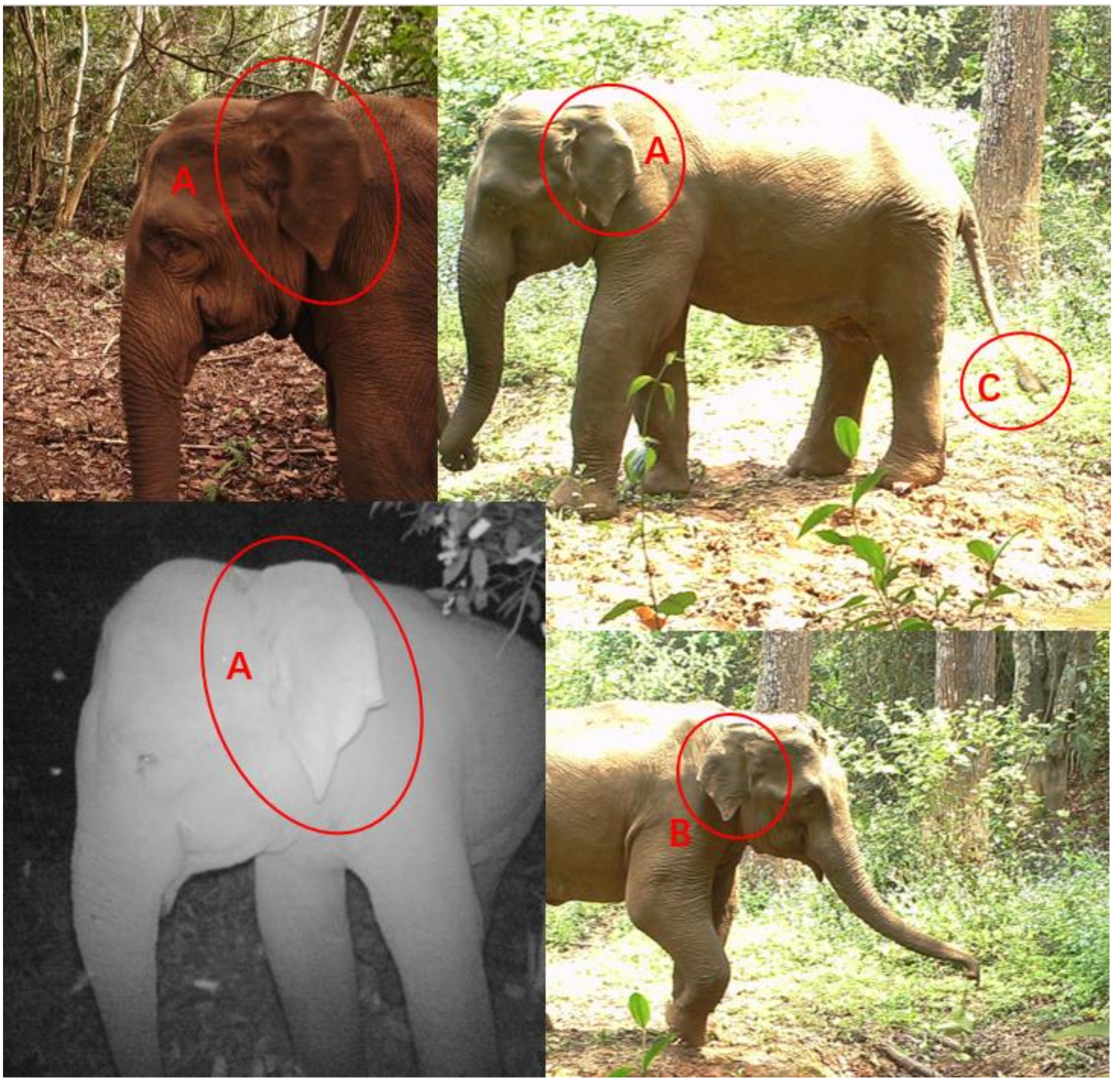

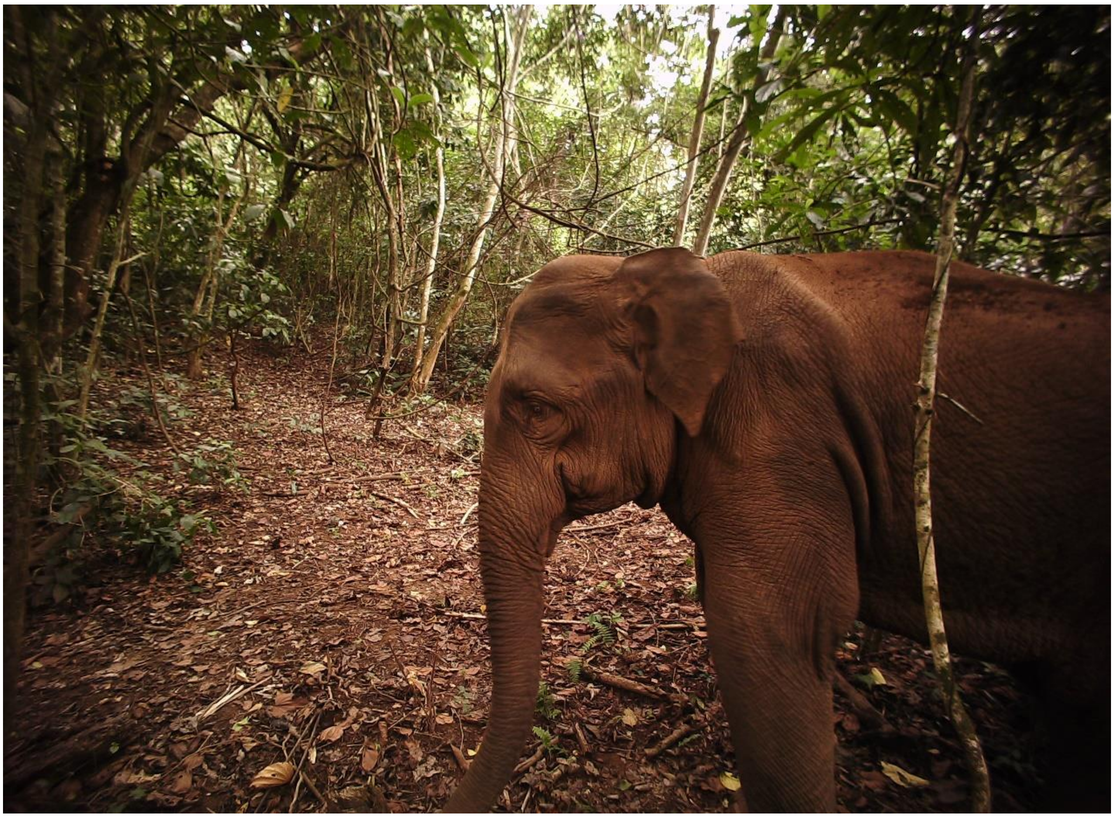

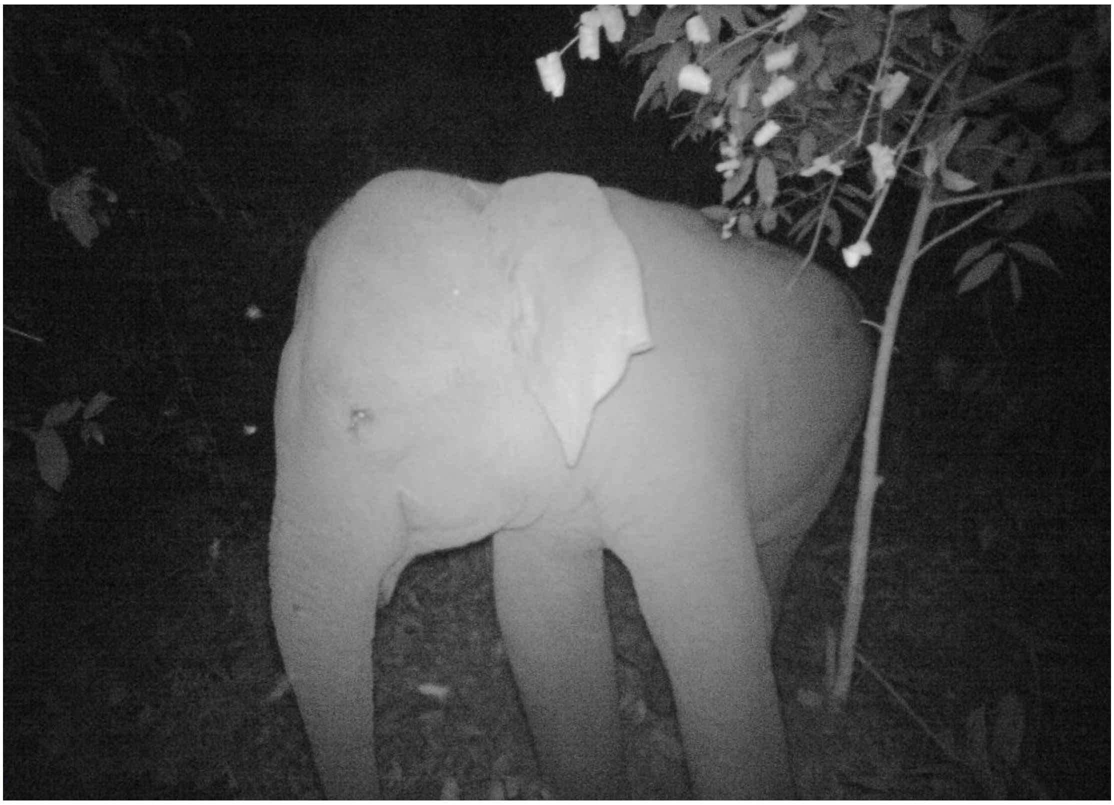

AE10: Adult male with long curve and thick tusks, there has a tear in left tusk. Both side tumors on board head, lots of tears on ears. The skin is rough and loose, and there has a flesh ring, the limbs are thick, only one side has little hair brush. BCS=9. See the following pictures.

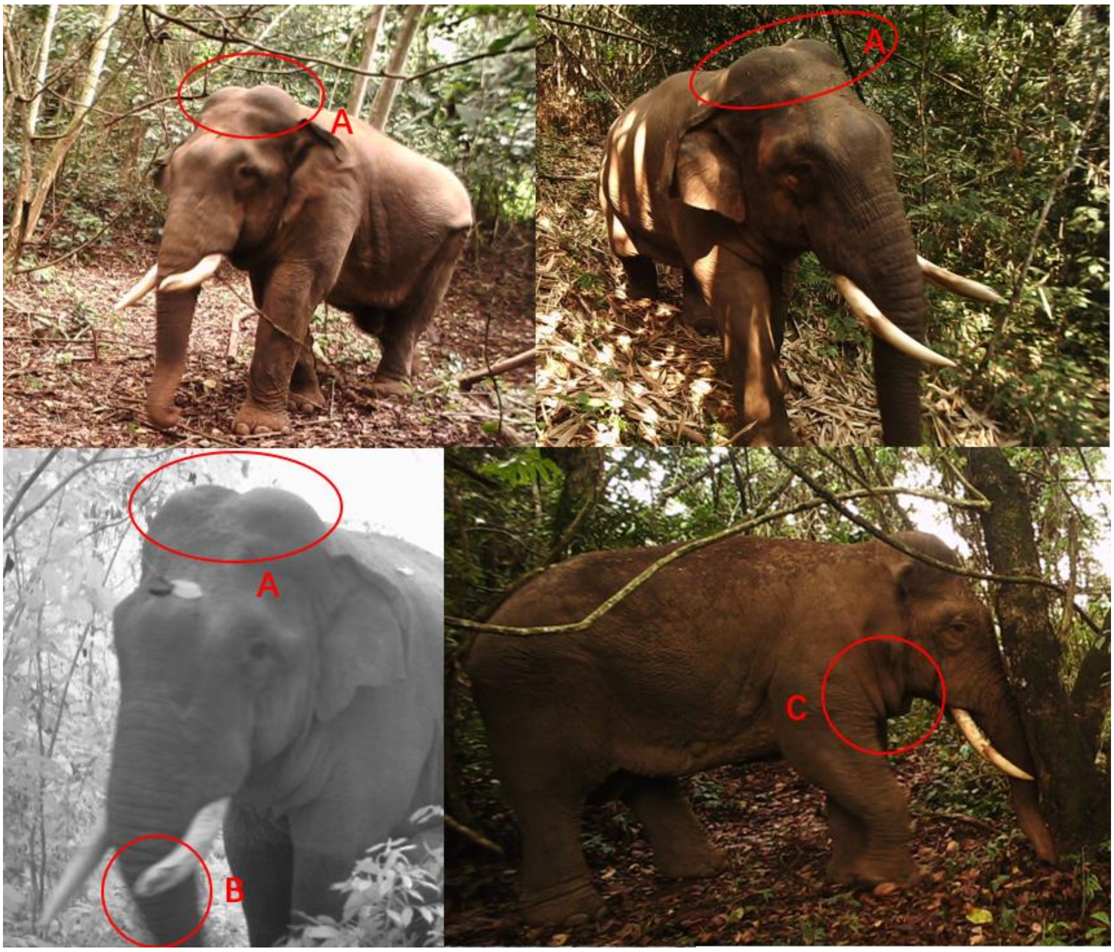

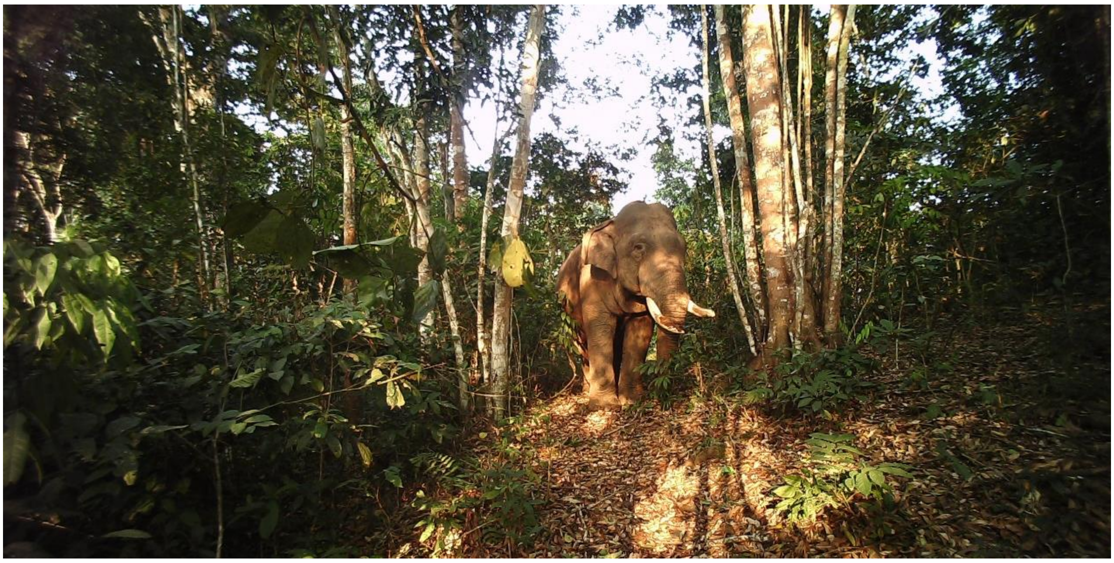

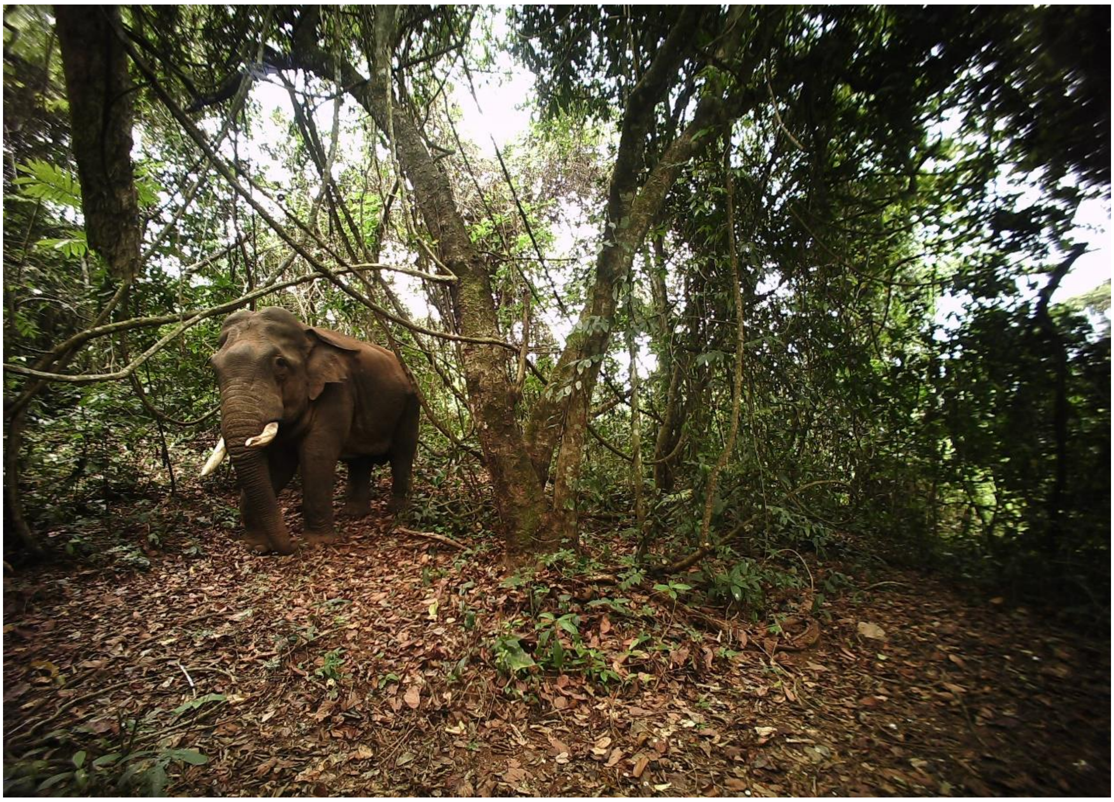

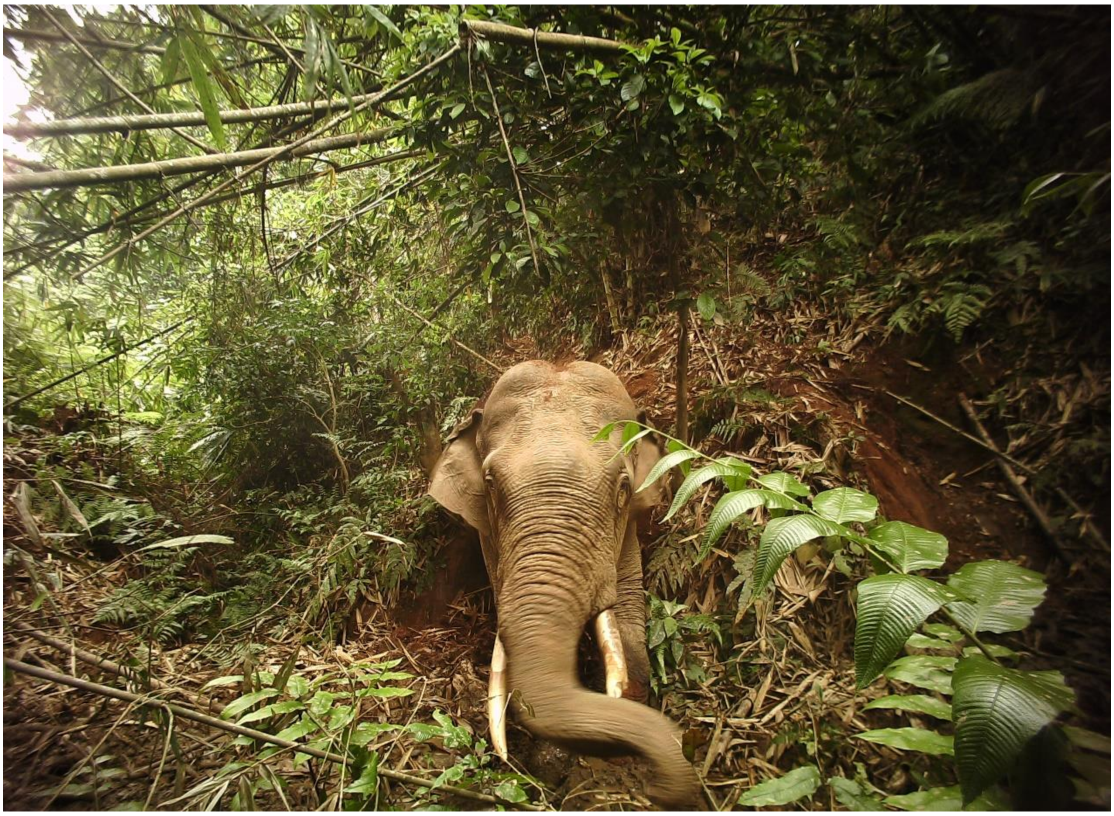

AE11: Adult male without any tusks, the body size is smaller than AE10, both sides ear has curve-shaped fold, there has an obvious tear in right ear, dis-continuous tail brush. BCS=8. See the following pictures.

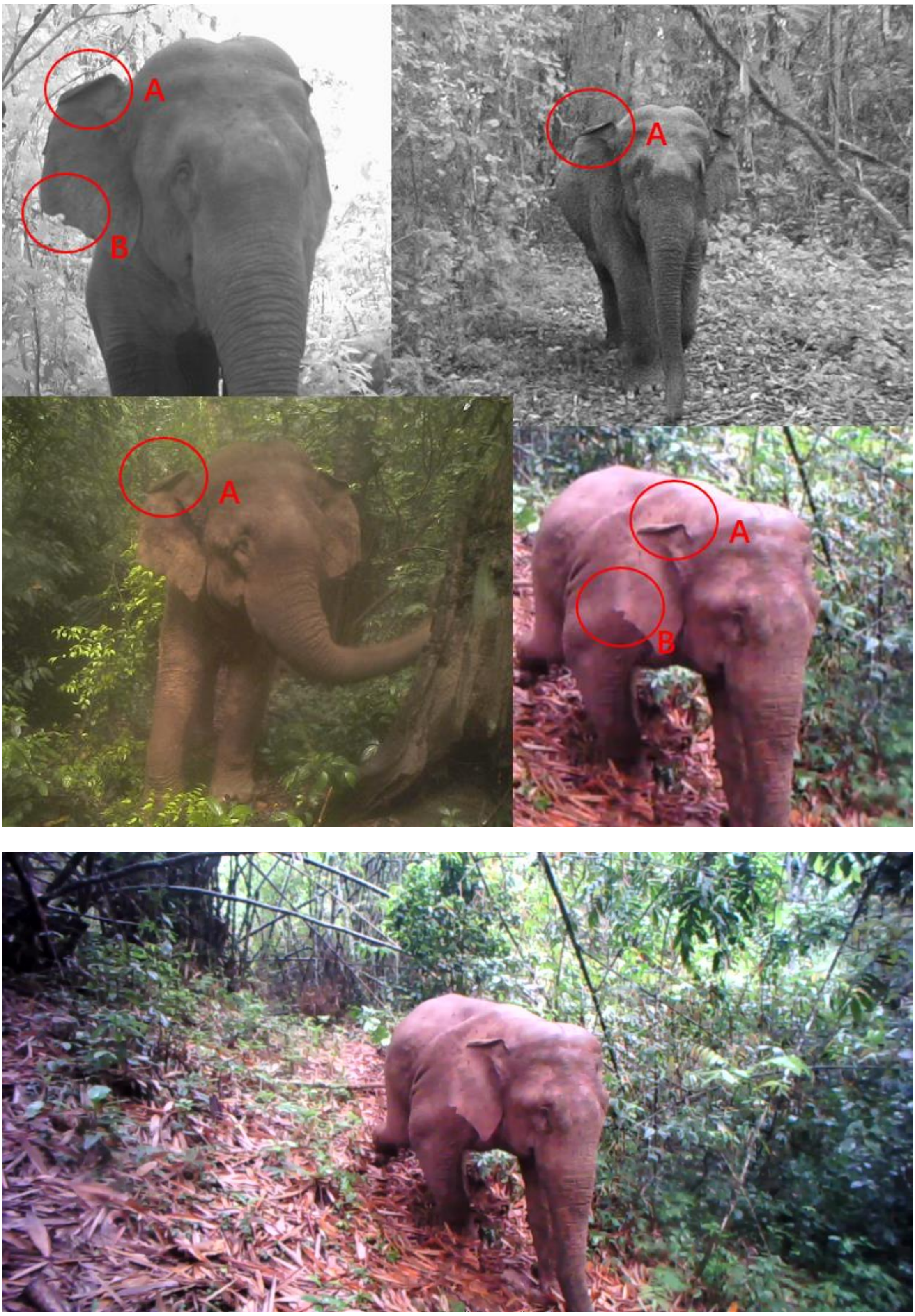

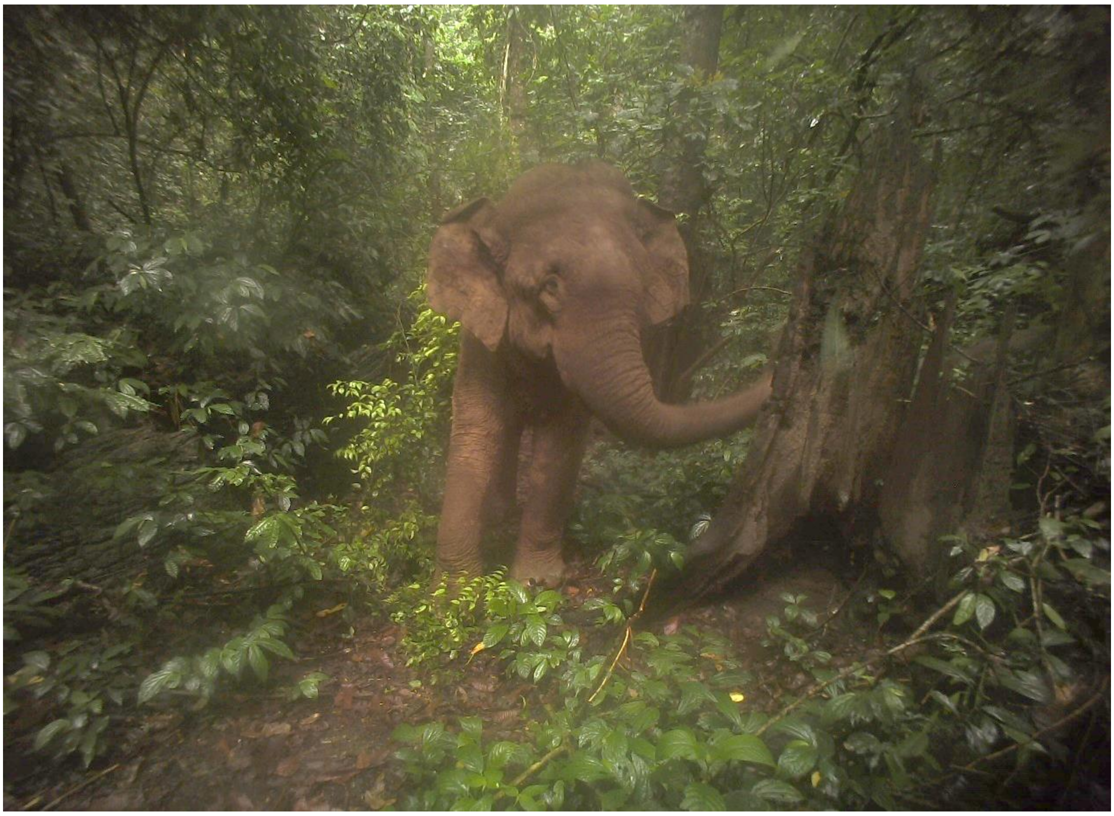

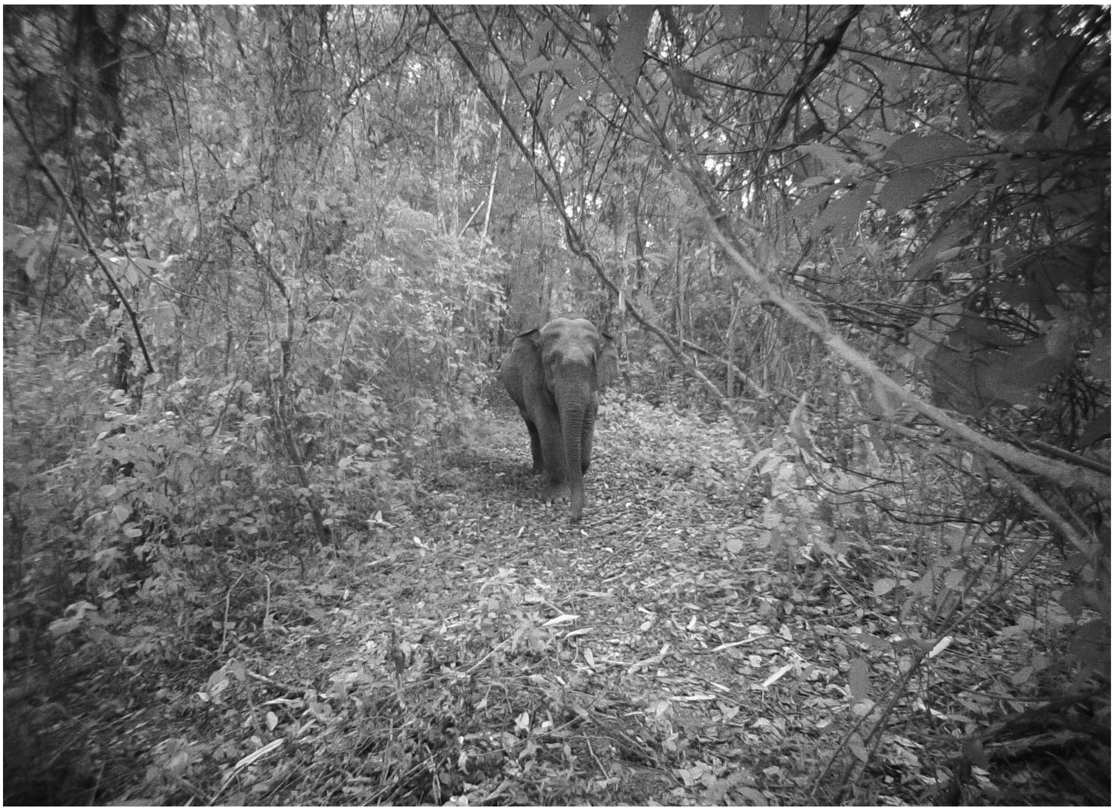

AE12: Adult male with very short tusk in left side, which is hard to see. The body size is similar with AE11, both sides ear lobes has no tears, standard tail brush. BCS=9. See the following pictures.

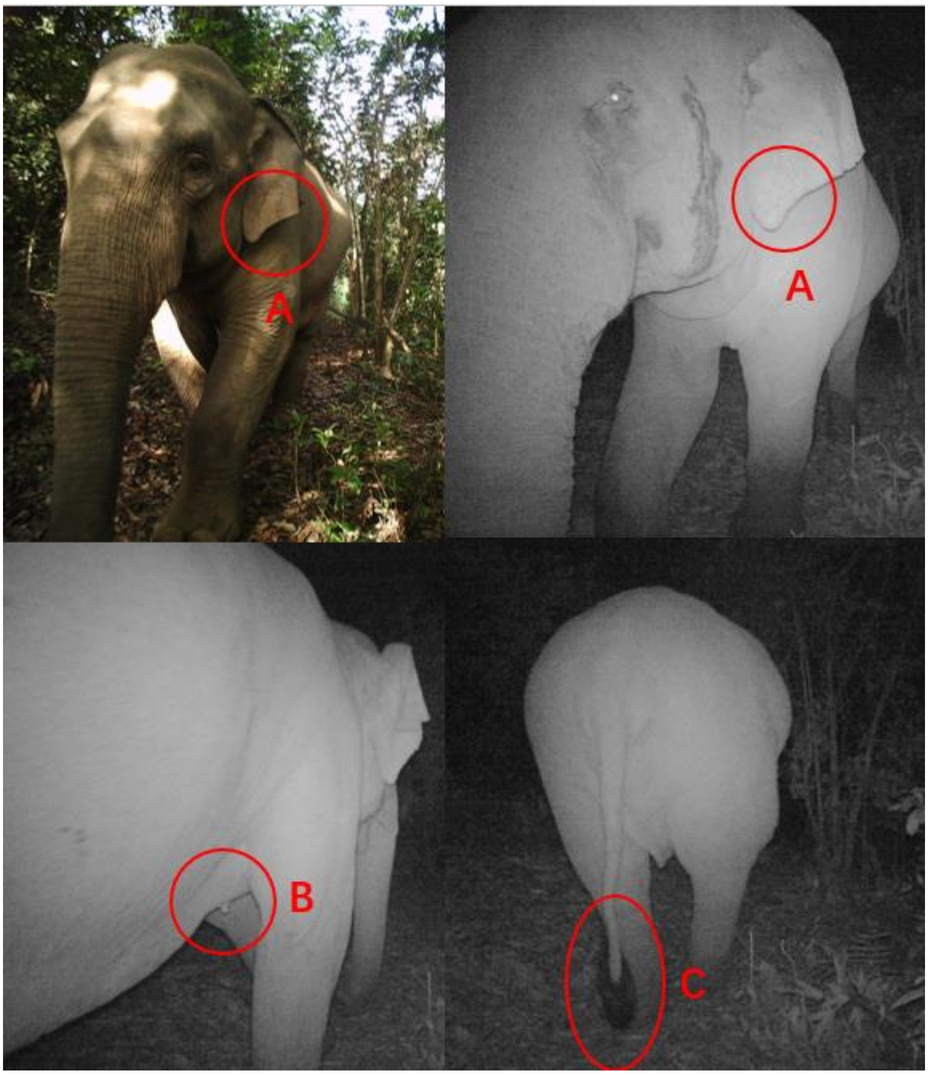

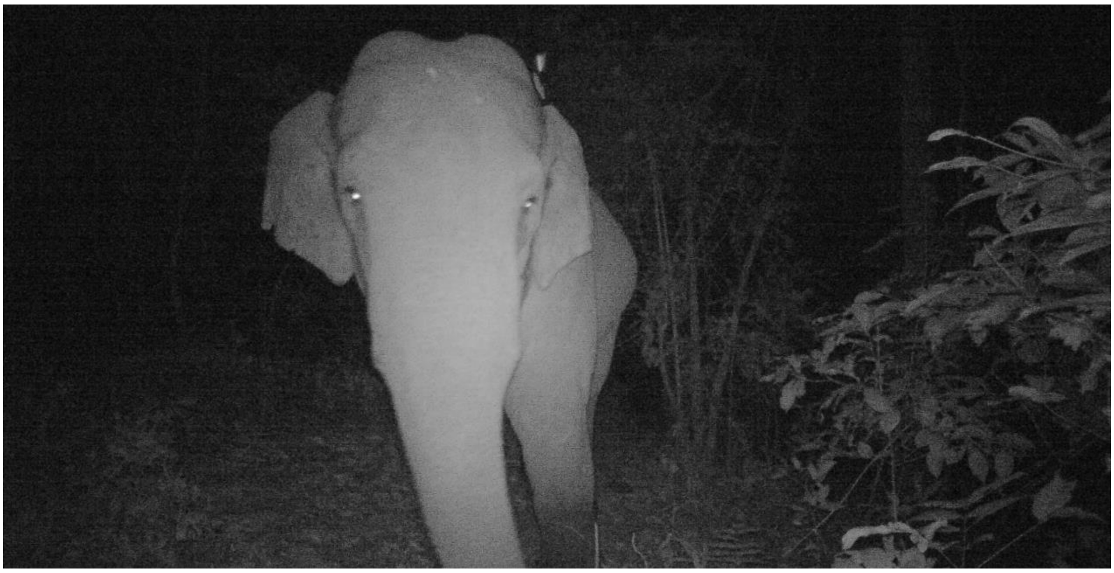

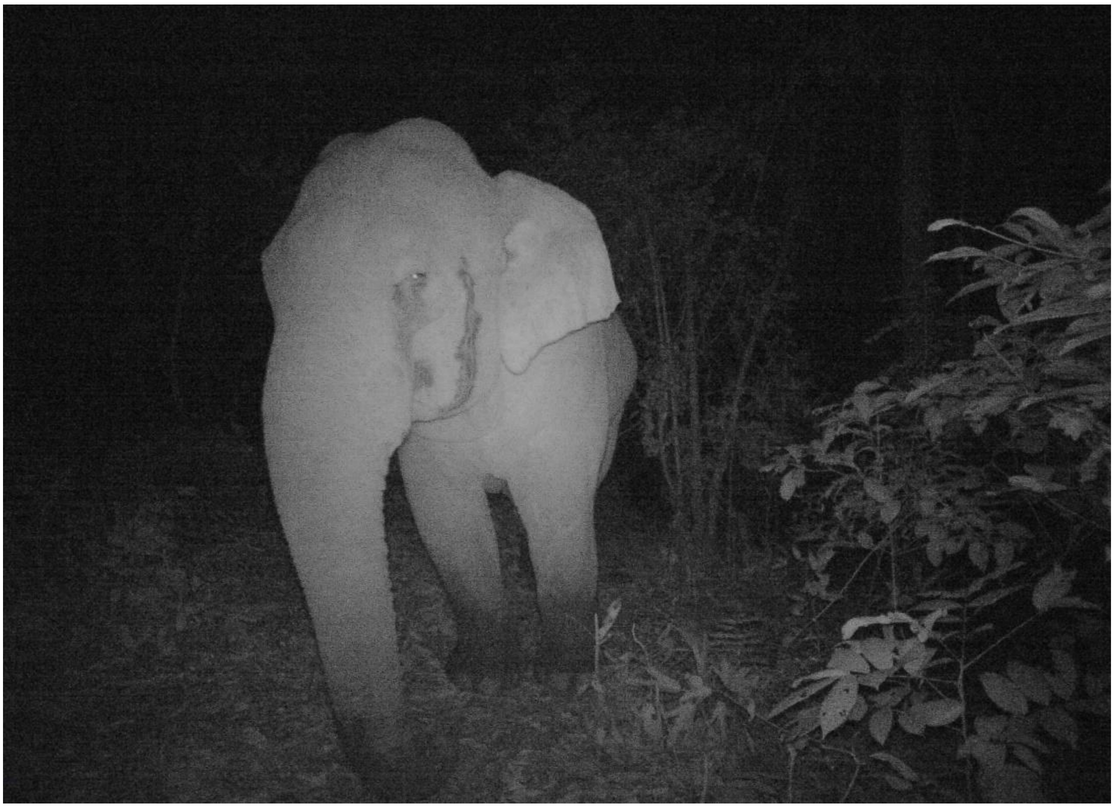

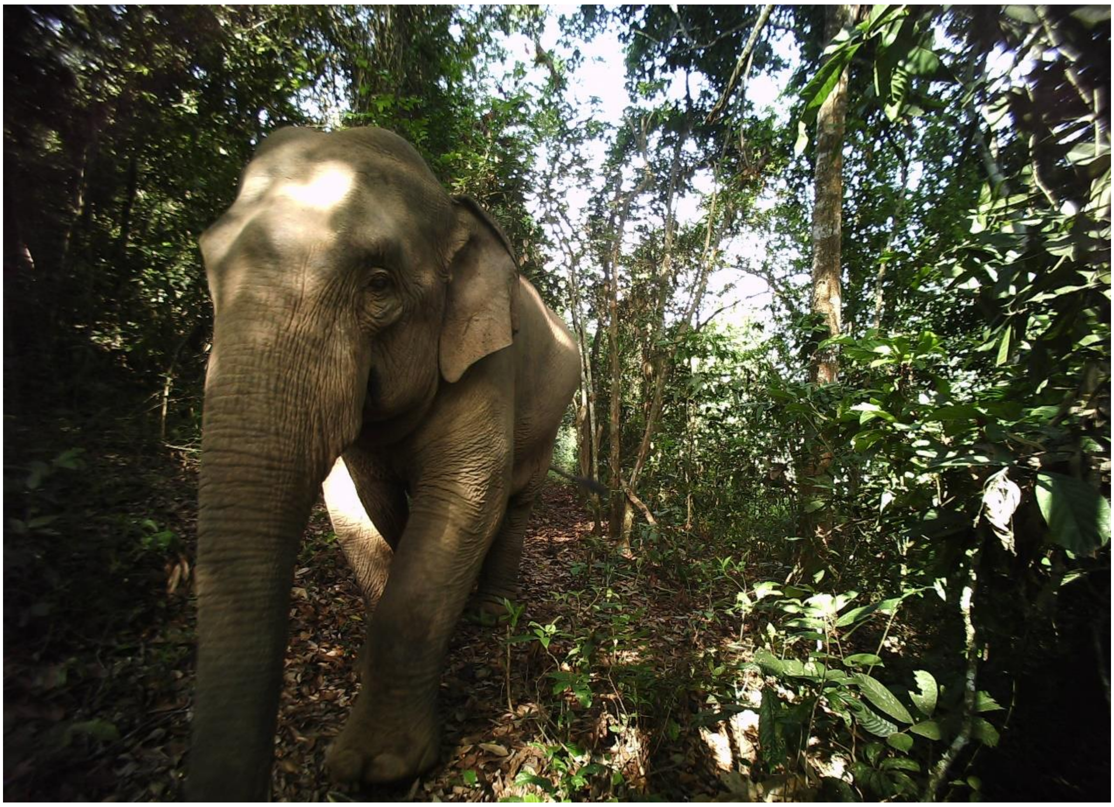

Supplement: S1 Dataset — (PDF) [file pone.0248210.s002.pdf]
