# Supplementary material for: Assessing population structure and body condition to inform conservation strategies for a small isolated Asian elephant (Elephas maximus) population in southwest China
Source: PLoS One. 2021 Mar 9;16(3):e0248210. doi: 10.1371/journal.pone.0248210 (PMC7942997; doi:10.1371/journal.pone.0248210)
Supplement: Attachment — Submitted filename: PONE-D-20-11590_reviewer.pdf [file pone.0248210.s003.pdf]

# PLOS ONE

## Population structure and body condition assessment to inform conservation strategies for a small isolated Asian elephant population in southwest China

--Manuscript Draft--

| | |
|---|---|
| **Manuscript Number:** | PONE-D-20-11590 |
| **Article Type:** | Research Article |
| **Full Title:** | Population structure and body condition assessment to inform conservation strategies for a small isolated Asian elephant population in southwest China |
| **Short Title:** | Population structure and body condition of Asian elephant in southwest China |
| **Corresponding Author:** | Kun Shi<br>Beijing Forestry University<br>Beijing, CHINA |
| **Keywords:** | Asian Elephant; population size; population density; population structure; camera traps; body condition scoring |
| **Abstract:** | The Asian elephant ( Elephas maximus ) population in Nangunhe National Nature Reserve represents a unique evolutionary branch in China, which has been isolated for more than twenty years from other neighboring populations in Myanmar. The scarcity of information on population structure, sex ratio and body condition make it difficult to develop effective conservation measures for this elephant population. Twelve individuals were identified from 3,860 valid elephant photographs at 52 camera sites that were obtained from February to June 2018 (5,942 sampling effort days). Three adult females, three adult males, one subadult male, two juvenile females, two juvenile males and one male calf were identified. The ratio of females to males was 1:1.4, and ratio of reproductive ability was 1:1.5, indicating the scarcity of reproductive females as an important limiting factor to population growth. Population density of 5/100 km 2 that was estimated by using Spatial Explicit Capture Recapture models was comparatively lower than the crude density estimate of 16.9/100 km 2 . A body condition scoring method that relies on six physical characteristics and ranks the elephant´s body condition according to a 11-points scale was used. Overall, the elephant population was found to be in normal health, with a median BCS of 5.75 (n=12, range 2-9). However, as an isolated population with an extremely small size as well as an inverted pyramid age structure, the population is at high risk of extinction, and therefore should be of top conservation priority in China. The database established in this study can provide researchers with valuable information to set up efficient conservation schemes. |
| **Order of Authors:** | Kun Shi |
| | Yakuan Sun |
| | Ying Chen |
| | Juan José Díaz-Sacco |
| **Additional Information:** | |
| **Question** | **Response** |
| **Financial Disclosure**<br><br>Enter a financial disclosure statement that describes the sources of funding for the work included in this submission. Review the submission guidelines for detailed requirements. View published research articles from *PLOS ONE* for specific examples.<br><br>This statement is required for submission | Funding for this work was provided by National Forest and Grassland Administration, http://http://www.forestry.gov.cn, China under the Project of Asian Elephant Habitat Assessment and Habitat Maintenance Pilot, with effective support by the Yunnan Forest and Grassland Administration. The funders had no role in study design, data collection and analysis, decision to publish, or preparation of the manuscript. |

Powered by Editorial Manager® and ProduXion Manager® from Aries Systems Corporation

| | |
|---|---|
| and **will appear in the published article** if the submission is accepted. Please make sure it is accurate.<br><br>**Unfunded studies**<br>Enter: *The author(s) received no specific funding for this work.*<br><br>**Funded studies**<br>Enter a statement with the following details:<br>• Initials of the authors who received each award<br>• Grant numbers awarded to each author<br>• The full name of each funder<br>• URL of each funder website<br>• Did the sponsors or funders play any role in the study design, data collection and analysis, decision to publish, or preparation of the manuscript?<br>• **NO** - Include this sentence at the end of your statement: *The funders had no role in study design, data collection and analysis, decision to publish, or preparation of the manuscript.*<br>• **YES** - Specify the role(s) played.<br><br>* typeset | |
| **Competing Interests**<br><br>Use the instructions below to enter a competing interest statement for this submission. On behalf of all authors, disclose any competing interests that could be perceived to bias this work—acknowledging all financial support and any other relevant financial or non-financial competing interests.<br><br>This statement **will appear in the published article** if the submission is accepted. Please make sure it is accurate. View published research articles from *PLOS ONE* for specific examples. | The authors have declared that no competing interests exist. |

| | |
|---|---|
| **NO authors have competing interests**<br><br>Enter: *The authors have declared that no competing interests exist.*<br><br>**Authors with competing interests**<br><br>Enter competing interest details beginning with this statement:<br><br>*I have read the journal's policy and the authors of this manuscript have the following competing interests: [insert competing interests here]*<br><br>* typeset | |
| **Ethics Statement**<br><br>Enter an ethics statement for this submission. This statement is required if the study involved:<br><br>• Human participants<br>• Human specimens or tissue<br>• Vertebrate animals or cephalopods<br>• Vertebrate embryos or tissues<br>• Field research<br><br>Write "N/A" if the submission does not require an ethics statement.<br><br>General guidance is provided below. Consult the submission guidelines for detailed instructions. **Make sure that all information entered here is included in the Methods section of the manuscript.** | Both National Forestry and Grassland Administration of China and Yunnan Forestry and Grassland Administration reviewed all sampling procedures and approved permits for the work conducted in NNNR. Non-invasive methods were applied and approval from an Institutional Animal Care and Use Committee or equivalent animal ethics committee was therefore not required. |

| | |
|---|---|
| **Format for specific study types**<br><br>**Human Subject Research (involving human participants and/or tissue)**<br>• Give the name of the institutional review board or ethics committee that approved the study<br>• Include the approval number and/or a statement indicating approval of this research<br>• Indicate the form of consent obtained (written/oral) or the reason that consent was not obtained (e.g. the data were analyzed anonymously)<br><br>**Animal Research (involving vertebrate animals, embryos or tissues)**<br>• Provide the name of the Institutional Animal Care and Use Committee (IACUC) or other relevant ethics board that reviewed the study protocol, and indicate whether they approved this research or granted a formal waiver of ethical approval<br>• Include an approval number if one was obtained<br>• If the study involved *non-human primates*, add *additional details* about animal welfare and steps taken to ameliorate suffering<br>• If anesthesia, euthanasia, or any kind of animal sacrifice is part of the study, include briefly which substances and/or methods were applied<br><br>**Field Research**<br><br>Include the following details if this study involves the collection of plant, animal, or other materials from a natural setting:<br>• Field permit number<br>• Name of the institution or relevant body that granted permission | |
| **Data Availability**<br><br>Authors are required to make all data underlying the findings described fully available, without restriction, and from the time of publication. PLOS allows rare exceptions to address legal and ethical concerns. See the PLOS Data Policy and FAQ for detailed information. | Yes - all data are fully available without restriction |

| | |
|---|---|
| A Data Availability Statement describing where the data can be found is required at submission. Your answers to this question constitute the Data Availability Statement and **will be published in the article**, if accepted.<br><br>**Important:** Stating 'data available on request from the author' is not sufficient. If your data are only available upon request, select 'No' for the first question and explain your exceptional situation in the text box.<br><br>Do the authors confirm that all data underlying the findings described in their manuscript are fully available without restriction? | |
| **Describe where the data may be found in full sentences. If you are copying our sample text, replace any instances of XXX with the appropriate details.**<br><br>• If the data are **held or will be held in a public repository**, include URLs, accession numbers or DOIs. If this information will only be available after acceptance, indicate this by ticking the box below. For example: *All XXX files are available from the XXX database (accession number(s) XXX, XXX.).*<br>• If the data are all contained **within the manuscript and/or Supporting Information files**, enter the following: *All relevant data are within the manuscript and its Supporting Information files.*<br>• If neither of these applies but you are able to provide **details of access elsewhere**, with or without limitations, please do so. For example:<br><br>*Data cannot be shared publicly because of [XXX]. Data are available from the XXX Institutional Data Access / Ethics Committee (contact via XXX) for researchers who meet the criteria for access to confidential data.*<br><br>*The data underlying the results presented in the study are available from (include the name of the third party* | Data cannot be shared publicly because of the restriction on confidentiality from the funding program. Data are available from the School of Nature and Conservation in Beijing Forestry University and Eco-Bridge Continental in Beijing for researchers who meet the criteria for access to confidential data. |

| | |
|---|---|
| *and contact information or URL).*<br>• This text is appropriate if the data are owned by a third party and authors do not have permission to share the data.<br><br>* typeset | |
| Additional data availability information: | |

Manuscript Click here to access/download;Manuscript;Manuscript.docx

# Population structure and body condition assessment to inform conservation strategies for a small isolated Asian elephant population in southwest China

Yakuan Sun[1¶], Ying Chen[2¶], Juan José Díaz-Sacco[1¶], Kun Shi [1,3¶*]

[1]School of Ecology and Nature Conservation, Beijing Forestry University, Beijing 100083, China

[2]School of Biological Science, The University of Hong Kong, Hong Kong, China

[3]Eco-Bridge Continental, Beijing 100085, China

[*]Corresponding author

E-mail: kunshi@bjfu.edu.cn (KS)

¶These authors contributed equally to this work.

# Abstract

The Asian elephant (*Elephas maximus*) population in Nangunhe National Nature Reserve represents a unique evolutionary branch in China, which has been isolated for more than twenty years from other neighboring populations in Myanmar. The scarcity of information on population structure, sex ratio and body condition make it difficult to develop effective conservation measures for this elephant population. Twelve individuals were identified from 3,860 valid elephant photographs at 52 camera sites that were obtained from February to June 2018 (5,942 sampling effort days). Three adult females, three adult males, one subadult male, two juvenile females, two juvenile males and one male calf were identified. The ratio of females to males was 1:1.4, and ratio of reproductive ability was 1:1.5, indicating the scarcity of reproductive females as an important limiting factor to population growth. Population density of 5/100 km$^2$ that was estimated by using Spatial Explicit Capture Recapture models was comparatively lower than the crude density estimate of 16.9/100 km$^2$. A body condition scoring method that relies on six physical characteristics and ranks the elephant´s body condition according to a 11-points scale was used. Overall, the elephant population was found to be in normal health, with a median BCS of 5.75 (n=12, range 2-9). However, as an isolated population with an extremely small size as well as an inverted pyramid age structure, the population is at high risk of extinction, and therefore should be of top conservation priority in China. The database established in this study can provide researchers with valuable information to set up efficient conservation schemes.


# Introduction

The Asian elephant (*Elephas maximus*), as the largest terrestrial mammal in Asia, is considered ~~to be~~ an ecosystem engineer because it can modify its habitat and aid in seed dispersal, ~~as~~ well as an umbrella species since its conservation can contribute to the preservation of the biodiversity ~~found in vast ecosystems~~, especially in tropical moist and in tropical dry broad-leaf forests of Southeast and South Asia [1-5]. It is also considered ~~to be~~ a flagship species that can attract interest for the protection of biodiversity ~~hotspot areas~~ [6]. It is a Class I Key Protected Species in China and is also listed as an Endangered Species under the IUCN Red List [7], showing decreasing population trends [8]. China sustains a population of c. 300 wild Asian elephants which are divided into seven geographic populations [9, 10]. Most Asian elephant populations are located in Mengyang, Mengla and Shangyong areas of Xishuangbanna National Nature Reserve, while there are also a few populations in Simao, Jiangcheng and Lancang counties of Pu'er city and ~~only~~ one small isolated population in the Nangunhe National Nature Reserve (NNNR) of Lincang city [9, 11].

The conventional methods used for tracking Asian elephants in the dense forest are difficult to accomplish and usually fail to ~~obtain~~ accurate population numbers and age structure [12, 13]. Although it is relatively easy to detect elephant signs, such as dung or footprints, by using sign surveys [14], it can be difficult to identify individual Asian elephants since ~~using only~~ signs ~~is considered to be~~ highly inaccurate and unreliable [15]. ~~By using DNA methods,~~ individuals can be identified, but it requires relatively fresh samples and the lab costs can be quite high; moreover, the population size can only be determined with genetic capture-mark-recapture methods, which can

be time and labor intensive [16, 17]. Furthermore, since calf elephants have lower defecation rates, these methods can likely underestimate the number of young individuals in a population [18].

Infrared trigger camera technology can make up for the above disadvantages [19, 20]. It has many advantages such as causing minimal disturbance to the study animal and its habitat, it can be used for long working hours and endure harsh weather conditions, and is relatively ~~of~~ low cost [19, 21, 22]. It is widely used in ecological baseline surveys of protected species [23, 24], to monitor endangered wildlife [25], to study habitat utilization [26], animal behavior [27] as well as the mechanisms of coexistence in sympatric species [28, 29]. Moreover, ~~the~~ infrared camera can also be used to estimate population size and density by using Spatial Explicit Capture-Recapture models [30-33]. This technology has been extensively ~~used~~ to estimate the population density of felines, especially the species that possess natural spots or stripes, such as the snow leopard (*Panthera uncia*), cougar (*Puma concolor*), Sumatran tiger (*Panthera tigris* ssp. Sumatrae), and serval (*Leptailurus serval*) [33-36]. For species without natural markings, identification can also be conducted according to morphology, such as with the Asian elephant, giant panda (*Ailuropoda melanoleuca*), and fox squirrel（*Sciurus niger*) [37-39].

Population structure and density estimation are essential features that can be used to study the ecology of wildlife populations [40, 41]. Throughout the Asian elephant range, there have been a number of studies that have identified individual elephants by directly photographing them or by obtaining images from infrared cameras [17, 37, 42,

43]. In China, these studies have mainly been concentrated in Xishuangbanna National Nature Reserve [17, 42], focusing on population size estimation from individual identification, but not on the density or health assessment of its populations. S[illegible]lies conducted on the elephant population in NNNR concluded that it was a separate evolutionary unit [44] isolated from other geographic populations [13], thus lacking genetic connectivity. Although the closest elephant populations can be found in Myanmar, the forest degradation in the China-Myanmar border area has led to the isolation of this elephant population since 1997 [9, 45, 46]. Additionally, this population is facing threats from habitat loss, fragmentation and illegal hunting [45, 47, 48]. Although conservation interventions have been proposed to rescue this population from extinction, the lack of scientific data and information on population structure, relatedness, activity pattern, and health status prevent the development of efficient conservation strategies.

[illegible]dy condition is an index of an animal's health, since it can reflect the quality of the environment in which it lives [49]. Body condition scoring (BCS) of Asian elephants based on photographs is considered to be as accurate as ultrasonic subcutaneous fat measurement, thus making it easier to evaluate the health condition of wild populations since individuals do not need to be captured [50, 51]. BCS is individual-based, but it is most meaningful when applied to a population, espe[illegible]ly small isolated populations [50]. It can be used for both longitudinal and cross-sectional studies, is comparatively inexpensive and does not require a high technical capacity [50]. However, there have been no studies focusing on assessing the health of any of

~~the Asian elephant populations in China.~~

Isolated populations are vulnerable to changes in the external environment and thus, their conservation should be a top priority [52, 53]. However, previous ~~research~~ were ~~only~~ limited to studying ~~the~~ population size and did not ~~analyze~~ the health condition of the population [13, 48]. Accurate understanding of the current situation of the ~~local~~ elephant population in NNNR, especially its ~~sex ratio~~ and breeding ~~information,~~ is key to establish a baseline for the development of a successful project focusing on the introduction of individual ~~elephants~~ from other populations and develop more effective protection and management schemes.

~~Therefore, it is essential to study the population structure and health condition of the Asian elephant in NNNR.~~ In this study, our aim was to ~~use camera trapping to~~ determine the ~~number,~~ density, demographic structure and health~~y~~ condition of this Asian elephant population, and to analyze ~~th~~[illegible]ctors that have affected its population size in recent years in order to develop targeted and effective protection strategies. ~~Furthermore,~~ our approach~~es~~ can be applied to other elephant populations in China with further implications for global Asian elephant conservation.

# Material and Methods

## Ethics Statement

Both National Forestry and Grassland Administration of China and Yunnan Forestry and Grassland Administration reviewed all sampling procedures and approved permits for the work conducted in NNNR. Non-invasive methods were applied and approval from an Institutional Animal Care and Use Committee or equivalent animal

ethics committee was therefore not required.

## Study area

The study area is located in the western part of the Nangunhe National Nature Reserve, Yunnan, where elephants are found [45, 47] (Fig 1). It occupies 70.82 km$^2$ at latitude 23° 13′ - 23° 19′, longitude 98° 54′ - 99° 05′, and the altitude ranges from 510 m to 1747 m. Rainfall is highly seasonal, with a dry season between November to April and a rainy season between May to October. Annual precipitation and temperature are 40.1 mm –1371.8 mm and 15.8–24.5 ℃, respectively [54]. Vegetation and climate vary across elevation zones. Natural tropical forest and tropical monsoon forest are located at altitudes below 600 m. Middle elevation forests are dominated by subtropical monsoon evergreen broad-leaf forests. Evergreen vegetation is found above 1700 m [47]. Bamboo forest, shrub and grasslands are scattered throughout this area. Deforestation along the China and Myanmar border has disrupted the connectivity between elephant populations that are found in both of these countries [45]. The iron fence in the southern part of reserve is used to protect the surrounding villages from wildlife (Fig 1).

**Fig 1. Map of study area in NNNR showing all the camera sites with elephants (presence points) or without elephants (absence points)**.

## Camera traps

We carried out the survey for four months between February and May 2018 using automatically triggered camera traps. We systematically deployed camera traps along

the animal trails with forest gaps where there were signs of elephants， including footprints, dung, foraging sites as well as other activity sites. However, we avoided the northwestern part of the reserve where there were steep slopes at higher altitudes of up to 1747 m. Cameras were strapped on trees at approximately 1.5 m above the ground with the sensor parallel to the ground to maximize the extension of the detection zone. The positions of all the camera sites were recorded by GPS. To avoid blank images caused by sunlight, the cameras were placed away from the east direction. Camera traps were deployed as hidden as possible to reduce the disturbance to wildlife and the probability of been stolen. Camera traps were set to operate 24 hours per day and programmed to take sequential photographs without delay registering date and time for each exposure. At each site, camera traps were operated continuously for a maximum of four months. SD cards and batteries were replaced every month. We recorded the time of installation and retrieval of each camera and calculated the total duration of sampling.

## Individual identification

The photographs obtained from the camera traps were categorized to independent events, which were defined as sequences, meaning from the first individual entering the field to the last one exiting it in the same camera, with at least up to 5 hours between two connected sequences [55]. Only photographs with sufficient quality, clarity and position of the elephant in the frame were used for individual identification [55]. Here, quality referred to how usable a picture was for age and sex classification; clarity indicated whether the elephant was “captured” in good light, the flash reached the

individual, and the picture was in focus and not over or under-exposed; and position referred to the occurrence of the elephant within an 'optimal distance and angle' from the camera [43, 55]. The age was broadly classified into adult, subadult, juvenile and calf using approximate shoulder height as recommended by previous studies [56, 57]. Gender of an elephant was mainly determined by its secondary characteristics. Females were recognized by their apparent breasts and lack of tusks. Males were identified by their distinctive tusks, lack of breasts and whenever possible, by the presence of a penis. We defined the age structure as the ratio of adults, subadults, juveniles and calves while the reproductive ability was the ratio of age structure between adult and juvenile females [43], due to the absence of subadult and calf females. Male elephants living completely or mostly outside the herd were classified as solitary males. Based on individual events, a capture history event database was established to record the individuals and their characteristics. Morphological characters including facial bones, ear, tusk, back and tail hair that did not change in a short period of time were clearly described, confirming the reliability of identification results [17, 37, 42, 58] (Table 1). According to the shooting time of Asian elephant photos, their daily activity patterns were also summarized.

**Table 1. Morphological characters used for individual identification of elephants photographed in NNNR, between February and May 2018**

| Characters | Categories | Description |
|---|---|---|
| Gender | Female | Without obvious tusks but with breasts |
| | Male | With obvious tusks or penis but without breasts |
| Age | Adult (>15years) | With the highest shoulder height |
| | Subadult (5-15years) | Shoulder height reaches the adult's eyes |

| | | |
|---|---|---|
| | Juvenile (1-5years) | Shoulder height reaches half the height of an adult |
| | Calf (0-1year) | Below the adult's knee |
| Facial bones | Wisdom tumor | Size |
| | Bones between two eyes | Extent of prominence |
| | Upper margin of eyes | Sunken[illegible]minence |
| Tusks | Length | Very short/short/long |
| | Thickness | Thick/normal/slender |
| | Shape | Straight/bend |
| | Crack | Yes/no |
| Ear | Upper edge | Fold/unfold |
| | Lobe | V-acute/U-rounded |
| | Lobe tear | Yes/no |
| | Ear hole | Yes/no |
| Tail brush | Standard | Present/both sides/both-continuous |
| | Nonstandard | Absent/single side/both-discontinuous |

## Density estimation

The elephants in NNNR were identified as a small isolated population without any migrants, thus conforming to the "close population" assumption of Spatial Explicit Capture-Recapture models (SECR) [59]. The model accounts for the movement in density estimation by combining a state model and an observation model [59]. The state model describes the movement pattern of individuals activity ce[illegible]r in study area, while the observation model (spatial detection model) describes the probability of detecting an individual at a particular detector (e.g. camera trap in this study) to the distance of the detector from a central point in each individual's activity center. Basically, SECR are generalized linear mixed models that assume that every individual *i* in the population has its own unobserved activity center $S_i$, and all the activity centers $S_i \cdots S_N$ distributed across the study area [60, 61].

The encounter rate of an individual *i* with a given detector *j* located at $X_j$ is a monotonically decreasing function of the distance from $S_i$ to *j*. $Y_{ij}$, the number of times

individual $i$ is detected by trap $j$ during a sampling occasion, is a random variable following a Poisson distribution and with mean $\lambda_{ij}$ [62]:

$$Y_{ij} \sim \text{Poisson}(\lambda_{ij})$$

For an elephant $i$ captured by a trap $j$, the model assumes a log-linear form [63]:

$$\text{Log}(\lambda_{ij})=\text{Log}(\lambda_0)-\left(\frac{1}{2\sigma^2}\right)\|S_i\text{-}X_j\|$$

Here, $\lambda_0$ is the baseline encounter probability ~~and~~ the function $\|S_i - X_j\|$ is the distance between the activity center and trapping station, and $\sigma$ is the Gaussian scale parameter for the distance function between activity centers and detector locations, assuming a bivariate normal model of space use, sigma can be used to calculate a 95% ~~home range~~ radius.

Density is a derived parameter calculated by dividing N by the area of the state space S, which N is the number of activity center in S, includes the trapping grids. SECR models estimate the density of animal activity centers in an area large enough that animals residing beyond it have a negligible chance of being detected [59]. We used a 5 km buffer from the outermost x and y coordinates of the camera sits which could cover the study area, corresponding to an area S of 255.8 $km^2$.

SECR 3.1.5 package in R ~~Language~~ was used for density estimation. ~~Therefore,~~ data ~~for analysis in SECR was~~ prepared as an R object of class "capthist". This object included both the capture data and the detector layout. The file named capture histories containing data on individual identification, the occasion (day), the detector (camera ID). The detector file containing data on cameras ID, and x, y coordinates. We used

monthly and whole data as five different sessions to run the model to see the estimation accuracy under different detection efforts.

# Body condition scoring

~~We established a database of each identified individual and~~ the photographs with the best angle of each individual elephant were selected to score the body condition. Compared with previous studies, our target population size was small, and ~~the~~ sample size was limited so we used a simple method which can rapidly and ~~more~~ easily be applied to wild elephant populations. In this study, we applied a BCS method that focuses on six physical characteristics and ranks the ~~elephant´s~~ body condition according to a 11-points scale [49, 50]. This method is based on a scale of 5 reference photographs of target scored elephants that focused on the ribs, shoulders, pelvic girdles, backbone and neck rolls [50]. The scale photographs were assigned scores of 1, 3, 5, 7, 9, with numerically higher values corresponding to better body condition (Table 2). But the range of scores extended from 0 to 10 due to the possibility of greater variation. If all the ribs of an elephant were visible, it meant that this individual presented a worse body condition than 1 and thus, it would be assigned 0. If an individual presented a fatter condition than 9 and had very thick rolls of skin fold below neck, it would be assigned a score of 10. All statistical analyses were carried out using SPSS. Correlation coefficient (r) was used to study the relationship between BCS and ratios of various covariates such as sex, age, and whether an individual was present or not in the herd.

**Table 2. Diagnostic characters pertaining to scores in photographic scale.**

| Score | Characters |
|---|---|
| 1 | Ribs from shoulder to pelvis are visible, some ribs prominent (spaces in between sunken in) |
| 3 | Some ribs visible (spaces in between not sunken in), shoulder and pelvic girdles prominent |
| 5 | Ribs not visible, shoulder and pelvic girdles visible |
| 7 | Backbone visible as a ridge, shoulder and pelvic girdles not visible |
| 9 | Backbone not visible or difficult to differentiate and pelvic bones not visible |

# Results

## Individual identification

We carried out camera trap surveys at 52 locations (Fig 1) for 5,942 trap days (114.27 ± 19.83 (SD); range: 73-129). All the camera traps were systematically deployed in broad-leaf forest, broad-leaf and bamboo forests, bamboo forest, pine forest and nearby the brush forest, which represented the dominant habitat types in NNNR. A total of 70,902 photos and videos were obtained, among which 7.34% (5,207) were Asian elephant data. There was a total of 134 independent events, including 40 herd independent events and 94 individual independent events. Excluding the image data that could not be used for individual identification, there were 3,860 valid Asian elephant photos and videos belonging to 85 independent events. A total of 12 individuals were identified among valid independent events (Fig 2~~). A simple database information is shown in S1 Table~~. The minimum population number of Asian elephants in each month was consistently 12. Photographic frequencies were different among individuals and capture histories: elephant number 12 (AE12) ~~accounted for~~ the least frequent (16) ~~of the records~~ whereas all other individuals were captured at least 20 times (Table 3).

**Fig 2. Several different characteristics used for individual identification of**

**elephants.** All the pictures come from different capture events. A & B were juvenile males and treated as two individuals according to A's short straight tusks and B's long curve tusks; C & D were treated as the same individual by the V-acute of ear lobe; E was distinguished by its distinctive protuberance on the center of forehead; Both F & G had similar tail hair but were ~~identified~~ by others features.

**Table 3. Number of capture events for a given individual per month.**

| Individual No. | Age | Gender | Tusk | Type | Capture events | | | | |
|---|---|---|---|---|---|---|---|---|---|
| | | | | | Feb. | Mar. | Apr. | May | Total |
| AE01 | Juvenile | Male | Yes | Her[illegible] | 2 | 6 | 5 | 8 | 21 |
| AE02 | Adult | Female | | | 3 | 5 | 6 | 6 | 20 |
| AE03 | Adult | Female | | | 3 | 5 | 6 | 7 | 21 |
| AE04 | Juvenile | Female | | | 2 | 6 | 5 | 7 | 20 |
| AE05 | Calf | Male | No | | 3 | 6 | 8 | 5 | 22 |
| AE06 | Adult | Female | | | 4 | 6 | 8 | 5 | 23 |
| AE07 | Juvenile | Female | | | 4 | 6 | 9 | 6 | 25 |
| AE08 | Juvenile | Male | Yes | | 4 | 7 | 6 | 7 | 24 |
| AE09 | Sub-adult | Male | No | Solitary | 3 | 9 | 2 | 9 | 23 |
| AE10 | Adult | Male | Yes | Solitary | 2 | 3 | 13 | 7 | 25 |
| AE11 | Adult | Male | No | Solitary | 3 | 5 | 8 | 5 | 21 |
| AE12 | Adult | Male | No | Solitary | 2 | 6 | 1 | 7 | 16 |
| Total | - | - | - | - | 35 | 70 | 77 | 79 | 261 |

Different types of behaviors, such as foraging (including ~~milk feeding of~~ calves), drinking water, sand bathing, sleeping, communication and the use of branches were recorded. A total of six scenes of Asian elephants lying down and resting were recorded on video, including that of one calf, four juveniles and one adult female. The longest sleeping time recorded was 1 h 36 min for adult females. The herd of elephants clearly showed a higher daily activity frequency at 6:00-8:00 and 14:00-16:00, while the activity time of the solitary elephants lagged behind at 8:00-10:00 and 18:00-20:00 respectively.

## Demographic structure ~~and relatedness~~

We identified an inverted pyramid age structure: three adult females, three adult males, one subadult male, two juvenile females, two juvenile males and one male calf. For the sex, the male to female ratio was 1.4:1, for adult as well as juvenile individuals, the ratio was 1:1. The ratio of reproductive ability was 1:1.5. Judging from camera trap data, there were two containment relationship family units. AE01-AE08 formed the big family unit, while individuals AE05, AE06 and AE07 formed the small unit which occasionally departed from the big unit. Adult female AE06 and male calf AE05 were definitely in a mother-child relationship since there were photographs of the calf sucking milk from the female, and juvenile AE07 might have a kindred relationship with AE05. Adult female AE03 and juvenile female AE04 were most likely in a mother-child relationship since lots of image data showed AE04 actively moving close to AE03.

## Population density

The estimated density was 5.32±1.56 individuals per 100 $km^2$ (with a 95% confidence interval of 3.02-9.36) and the population size was 12.00±0.71 (with a 95% confidence interval of 12.00-12.11) (Table 4). April had the lowest density estimation at 6.07±1.79 individuals per 100 $km^2$, while in May the density reached 24.43±7.20 individuals per 100 $km^2$. The movement parameter σ was twice as large for April (6643m±2356m) than for May (3771m±1471m). Each session had a similar population size, excluding the standard error estimate in February which was higher than in the remaining sessions. The 95% confidence interval reached 12.35-23.43.

**Table 4. Population density estimation by using SECR models under five sessions.**

| Sessions | Occasions | Density | | | $\lambda_0$ | | sigma | | Population size | | |
|---|---|---|---|---|---|---|---|---|---|---|---|
| | | estimate | SE.estimate | 95% CI | estimate | SE.estimate | estimate | SE.estimate | estimate | SE.estimate | 95% CI |
| February | 11 | 10.71 | 3.31 | 5.91-19.38 | 0.15 | 0.04 | 2983 | 664 | 14.00 | 2.19 | 12.35-23.43 |
| March | 26 | 6.57 | 1.94 | 3.73-11.58 | 0.07 | 0.02 | 4193 | 700 | 12.20 | 0.87 | 12.00-17.95 |
| April | 22 | 6.07 | 1.79 | 3.45-10.70 | 0.02 | 0.004 | 6643 | 2356 | 12.05 | 0.75 | 12.00-16.87 |
| May | 26 | 24.43 | 7.20 | 13.87-43.03 | 0.01 | 0.003 | 3711 | 1471 | 12.03 | 0.74 | 12.00-16.48 |
| Total | 85 | 5.32 | 1.56 | 3.02-9.36 | 0.02 | 0.003 | 6474 | 1065 | 12.00 | 0.71 | 12.00-12.11 |

# Body condition scoring

The distribution of BCS for the population had a median ~~BCS~~ of 5.75 (n=12, range 2-9) (Fig 3). ~~Collectively,~~ only one individual had a minimum BCS of 2 and two had a maximum BCS of 9, respectively. Half of the individuals reached above the median scores. ~~The frequency of BCSs by sex is shown in Fig.3.~~ BCS ranged 2-7 in females and 4-9 in males [illegible]e reproductive adult female AE02 had a BCS just above the median score while the other reproductive female AE03 had a lower BCS of 3. The calf'~~s~~ BCS was 8, juveniles~~'~~ BCS ranged from 4-7, subadult was 4, adults~~'~~ BCS ranged from 2-9. Age ~~group~~ was not significantly correlated to ~~the~~ BCS (r= 0.03). Solitary or not ~~and sex~~ played more important roles in the BCS (r = 0.53 ~~and 0.42 respectively~~).

**Fig 3. Body condition score of 12 individuals in NNNR.**

# Discussion

# Asian elephant population number in NNNR

I~~ndividual i~~dentification from captured photos and videos was robust to identify individuals [64]. Unlike dungs and footprints that are easily influenced by slope, soil ~~quality~~ and c~~limatic~~ factors [65], this study ~~installed~~ cameras in the elephant~~'s~~ preferred habitat~~s~~ that covered most of the western part of the reserve, which provided a higher chance of obtaining ~~the~~ data reflecting the real population. Overall, the population size identified in this study was consistent with the survey results ~~of 40 years ago~~ [66] that determined ~~that it presented~~ a small population size ~~status~~ fluctuating around 20 individuals [48]. Compared with other Asian elephant populations in China as well as in other Asian countries, the NNNR population is definitely an extremely small population [1, 9, 10, 12]. Unlike other small populations in China, such as the Xishuangbanna Menghai-Pu'er Lancang population with 19 individuals distributed outside the reserve that can move freely around farmlands and natural forest [67], the NNNR population is completely locked into this small area. Illegal hunting could have had a direct impact on this population with a total of 16 unnatural deaths since 1987 (Table 5). ~~Moreover, inconsistent survey methods that have been adopted throughout the years might explain slight differences in population size results (see more details in S2 Table).~~ All these results indicate that the small population ~~size~~ in NNNR is in urgent need of conservation interventions.

**Table 5. Unnatural deaths of Asian elephant since the establishment of the NNNR.**

| Year | Number | ~~Reason~~ | Data resources |
|---|---|---|---|

| | | | |
|---|---|---|---|
| 1987 | One female and one male | Illegal killing | [54, 68] |
| 1988 | Two adult males, one adult female and one calf | Poaching, electrocuting and unknown reason | [45] |
| 1995 | One male | Unknown | [48] |
| 1996 | One male | Vengefully hunted by local people | [47] |
| 1997 | One female, one male and a calf | Unknown for the calf, the female and male were killed | [54] |
| 2003 | ~~A~~ Subadult male and one adult male | Unknown for the subadult, the adult male died for its ivory | [47, 54] |
| 2008 | ~~A~~ Subadult male | Unknown | Communication with local residents and rangers |
| 2011 | One juvenile | Unknown | [54] |
| 2016 | One Adult male | ~~The cause of death was~~ unknown, but the tusks show visible saw marks | Communication with local residents and rangers |

# Population structure in NNNR

Our survey~~s~~ showed differences in age structure and sex composition of the NNNR population compared with another recent study [48]. We identified one more adult male elephant than in this study, a more detailed age ranking structure and sex composition among young elephants, and even identified the male calf by observing the penis sheath from high quality images. There are more adult and subadult individuals in the NNNR than juveniles and calves, thus indicating a declining population trend in the future. Juveniles accounted for 33% of the population, although studies have shown that it is suitable for juveniles to account for more than 20% of the population for the population to be self-sustaining in a short period of time [18]. Small populations are likely to suffer from inbreeding depression [69]. Two mother-calf relationships were identified from camera trap photos, and the NNNR population was likely to be a big family unit, which may not be conducive to the long-term survival of

the population. Genetic studies are urgently needed to better understand the degree of inbreeding depression in the NNNR population.

Male elephants are commonly the first choice of poachers since illegal ivory-based poaching leads to a serious gender imbalance, with females outnumbering males [70]. For example, the ratio of female to male in Shangyong reserve in China is 10.5:1[17], and as high as 100:1 in Periyar Tiger reserve in India [71]. The NNNR population had an even sex ratio for adults which was consistent with a recent population survey by aerial drones [48]. Large number of females could have a long-term negative effect on the population as shown by the decrease in the number of effective elephants, the decrease in the birth rate, and the low genetic viability caused by inbreeding, while a balanced sex ratio can avoid negative effects driven by skewed sex ratio [12, 72].

The high proportion of tuskless adult males in NNNR could indicate that the juveniles that were identified as females might in fact be males. We judged these juveniles as females under the condition that they have similar body height as juvenile males who had apparent tusks. Males in some populations tend to be tuskless for survival due to the intensive historical poaching pressure [12, 73]. The number of unnatural deaths in the past 30 years was skewed towards males (Table 5) and at least 116 individual deaths in the past century might account for tuskless males [74, 75]. Since tuskless males are common in this population, it would be helpful to track the historical trend of the proportion of males with or without tusks in order to obtain scientific evidence-based explanations for the current sex ratio and proportion of tuskless males [74]. Moreover, genetic studies on elephant specimens and living

individuals could give us more insights on the sex ratio trend in the last few decades as well as ~~in~~ the proportion of tuskless males. If further molecular studies confirm that all current juveniles are males, this could lead to a relatively high skewed sex ratio. Currently, only AE02, AE03 and AE06 are in reproductive age, but the latter was nurturing a calf, leaving only two sexually active females in the reserve. Since females do not enter the breeding period until they are 10-14 years old ~~and afterwards they have a long birth interval (4-5 years between calves)~~, female juveniles still need 3-7 years to become mature [9, 12, 76]. In either case, the scarcity of fertile females would be the primary constraint on population growth.

## Population density

Accurate assessment of population size is essential for species conservation and management [77]. Before this study, only crude density estimation (estimated population number/study area) was available to compare the density of Asian elephants in different regions [18, 43]. The crude density estimate of Asian elephants in NNNR was the highest at 16.9/100 km$^2$ compar~~ing~~ with that in Shangyong, Mengla, Menyang with a density of 10.68, 4.26, 1.85 per 100 km$^2$ respectively [13, 17]. ~~Although~~ elephants are known to swim across the river, a short iron fence was built on the opposite side of the river in the southern boundary of ~~this part of~~ the reserve thus restricting their range (Fig 1)~~, none~~ signs were found in this area and no records of elephant invasion from the settlements. Therefore, the fence and the high elevations found on the northwestern side of the reserve ~~are obstacles~~ that reduce the ~~activity zones~~ for the NNNR population. High population density could lead to more intensive

competition among individuals for various natural resources [78].

At present, SECR models have become a powerful tool to estimate small population sizes like the one in NNNR [32], with the results being more robust than by using crude density estimation. Compared other population density in India by using SECR, the NNNR population density was quite low [79]. The model defines the activity center according to the range of the species activities [80], so it is easy to overestimate the population density in a short monitoring time period, which was confirmed in this study. May presented the highest density because there were 8 cameras that captured elephants, and the distance between each one was less than 800 m. Thus, after four months of monitoring, more accurate elephant movement data was obtained, and we got the smallest standard deviation of density value and number, which indicated that the results of the total data were closer to that of the real value [43]. A minimum number of 20 elephants were estimated in NNNR under the assumption of a population density of 0.1-0.3 elephant km$^2$, which is commonly seen in tropical forests across elephant range countries [81, 82]. Thus, our estimate of 12 elephants is likely to be below the area´s carrying capacity. Therefore, we assumed that there is enough space in NNNR to allow for this elephant population to reach its highest ever recorded population size of 24-25 individuals [74, 83].

## Health condition

Due to the small sample size, this study could not adopt more detailed assessment methods and only focused on dry season, but the results can still reflect the physical condition of NNNR population as well as the individual differences in range, foraging

and behavioral activities [84]. Sex was a significant factor affecting BCS. Females, however, usually have higher BCS than males as documented in other Asian and African elephant populations as well as in dogs and horses [85, 86]. However, a trend towards higher body fat in males compared to females in NNNR population was found. This situation was also observed in another elephant population in Southern India [87]. Solitary males are known to have larger home areas than family units, and adopt a high-risk high-gain foraging strategy by venturing into agricultural areas and feeding on nutritious crops [88]. The camera sites that only captured adult males also recorded highly intensive anthropogenic activities which included hunting, gathering, fishing and collecting. Besides, nutritional stress in lactating females (AE06) could have resulted in their poor body condition [87]. The BCS of juveniles ranged 4-7, which is slightly lower than median scores were reasonable. Difference in patterns of fat deposition may exist between sexes, age-groups and sub-species. Previous studies reported that depressions and bony appearance were more visible in adult elephants than in subadults and juveniles [84, 87]. The quantity and quality of food and availability of water are the primary factors determining body condition of elephants in the wild [49], while there is also less availability of nutritional food during the dry season. Since non-waterproof cameras failed work in the rainy season, we did not have the opportunity to conduct camera trap surveys during this period of time. However, in recent years, based on the data recorded by the bureau to compensate villagers for damages caused by elephants in NNNR, the elephants have been recorded foraging for corn and rice during the rainy season from the month of July to October. Establishing

the food base in an accessible area for the family unit during the whole year could improve their health condition in dry season. Therefore, the body condition of the elephant is not only a reflection of its health but also an indicator of other factors that affect body condition, such as energy reserve, fitness for reproduction and breeding behaviour that can help managers elaborate future conservation schemes [51, 87].

Moreover, the body condition assessment in this study can have implications for the research and conservation of other elephant populations in China. Since the vast majority of Asian elephant populations are found outside reserves, these elephants can have access to plenty of food crop resources, thus causing serious conflicts with local inhabitants [89]. Therefore, analyzing the body condition of different regional Asian elephant populations can be used as an early indicator of the impact of management actions [50]. The data can directly reflect the obesity level of individuals among the population, and indirectly reflect the status of food resources in areas of distribution, which can guide conservationists and policymakers to restore suitable habitats as well as improve crop management schemes [49].

## Conservation recommendations

By using camera trap photos, NNNR was found to have a small elephant population with an uneven age structure as well as few females in reproductive period with relatively poor body conditions. Since the long-term placement of cameras in the wild can provide valuable data for the study of demography, reproductive and health status of Asian elephants [43, 55, 56, 90], systematic camera surveys and continuous long-term monitoring of NNNR population could aid in eliminating errors in the

population size and density estimated in this study, by incorporating data on survival and reproductive rates as well as movement patterns. Small population size, inbreeding and restricted gene flow all contribute to the reduction in the genetic diversity [91], which has been shown to affect population persistence and individual fitness as well as adaptation, thus likely increasing the risk of extinction [92]. Population viability analyses for Asian elephant show that such a small population may have a 70% probability of extinction from chance alone if the deterministic population growth rate is zero [81, 93]. The presence of both breeding females and males, and the availability of areas with suitable habitat and resources, however, offer some hope for partial recovery. External conditions like strict protection measures, the promotion of the "Elephant Worshipping Festival" and successful strategies to mitigate human-elephant conflicts can all have a positive effect on its population recovery [47, 94]. Molecular techniques should be conducted in the near future to obtain information related to kinship, reproductive matters as well as physical health. The results obtained in this study have reconfirmed that the reintroduction of individual Asian elephants should be put on top of the conservation agenda of China and that the enlargement of suitable habitat is urgently needed to ensure the richness of food resources for this isolated population [13, 45, 47, 48, 95]. Preferred elephant habitats that were confirmed from previous studies, such as mixed forests and thickets below 1000 m [45, 96], should be prioritized for habitat restoration. The main activity regions have now been partially blocked by a thick iron fence to separate settlements from elephants. This approach can partly relieve human-elephant conflict [97]. However, it has also completely isolated

this elephant population from other populations, and is considered to be a money-wasting and time-consuming approach. Fence dismantlement could provide more opportunities for elephants to explore new suitable ~~activity places and~~ areas with enough food resources. Due to the very small population size and the scarcity of reproductive females, the translocation of Asian elephants must be urgently accomplished before the juveniles reach reproductive age to avoid inbreeding [47]. We also suggest that research on the evaluation of the environmental capacity and availability of food resources should be conducted to set up a scientific baseline before a detailed plan on the reintroduction of new individuals can be made.

# Acknowledgments

This study was implemented by Beijing Forestry University and Beijing Eco-Bridge Continental. We thank for the support received from the Second National Survey of Terrestrial Wildlife in China, National Forestry and Grassland Administration of China. The field work was strongly supported by the administrators of Nangunhe National Nature Reserve. We also thank all the field staff, including Wang Jiahui, Liu Zhen, Liu Ying and all local rangers, especially Li Yongxiang, Wang Zhisheng, Li Chunlian and Li Zhimin, for their hard and invaluable work.

# References

1. Riddle HS, Schulte BA, Desai AA, Meer Lvd. Elephants - A conservation overview. Journal of Threatened Taxa. 2010;2(1):653-661.
2. Campos-Arceiz A, Blake S. Megagardeners of the forest – the role of elephants in

seed dispersal. Acta Oecologica. 2011;37(6):542-553. doi: 10.1016/j.actao.2011.01.014.

3. Aijun T, Lingjiang Y. Distribution and Protection of Asian elephant in China. Protection Forest Science and Technology. 2015;(5):89-91. doi: 10.13601/j.issn.1005—5215.2015.05.035.

4. McConkey KR, Nathalang A, Brockelman WY, Saralamba C, Santon J, Matmoon U, et al. Different megafauna vary in their seed dispersal effectiveness of the megafaunal fruit Platymitra macrocarpa (Annonaceae). Plos One. 2018;13(7):e0198960.

5. Platt SG, Bickford DP, Win MM, Rainwater TR. Water-filled Asian elephant tracks serve as breeding sites for anurans in Myanmar. Mammalia. 2019;83(3):287-289. doi: 10.1515/mammalia-2017-0174

6. Bowen-Jones E, Entwistle A. Identifying appropriate flagship species: the importance of culture and local contexts. Oryx. 2002;36(2):189-195. doi: 10.1017/S0030605302000261

7. Jiang Z, Jiang J, Wang Y, Zhang E, Zhang Y, Li L, et al. Red List of China's Vertebrates. Biodiversity Science. 2016;24(5):500-551. doi: 10.17520/biods.2016076.

8. Choudhury A, Lahiri Choudhury DK, Desai A, Duckworth JW, Easa PS, Johnsingh AJT, et al. Elephas maximus. The IUCN Red List of Threatened Species, 2008. IUCN SSC Asian Elephant Specialist Group (2008). 2008. doi: e.T7140A12828813.

9. Li Z. Research on Asian elephant Conservation in China: Science Press; 2018.

10. Yu Z, Kun J. Distribution, Population, Habitat Status and Population Management of Asian Elephant. World Forestry Research. 2018;31(2):25-30. doi: 10. 13348 / j. cnki. sjlyyj. 2018. 0002. y.

11. Mingyong C. Asian elephant research of China: Science Press; 2006.

12. Sukumar R. A brief review of the status, distribution and biology of wild Asian elephants Elephas maximus. International Zoo Yearbook. 2006;40(1):1-8.

13. Zhang L, Dong L, Lin L, Feng LM, Yan F, Wang LX, et al. Asian Elephants in China: Estimating Population Size and Evaluating Habitat Suitability. Plos One. 2015;10(5):e0124834. doi: 10.1371/journal.pone.0124834. PubMed PMID: WOS:000354918600011.

14. Tian Y, Feng Y, Zhang C, Yu B, Tang X, Hu H. Effectiveness of line transects during wild animal surveys in mountain forests of South China. Biodiversity Science. 2015;23(1):109-115. doi: 10.17520/biods.2014128.

15. Barnes RFW. The problem of precision and trend detection posed by small elephant populations in West Africa. Afr J Ecol. 2002;40:179-185. doi: 10.1046/j.1365-2028.2002.00376.x PubMed PMID: WOS:000325984300002.

16. Mills LS, Citta JJ, Lair KP, Schwartz MK, Tallmon DA. Estimating Animal Abundance Using Noninvasive DNA Sampling: Promise and Pitfalls. Ecol Appl. 2000;10(1):283-294.

17. Zicheng Y, Ying C, Junsong L, Lifan W, You P, Zhiyong S, et al. Individual identification and population size assessment for Asian elephant based on camera-

trapping techniques. Acta Theriologica Sinica. 2018;38(1):18-27. doi: 10.16829/j.slxb.150109

18. L.S.EGGERT, J.A.EGGERT, D.S.WOODRUFF. Estimating population sizes for elusive animals: the forest elephants of Kakum National Park, Ghana. Molecular Ecology. 2003;(2):1389-1402. doi: 10.1046/j.1365-294X.2003.01822.x.

19. Liu X, Wu P, He X, Zhao X. Application and data mining of infra-red camera in the monitoring of species. Biodiversity Science. 2018;26(08):70-81. doi: 10.17520/biods.2018053.

20. Xiao W, Hu L, Huang X, Xiao Z. Using capture-recapture models in wildlife camera-trapping monitoring and the study case. Biodiversity Science. 2019;27(3):257-265. doi: 10.17520/biods.2018194

21. Zhang L, Cui S, Huang Y, Chen D, Qiao H, Li C, et al. Infrared camera traps in wildlife research and monitoring in China: issues and insights. Biodiversity Science. 2014;22(6):696-703. doi: 10.3724/SP.J.1003.2014.14225.

22. Wearn OR, Glover-Kapfer P. Snap happy: camera traps are an effective sampling tool when compared with alternative methods. Roy Soc Open Sci. 2019;6(3):181748. doi: 10.1098/rsos.181748. PubMed PMID: WOS:000465470300048.

23. Jorge A A, Carlos E F S, Krisna G, Chris H, Johanna H, Emanuel M, et al. Community structure and diversity of tropical forest mammals: data from a global camera trap network. Philosophical Transactions of the Royal Society of London. 2011;366(1578):2703-2711. doi: 10.1098/rstb.2011.0115 PubMed Central

PMCID: PMC21844049.

24. Chengcheng Z, Jun W, ALEXANDER JS, Zhigang D, Liji W, Wantao D, et al. Biodiversity Assessment of Mammal and Bird Species from Camera Trap Data in Yanchiwan National Nature Reserve, Gansu Province, China. Journal of Resources and Ecology. 2018;9(5):114-122. doi: 10.5814/j.issn.1674-764x.2018.05.014.

25. Broto B, Mortelliti A. The status of research on the mammals of Sulawesi, Indonesia. Mammal Rev. 2019;49(1):78-93. doi: 10.1111/mam.12141. PubMed PMID: WOS:000454421000006.

26. Montalvo VH, Sáenz-Bola˜nos C, Luis D. Alfaro1 JCC, Guimarães-Rodrigues FH, Carrillo E, Sutherland C, et al. Seasonal use of waterholes and pathways by macrofauna in the dry forest of Costa Rica. J Trop Ecol. 2019;35(2):68-73. doi: 10.1017/S0266467418000457. PubMed PMID: WOS:000461472900003.

27. Matsukawa A, Ahmad AH, Kohshima S. Social structure and reproduction of long-tailed porcupine (Trichys fasciculata). Mammalia. 2019;83(1):23-33. doi: 10.1515/mammalia-2017-0104. PubMed PMID: WOS:000453693000003.

28. Sunarto S, Kelly MJ, Parakkasi K, Hutajulu MB. Cat coexistence in central Sumatra: ecological characteristics, spatial and temporal overlap, and implications for management. J Zool. 2015;296(2):104-115. doi: 10.1111/jzo.12218.

29. Rich LN, Miller DAW, Muñoz DJ, Robinson HS, McNutt JW, Kelly MJ. Sampling design and analytical advances allow for simultaneous density estimation of seven sympatric carnivore species from camera trap data. Biol Conserv. 2019;233:12-20.

doi: 10.1016/j.biocon.2019.02.018. PubMed PMID: WOS:000467513500002.

30. BLANC L, MARBOUTIN E, GATTI S, GIMENEZ O. Abundance of rare and elusive species: Empirical investigation of closed versus spatially explicit capture–recapture models with lynx as a case study &dagger. J Wildlife Manage. 2013;77(2):372-378. doi: 10.1002/jwmg.453.

31. Efford MG, Fewster RM. Estimating population size by spatially explicit capture-recapture. Oikos. 2013;122(6):918-928. doi: 10.1111/j.1600-0706.2012.20440.x.

32. Bashir T, Bhattacharya T, Poudyal K, Sathyakumar S, Qureshi Q. Estimating leopard cat Prionailurus bengalensis densities using photographic captures and recaptures. Wildlife Biol. 2013;19(4):462-472. doi: 10.2981/12-098.

33. Alexander JS. Advancing research and conservation of snow leopards in the Qilianshan mountains of China: management and methodological implications [Doctoral dissertation]: Beijing Forestry University; 2015.

34. O'Brien TG, Kinnaird MF, Wibisono HT. Crouching tigers, hidden prey: Sumatran tiger and prey populations in a tropical forest landscape. Anim Conserv. 2003;6(2):131-139. doi: 10.1017/S1367943003003172

35. Alexander JS, Shi K, Tallents LA, Riordan P. On the high trail: examining determinants of site use by the Endangered snow leopard Panthera uncia in Qilianshan, China. Oryx. 2016;50(2):231-238. doi: 10.1017/S0030605315001027. PubMed PMID: WOS:000372525900014.

36. Bohm T, Hofer H. Population numbers, density and activity patterns of servals in savannah patches of Odzala-Kokoua National Park, Republic of Congo. Afr J Ecol.

2018;56(4):841-849. doi: 10.1111/aje.12520. PubMed PMID: WOS:000451574200017.

37. Goswami VR, Madhusudan MD, Karanth KU. Application of photographic capture-recapture modelling to estimate demographic parameters for male Asian elephants. Anim Conserv. 2007;10(3):391-399. doi: 10.1111/j.1469-1795.2007.00124.x.

38. Tye CA, Greene DU, Giuliano WM, Mccleery RA. Using camera-trap photographs to identify individual fox squirrels (Sciurus niger) in the Southeastern United States. Wildlife Soc B. 2015;39(3):645-650. doi: 10.1002/wsb.573.

39. Zheng X, Owen MA, Nie Y, Hu Y, Swaisgood RR, L.Yan, et al. Individual identification of wild giant pandas from camera trap photos - a systematic and hierarchical approach. J Zool. 2016;300:247-256. doi: 10.1111/jzo.12377.

40. Hedges S, Johnson A, Ahlering M, Tyson M, Eggert LS. Accuracy, precision, and cost-effectiveness of conventional dung density and fecal DNA based survey methods to estimate Asian elephant (Elephas maximus) population size and structure. Biol Conserv. 2013;159:101-108. doi: 10.1016/j.biocon.2012.12.010. PubMed PMID: WOS:000320096300015.

41. Gupta M, Joshi A, Vidya TNC. Effects of social organization, trap arrangement and density, sampling scale, and population density on bias in population size estimation using some common mark-recapture estimators. Plos One. 2017;12(3):e0173609. doi: 10.1371/journal.pone.0173609. PubMed PMID: WOS:000396710300013.

42. Zhiqiang Y, Li Z. Population and activity characteristic of wild Asian elephant in the Wild Elephant Valley, Xishuangbanna National Nature Reserve, Yunnan, China. Acta Theriologica Sinica. 2006;26(4):359-367.
43. Chaiyarat R, Youngpoy N, Prempree P. Wild Asian elephant Elephas maximus population in Salakpra Wildlife Sanctuary, Thailand. Endanger Species Res. 2015;29(2):95-102. doi: 10.3354/esr00706. PubMed PMID: WOS:000367451200001.
44. Fan Y, Li Z. Population genetic structure and population genetic diversity analysis based on mitochondrial DNA of Asian elephant Elephas maximus in Chhina. Acta Theriologica Sinica. 2012;32(2):90-100. doi: 10.16829/j.slxb.2012.02.002.
45. Limin F, Zhisheng W, Liu L, Shaobing Y, Bin Z, Chunhua L, et al. Habitat selection in dry season of Asian elephant (Elephas maximus) and conservation strategies in Nangunhe National Nature Reserve, Yunnan, China. Acta Theriologica Sinica. 2010;30(1):1-10. doi: 10.1682/j.slxb.2001.01.001.
46. Reddy CS, Unnikrishnan A, Asra M, Manikandan TM, R.Jaishanker. Spatial Conservation Prioritisation of Threatened Forest Ecosystems in Myanmar. Journal of the Indian Society of Remote Sensing. 2019;47(10):1737-1749. doi: 10.1007/s12524-019-01025-2.
47. Peng L, Hui W, Liu L, Jing L, Li Z. Habitat evaluation for Asian elephants ( Elephas maximus ) in Lincang: Conservation planning for an extremely small population of elephants in China. Biol Conserv. 2016;198:113-121. doi: 10.1016/j.biocon.2016.04.005.

48. Yong-jing T, Zhi-sheng W, Gui-lian J, Xuan Z, Yuan H, Zheng-ling L, et al. Population dynamics analysis of Asian elephant in Nangunhe river basin. Forestry Construction. 2019;6:101-106.

49. Wijeyamohan S, Treiber K, Schmitt D, Santiapillai C. A Visual System for Scoring Body Condition of Asian Elephants (Elephas maximus). Zoo Biol. 2015;34(1):53-59. doi: 10.1002/zoo.21181. PubMed PMID: WOS:000349223800007.

50. Fernando P, Janaka HK, Ekanayaka SKK, Nishantha HG, Pastorini J. A simple method for assessing elephant body condition. Gajah. 2009;31:29-31. doi: 10.5167/uzh-32262.

51. Morfeld KA, Lehnhardt J, Alligood C, Bolling J, Brown JL. Development of a body condition scoring index for female African elephants validated by ultrasound measurements of subcutaneous fat. Plos One. 2014;9(4):e93802. doi: 10.1371/journal.pone.0093802.

52. Barrett SCH, Kohn JR, Falk DA, Holsinger KE. Genetic and evolutionary consequences of small population size in plants: implications for conservation. Genetics & Conservation of Rare Plants. 1991.

53. Ruping W, Lin L. The extinction vortex of small population. Journal of Biology. 2008;25(6):14-16.

54. Tang F, Du F, Sun G. Investigation report on background resources of Nangunhe National Nature Reserve, Yunnan China Forestry Press. 2015.

55. Varma S, Pittet A, Jamadagni HS. Experimenting usage of camera-traps-for population dynamics study of the Asian elephant Elephas maximus in southern

India. Curr Sci India. 2006;91(3):324-331. doi: 10.1126/science.1127215 PubMed PMID: WOS:000239880100021.

56. Silva Sd, Ranjeewa A, Weerakoon D. Demography of Asian elephants (Elephas maximus) at Uda Walawe National Park, Sri Lanka based on identified individuals. Biol Conserv. 2011;144(5):1742-1752. doi: 10.1016/j.biocon.2011.03.011. PubMed PMID: WOS:000292668200059.

57. Vidya TNC, Prasad D, Ghosh A. Individual Identification in Asian Elephants. Gajah. 2014;40:3-17.

58. Goswami VR, Lauretta MV, Madhusudan MD, Karanth KU. Optimizing individual identification and survey effort for photographic capture-recapture sampling of species with temporally variable morphological traits. Anim Conserv. 2012;15(2):174-183. doi: 10.1111/j.1469-1795.2011.00501.x.

59. Borchers DL, Efford MG. Spatially explicit maximum likelihood methods for capture-recapture studies. Biometrics. 2008;64(2):377-385. doi: 10.1111/j.1541-0420.2007.00927.x. PubMed PMID: WOS:000255959500007.

60. Gardner B, Reppucci J, Lucherini M, Royle JA. Spatially explicit inference for open populations: estimating demographic parameters from camera‐trap studies. Ecology. 2010;91(11):3376-3383. doi: 10.2307/20788170.

61. López-Bao JV, Godinho R, Pacheco C, Lema FJ, García E, Llaneza L, et al. Toward reliable population estimates of wolves by combining spatial capture-recapture models and non-invasive DNA monitoring. Sci Rep-Uk. 2018;8(1):2177. doi: 10.1038/s41598-018-20675-9

62. Royle JA, Chandler RB, Sollman R, Gardner B. Spatial Capture-Recapture: Academic Press; 2014.

63. Royle JA, Gardner B. Hierarchical spatial capture-recapture models for estimating density from trapping arrays. In: O’Connell A.F., Nichols J.D., Karanth K.U. (eds) Camera Traps in Animal Ecology. : Springer, Tokyo; 2011.

64. Li Z, Kang A, Lang J, Xue Y, Ren Y, Zhu Z, et al. On the assessment of big cats and their prey populations based on camera trap data. Biodiversity Science. 2014;22(6):725-732. doi: 10.3724/SP.J.1003.2014.14079

65. Ling Q. Habitat Selection by Asian Elephant (Elephas maximus) in Nangunhe Nature Reserve, Yunnan, China [Masters]: Northwest University; 2007.

66. Peiyan L, Jianhao W. WIld elephant population in China. Chinese Journal of Wildlife. 1983:27-29.

67. Zhi-hong W, Heng-zhen Z, Wen-lan Z, Xuan Z, Wen-bian T, Zheng-ling L, et al. An analysis of population dynamic on the Xishuangbanna Menghai-Pu'er Lancang Asian elephant population. Forestry Construction. 2019;(06):79-84.

68. Guo B. Investigation and protection advice of wild elephant in Nangunhe reserve. YUnnan Forestry 1989.

69. Wright LI, Tregenza T, Hosken DJ. Inbreeding, inbreeding depression and extinction. Conserv Genet. 2008;9(4):833-843. doi: 10.1007/s10592-007-9405-0

70. Paulraj S, Subramanian C. Skewed sex ratio in elephant population - How it may affect the demography and population growth? A case study in Mudumalai Wildlife Sanctuary and National Park, Tamil Nadu, Southern India. Indian Forester.

2000;126:1046-1053.

71. Ramakrishnan U, J.A.Santosh, R.Sukumar. The population and conservation status of Asian elephants in the Periyar Tiger Reserve,Southern India. Curr Sci India. 1998;74(2):110-113.

72. Vidya TNC, Kumar VR, Arivazhagan C, Sukumar R. Application of molecular sexing to free-ranging Asian elephant (Elephas maximus) populations in southern India. Curr Sci India. 2003;85(7):1074-1077. PubMed PMID: WOS:000186263400043.

73. Kurt F, Hartl G, Tiedemann R. Tuskless bulls in Asian elephant Elephas maximus . History and population genetics of a man-made phenomenon. Acta Theriol. 1995;40:125-143. doi: 10.4098/AT.arch.95-51.

74. Guo B, Lan D. Status of wildlife resources in Nangunhe reserve. Chinese Journal of Wildlife. 1999;(4):46-47.

75. Yuming Y, Fan D. Nangunhe National Nature Reserve in China. Yunnan Science Publication. 2004.

76. Silva Sd, Webber CE, Weerathunga US, Pushpakumara TV, Weerakoon DK, Wittemyer G. Demographic Variables for Wild Asian Elephants Using Longitudinal Observations. Plos One. 2013;8:e82788. doi: 10.1371/journal.pone.0082788.

77. Silveira L, Anah TAJ, Diniz-Filho JAF. Camera trap, line transect census and track surveys: a comparative evaluation. Biol Conserv. 2003;114(3):351-355. doi: 10.1016/S0006-3207(03)00063-6

78. Verbrugge LM, Taylor RB. Consequences of population density and size. 1980;16(2):135-160. doi: 10.1177/107808748001600202

79. Goswami VR, Yadava MK, Vasudev D, Prasad PK, Sharma P, Jathanna D. Towards a reliable assessment of Asian elephant population parameters: the application of photographic spatial capture– recapture sampling in a priority floodplain ecosystem. Sci Rep-Uk. 2019;9(1):1-10. doi: 10.1038/s41598-019-44795-y.

80. Royle JA, Nichols JD, Karanth KU, Gopalaswamy AM. A hierarchical model for estimating density in camera-trap studies. J Appl Ecol. 2010;46(1):118-127. doi: 10.1111/j.1365-2664.2008.01578.x.

81. Surendra Varma, Nguyen Xuan D, Thanh TV, Sukumar R. The Asian elephants Elephas maximus of Cat Tien National Park, Vietnam: Status and conservation of a vanishing population. Oryx. 2008;42(1):92-99. doi: 10.1017/S0030605308010090.

82. Sulumar R. Study of Elephant in Buxa Tiger Reserve and adjoining areas of northern West Bengal and preparation of conservation Action plan2003.

83. Yuming Y, Genmin W, Zhisheng Z, Zhongli W. Wild elephant population in Nangunhe reserve. Forest Inventory & Planning. 1982.

84. Wemmer C, Krishnamurthy V, Shrestha S, Hayek LA, Thant M, Nanjappa KA. Assessment of body condition in Asian elephants (Elephas maximus). Zoo Biol. 2006;25(3):187-200. doi: 10.1002/zoo.20099. PubMed PMID: WOS:000238705300004.

85. Christie JL, Hewson CJ, Riley CB, McNiven MA, Dohoo IR, Bate LA. Management factors affecting stereotypes and body condition score in nonracing horses in Prince Edward Island. The Canadian veterinary journal La revue vétérinaire canadienne. 2006;47:136-143. doi: 10.1080/03079450500465817
86. Morfeld KA, Meehan CL, Hogan JN, Brown JL. Assessment of Body Condition in African (Loxodonta africana) and Asian (Elephas maximus) Elephants in North American Zoos and Management Practices Associated with High Body Condition Scores. Plos One. 2016;11(7):e0155146. doi: 10.1371/journal.pone.0155146. PubMed PMID: WOS:000379579500009.
87. Ramesh T, Sankar K, Qureshi Q, Kalle R. Assessment of wild Asiatic elephant (Elephas maximus indicus) body condition by simple scoring method in a tropical deciduous forest of Western Ghats, Southern India. Wildlife Biology in Practice. 2011;7(2):47-54. doi: 10.2461/wbp.2011.7.17.
88. Srinivasaiah N, Kumar V, Vaidyanathan S, Sukumar R, Sinha A. All-Male Groups in Asian Elephants: A Novel, Adaptive Social Strategy in Increasingly Anthropogenic Landscapes of Southern India. Sci Rep-Uk. 2019;9:8678. doi: 10.1038/s41598-019-45130-1.
89. Guozheng S, Guangyuan Z, Zicheng Y, Mengjun W. Research progress and direction of human elephant conflict mitigation projects. Forestry Construction. 2019;6:5-9.
90. Pokharel SS, Seshagiri PB, Sukumar R. Assessment of season-dependent body condition scores in relation to faecal glucocorticoid metabolites in free-ranging

Asian elephants. Conserv Physiol. 2017;5(1):cox039. doi: 10.1093/conphys/cox039. PubMed PMID: WOS:000404752400001.

91. Furlan E, J.Stoklosa, J.Griffiths, N.Gust, Ellis R, Huggins RM, et al. Small population size and extremely low levels of genetic diversity in island populations of the platypus, Ornithorhynchus anatinus. Ecol Evol. 2012;2:844-857. doi: 10.1002/ece3.195.

92. Frankham R, Ballou JD, Briscoe DA. Introduction to conservation genetics, second edition. 2010.

93. Sukumar R. Minimum viable population for Asian elephant conservation. A Week with Elephants(eds JC Daniel & H Datye). 1995:279-288.

94. Linping Z. The "Elephant Worshipping Festival" of the Wa People Along the Nangunhe River in Cangyuan. Journal of Lincang Education College. 2005;14(3):84-85.

95. Li W, Yu Y, Liu P, Tang R, Dai Y, Li L, et al. Identifying climate refugia and its potential impact on small population of Asian elephant (Elephas maximus) in China. Glob Ecol Conserv. 2019;19:e00664. doi: 10.1016/j.gecco.2019.e00664.

96. Xue—dong L, Ming—chun P, Chong—yun W, Yong—jie L, He—xiang D, Xiao—hua L, et al. Assessment of Asian elephant habitat quality status in Nangunhe valley, China. Journal of Yunnan University. 2008;30(4):415-423. doi: 10.3321/j.issn:0258-7971.2008.04.018.

97. Eden MV, Ellis E, Bruyere BL. The Influence of Human–Elephant Conflict on Electric Fence Management and Perception Among Different Rural Communities

in Laikipia County, Kenya. Human Dimensions of Wildlife. 2016;21(4):283-296. doi: 10.1080/10871209.2016.1149746.

# Supporting information

**S1 Table. A simplified database of Asian elephant in NNNR**

**S2 Table. Historical elephant population in NNNR estimated by different methods**

Fig 1

Click here to access/download;Figure;Fig 1.tif

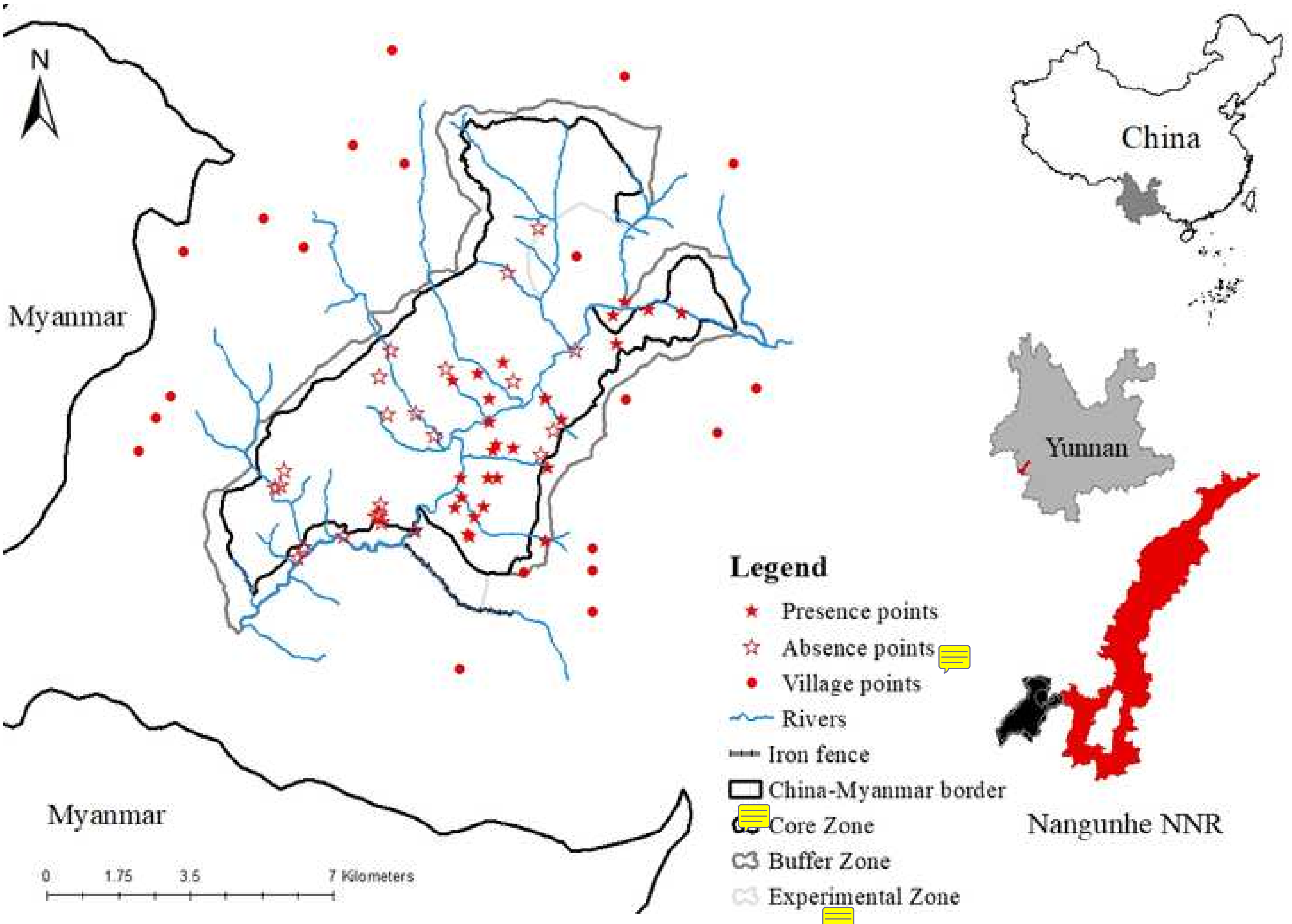

Fig 2

Click here to access/download;Figure;Fig 2.tif

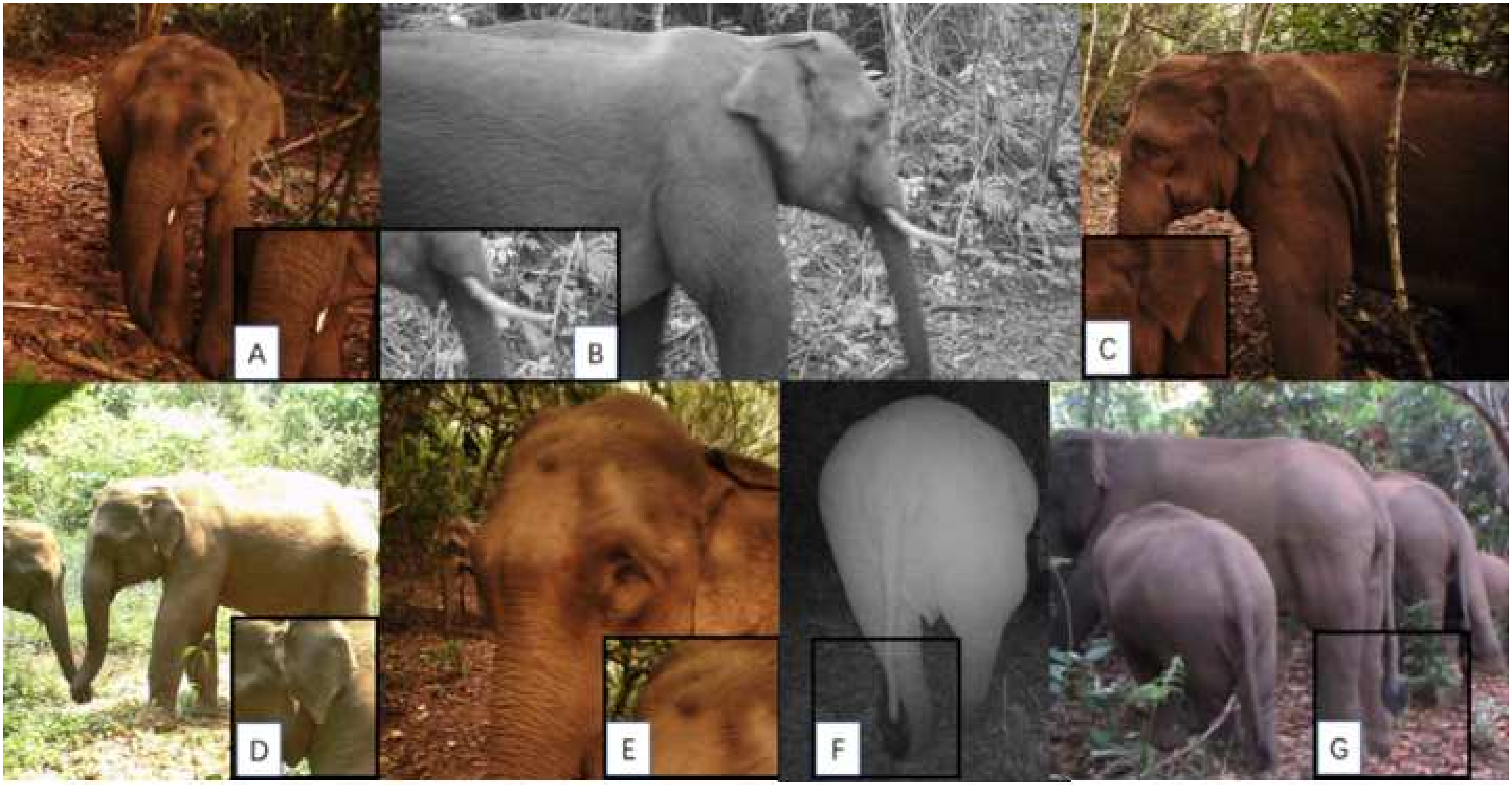

Fig 3

Click here to access/download;Figure;Fig 3.tif

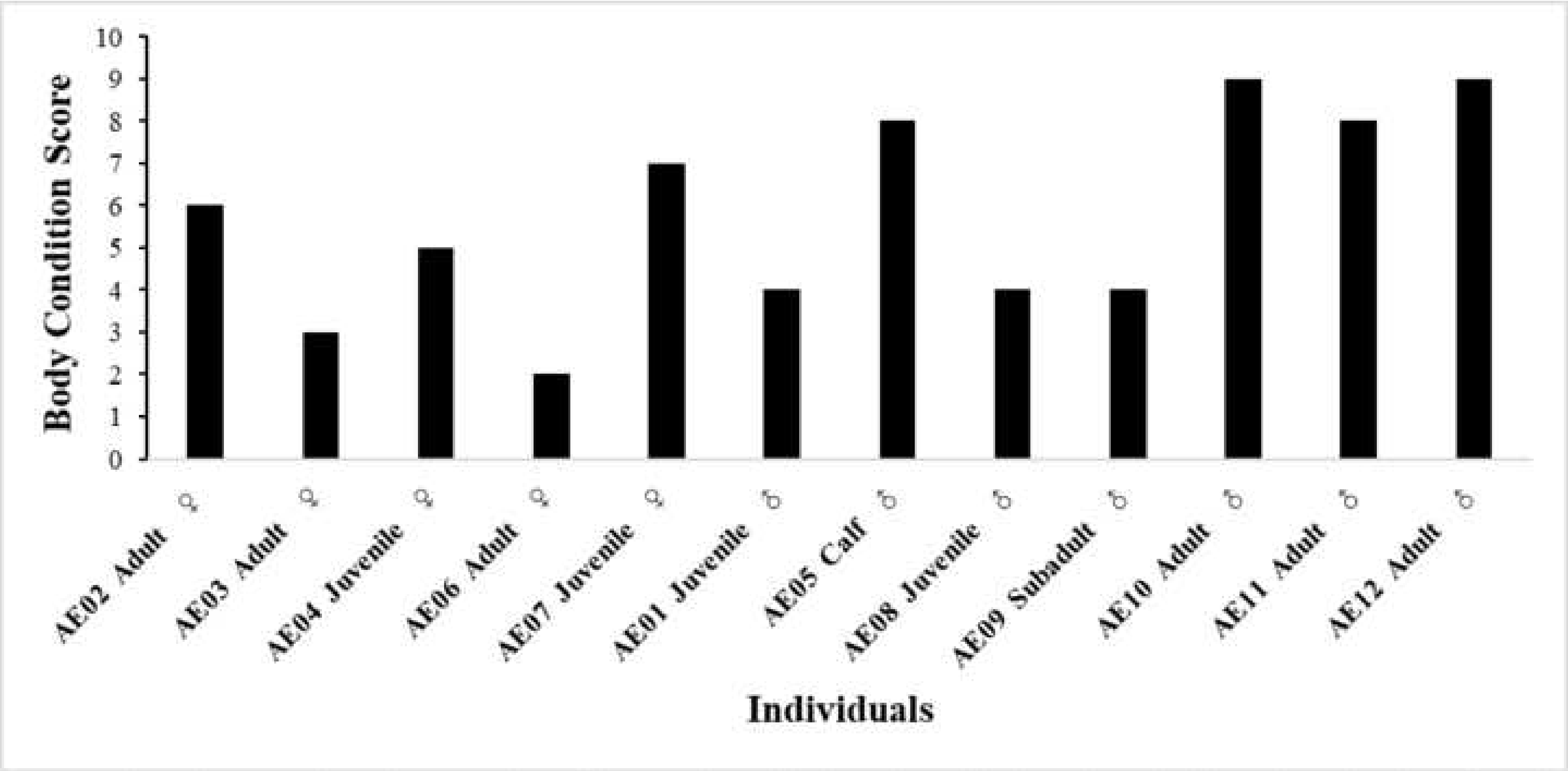

S1 Table

Click here to access/download
**Supporting Information**
S1 Table.docx

S2 Table

Click here to access/download
**Supporting Information**
S2 Table.docx
