# Supplementary material for: Assessing population structure and body condition to inform conservation strategies for a small isolated Asian elephant (Elephas maximus) population in southwest China
Source: PLoS One. 2021 Mar 9;16(3):e0248210. doi: 10.1371/journal.pone.0248210 (PMC7942997; doi:10.1371/journal.pone.0248210)
Supplement: Attachment — Submitted filename: PONE-D-20-11590_R1 revSC.pdf [file pone.0248210.s005.pdf]

PLOS ONE

Population structure and body condition assessment to inform conservation strategies for a small isolated Asian elephant population in southwest China

--Manuscript Draft--

**Manuscript Number:**	PONE-D-20-11590R1
**Article Type:**	Research Article
**Full Title:**	Population structure and body condition assessment to inform conservation strategies for a small isolated Asian elephant population in southwest China
**Short Title:**	Population structure and body condition of Asian elephant in southwest China
**Corresponding Author:**	Kun Shi<br>Beijing Forestry University<br>Beijing, CHINA
**Keywords:**	Asian elephant; population size; population density; population structure; camera traps; body condition scoring
**Abstract:**	The Asian elephant (Elephas maximus) population in Nangunhe National Nature Reserve represents a unique evolutionary branch in China, which has been isolated for more than twenty years from other neighboring populations in Myanmar. The scarcity of information on population structure, sex ratio and body condition make it difficult to develop effective conservation measures for this elephant population. Twelve individuals were identified from 3,860 valid elephant images obtained from February to June of 2018 (5,942 sampling effort nights) at 52 camera sites. Three adult females, three adult males, one subadult male, two juvenile females, two juvenile males and one calf male were identified. The ratio of adult females to adult males was 1:1, and the ratio of reproductive ability was 1:0.67, indicating the scarcity of reproductive females as an important limiting factor to population growth. A population density of 5.32 ± 1.56 elephants/100 km^2 was estimated by using Spatially Explicit Capture Recapture models. The health condition of this elephant population was assessed by using an 11-points scale of body condition scoring (BCS) method which showed that this population had a medium body condition with an average of 5.75 (n=12, range 2-9). This study finds that this isolated population has an extremely small size and an inverted pyramid age structure and therefore the population is at high risk of extinction. As such, this population should be of top conservation priority among the few remnant populations in China. The database established in this study can provide researchers with valuable information to set up efficient conservation schemes.
**Order of Authors:**	Kun Shi
	Yakuan Sun
	Ying Chen
	Juan José Díaz-Sacco
**Response to Reviewers:**	Specific Comments of Reviewer #1:<br>Comment 1: On line 220, the buffer zone is said to be 5km around the outermost camera sites. There was no citation here, so what was the justification for using 5km and not a different value?<br>Response: Considering the small size of the elephant distribution, with the east and west boundary being no wider than 10 km, we therefore defined our study area as extending 5 km beyond all camera stations, corresponding to an area of 255.8 km2. We cited one reference. Please go to P12, L226-228.<br>Comment 2: Lines 245-246 Please list all the model covariates and provide some description e.g. was age a categorical factor in the models? Was BCS considered as a continuous variable for the purposes of getting the correlation coefficients?<br>Response: The covariates include sex, age and whether the elephant was solitary or in the herd. BCS was not a continuous variable. Please go to P13, L260-262.<br>Comment 3: It is unclear how many photographs were used to calculate the scores for each elephant. If multiple, were they all from around the same time and consistent? If the score is an average or not needs to be stated.<br>Response: Five of the clearest images for each physical characteristic were selected to

assess the body condition. Thirty images were used to calculate the comprehensive scores for each individual. Please go to P13, L244-246.
Comment 4: Lines 285-287 Asian elephants may allolactate (Rapaport, L. & Haight, J. 1987 Some observations regarding allomaternal caretaking among captive Asian elephants (Elephas maximus). J. Mammal. 68, 438–442), and there may be other forms of allomothering too (Lahdenperä M., Mar, K.U., Lummaa V. 2016 Nearby grandmother enhances calf survival and reproduction in Asian elephants. Scientific Reports 6: 27213; Lynch E. C., Lummaa, V., Htut W., Lahdenperä M. 2019 Evolutionary significance of maternal kinship in a long-lived mammal. Phil. Trans. R. Soc B. 374: 20180067) – I'm not sure that any relationship can be considered 'definite'.
Response: Yes, we lost sight of that. Only the DNA methods can tell us the true relationship among individuals in this population. The word "definitely" has been deleted. We discussed this situation in the Discussion section. P16, L297-301; P20, L370-379.
Specific Comments of Reviewer #2:
Comment 1: L. 34: Insert BCS here for the first time.
Response: We have inserted BCS. This sentence has been changed to "The health condition of this elephant population was assessed by using an 11-points scale of Body Condition Scoring (BCS) method which showed that this population had a medium body condition with an average of 5.75 (n=12, range 2-9)." Please go to P2, L33-35.
Comment 2: The fact that individuals had such low BCS should be highlighted in the abstract (if space allows).
Response: We have rewritten this sentence. Please see the response to comment 1 or P2, L33-35.
Comment 3: L. 44: End the sentence at "dispersal" and start a new sentence about elephants as umbrella species.
Response: This sentence has been changed as you recommended. Please go to P3, L42-46.
Comment 4: L.49: When you mention that the species is listed as Endangered in IUCN Red List, and then that its population is decreasing, you make a tautological argument. Any endangered population is decreasing. You should rather change the sentence and say that however important it is for conservation (as an umbrella species and an ecosystem engineer), there is a problem with the species, which population is decreasing, hence justifying its endangered status.
Response: Yes, we believe that this is more logical. This sentence has been changed to make it simpler. "It is a Class I Key Protected Species in China and listed as an Endangered under the IUCN Red List.". Please go to P3, L48-49.
Comment 5: L.77: Puma is not a good example, as they do not have spots or stripes. I would also keep this list more general, citing genera that have spots or stripes, such as tigers, leopards, giraffes, etc.
Response: Puma has been replaced by leopard cats since they have stripes on their bodies. Please go to P4, L76.
Comment 6: L.88: There is a jump between the two sentences (no logical sequence). Maybe you could close the previous argument by saying that for NNNR there are not such data available. Then you could start a new paragraph on this isolated population, its genetic status, etc.
Response: A new paragraph has been created. Please go to P5, L99.
Comment 7: L.99-108: This new paragraph also makes a jump. You should place it in the series of paragraphs describing how trap cameras (and photographs more broadly) are another way that tool can be useful in conservation and management.
Response: We moved the BCS paragraph forward placing it behind the paragraph that introduces individual identification by camera traps. Please go to P4-5, L81-92.
Comment 8: L.104-105: It is unclear why BCS is most meaningful "especially [in] small isolated populations". Explain it or delete it.
Response: We deleted the word "small". Now the sentence looks like "While BCS is individual-based, it is most meaningful when applied to a population, as an early indicator of the impact of management actions on the average health condition of a population.". Please go to P5, L87-90.
Comment 9: L. 107-108. Delete this sentence. It is of very little relevance for a journal with a broad, worldwide audience.
Response: This sentence has been deleted. Please go to P5, L92.
Comment 10: L.109-116: There is one more reason to move the previous paragraph upward: this paragraph is connected to the one preceding the previous one (i.e., after

	the paragraph l. 82-98).<br>Response: That is correct. After moving the previous paragraph upward, this paragraph is now well connected to the previous and following paragraphs. Please go to P6, L109-115.<br>Comment 11: L. 114-115: “for the development of a successful project focusing on the introduction of individual elephants from other populations”. That is the kind of sentence that does not serve you well. You talk about introducing individuals, but you have provided no evidence that the conditions to ensure the survival of introduced animals are met in the area. In many areas, habitat (quantity or quality) or negative relationships with humans do not ensure survival, making the prospect of reintroduction a loosing strategy.<br>Response: We did not obtain enough evidence to support the introduction of individuals as a successful plan for the survival of this small population. Thus, we deleted any mention about the introduction of elephants throughout the manuscript. Please go to P6, L112-115 and Conservation Recommendation section.<br>Comment 12: L. 120: I would change “to analyze the factors” to reflect that it is indeed a discussion of some factors that might have affected the population that you will present.<br>Response: This sentence has been changed to “We sought to understand which factors might have affected this populations size in recent years in order to develop targeted and effective protection strategies.”. Please go to P6, L118-119.<br>Comment 13: L. 122: Why in China only and not in other parts of the species distribution?<br>Response: We have deleted “in China”. The new sentence is “Furthermore our approaches can be applied to other elephant populations with further implications for global Asian elephant conservation.” Please go to P6, 120-121.<br>Comment 14: L. 134: Give properly the geographical coordinates (including N to the latitude, and W to the longitude).<br>Response: The sentence had been changed to “It occupies 70.82 km2 at latitude 23°13'- 23°19'N, longitude 98°54'- 99°05'E, and the altitude ranges from 510 m to 1747 m.”. P7, L131-133.<br>Comment 15: L. 137: Please give a proper annual precipitation and temperature (a single value), or explain the range you provide.<br>Response: Mean annual precipitation is 351.3 mm in the dry season and 1983.3 mm in the rainy season, while temperature varies at different altitudes. The average monthly temperature range is hottest in July, varying between 12.3-25.5°C and it is coldest in January with values between 2.8-14.3 ℃. (Reference: NGH scientific report. No single value for annual temperature, just range value.) Please go to P7, L134-137.<br>Comment 16: L. 138: Why natural in “natural tropical forest”?<br>Response: It should be a tropical forest. We made a little mistake when we read the corresponding reference. Please go to P7, L138.<br>Comment 17: L.140: By evergreen vegetation do you mean evergreen broadleaf species, in opposition to coniferous species?<br>Response: Yes, it is evergreen broadleaf species. We have added “broadleaf” in the manuscript. Please go to P7, L141.<br>Comment 18: L. 148: Use a better title, such as survey design or camera trap set-up.<br>Response: The title has been changed to “Camera trap set-up”. Please go to P7, L148.<br>Comment 19: L. 151: “along the animal trails with forest gaps where there were signs of elephants”. Unclear. Did you set up the cameras in forest gaps that were crossed by elephant trails? If so replace “with” by “in”. In any case, review this.<br>Response: Actually, we set up the cameras in trails and forest gaps, but the gaps were often located in trails. So, here we replace “with” by “and” to make it clearer. Please go to P8, L155-157.<br>Comment 20: L. 152: “as well as other activities”. You cannot remain vague here; mention them all.<br>Response: The elephant signs included lying sites, foraging sites, footprints, dungs and urine marks. Please go to P8, L155-157.<br>Comment 21: L. 152: Explain why you avoided certain areas. Was it because elephants do not use them, or because you had concerns for the safety of people in the field?<br>Response: We avoided the northwestern part of the reserve as the topography is tough, with steep cliffs of up to 1747 meters, and historically, there have not been any records of elephants in this area. Please go to P8, L159-162.<br>Comment 22: L. 149-164: In this section, there is information missing. As far as I

	understand you had one camera per site, but one sentence mentions that "at each site, camera traps were operated...", which suggests there were several cameras at each site. Also, there is information missing regarding the number of sites or cameras, the minimum distance that could separate two sites (because there could be spatial autocorrelation in your data). Finally, you should mention the camera model(s) you used in your study. Elephants are not small nor super fast, so trigger time should not be an issue, but still, getting information on the material used is necessary for replication purposes.<br>Response: A total of 52 cameras at 52 stations or sites (P8, L150-151). We set one camera trap at each camera site, but the number of camera sites varied in each grid. More than one camera was installed in the grids that contained lots of elephant signs (P8, L170-171), and the minimum distance between two cameras was 200 meters (P8, L158-159). The camera model was Ltl Acorn 6210 (P8, L150) and we programmed the camera to take sequential images consisting of three photographs plus one 12 second video (P8, L168-170).<br>Comment 23: L. 166-169: The whole sentence needs to be rewritten for better clarity. After reading it, I am left with doubt about what constituted an independent event. I think I understood that an independent event could include several photographs of various individuals, with at least 5 h between such photographic sequences to make it temporally independent, but it is far from clear.<br>Response: We have provided a more detailed explanation about independent events than in the previous manuscript version. Please see P9, L175-181.<br>Comment 24: L. 177: Did you determine the sex in juveniles and calves? If so, how did you do that?<br>Response: Yes, we made it clearer in Table 1. Please see P10, L199.<br>Comment 25: L. 185-187: How do you know they did not change? Ear can be teared at any moment; a tusk could be broken digging up... maybe it is the way it is written that makes it not very convincing. If you have an elephant with a particular tear in the ear, it is easy to "recapture" it, but if there was no marking, I am not sure how this could be reliable. I think a better explanation would help.<br>Response: Yes, that is a better explanation that would be more convincing. Morphological characters including facial bones, ear, tusk, back and tail hair, old scars and injuries, as well as physical characteristics related to sex and age classes, confirmed the reliability of identification results. Please see P10, L196-198.<br>Comment 26: Table 1: 1) Regarding sexing, as mentioned earlier I think male and female should be labeled properly adult female and adult male, as the diagnostic characteristics are found in adult (and possibly subadult) individuals only. 2) “Sunken prominence”. How can a prominence be sunken? 3) Your classes for tusk length are ok, but I am not ok with the fact that you do not explain how you classified them in each of these categories. I am sure you had a criteria to decide (such as tusk length relative to the trunk).<br>Response: 1): We had rearranged sex categories as adult female, adult male, subadult male, juvenile female, juvenile male and calf male. 2): It was a description mistake. The sentence has been changed to “Extent of depression”. 3): Yes, we have a criterion that uses trunk as a reference to classify the tusk length. For more details please go to Table 1 on P10, L199.<br>Comment 27: L. 197: “The movement pattern of individuals activity center in study area”. This is unclear. Do you mean “The movement pattern of individual activity centers in the study area”, i.e., the movement pattern of the activity centers of a given individual evaluated at different times (sessions)? Sorry, but the devil is in the details!<br>Response: Yes, the model can evaluate the movement pattern according to the capture history. We missed the time limitation. The sentence has been changed to “The state model describes statistically the mechanisms determining the distribution of the animals in the study area, while the observation model quantifies the probabilities of detection or capture, given the searched region, their locations and characteristics at different sessions”. Please go to P11, L205-209.<br>Comment 28: L. 198-200: "the probability of detecting an individual at a particular detector (…) to the distance of the detector" makes no sense. Please rewrite.<br>Response: The sentence has been rewritten combining it with the forward sentence. Please see the response of comment 27 or onP11, L205-209.<br>Comment 29: L. 206 and elsewhere in this section: Use trap or detector, or explain that detectors are traps, but remain consistent throughout your explanations.<br>Response: All the words corresponding to “detectors” were replaced by “traps”. Please go to P11, L212; L214; L217.

Powered by Editorial Manager® and ProduXion Manager® from Aries Systems Corporation

	Comment 30: L. 217: Again, there is a problem with the phrasing and it is unclear what you mean.<br>Response: This sentence has been deleted. Please go to L12, L224.<br>Comment 31: L. 219-221: The whole sentence needs to be rephrased so that the reader can understand exactly what you did. Also "from the outermost x and y coordinates of the camera sites" is very unclear. A camera site is not a discrete point in space???<br>Response: The sentence has been changed to "Considering the small size of the elephant distribution, with the east and west boundary being no wider than 10 km, we therefore defined our study area as extending 5 km beyond all camera stations, corresponding to an area of 255.8 km2.". Please go to P12, L226-228.<br>Comment 32: L. 224: Again, this is unclear. Is the file named "Capture histories"? Please check the sentence.<br>Response: Yes, the file is named "capture histories". The sentence has been changed. Please go to P12, L231.<br>Comment 33: L. 225: Is it "occasion" (you haven't defined it previously) or "event"? Also, earlier you defined individual events as being separated by at least 5 hours. This means that several individual events could occur on a same day. How can your data base account for that if your time unit is "day"?<br>Response: "Occasion" was defined as each event, as well as the "sequence". The cameras captured several events, accounting for different occasions. Please go to P12, L232.<br>Comment 34: L. 227: "whole data as five different sessions". Again, it is unclear. Do you mean you divided your 4-month sampling period in 5 (i.e. each session would last 24 days)?<br>Response: Five different sessions, corresponding to each of the months and one corresponding to the whole survey period, were used to run the model to see the estimation accuracy under different detection efforts. P12, L233-236.<br>Comment 35: L. 236-244: The first of these five sentences is very unclear. Further, if you followed the method developed by 49 or 50, just cite them, and mention that the part on which the scoring focuses are the ribs, shoulders, etc. and say why. Usually it is because these parts can accumulate fat.<br>Response: We added more information to describe the reasons we choose ribs, shoulders and other body parts as scoring criterion. Please go to P13, L246-252.<br>Comment 36: L. 244: Before mentioning which software you used to perform your analyses, say what kind of analysis you performed.<br>Response: This sentence has been deleted. Please go to P13, L260.<br>Comment 37: L. 244-245: Which type of correlation coefficient?<br>Response: It is Pearson Correlation Coefficient. P13, L260.<br>Comment 38: L. 245: "ratios of various covariates": why ratios? Age is not a ratio, nor is sex or the solitary vs group status.<br>Response: The word "ratio" has been deleted. Please go to P13, L260-262.<br>Comment 39: L. 246: "whether an individual was present or not in the herd". Here you lost me... do you mean you checked the correlation between BCS and whether an individual was solitary or in herd? (note that this is not what you have written; you have written "the herd")<br>Response: Yes, we wanted to divide individual elephants into those that were solitary and those that were part of a herd to see if this factor could affect the BCS. Please go to P13, L260-262.<br>Comment 40: Table 2: 1) Revise the title of this table. 2) Characters is incorrect here and in several places in the manuscript. The correct word here is characteristics.<br>Response: 1): The title has been changed to "Table 2. Characteristics used to assess the body condition in Asian elephant, NNNR". 2): All the words "characters" have been changed to "characteristics". Please go to P13, L263 to see Table 2.<br>Comment 41: L. 250-253: This is much better suited in Methods. I kept wondering how you set up your cameras, how many, and here is the answer. Yours results should start with what you obtained, not what you did.<br>Response: Yes, these sentences presented in the Methods section were more logical. These sentences have been moved to their current location. P7-8, L149-152.<br>Comment 42: L. 254: You never spoke of video before! Fix that in Methods or delete here if they were not used.<br>Response: We apologize for this mistake. We programmed the camera to take three pictures and a 12 second video. We replaced the words "picture" and "video" by "image" in the Methods section. Please go to P8, L168-170.

Comment 43: L. 261-264: You should revise that sentence. As it is, there is not much difference between the individual that was seen the least and the others. Rather give the mean and the range.
Response: We strongly agree with your suggestions. Currently, there is a brief comment about the capture histories of individual and herds. Please go to P14, L271-274.
Comment 44: Fig. 2: This title does not correspond to what you describe in the text (i.e., the 12 identified individuals). I think it should say something like "Asian elephants individually identified in NNNR park, China". Also, note that the way you cited the figure, one would expect to see the 12 individuals identified. Here you show only half of them.
Response: We strongly agree with your suggestions and the title of "Fig 2" was changed as you recommended. The figure was re-edited to combine all 12 individuals in one picture, and we used black squares to emphasize each individual's prominent characteristic so as to make it clearer for readers. Please go to P15, L285-287 and the file named "Fig2".
Comment 45: Table 3: 1) Put "No" in the column for Tusk for each female. This is all the more important that you later discuss the fact that you might have misclassified some individuals. 2) Put "Herd" in each file for individuals AE01 to AE08.
Response: The changes you proposed have been achieved in Table 3 of the new manuscript. Please go to P14, L275.
Comment 46: L. 272-279: This is interesting, but falls out of the scope of the section (Individual identification).
Response: Yes, we strongly agree with you and thus, this paragraph has been deleted after careful consideration.
Comment 47: L. 283-284: This sentence is not built correctly, and it is unclear what the 1:1 ratio stands for (is it a sex ratio too?). Also, as I mentioned previously, the way you mentioned the criteria for sexing seemed to refer to sexually mature individuals only, so I wonder how you determined sex in juvenile individuals, if the 1:1 ratio is indeed a sex ratio. This needs to be clarified in the methods too!
Response: We wrote this sentence incorrectly. The 1:1 ratio stands for juvenile individuals as we identified two juvenile males and two juvenile females. But we failed to make it clear in the Methods section. Please go to P16, L290-296.
Comment 48: L. 285: What are "containment relationship family units"?
Response: Since we wrote this sentence using the wrong words, it has been deleted. Please go to P16, L297.
Comment 49: Table 4: In the title you mention five sessions, but in the table there are only four sessions, one for each sampled month.
Response: We apologize for the way that it was written. First, we used single month as a single session, and then we used the whole months data as the fifth session. Please see more details in comment 34 or go to P12, L233-236.
Comment 50: L. 306-307: This is the definition of the median!!!!! Please, take this out, or give the mean and here you could assess how skewed the distribution is.
Response: This sentence has been deleted. Please go to P16, L304.
Comment 51: L. 311-312: Say which sex had the highest BCI. Rephrase "solitary or not" (being in a herd or solitary), and explain which status was associated with having a lower or higher BCS.
Response: This information has been combined. Please go to P16-17, L307-313.
Comment 52: Figure 3: The figure does not help much, and a table would probably be more efficient here.
Response: We think a figure would be clearer and more visible than a table. We have improved the figure by changing the order of individuals by placing individuals of the same sex together and ranking them by scores. Please go to the file named "Fig3".
Comment 53: Table 5: For the adult individual that died in 2003, how can you know "it died for its ivory"? Was it killed (with bullets inside the body)? You could find an individual with the tusks removed post-mortem.
Response: We read these two references again and deleted one reference which provided us with incomplete information about an adult male that had died for its ivory without any citation nor detailed explanation. So, the description of "the adult male died for its ivory" has been deleted. Please go to Table 5 on P18, L338.
Comment 54: L. 337-341: This is definitely not of interest for the readership of PLoS One.
Response: These sentences have been deleted. Please go to P19, L364.
Comment 55: L. 343: Use a reference to justify the link you make between the

population structure and its future.
Response: Four references have been added. Please go to P17, L323-327.
Comment 56: L. 343-345: Rephrase that sentence (self contradiction the way it is written), and note that this sentence contradicts the previous one (speaks of a possibly stable population, when earlier you say that it indicates a decline).
Response: This sentence has been rephrased. Please go to P20, L366-369.
Comment 57: L. 345-346: This sentence comes out of the blue. Link it to an argument. You can actually use it to finish the following sentence with this argument of inbreeding depression in small populations.
Response: This sentence has been combined. Please go to comment 56 or P20, L366-369.
Comment 58: L. 351-352: Again, a sentence out of the blue, which has no direct link with your results. What argument do you want to discuss in this paragraph? Start with that, and then develop the argument.
Response: We strongly agree with your comments. This sentence has been changed to "There were two tuskless juveniles that had similar body height as the juvenile males identified by their apparent tusks and these were identified as females. However, it should be noted that males in some populations tend to be tuskless for survival due to the intensive historical poaching pressure. The high proportion of makhnas in NNNR identified from this investigation could indicate that these juveniles that were identified as females might in fact be males." Please go to P20, L380-385.
Comment 59: L. 355: Why do you say even if it was 1:1.5?
Response: This mistake has been corrected. Please go to P16, L290-296.
Comment 60: L. 359: Ref. 72 is not about balance in sex ratio in Asian elephant, it is about a sexing method. Make sure it is appropriate to cite it.
Response: These sentences and reference 72 have all been deleted. Please go to P17, L323.
Comment 61: L. 360-361: In this sentence you refer to tuskless males, but who identified these tuskless males? If it is another study, cite it.
Response: We identified these tuskless males. Approaches were displayed in Table 1 section. Please go to Table 1.
Comment 62: L. 366: "116 individual deaths (…) might account for tuskless males". It is unclear here what you mean.
Response: High poaching pressure could lead to the predominance of tuskless males. Since currently there is a high proportion of tuskless males in this population, we had assumed that the reason for this situation was historical poaching. But since we don't have data to support this assumption, this sentence has been deleted. Please go to P20, L383-385.
Comment 63: L. 360-370: I find all this difficult to follow because of some problems of clarity, but above all, you discuss a point when you recognize that you do not know for sure if the tuskless individuals you identified were males or females!
Response: Yes. We judged these juveniles as females under the condition that they have similar body height as juvenile males who had apparent tusks. And at the same time, we discussed that since tuskless males are common in this population, it would be helpful to conduct genetic studies on this population that can confirm the sex of these two juveniles. Please go to P20, L380-387.
Comment 64: L. 379: Add at the end of that sentence that this is especially true due to birth intervals.
Response: The sentence has been changed to "In either case, the scarcity of fertile females would be the primary constraint on population growth due to their long birth intervals." P21, L394-395.
Comment 65: L. 398-399: OK, but the way you present it by comparing to India leaves the reader with the idea that these authors (ref 79) did the study over a short period of time. Was this the case, if not, why discuss this and in that way? This is very troubling.
Response: It has been deleted. Please go to P19, L344.
Comment 66: L. 401: This value (800 m distance) makes sense only if we have an idea of elephant activity areas and movements.
Response: Here, we cited two references that pointed out that this value was smaller than elephant daily movement distance. Please go to P19, L346-348.
Comment 67: L. 404: Not real, but actual. It is still based on what you captured on photographs.
Response: Yes, the result should be actual value. The changes have been made in the new manuscript. Please go to P19, L350-351.
Comment 68: L. 405: estimated by whom? Because your sentences are not well

linked, it is hard to follow.
Response: We estimated it. We found the reference by Sukumar (2003) who gave an assumption that a population density of 0.1-0.3 elephant/km2 was normal. So, we used this assumption to estimate there should be 20 individuals in NNNR (71 km2). In the new manuscript, we deleted this hypothesis. Please go to P17, L329-331.
Comment 69: L. 408: About carrying capacity: well, you said before that 40 years ago the estimate was of 20 individuals, so at 12 it is obvious that you are below (even if we don't know if 20 was at carrying capacity or not).
Response: The use of the word "carrying capacity" made the sentence less rigorous. This word has been deleted. Please go to P17, L327-331.
Comment 70: L. 394-410: All this paragraph is convoluted. Why make weird assumptions (two sentences above) if you know that the population has already been that size.
Response: The whole paragraph has been rewritten. Thus, we combined the density section with population size section to make it simpler. Please go to the Population size and density part of the Discussion section. P18, L323-337.
Comment 71: L. 415-418: Rewrite these two sentences, and put what is a result in the corresponding section.
Response: These two sentences have been placed in the corresponding section. Please go to P16, L307-308.
Comment 72: L. 422-424: So what? Here it is not the males venturing into human areas, but rather humans venturing into elephant territories. It means that males are more tolerant to humans, and has nothing to do with the quest for better food as when they venture in crop areas. You need to thread your arguments better.
Response: Thank you for your recommendation. This part has been rewritten. Please see P21, L400-401.
Comment 73: L. 424-425: What you mention is well known, but you said that the other females were not lactating, so how does your argument hold?
Response: The only three adult females in this population were all considerd to be potentially productive, and the body health condition of all of them was considered to be average or even in poor health condition. Our data showed that the individual AE06 who was nursing an offspring had the lowest BCS in this population. Therefore, we mentioned additional nutritional stress from lactation for AE06 could have resulted in an even poorer body condition. Please go to P21, L404-409.
Comment 74: L. 426: What does "reasonable" mean here? Also, you cannot consider the median or the mean as a standard. You may have a very healthy or very unhealthy population and the mean (or the median) can vary quite a lot.
Response: This sentence has been deleted. We had classified the 11-point scale with three ranks: poor, medium and good. Please go to P13, L259-260.
Comment 75: L. 427-429: Yes, this is true, but this would skew evaluation towards lower BCS for adults, compared to juveniles.... and you found the opposite.
Response: Yes, that is correct. Maybe the adults have lower BCS than juveniles assuming that we had a large population like previous studies. The truth is the population in NNNR was extremely small, and the fact is that we found that the adults had higher BCS than the juveniles. These sentences have been deleted since we do not want to confuse readers. Please go to P21, L406.
Comment 76: L. 431-438: All this is very speculative, and not very convincing. If the animals raid cultures during the rain season it might be for different reasons, and if they don't during the dry season maybe is it because they find enough food within the reserve. You have to be very careful: you do not have enough information to really discuss all that without speculating.
Response: We strongly agree with your comments. Please go to P22, L410-422.
Comment 77: L. 438-441: Yes, but you have nothing on that: your data is from one season, in one particular year... not much to inform conservation managers.
Response: This sentence has been deleted. Please go to P22, L419-422.
Comment 78: L. 445-446: Take out the conflict part. It is not part of this argument and just distract the reader.
Response: This conflict part has been deleted. Please go to P22, L423-427.
Comment 79: L. 448: "obesity level". Surely you want to say something else! In nature animals are not obese (or very rarely), only in zoos.
Response: We wanted to say "health condition". Please go to P23, L432.
Comment 80: L. 442-451: All this is well beyond what your data can tell.
Response: We aimed to extend our research to other elephant populations. Although more and more studies have been conducted in China, the study of the BCS has been

	neglected. Thus, we wanted to draw attention on this kind research for Chinese researchers and conservationists. Please go to P22-23, L423-435.<br>Comment 81: L. 454: You keep mentioning the question of evenness in the sex ratio, but there is no foundation in your manuscript to justify why an even structure would be better.<br>Response: We have deleted this part. Please go to P23, L436.<br>Comment 82: L. 472-474: About reintroduction. Your study does not confirm that reintroduction is the solution for this population!!! In no way. See my previous comment on this in the introduction.<br>Response: All the information related to reintroduction has been deleted. Please go to comment 11 and P6, L112-115 or Conservation Recommendation section.<br>Comment 83: L. 478: Why mention restoration here? You have never mentioned that the habitat was either destroyed or degraded within the reserve. If it is the case, you should have better described your study area.<br>Response: The word "restoration" has been deleted. Please go to Conservation Recommendation section on P436.<br>Comment 84: L. 485-486: About reintroduction to reduce inbreeding. You have no data on that, do not speculate, especially when recommending such drastic approach as translocation, which comes with lots of cons.<br>Response: All the information related to reintroduction has been deleted. Please go to comment 11 and P6, L112-115 or Conservation Recommendation section.<br>Comment 85: L. 489: Please restrain from mentioning reintroduction. Just managing the population to ensure its viability (if it is possible), would be enough! As I mentioned in the comments to the introduction, reintroduction is rarely a good solution, as the problem is usually with the habitat (quality or quantity) or with the negative interactions with humans.<br>Response: All the information related to reintroduction has been deleted. Please go to comment 11 and P6, L112-115 or Conservation Recommendation section.<br>Comment 86: Figure 3: 1) Eliminate "points" (after Presence, Absence, and Village). 2) The border with Myanmar and the limits of the reserve are similar (same width, same colour). 3) It is har to say where the experimental zone is on the map. It would be better to use the light shade to shade the area.<br>Response: All the suggestions that you proposed have been taken into consideration. See more details in file named "Fig 1".
**Additional Information:**	
**Question**	**Response**
**Financial Disclosure**<br><br>Enter a financial disclosure statement that describes the sources of funding for the work included in this submission. Review the submission guidelines for detailed requirements. View published research articles from *PLOS ONE* for specific examples.<br><br>This statement is required for submission and **will appear in the published article** if the submission is accepted. Please make sure it is accurate.	Funding for this work was provided by National Forest and Grassland Administration, http://http://www.forestry.gov.cn, China under the Project of Asian Elephant Habitat Assessment and Habitat Maintenance Pilot, with effective support by the Yunnan Forest and Grassland Administration. The funders had no role in study design, data collection and analysis, decision to publish, or preparation of the manuscript.

**Unfunded studies**<br>Enter: *The author(s) received no specific funding for this work.*<br><br>**Funded studies**<br>Enter a statement with the following details:<br>• Initials of the authors who received each award<br>• Grant numbers awarded to each author<br>• The full name of each funder<br>• URL of each funder website<br>• Did the sponsors or funders play any role in the study design, data collection and analysis, decision to publish, or preparation of the manuscript?<br>• **NO** - Include this sentence at the end of your statement: *The funders had no role in study design, data collection and analysis, decision to publish, or preparation of the manuscript.*<br>• **YES** - Specify the role(s) played.<br><br>* typeset	
**Competing Interests**<br><br>Use the instructions below to enter a competing interest statement for this submission. On behalf of all authors, disclose any competing interests that could be perceived to bias this work—acknowledging all financial support and any other relevant financial or non-financial competing interests.<br><br>This statement **will appear in the published article** if the submission is accepted. Please make sure it is accurate. View published research articles from *PLOS ONE* for specific examples.	The authors have declared that no competing interests exist.

**NO authors have competing interests**<br><br>Enter: *The authors have declared that no competing interests exist.*<br><br>**Authors with competing interests**<br><br>Enter competing interest details beginning with this statement:<br><br>*I have read the journal's policy and the authors of this manuscript have the following competing interests: [insert competing interests here]*<br><br>* typeset	
**Ethics Statement**<br><br>Enter an ethics statement for this submission. This statement is required if the study involved:<br><br>• Human participants<br>• Human specimens or tissue<br>• Vertebrate animals or cephalopods<br>• Vertebrate embryos or tissues<br>• Field research<br><br>Write "N/A" if the submission does not require an ethics statement.<br><br>General guidance is provided below. Consult the submission guidelines for detailed instructions. **Make sure that all information entered here is included in the Methods section of the manuscript.**	Both National Forestry and Grassland Administration of China and Yunnan Forestry and Grassland Administration reviewed all sampling procedures and approved permits for the work conducted in NNNR. Non-invasive methods were applied and approval from an Institutional Animal Care and Use Committee or equivalent animal ethics committee was therefore not required.

Powered by Editorial Manager® and ProduXion Manager® from Aries Systems Corporation

**Format for specific study types**<br><br>**Human Subject Research (involving human participants and/or tissue)**<br>• Give the name of the institutional review board or ethics committee that approved the study<br>• Include the approval number and/or a statement indicating approval of this research<br>• Indicate the form of consent obtained (written/oral) or the reason that consent was not obtained (e.g. the data were analyzed anonymously)<br><br>**Animal Research (involving vertebrate animals, embryos or tissues)**<br>• Provide the name of the Institutional Animal Care and Use Committee (IACUC) or other relevant ethics board that reviewed the study protocol, and indicate whether they approved this research or granted a formal waiver of ethical approval<br>• Include an approval number if one was obtained<br>• If the study involved *non-human primates*, add *additional details* about animal welfare and steps taken to ameliorate suffering<br>• If anesthesia, euthanasia, or any kind of animal sacrifice is part of the study, include briefly which substances and/or methods were applied<br><br>**Field Research**<br><br>Include the following details if this study involves the collection of plant, animal, or other materials from a natural setting:<br>• Field permit number<br>• Name of the institution or relevant body that granted permission	
**Data Availability**<br><br>Authors are required to make all data underlying the findings described fully available, without restriction, and from the time of publication. PLOS allows rare exceptions to address legal and ethical concerns. See the PLOS Data Policy and FAQ for detailed information.	Yes - all data are fully available without restriction

A Data Availability Statement describing where the data can be found is required at submission. Your answers to this question constitute the Data Availability Statement and **will be published in the article**, if accepted.<br><br>**Important:** Stating 'data available on request from the author' is not sufficient. If your data are only available upon request, select 'No' for the first question and explain your exceptional situation in the text box.<br><br>Do the authors confirm that all data underlying the findings described in their manuscript are fully available without restriction?	
**Describe where the data may be found in full sentences. If you are copying our sample text, replace any instances of XXX with the appropriate details.**<br><br>• If the data are **held or will be held in a public repository**, include URLs, accession numbers or DOIs. If this information will only be available after acceptance, indicate this by ticking the box below. For example: *All XXX files are available from the XXX database (accession number(s) XXX, XXX.*).<br>• If the data are all contained **within the manuscript and/or Supporting Information files**, enter the following: *All relevant data are within the manuscript and its Supporting Information files.*<br>• If neither of these applies but you are able to provide **details of access elsewhere**, with or without limitations, please do so. For example:<br><br>*Data cannot be shared publicly because of [XXX]. Data are available from the XXX Institutional Data Access / Ethics Committee (contact via XXX) for researchers who meet the criteria for access to confidential data.*<br><br>*The data underlying the results presented in the study are available from (include the name of the third party*	All relevant data are within the manuscript and its Supporting Information files.

and contact information or URL).<br>• This text is appropriate if the data are owned by a third party and authors do not have permission to share the data.<br><br>* typeset	
Additional data availability information:	

Manuscript Click here to access/download;Manuscript;Manuscript.docx

Population structure and body condition assessment to inform conservation strategies for a small isolated Asian elephant population in southwest China

Yakuan Sun[1¶], Ying Chen[2¶], Juan José Díaz-Sacco[1¶], Kun Shi [1,3¶*]

[1]School of Ecology and Nature Conservation, Beijing Forestry University, Beijing, China

[2]School of Biological Science, The University of Hong Kong, Hong Kong, China

[3]Eco-Bridge Continental, Beijing, China

[*]Corresponding author

E-mail: kunshi@bjfu.edu.cn (KS)

[¶]These authors contributed equally to this work.

Abstract

The Asian elephant (*Elephas maximus*) population in Nangunhe National Nature Reserve represents a unique evolutionary branch in China, which has been isolated for more than twenty years from other neighboring populations in Myanmar. The scarcity of information on population structure, sex ratio and body condition make it difficult to develop effective conservation measures for this elephant population. Twelve individuals were identified from 3,860 valid elephant images obtained from February to June of 2018 (5,942 sampling effort nights) at 52 camera sites. Three adult females, three adult males, one subadult male, two juvenile females, two juvenile males and one calf male were identified. The ratio of adult females to adult males was 1:1, and the ratio of reproductive ability was 1:0.67, indicating the scarcity of reproductive females as an important limiting factor to population growth. A population density of 5.32 ± 1.56 elephants/100 km^2 was estimated ~~by~~ using Spatially Explicit Capture Recapture (SECR) models. The health condition of this elephant population was assessed by using an 11-points scale of Body Condition Scoring (BCS) ~~method~~ which showed that this population had a medium body condition with an average of 5.75 (n=12, range 2-9). This study finds that this isolated population has an extremely small size and an inverted pyramid age structure and therefore the population is at high risk of extinction. As such, this population should be of top conservation priority among the few remnant populations in China. The database established in this study can provide researchers with valuable information to set up efficient conservation schemes.

Introduction

The Asian elephant (*Elephas maximus*), as the largest terrestrial mammal in Asia, is considered an ecosystem engineer because it can modify its habitat and aid in seed dispersal [1, 2]. The conservation of this umbrella species contributes to the preservation of ~~most of the~~ biodiversity in the ecosystems it uses, especially in tropical moist and in tropical dry broadleaf forests of Southeast and South Asia [3-5]. It is also considered a flagship species that can attract interest for the protection of biodiversity [6]. It is a Class I Key Protected Species in China and listed as Endangered under the IUCN Red List [7, 8]. China sustains a population of c. 300 wild Asian elephants which are divided into seven geographic populations [9, 10]. Most Asian elephant populations are located in Mengyang, Mengla and Shangyong areas of Xishuangbanna National Nature Reserve, while there are also a few populations in Simao, Jiangcheng and Lancang counties of Pu'er city and one small isolated population in the Nangunhe National Nature Reserve (NNNR) of Lincang city [9, 11].

The conventional methods used for tracking Asian elephants in ~~the~~ dense forest are difficult to accomplish and usually fail to provide accurate population numbers and age structure [12, 13]. Although it is relatively easy to detect elephant signs, such as dungs or footprints, by using sign surveys [14], it can be difficult to identify individual Asian elephants since signs are highly inaccurate and unreliable [15]. Individuals can be identified using non-invasive DNA methods, but it requires relatively fresh samples and the lab costs can be quite high; moreover, the population size can only be determined with genetic capture-mark-recapture methods, which can be time and labor intensive [16, 17]. Furthermore, since elephant calves have lower defecation rates, this

method can underestimate the number of young individuals in a population [18].

Infrared trigger camera technology can make up for the above-mentioned disadvantages [19, 20]. Infrared cameras cause minimal disturbance to the study animal and its habitat, can be used for long working hours, can withstand harsh weather conditions, and are relatively cheap [19, 21, 22]. Infrared cameras are widely used to obtain ecological baseline surveys of protected species [23, 24], to monitor endangered wildlife [25], to study habitat utilization [26], to observe animal behavior [27], and to study the mechanisms of coexistence in sympatric species [28, 29]. Moreover, infrared cameras can also be used to estimate population size and density by using Spatially Explicit Capture-Recapture models (SECR) [30-33]. This technology has been used extensively to estimate the population density of multiple species, especially those ~~which~~ possess easily observed markings, such as the snow leopard (*Panthera uncia*), the leopard cat (*Prionailurus bengalensis*), the Sumatran tiger (*Panthera tigris* ssp. Sumatrae), and the serval (*Leptailurus serval*) [33-36]. For species without natural markings, identification can also be conducted according to morphology, such as with the Asian elephant, the giant panda (*Ailuropoda melanoleuca*), and the fox squirrel （*Sciurus niger*) [37-39].

Population structure and density estimation are essential features that can be used to study the ecology of wildlife populations [40, 41]. The Body Condition Scoring (BCS) is an index of ~~an~~ animal’s health that reflects the quality of the environment in which the animal lives [42]. It can be used in conjunction with population structure and density estimation to obtain a more accurate picture of the health of a population. Furthermore,

it can be used for both longitudinal and cross-sectional studies, is comparatively inexpensive, and does not require a high technical capacity [43]. While BCS is individual-based, it is most meaningful when applied to a population, as an early indicator of the impact of management actions on the average health condition of a population [43, 44]. For Asian elephants, the BCS obtained through photographs can be as accurate as ultrasonic subcutaneous fat measurement, thus providing a safer and non-invasive method to evaluate the health condition of wild populations [43, 45].

Throughout the Asian elephant range, there have been a number of studies that have identified individual elephants by directly photographing them or by obtaining images from infrared cameras [17, 37, 46, 47]. In China, population studies have mainly been concentrated in Xishuangbanna National Nature Reserve [17, 46], with a few conducted in NNNR, focusing on population size estimation, but not on the density or the health of its populations.

Studies conducted on the elephant population in NNNR concluded that it was a separate evolutionary unit isolated from other geographic populations [13, 48], thus lacking genetic connectivity. Although other elephant populations can be found in nearby Myanmar, the forest degradation in the China-Myanmar border area has led to the isolation of this elephant population since 1997 [9, 49, 50]. Additionally, this population is facing threats from habitat loss, fragmentation and illegal hunting [49, 51, 52]. Although conservation interventions have been proposed to rescue this population from extinction, the lack of scientific data and information on population structure, relatedness, and health status prevent the development of efficient conservation

strategies.

Isolated populations are vulnerable to changes in the external environment and thus, their conservation should be a top priority [53, 54]. However, previous studies on Asian elephants in China were limited to studying population sizes and did not assess the health condition of the population [13, 52]. An accurate understanding of the current situation of the local elephant population in NNNR, especially its structure and health condition, is key to establishing a baseline for the development of more effective protection and management schemes.

In this study, our aim was to determine the size, density, demographic structure and health condition of the NNNR Asian elephant population by using infrared camera trapping. We sought to understand which factors might have affected its population size in recent years in order to develop targeted and effective protection strategies. Furthermore, our approaches can be applied to other elephant populations with further implications for global Asian elephant conservation.

Material and Methods

Ethics Statement

Both National Forestry and Grassland Administration of China and Yunnan Forestry and Grassland Administration reviewed all sampling procedures and approved permits for the work conducted in NNNR. Non-invasive methods were applied and approval from an Institutional Animal Care and Use Committee or equivalent animal ethics committee was therefore not required.

Study area

The study area is located in the western part of the Nangunhe National Nature Reserve, Yunnan, where elephants are found [49, 51] (Fig 1). It occupies 70.82 km^2 at latitude 23°13'- 23°19'N, longitude 98°54'- 99°05'E, and the altitude ranges from 510 m to 1747 m. Rainfall is highly seasonal, with a dry season between November and April, and a rainy season between May and October. Mean annual precipitation is 351.3 mm in the dry season and 1983.3 mm in the rainy season, while temperature varies at different altitudes. The average monthly temperature range is hottest in July, varying between 12.3-25.5 ℃ and it is coldest in January with values between 2.8-14.3 ℃ [55]. Vegetation and climate vary across elevation zones. Tropical forest and tropical monsoon forest are located at altitudes below 600 m, middle elevation forests are dominated by subtropical monsoon evergreen broad-leaf forests, and evergreen broadleaf forests are found above 1700 m [51]. Bamboo forest, shrub and grasslands are scattered throughout this area. Deforestation along the China and Myanmar border has disrupted the connectivity between elephant populations that are found in both countries [49]. Furthermore, an iron fence in the southern part of reserve is used to protect the surrounding villages from wildlife (Fig 1).

Fig 1. Map of study area in NNNR showing the camera sites with ~~elephants~~ or without elephants.

Camera trap set-up

We carried out the survey for four months between February and May 2018 using

automatically triggered camera traps (Ltl Acorn 6210). There was a total of 52 cameras stations which successfully recorded 43-129 camera trap nights in the field (Fig 1). The total camera trapping effort was 5,942 trap nights (114.27±19.83). All the stations were located in evergreen broadleaf forest, broadleaf and bamboo forest, bamboo forest, pine forest and nearby ~~the~~ brush forest, which represente~~d~~ the preferred habitat of elephants in NNNR. We systematically deployed camera traps along the animal trails and forest gaps where there were signs of elephants, including lying sites, foraging sites, footprints, dungs and urine marks. ~~Cameras had~~ attempted to ~~be~~ deploy~~ed~~ throughout the reserve ~~in~~ a 1 km^2 grid ~~formation~~. We increased the number of cameras in the grids with many elephant signs, ~~but~~ always with a minimum of 200 m between any two cameras. We avoided the northwestern part of the reserve as the topography is tough, with steep cliffs of up to 1747 meters, and historically~~,~~ there have not been any records of elephants in this area [56, 57].

Cameras were strapped onto trees at approximately 1.5 m above the ground with the sensor parallel to the ground to maximize the extension of the detection zone. The positions of all the camera sites were recorded by GPS. To avoid blank images caused by sunlight, the cameras were never placed facing east. Camera traps were hidden as much as possible to reduce the disturbance to wildlife and the probability of being stolen. Camera traps were set to operate 24 hours per day and programmed to take three photographs and one 12 second video as a sequential without delay, registering the date and time for each exposure. At each site, a camera trap was operated continuously for a maximum of four months. SD cards and batteries were replaced every month. We

recorded the time of installation and retrieval of each camera and calculated the total duration of sampling.

Individual identification

The images obtained from the camera traps were categorized to independent events, which were defined as a “sequence”. Each sequence is defined by a set of images adjacent in time. For an elephant unit (defined as all or the maximum number of individuals in a group), the sequence includes the images from the first one individual entering the field to the last one exiting it, making it possible to identify different individuals based on distinct features. For solitary individuals, the sequence is a series of captured images of same individual within continuous time period [58]. Only images with sufficient quality, clarity and position of the elephant in the frame were used for individual identification [58]. Here, quality referred to how usable an image was for age and sex classification; clarity indicated whether the elephant was “captured” in good light or that the flash reached the individual, and the image was in focus and not over or under-exposed; and position referred to the occurrence of the elephant within an ‘optimal distance and angle’ from the camera [47, 58]. The age of each animal was broadly classified into adult, subadult, juvenile and calf using approximate shoulder height as recommended by previous studies, and gender was mainly determined by secondary sexual characteristics [59-61] (Table 1). We defined the age structure as the ratio of adults, subadults, juveniles and calves while the reproductive ability was the ratio of age structure between adult and juvenile females [47], due to the absence of subadult and calf females in this reserve. Male elephants living completely or mostly

outside the herd were classified as solitary males [12]. Based on individual events, a capture-history event database was established to record the individuals and their characteristics. Morphological characteristics including facial bones, ear, tusk, back and tail hair, old scars and injuries, as well as physical characteristics related to sex and age classes, confirmed the reliability of identification results [17, 37, 46, 62] (Table 1).

Table 1. Morphological characteristics used for individual identification of elephants photographed in NNNR, between February and May 2018.

Characteristics	Categories	Description
Sex	Adult female	Without obvious tusks but with prominent breasts, belly swelling or the presence of nursing offspring
	Adult male	With obvious tusks or visible penis but without prominent breasts, the presence of penis for tuskless individual
	Subadult male	Without obvious tusks but with visible penis
	Juvenile female	Without obvious tusks
	Juvenile male	With obvious tusks
	Calf male	~~Only the~~ testicles or penis ~~were visible~~
Age	Adult (>15years)	With the highest shoulder height
	Subadult (5-15years)	Shoulder height reaches an adult's eyes
	Juvenile (1-5years)	Shoulder height reaches half the height of an adult
	Calf (0-1year)	Below an adult's knee
Facial bones	Wisdom tumor	Size
	Bones between the eyes	Extent of prominence
	Upper margin of eyes	Extent of depression
Tusks	Length	Very short/short/long compared with trunk
	Thickness	Thick/normal/slender
	Shape	Straight/bent
	Crack	Yes/no
Ear	Upper edge	Fold/unfold
	Lobe	V-acute/U-rounded
	Lobe tear	Yes/no
	Ear hole	Yes/no
Tail brush	Standard	Present/both sides/both-continuous

	Nonstandard	Absent/single side/both-discontinuous

Density estimation

The elephants in NNNR belong to a small isolated population without any migrants, thus conforming to the "close population" assumption of SECR [63]. The model accounts for the movement in density estimation by combining a state model and an observation model, which describe how the observed data were generated [63]. The state model describes statistically the mechanisms determining the distribution of the animals in the study area, while the observation model quantifies the probabilities of detection or capture, given the searched region, their locations and characteristics at different sessions [64]. Basically, SECR are generalized linear mixed models that assume that ev[illegible] individual *i* in the population has its own unobserved activity center S_i, and all the activity centers $S_i \cdots S_N$ are distributed across the study area [65, 66].

The encounter rate of an individual *i* with a given trap *j* located at X_j is a monotonically decreasing function of the distance from S_i to *j*. Y_{ij}, the number of times individual *i* is detected by trap *j* during a sampling occasion, is a random variable following a Poisson distribution and with mean λ_{ij} [67]:

$$Y_{ij} \sim \text{Poisson}(\lambda_{ij})$$

For an elephant *i* captured by a trap *j*, the model assumes a log-linear form [68]:

$$\text{Log}(\lambda_{ij}) = \text{Log}(\lambda_0) - \left(\frac{1}{2\sigma^2}\right) \|S_i - X_j\|$$

Here, λ_0 is the baseline encounter probability, the function $\|S_i - X_j\|$ is the distance between the activity center and the trapping station, and σ is the Gaussian scale

parameter for the distance function between activity centers and trap locations; assuming a bivariate normal model of space use, sigma can be used to calculate a 95% activity area radius.

SECR models estimate the density of animal activity centers in an area large enough so that animals residing beyond it have a negligible chance of being detected [63]. Considering the small size of the elephant distribution, with the east and west boundary being no wider than 10 km, we therefore defined our study area as extending 5 km beyond all camera stations, corresponding to an area of 255.8 km^2 [69].

SECR 3.1.5 package in R 3.6.1 was used for density estimation. Data were prepared as an R object of class “capthist”. This object included both the capture data and the detector layout. The file named “capture histories” contained data on individual identification, the occasion (each sample time, tha[illegible] event here), the detector (camera ID). The detector file contained data on cameras ID, and x, y coordinates. Five different sessions, corresponding to each of the months and one corresponding to the whole survey period, were used to run the model to see the estimation accuracy under different detection efforts.

Body condition scoring

We established a database of each identified individual and the images with the best angle of each individual elephant were selected to score the body condition. Compared with previous studies, our target population size was small, and the sample size was limited so we used a simple method which can rapidly and more easily be applied to wild elephant populations. In this study, we applied a BCS method that

focuses on six physical characteristics and ranks the elephant´s body condition according to an 11-points scale [42, 43]. Five of the clearest images for each physical characteristic were selected to assess the body condition. Thirty images were used to calculate the comprehensive scores for each individual. This scoring method is based on the extent of visibility of depressions around bone structures. Depressions around bones become visible as an animal loses its subcutaneous fat deposits and muscles in the region concerned, thereby making bones appear more prominent. Skull, pectoral girdles in shoulders, vertebral column, ribs and pelvic girdles are prominent bone structures. Thick rolls of skin that fold below the neck can accumulate fat, and thus, also represent a criteria of good body condition. We presented a scale of 5 reference scored elephants description of 1, 3, 5, 7, 9, with numerically higher values corresponding to better body condition (Table 2). However, the range of scores extended from 0 to 10 due to the possibility of greater variation. For example, if all the ribs of an elephant were visible, it meant that this individual presented a worse body condition than 1 and thus, it would be assigned 0. If an individual presented a fatter condition than 9 and had very thick rolls of skin fold below neck, it would be assigned a score of 10. Scores ranging from 0-3 were considered as poor, 4-7 were considered medium and 8-10 were considered as good [44]. Pearson correlation coefficient (r) was used to study the relationship between BCS and various covariates such as sex, age, and whether an individual was solitary or in a herd.

Table 2. Diagnostic characteristics pertaining to scores in photographic scale.

**Score**	**Characteristics**

1	Ribs from shoulder to pelvis are visible, some ribs prominent (spaces in between sunken in)
3	Some ribs visible (spaces in between not sunken in), shoulder and pelvic girdles prominent
5	Ribs not visible, shoulder and pelvic girdles visible
7	Backbone visible as a ridge, shoulder and pelvic girdles not visible
9	Backbone not visible or difficult to differentiate and pelvic bones not visible

Results

Population size and density

A total of 70,902 images were obtained, among which 7.34% (5,207) were Asian elephant data. There was a total of 134 independent events, including 40 events of elephants in a herd and 94 events of solitary elephants. Excluding the image data that could not be used for individual identification, there were 3,860 valid Asian elephant images belonging to 85 independent events (26 events of individuals in a herd and 59 events of individuals). Generally, all herds and individuals had similar frequencies of recapture histories during the study period, particularly herds. However, solitary elephants were image-captured with a minor difference: a minimum of 16 capture times ~~of~~ AE12 compared to a maximum of 25 capture times ~~of~~ AE10 (Table 3).

Table 3. Number of capture events ~~for a given~~ individual per month.

Individual No.	Age	Gender	Tusk	Type	Capture events				
					Feb.	Mar.	Apr.	May	Total
AE01	Juvenile	Male	Yes	Herd	2	6	5	8	21
AE02	Adult	Female	No	Herd	3	5	6	6	20
AE03	Adult	Female	No	Herd	3	5	6	7	21
AE04	Juvenile	Female	No	Herd	2	6	5	7	20
AE05	Calf	Male	No	Herd	3	6	8	5	22
AE06	Adult	Female	No	Herd	4	6	8	5	23
AE07	Juvenile	Female	No	Herd	4	6	9	6	25
AE08	Juvenile	Male	Yes	Herd	4	7	6	7	24
AE09	Sub-adult	Male	No	Solitary	3	9	2	9	23
AE10	Adult	Male	Yes	Solitary	2	3	13	7	25

AE11	Adult	Male	No	Solitary	3	5	8	5	21
AE12	Adult	Male	No	Solitary	2	6	1	7	16
Total	-	-	-	-	35	70	77	79	261

A total of 12 individuals were identified among these valid independent events (Fig 2; S1 Dataset). The elephant population size in each session was consistent, except in February. Moreover, the population size estimated by SECR was 12.00 ± 0.71 (with a 95% confidence interval of 12.00-12.11; Table 4). The estimated density by SECR was 5.32 ± 1.56 elephants/100km^2 (with a 95% confidence interval of 3.02-9.36). In camera-trapping session at monthly intervals, April had the lowest density estimation at 6.07 ± 1.79 elephants/100km^2, while May had the highest density estimation at 24.43 ± 7.20 elephants/100km^2. The movement parameter σ was twice as large in April (6643 m ± 2356 m) than in May (3771 m ± 1471 m).

Fig 2. Asian elephants individually identified in NNNR, China. Each individual's most distinguished characteristic was emphasized, except easily identified AE05 and AE10, the only calf and adult male with tusks.

Table 4. Population density estimation by using SECR models under five sessions.

Sessions	Occasions	Density			λ_0		sigma		Population size		
		estimate	SE.estimate	95% CI	estimate	SE.estimate	estimate	SE.estimate	estimate	SE.estimate	95% CI
February	11	10.71	3.31	5.91-19.38	0.15	0.04	2983	664	14.00	2.19	12.35-23.43
March	26	6.57	1.94	3.73-11.58	0.07	0.02	4193	700	12.20	0.87	12.00-17.95
April	22	6.07	1.79	3.45-10.70	0.02	0.004	6643	2356	12.05	0.75	12.00-16.87
May	26	24.43	7.20	13.87-43.03	0.01	0.003	3711	1471	12.03	0.74	12.00-16.48
Total	85	5.32	1.56	3.02-9.36	0.02	0.003	6474	1065	12.00	0.71	12.00-12.11

Demographic structure

We identified an inverted pyramid age structure for this population: three adult females, three adult males, one subadult male, two juvenile females, two juvenile males and one male calf, with a sex ratio of 1:1.4 using females as a reference (Table 4). The three adult males comprised one tusker and two without tusks (Makhnas), giving an adult tusked to tuskless male ratio of 1:2. The subadult male was also a makhna, increasing the tusked to tuskless male ratio to 1:3. The ratio of reproductive ability was 1:0.67.

The social network judging from camera trap data is AE01-AE08 formed the joint-family unit, while individual calf AE05, adult female AE06 and juvenile female AE07 as a mother-calf unit which occasionally joined and departed from the large family unit. AE06 and AE05 were in a mother-child relationship since there were images of the calf nursing from the female. The solitary subadult male AE09 and adult male AE10 were observed to occasionally join the family unit.

Body condition scoring

The distribution of BCS for the population had an average of 5.75 (n=12, range 2-9; Fig 3). Body condition evaluation revealed that 33.3% of the elephants were in a good health condition, while 50% and 16.7% were in medium, and poor health conditions respectively (Fig 3). Age was not significantly correlated to BCS (r=0.03), but sex was (r=0.42), as adult males had better body conditions than adult females. The body condition of the only three fertile females in this population was not good. The

adult female AE02 had a BCS just above the average score, while the other females, AE03 and AE06 were in poor health conditions with a low BCS of 3. Being solitary or in the herd played more important roles in the BCS (r=0.53) than sex. Individuals that were part of the herd had a lower BCS than solitary individuals.

Fig 3. Body condition score of the 12 individuals identified in NNNR.

Discussion

Population size and density

Accurate assessment of population size is essential for species conservation and management [70]. Identification from captured images was robust enough to identify individuals [71], unlike dung and footprints which are easily influenced by slope, soil quality and climatic factors [72]. This study used cameras installed in the elephant preferred habitats that covered most of the western part of the reserve, which provided a higher chance of obtaining the data reflecting the real population, leading to a more reliable population assessment ~~as~~ in the study that used drone surveys [52]. Historical overview of the research on this population estimated by several different methods may generate inevitably a bias in direct comparison, the NNNR population showed a population size fluctuating around 20 individuals, and a tendency towards a slight population decrease [52, 72-74] (S1 Table). Nowadays, ~~the distribution for~~ this population still remains in the western part of the NNNR Cangyuan county [52, 73, 75] (Fig 1). Therefore, we assumed that there is space available in NNNR to allow for this small population to reach its highest ever recorded population size of 24-25 individuals [56, 73]. The NNNR population is an extremely small and isolated population,

compared with other elephant populations found around the world [3, 9, 10, 12]. Historical illegal hunting has had a direct impact on this population with a total of 15 unnatural deaths since 1987 (Table 5). Additionally, the high mortality of immature elephants (75%) among these unnatural deaths since 2003, as well as a long adolescence and long inter-birth intervals [12, 76], have had potentially negative effects on this populations growth.

Table 5. Unnatural deaths of Asian elephant since the establishment of NNNR.

Year	Number of individuals	Cause of death	Data resources
1987	Two: One female and one male	Illegal killing	[55, 77]
1988	Four: Two adult males, one adult female and one calf	Poaching, electrocuting and unknown reason	[49]
1995	One male	Unknown	[52]
1996	One male	Vengefully hunted by local people	[51]
1997	Three: One female, one male and a calf	Unknown for the calf, the female and male were killed	[55]
2003	A Subadult male (about 10 years old)	Unknown	[55]
2008	One subadult male	Unknown	Communication with local residents and rangers
2011	One juvenile	Unknown	[55]
2016	One adult male	Unknown, but the tusks show visible saw marks	Communication with local residents and rangers

SECR modelling has become a powerful tool to estimate the small population sizes like the one in NNNR [32]. The model defines the activity center according to the range of the species activities [78], so it is easy to overestimate the population density in a short survey period, which was confirmed by monthly estimates in this study (Table 4). We first used the single monthly session to run the SECR model, with which we

obtained a significant difference in density estimation. The SECR model simulated a larger movement buffer (sigma=6643 m) in April, which resulted in the smallest population density. May presented the highest density because there were 8 cameras that captured elephants, and the distance between each one was less than 800 m, which is smaller than its daily movement distance [79, 80]. The instability of single month session results by SECR, prompted us to use the four months data as the fifth session to run the model. We thus obtained the smallest standard deviation, which indicated that the result was closer to the actual value [47].

The population density results of the NNNR elephant population, as an unique evolutionary clade in China, can be used to compare with results from other populations in order to improve our understanding of elephant ecology and develop more detailed management and conservation plans [81]. However, with no other SECR analyses conducted on other populations in China, we used the crude density estimation (estimated population number/study area) to draw comparison between the NNNR population and others in Yunnan [47]. The crude density estimation of Asian elephants in NNNR was the highest at 16.9 elephants/100 km^2 due to its smaller area of distribution, compared with that in Shangyong, Mengla, and Menyang with a density of 10.68, 4.26, and 1.85 elephants/100 km^2 respectively [13, 17]. This is concerning as a high population density could lead to more [illegible]ensive competition among individuals for limited natural resources [82], and even greater human-elephant conflict as elephants resort to crop raiding [83].

Population structure in NNNR

Although the juveniles in this population accounted for 33% of the population, higher than the minimum of 20% of the population considered to be needed for it to be self-sustaining in a short period of time [18], inbreeding is inevitable in the near future due to the small population size.

We attempted to identify the relationship between individuals in the family unit through images captured by camera trap, since the allomaternal care or mother-like behavior directed by females are prevalent in the Asian elephant social structure [84, 85]. We could not match adult females with their offspring in the groups relying on the observed behaviors, even though we captured intimate interaction between AE02 and AE08, as well as between AE03 and AE04. In recent years, the death of adult females has not been recorded in the reserve (Table 5). Therefore, we assumed that all the current juveniles came from these three adult females. Genetic studies are urgently needed to better understand the lineage and degree of inbreeding depression in the NNNR population.

There were two tuskless juveniles that had similar body heights as the juvenile males identified by their apparent tusks and these were identified as females. However, it should be noted that males in some populations tend to be tuskless for survival due to the intensive historical poaching pressure [12, 86]. The high proportion of makhnas in NNNR identified from this investigation could indicate that these juveniles that were identified as females might in fact be males. Since tuskless males are common in this population, it would be helpful to conduct genetic studies on this population that can confirm the sex of these two juveniles. Unbalanced sex ratio is not beneficial for

population growth [87]. Currently, only AE02, AE03 and AE06 are of reproductive age, but the latter was nursing a calf, leaving only two sexually active females in the reserve. The reproductive ratio of this small population was relatively high compared to the findings in Ruhuna National Park, Sri Lanka [83], and Salakpra Wildlife Sanctuary, Thailand [47], since females do not enter the breeding period until they are 10-14 years old and as such these two juvenile females in the NNNR population still needed 3-7 years to become mature [9, 12, 88]. In either case, the scarcity of fertile females would be the primary constraint on population growth due to their long birth intervals.

Health condition

We chose a simple but accurate body condition method to assess the health of this small population. Our results showed a higher body condition of adult males than adult females, which was also observed in another elephant population in Southern India [44]. This may be because adult males are known to have larger home ranges than adult females ~~and immature males~~ [80, 89], and are more tolerant to human disturbance [90]. ~~Which~~ means they explore areas near the reserve boundary ~~frequently~~, where intensive anthropogenic activities like hunting, ~~gathering~~, fishing and collecting have also been recorded by cameras. Solitary elephants could have also had a higher BCS than the individuals in the family units because at least one of the herds could have had an even more reduced area of movement due to the presence of a calf [89]. Furthermore, additional nutritional stress from lactation for AE06 could have resulted in an even poorer body condition [44]. Additionally, the other two adult females both presented a medium health condition, which would not be conducive for reproduction [42].

Most of the individuals (66.7%) had medium or poor body conditions during the survey period. Since the quantity and quality of food and availability of water are the primary factors determining the body condition of free-ranging elephants [42], both female and male individuals show an obvious thinner body condition during the dry season compared to the rainy season [91]. We did not have the opportunity to conduct camera trap surveys during the rainy season since many cameras failed to work in this season. Additionally, the rainy season coincides with the harvest period. In the past, especially in recent years, NNNR elephant-damage compensation data has shown that the elephants forage for corn and rice during the rainy season from the month of July to October (data from the compensation documentary of the reserve bureau). Therefore, we predict that this population is foraging off of crops to supplement its diet. Thus, future studies should focus on determining if crops could improve the body condition of elephants during the rainy season.

Moreover, the body condition assessment in this study could have implications for the research and conservation of other elephant populations. Since the vast majority of Asian elephant populations, especially in China, are found outside of reserves and near cropland, the health of these populations are likely heavily influenced by access to crops [92]. Massive amounts of data have been collected by camera traps and drones in these areas [93, 94], but none have been used to determine the health of the populations. We have proven that using images for BCS to be a simple and fast method for determing the health of free-ranging elephant populations. Therefore, future studies should consider analyzing the body condition of different regional Asian elephant populations

from these data sets to ~~be used as an~~ early indicator of the health of individuals and quality of food resources in their habitats [43]. This in turn can be used to guide conservationists and policymakers to evaluate the necessity for interventions ~~and define regions for~~ suitable habitat ~~restoration~~ [42].

Conservation recommendations

To maintain this extremely small isolated elephant population, the long-term placement of cameras in NNNR is needed to get valuable data for the study of demography, fecundity and health status [47, 58, 59, 91]. Molecular biological approaches are also needed in further studies to determine the kinship and genetic health of this population [41].

The results of this study have reconfirmed that ~~the enlargement of~~ suitable habitat is urgently needed to ensure an adequate supply of food ~~is available~~ for this isolated population during the dry season [13, 49, 51]. Attention should be paid to the low body condition in the dry season and a follow-up survey carried out in the rainy season ~~as soon as posible~~ to verify the health status of this elephant population throughout the year. Therefore, the short-term objective should be to ~~supplement~~ the food shortages the NNNR population during the dry season by establishing or improving the ~~vegetation~~ quality of the food~~-~~sources [95]. Fence dismantlement~~, as the mid-term goal,~~ should be prioritized because it has partially blocked the movement of elephants and hindered their ability to find new food resources (Fig 1). Allowing the animals greater movement could provide more opportunities for the elephants to explore new suitable areas with enough food, eventually improving ~~the~~ health condition regardless of the season.

Ecological corridors should be considered as a long-term objective to connect this isolated elephant population to other suitable habitats. Since the western part of this reserve (Fig 1. Red part of NNNR) has some suitable habitats[51], an ecological corridor could be created by planting bamboo forest or bamboo-broadleaf forests, allowing ~~this population to migrate~~ between the eastern and western part of the reserve. We also suggest that research on the evaluation of the environmental capacity and availability of food resources should be conducted to set up a scientific baseline. Due to the very small population size and the scarcity of reproductive females, all suggestions should be urgently accomplished before the juveniles reach reproductive age to avoid potential inbreeding.

Acknowledgments

This study was implemented by Beijing Forestry University and Beijing Eco-Bridge Continental. We thank the Second National Survey of Terrestrial Wildlife in China and National Forestry and Grassland Administration of China for their support of this study. Furthermore, the field work was strongly supported by the administrators of Nangunhe National Nature Reserve, for which we thank them. We also thank all the field staff, including Wang Jiahui, Liu Zhen, Liu Ying and all local rangers, especially Chen Deming, Li Yongxiang, Yang Shaobing, Wang Zhisheng, Li Chunlian and Li Zhimin, for their hard and invaluable work.

References

1. Campos-Arceiz A, Blake S. Megagardeners of the forest – the role of elephants in seed dispersal.

Acta Oecologica. 2011;37(6):542-553. https://doi.org/10.1016/j.actao.2011.01.014.

2. McConkey KR, Nathalang A, Brockelman WY, Saralamba C, Santon J, Matmoon U, et al. Different megafauna vary in their seed dispersal effectiveness of the megafaunal fruit Platymitra macrocarpa (Annonaceae). Plos One. 2018;13(7):e0198960. https://doi.org/10.1371/journal.pone.0198960. PMID: 30020929.

3. Riddle HS, Schulte BA, Desai AA, Meer Lvd. Elephants - A conservation overview. Journal of Threatened Taxa. 2010;2(1):653-661.

4. Aijun T, Lingjiang Y. Distribution and Protection of Asian elephant in China. Protection Forest Science and Technology. 2015;(5):89-91. https://doi.org/10.13601/j.issn.1005-5215.2015.05.035.

5. Platt SG, Bickford DP, Win MM, Rainwater TR. Water-filled Asian elephant tracks serve as breeding sites for anurans in Myanmar. Mammalia. 2019;83(3):287-289. https://doi.org/10.1515/mammalia-2017-0174.

6. Bowen-Jones E, Entwistle A. Identifying appropriate flagship species: the importance of culture and local contexts. Oryx. 2002;36(2):189-195. https://doi.org/10.1017/S0030605302000261.

7. Jiang Z, Jiang J, Wang Y, Zhang E, Zhang Y, Li L, et al. Red List of China's Vertebrates. Biodiversity Science. 2016;24(5):500-551. http://www.biodiversity-science.net/EN/10.17520/biods.2016076.

8. Choudhury A, Lahiri Choudhury DK, Desai A, Duckworth JW, Easa PS, Johnsingh AJT, et al. Elephas maximus. The IUCN Red List of Threatened Species. 2008:e.T7140A12828813. https://dx.doi.org/10.2305/IUCN.UK.2008.RLTS.T7140A12828813.en.

9. Li Z. Research on Asian elephant Conservation in China: Science Press; 2018.

10. Yu Z, Kun J. Distribution, Population, Habitat Status and Population Management of Asian Elephant. World Forestry Research. 2018;31(2):25-30.

https://doi.org/10.13348/j.cnki.sjlyyj.2018.0002.y.

11. Mingyong C. Asian elephant research of China: Science Press; 2006.
12. Sukumar R. A brief review of the status, distribution and biology of wild Asian elephants Elephas maximus. International Zoo Yearbook. 2006;40(1):1-8. https://doi.org/10.1111/j.1748-1090.2006.00001.x.
13. Zhang L, Dong L, Lin L, Feng LM, Yan F, Wang LX, et al. Asian Elephants in China: Estimating Population Size and Evaluating Habitat Suitability. Plos One. 2015;10(5):e0124834. https://doi.org/10.1371/journal.pone.0124834. PMID: 25992617.
14. Tian Y, Feng Y, Zhang C, Yu B, Tang X, Hu H. Effectiveness of line transects during wild animal surveys in mountain forests of South China. Biodiversity Science. 2015;23(1):109-115. http://www.biodiversity-science.net/EN/10.17520/biods.2014128.
15. Barnes RFW. The problem of precision and trend detection posed by small elephant populations in West Africa. African Journal of Ecology. 2002;40:179-185. https://doi.org/10.1046/j.1365-2028.2002.00376.x.
16. Mills LS, Citta JJ, Lair KP, Schwartz MK, Tallmon DA. Estimating Animal Abundance Using Noninvasive DNA Sampling: Promise and Pitfalls. Ecological Applications. 2000;10(1):283-294. https://doi.org/10.2307/2641002.
17. Zicheng Y, Ying C, Junsong L, Lifan W, You P, Zhiyong S, et al. Individual identification and population size assessment for Asian elephant based on camera-trapping techniques. Acta Theriologica Sinica. 2018;38(1):18-27. https://doi.org/10.16829/j.slxb.150109
18. L.S.EGGERT, J.A.EGGERT, D.S.WOODRUFF. Estimating population sizes for elusive animals: the forest elephants of Kakum National Park, Ghana. Molecular Ecology. 2003;12(6):1389-1402.

https://doi.org/10.1046/j.1365-294X.2003.01822.x. PMID: 12755869.

19. Liu X, Wu P, He X, Zhao X. Application and data mining of infra-red camera in the monitoring of species. Biodiversity Science. 2018;26(08):850-861. https://doi.org/10.17520/biods.2018053.

20. Xiao W, Hu L, Huang X, Xiao Z. Using capture-recapture models in wildlife camera-trapping monitoring and the study case. Biodiversity Science. 2019;27(3):257-265. https://doi.org/10.17520/biods.2018194

21. Zhang L, Cui S, Huang Y, Chen D, Qiao H, Li C, et al. Infrared camera traps in wildlife research and monitoring in China: issues and insights. Biodiversity Science. 2014;22(6):696-703. https://doi.org/10.3724/SP.J.1003.2014.14225.

22. Wearn OR, Glover-Kapfer P. Snap happy: camera traps are an effective sampling tool when compared with alternative methods. Royal Society Open Science. 2019;6(3):181748. https://doi.org/10.1098/rsos.181748. PMID: 31032031.

23. Jorge A A, Carlos E F S, Krisna G, Chris H, Johanna H, Emanuel M, et al. Community structure and diversity of tropical forest mammals: data from a global camera trap network. Philosophical Transactions of the Royal Society of London. 2011;366(1578):2703-2711. https://doi.org/10.1098/rstb.2011.0115. PMID: 21844049.

24. Chengcheng Z, Jun W, ALEXANDER JS, Zhigang D, Liji W, Wantao D, et al. Biodiversity Assessment of Mammal and Bird Species from Camera Trap Data in Yanchiwan National Nature Reserve, Gansu Province, China. Journal of Resources and Ecology. 2018;9(5):566-574. https://doi.org/10.5814/j.issn.1674-764x.2018.05.014.

25. Broto B, Mortelliti A. The status of research on the mammals of Sulawesi, Indonesia. Mammal Review. 2019;49(1):78-93. https://doi.org/10.1111/mam.12141.

26. Montalvo VH, Sáenz-Bola˜nos C, Luis D. Alfaro1 JCC, Guimarães-Rodrigues FH, Carrillo E, Sutherland C, et al. Seasonal use of waterholes and pathways by macrofauna in the dry forest of Costa Rica. Journal of Tropical Ecology. 2019;35(2):68-73. https://doi.org/10.1017/S0266467418000457.

27. Matsukawa A, Ahmad AH, Kohshima S. Social structure and reproduction of long-tailed porcupine (*Trichys fasciculata*). Mammalia. 2019;83(1):23-33. https://doi.org/10.1515/mammalia-2017-0104.

28. Sunarto S, Kelly MJ, Parakkasi K, Hutajulu MB. Cat coexistence in central Sumatra: ecological characteristics, spatial and temporal overlap, and implications for management. Journal of Zoology. 2015;296(2):104-115. https://doi.org/10.1111/jzo.12218.

29. Rich LN, Miller DAW, Muñoz DJ, Robinson HS, McNutt JW, Kelly MJ. Sampling design and analytical advances allow for simultaneous density estimation of seven sympatric carnivore species from camera trap data. Biological Conservation. 2019;233:12-20. https://doi.org/10.1016/j.biocon.2019.02.018.

30. BLANC L, MARBOUTIN E, GATTI S, GIMENEZ O. Abundance of rare and elusive species: Empirical investigation of closed versus spatially explicit capture-recapture models with lynx as a case study. The Journal of Wildlife Management. 2013;77(2):372-378. https://doi.org/10.1002/jwmg.453.

31. Efford MG, Fewster RM. Estimating population size by spatially explicit capture-recapture. Oikos. 2013;122(6):918-928. https://doi.org/10.1111/j.1600-0706.2012.20440.x.

32. Bashir T, Bhattacharya T, Poudyal K, Sathyakumar S, Qureshi Q. Estimating leopard cat Prionailurus bengalensis densities using photographic captures and recaptures. Wildlife Biology. 2013;19(4):462-472. https://doi.org/10.2981/12-098.

33. Alexander JS. Advancing research and conservation of snow leopards in the Qilianshan mountains of China: management and methodological implications [Doctoral dissertation]: Beijing Forestry University; 2015.

34. O'Brien TG, Kinnaird MF, Wibisono HT. Crouching tigers, hidden prey: Sumatran tiger and prey populations in a tropical forest landscape. Animal Conservation. 2003;6(2):131-139. https://doi.org/10.1017/S1367943003003172

35. Bohm T, Hofer H. Population numbers, density and activity patterns of servals in savannah patches of Odzala-Kokoua National Park, Republic of Congo. African Journal of Ecology. 2018;56(4):841-849. https://doi.org/10.1111/aje.12520.

36. Park H, Lim A, Choi T-Y, Baek S-Y, Song E-G, Park YC. Where to spot: individual identification of leopard cats (*Prionailurus bengalensis* euptilurus) in South Korea. Journal of Ecology and Environment. 2019;43. https://doi.org/10.1186/s41610-019-0138-z.

37. Goswami VR, Madhusudan MD, Karanth KU. Application of photographic capture-recapture modelling to estimate demographic parameters for male Asian elephants. Animal Conservation. 2007;10(3):391-399. https://doi.org/10.1111/j.1469-1795.2007.00124.x.

38. Tye CA, Greene DU, Giuliano WM, Mccleery RA. Using camera-trap photographs to identify individual fox squirrels (*Sciurus niger*) in the Southeastern United States. Wildlife Society Bulletin. 2015;39(3):645-650. https://doi.org/10.1002/wsb.573.

39. Zheng X, Owen MA, Nie Y, Hu Y, Swaisgood RR, L.Yan, et al. Individual identification of wild giant pandas from camera trap photos - a systematic and hierarchical approach. Journal of Zoology. 2016;300(4):247-256. https://doi.org/10.1111/jzo.12377.

40. Gupta M, Joshi A, Vidya TNC. Effects of social organization, trap arrangement and density,

sampling scale, and population density on bias in population size estimation using some common mark-recapture estimators. Plos One. 2017;12(3):e0173609. https://doi.org/10.1371/journal.pone.0173609. PMID: 28306735.

41. Hedges S, Johnson A, Ahlering M, Tyson M, Eggert LS. Accuracy, precision, and cost-effectiveness of conventional dung density and fecal DNA based survey methods to estimate Asian elephant (*Elephas maximus*) population size and structure. Biological Conservation. 2013;159:101-108. https://doi.org/10.1016/j.biocon.2012.12.010.

42. Wijeyamohan S, Treiber K, Schmitt D, Santiapillai C. A Visual System for Scoring Body Condition of Asian Elephants (*Elephas maximus*). Zoo Biology. 2015;34(1):53-59. https://doi.org/10.1002/zoo.21181. PMID: 25323789.

43. Fernando P, Janaka HK, Ekanayaka SKK, Nishantha HG, Pastorini J. A simple method for assessing elephant body condition. Gajah. 2009;31:29-31. https://doi.org/10.5167/uzh-32262.

44. Ramesh T, Sankar K, Qureshi Q, Kalle R. Assessment of wild Asiatic elephant (*Elephas maximus* indicus) body condition by simple scoring method in a tropical deciduous forest of Western Ghats, Southern India. Wildlife Biology in Practice. 2011;7(2):47-54. doi:10.2461/wbp.2011.7.17.

45. Morfeld KA, Lehnhardt J, Alligood C, Bolling J, Brown JL. Development of a body condition scoring index for female African elephants validated by ultrasound measurements of subcutaneous fat. Plos One. 2014;9(4):e93802. https://doi.org/10.1371/journal.pone.0093802. PMID: 24718304.

46. Zhiqiang Y, Li Z. Population and activity characteristic of wild Asian elephant in the Wild Elephant Valley, Xishuangbanna National Nature Reserve, Yunnan, China. Acta Theriologica Sinica. 2006;26(4):359-367. https://doi.org/10.3969/j.issn.1000-1050.2006.04.007.

47. Chaiyarat R, Youngpoy N, Prempree P. Wild Asian elephant Elephas maximus population in

Salakpra Wildlife Sanctuary, Thailand. Endangered Species Research. 2015;29(2):95-102. https://doi.org/10.3354/esr00706.

48. Fan Y, Li Z. Population genetic structure and population genetic diversity analysis based on mitochondrial DNA of Asian elephant Elephas maximus in Chhina. Acta Theriologica Sinica. 2012;32(2):90-100. https://doi.org/10.16829/j.slxb.2012.02.002.

49. Limin F, Zhisheng W, Liu L, Shaobing Y, Bin Z, Chunhua L, et al. Habitat selection in dry season of Asian elephant (*Elephas maximus*) and conservation strategies in Nangunhe National Nature Reserve, Yunnan, China. Acta Theriologica Sinica. 2010;30(1):1-10. https://doi.org/10.16829/j.slxb.2010.01.001.

50. Reddy CS, Unnikrishnan A, Asra M, Manikandan TM, R.Jaishanker. Spatial Conservation Prioritisation of Threatened Forest Ecosystems in Myanmar. Journal of the Indian Society of Remote Sensing. 2019;47(10):1737-1749. https://doi.org/10.1007/s12524-019-01025-2.

51. Peng L, Hui W, Liu L, Jing L, Li Z. Habitat evaluation for Asian elephants (*Elephas maximus*) in Lincang: Conservation planning for an extremely small population of elephants in China. Biological Conservation. 2016;198:113-121. https://doi.org/10.1016/j.biocon.2016.04.005.

52. Yong-jing T, Zhi-sheng W, Gui-lian J, Xuan Z, Yuan H, Zheng-ling L, et al. Population dynamics analysis of Asian elephant in Nangunhe river basin. Forestry Construction. 2019;6:101-106.

53. Barrett SCH, Kohn JR, Falk DA, Holsinger KE. Genetic and evolutionary consequences of small population size in plants: implications for conservation. Genetics & Conservation of Rare Plants. 1991.

54. Ruping W, Lin L. The extinction vortex of small population. Journal of Biology. 2008;25(6):14-16. https://doi.org/10.3969/j.issn.2095-1736.2008.06.004.

55. Tang F, Du F, Sun G. Investigation report on background resources of Nangunhe National Nature Reserve, Yunnan. China Forestry Press. 2015.

56. Guo B, Lan D. Status of wildlife resources in Nangunhe reserve. Chinese Journal of Wildlife. 1999;(4):46-47.

57. BOHNETT, Philip, RIORDAN, Kun S. Initial Assessment on Large and Medium Sized Terrestrial Mammal Assemblage Using Camera Trapping in Nangunhe Nature Reserve in Yunnan, China. Journal of Resources and Ecology. 2015;6(5):331-344. https://doi.org/10.5814/j.issn.1674-764x.2015.05.007.

58. Varma S, Pittet A, Jamadagni HS. Experimenting usage of camera-traps-for population dynamics study of the Asian elephant Elephas maximus in southern India. Current Science. 2007;6(1):81-95. https://doi.org/10.12723/mjs.10.7.

59. Silva Sd, Ranjeewa A, Weerakoon D. Demography of Asian elephants (*Elephas maximus*) at Uda Walawe National Park, Sri Lanka based on identified individuals. Biological Conservation. 2011;144(5):1742-1752. https://doi.org/10.1016/j.biocon.2011.03.011.

60. Vidya TNC, Prasad D, Ghosh A. Individual Identification in Asian Elephants. Gajah. 2014;40:3-17.

61. Sukumar R, Joshi nv, Krishnamurthy V. Growth in the Asian elephant. Proceedings: Animal Sciences. 1988;97(6):561-571. https://doi.org/10.1007/BF03179558.

62. Goswami VR, Lauretta MV, Madhusudan MD, Karanth KU. Optimizing individual identification and survey effort for photographic capture-recapture sampling of species with temporally variable morphological traits. Animal Conservation. 2012;15(2):174-183. https://doi.org/10.1111/j.1469-1795.2011.00501.x.

63. Borchers DL, Efford MG. Spatially explicit maximum likelihood methods for capture-recapture

studies. Biometrics. 2008;64(2):377-385. https://doi.org/10.1111/j.1541-0420.2007.00927.x. PMID: 17970815.

64. Borchers DL, Buckland ST, Zucchini W. Estimating Animal Abundance: Closed Populations. Springer，London. 2002. https://doi.org/10.1007/978-1-4471-3708-5.

65. López-Bao JV, Godinho R, Pacheco C, Lema FJ, García E, Llaneza L, et al. Toward reliable population estimates of wolves by combining spatial capture-recapture models and non-invasive DNA monitoring. Scientific Reports. 2018;8(1):2177. https://doi.org/10.1038/s41598-018-20675-9 PMID: 29391588.

66. Gardner B, Reppucci J, Lucherini M, Royle JA. Spatially explicit inference for open populations: estimating demographic parameters from camera‐trap studies. Ecology. 2010;91(11):3376-3383. https://doi.org/10.1890/09-0804.1. PMID: 21141198.

67. Royle JA, Chandler RB, Sollman R, Gardner B. Spatial Capture-Recapture: Academic Press; 2014.

68. Royle JA, Gardner B. Hierarchical spatial capture-recapture models for estimating density from trapping arrays. In: O'Connell A.F., Nichols J.D., Karanth K.U. (eds) Camera Traps in Animal Ecology. : Springer, Tokyo; 2011.

69. Efford MG. Estimation of population density by spatially explicit capture-recapture analysis of data from area searches. Ecology. 2011;92(12):2202-2207. https://doi.org/10.1890/11-0332.1. PMID: 22352159.

70. SILVEIRA, Leandro, JACOMO, Anah TA, DINIZFILHO, José AF. Camera trap, line transect census and track surveys: a comparative evaluation. Biological Conservation. 2003;114(3):351-355.

71. Li Z, Kang A, Lang J, Xue Y, Ren Y, Zhu Z, et al. On the assessment of big cats and their prey populations based on camera trap data. Biodiversity Science. 2014;22(6):725-732.

https://doi.org/10.3724/SP.J.1003.2014.14079

72. Ling Q. Habitat Selection by Asian Elephant (*Elephas maximus*) in Nangunhe Nature Reserve, Yunnan, China [Masters]: Northwest University; 2007.

73. Yuming Y, Genmin W, Zhisheng Z, Zhongli W. Wild elephant population in Nangunhe reserve. Forest Inventory & Planning. 1982.

74. Tang J, Wang J, Huang X. Summary of Nangunhe nature reserve. Forest Inventory & Planning. 1986.

75. Yuming Y, Fan D. Nangunhe National Nature Reserve in China. Yunnan Science Publication. 2004.

76. Vidya TNC, Sukumar R. Social and reproductive behaviour in elephants. Current Science. 2005;89.

77. Guo B. Investigation and protection advice of wild elephant in Nangunhe reserve. Yunnan Forestry. 1989.

78. Royle JA, Nichols JD, Karanth KU, Gopalaswamy AM. A hierarchical model for estimating density in camera-trap studies. Journal of Applied Ecology. 2010;46(1):118-127. https://doi.org/10.1111/j.1365-2664.2008.01578.x.

79. Alfred R, Ahmad A, Payne J, Williams C, Ambu L, Phua M-H, et al. Home Range and Ranging Behaviour of Bornean Elephant (*Elephas maximus* borneensis) Females. Plos One. 2012;7(2):e31400. https://doi.org/10.1371/journal.pone.0031400. PMID: 22347469.

80. Leggett KEA. Home range and seasonal movement of elephants in the Kunene Region, northwestern Namibia. African Zoology. 2006;41(1):17-36. https://doi.org/10.3377/1562-7020(2006)41[17:Hrasmo]2.0.Co;2.

81. Kumara H, Rathnakumar S, Mavatur A, Singh M. Estimating Asian elephant, Elephas maximus, density through distance sampling in the tropical forests of Biligiri Rangaswamy Temple Tiger

Reserve, India. Tropical Conservation Science. 2012;5(52):163-172. https://doi.org/10.1177/194008291200500206.

82. Baskaran N. RANGING AND RESOURCE UTILIZATION BY ASIAN ELEPHANT (*Elephas maximus* Linnaeus) IN NILGIRI BIOSPHERE RESERVE, SOUTHERN INDIA. 1998.

83. Gubbi S, Swaminath M, Poornesha H, Bhat R, Raghunath R. An elephantine challenge: Human-elephant conflict distribution in the largest Asian elephant population, southern India. Biodiversity and Conservation. 2014;23:633-647. https://doi.org/10.1007/s10531-014-0621-x.

84. Lisa R, Jay H. Some Observations Regarding Allomaternal Caretaking among Captive Asian Elephants (*Elephas maximus*). Journal of Mammalogy. 1987;68(2):438-442. https://doi.org/10.2307/1381494.

85. Lahdenperä M, Mar KU, Lummaa V. Nearby grandmother enhances calf survival and reproduction in Asian elephants. Scientific Reports. 2016;6:27213. https://doi.org/10.1038/srep27213. PMID: 27282468.

86. Kurt F, Hartl G, Tiedemann R. Tuskless bulls in Asian elephant Elephas maximus . History and population genetics of a man-made phenomenon. Acta Theriologica. 1995;40:125-143. https://doi.org/10.4098/AT.arch.95-51.

87. Paulraj S, Subramanian C. Skewed sex ratio in elephant population - How it may affect the demography and population growth? A case study in Mudumalai Wildlife Sanctuary and National Park, Tamil Nadu, Southern India. Indian Forester. 2000;126:1046-1053.

88. Silva Sd, Webber CE, Weerathunga US, Pushpakumara TV, Weerakoon DK, Wittemyer G. Demographic Variables for Wild Asian Elephants Using Longitudinal Observations. Plos One. 2013;8(12):e82788. https://doi.org/10.1371/journal.pone.0082788. PMID: 24376581.

89. Fernando P, Wikramanayake ED, Janaka HK, Jayasinghe LKA, Gunawardena M, Kotagama SW, et al. Ranging behavior of the Asian elephant in Sri Lanka. Mammalian Biology. 2008;73(1):2-13. https://doi.org/10.1016/j.mambio.2007.07.007.

90. Srinivasaiah N, Kumar V, Vaidyanathan S, Sukumar R, Sinha A. All-Male Groups in Asian Elephants: A Novel, Adaptive Social Strategy in Increasingly Anthropogenic Landscapes of Southern India. Scientific Reports. 2019;9:8678. https://doi.org/10.1038/s41598-019-45130-1. PMID: 31273235.

91. Pokharel SS, Seshagiri PB, Sukumar R. Assessment of season-dependent body condition scores in relation to faecal glucocorticoid metabolites in free-ranging Asian elephants. Conservation Physiology. 2017;5(1):cox039. https://doi.org/10.1093/conphys/cox039. PMID: 28721215.

92. Guozheng S, Guangyuan Z, Zicheng Y, Mengjun W. Research progress and direction of human elephant conflict mitigation projects. Forestry Construction. 2019;6:5-9.

93. Sheng-dong Y, Yun D, Ai-dong L, Mang-cai W, Bin W, De Z, et al. A preliminary study of wireless infrared camera technology in alleviating the human-elephant conflict. Forestry Construction. 2019;(6):61-66.

94. Zhi-hong W, Heng-zhen Z, Wen-lan Z, Xuan Z, Wen-bian Y, Zheng-ling L, et al. An analysis of population dynamic on the Xishuangbanna Menghai-Pu'er Lancang Asian elephant population. Forestry Construction. 2019;(6):79-84.

95. xian-ming G, lan-xin W. Thoughts on the construction of Asian elephant food source base in Xishuangbanna. Forestry Construction. 2019;(6):30-33.

Supporting information

S1 Dataset. A simplified database of Asian elephant in NNNR.

S1 Table. Historical elephant population in NNNR estimated by different methods.

Figure 2

Click here to access/download;Figure;Fig2.tif

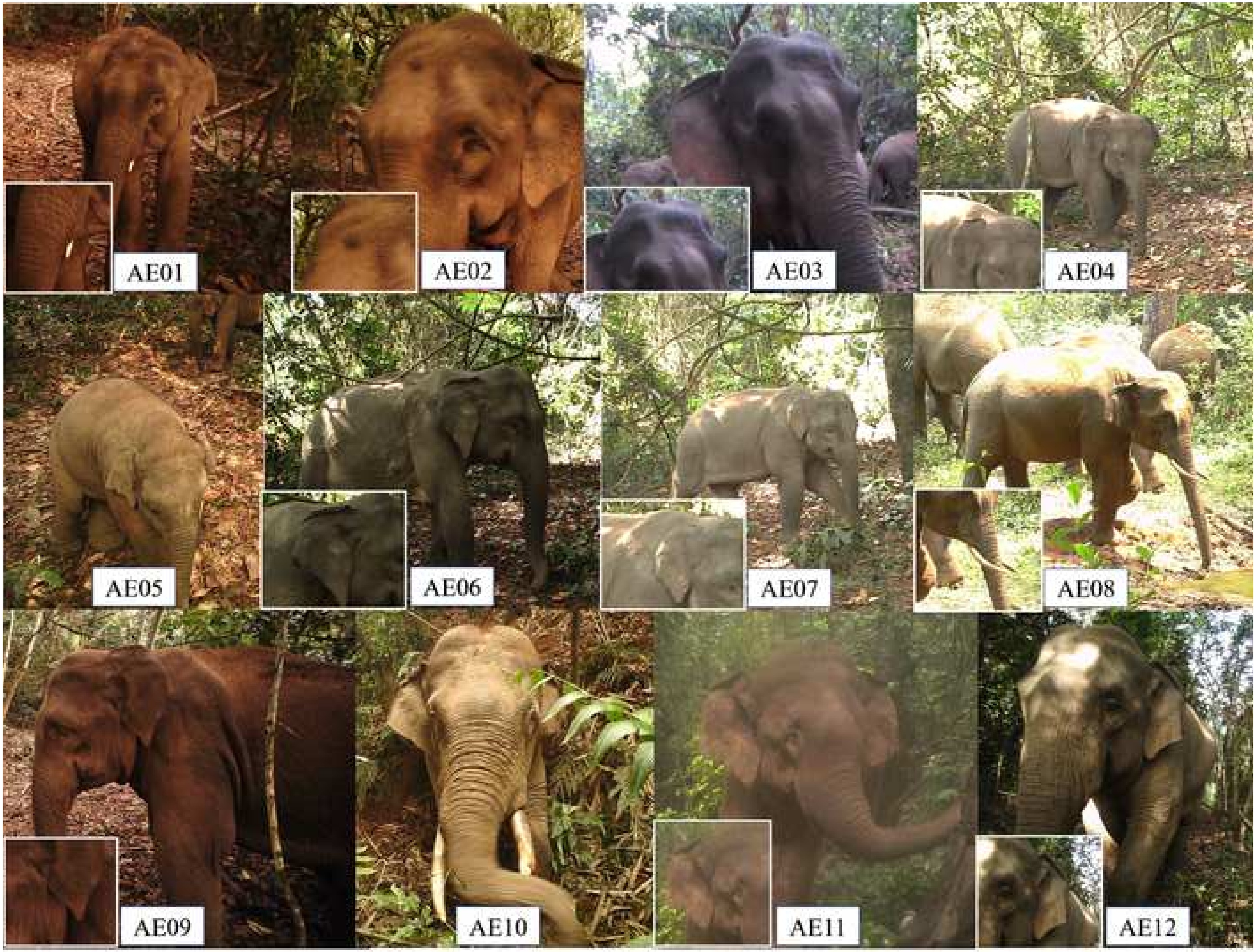

Figure 3

Click here to access/download;Figure;Fig3.tif

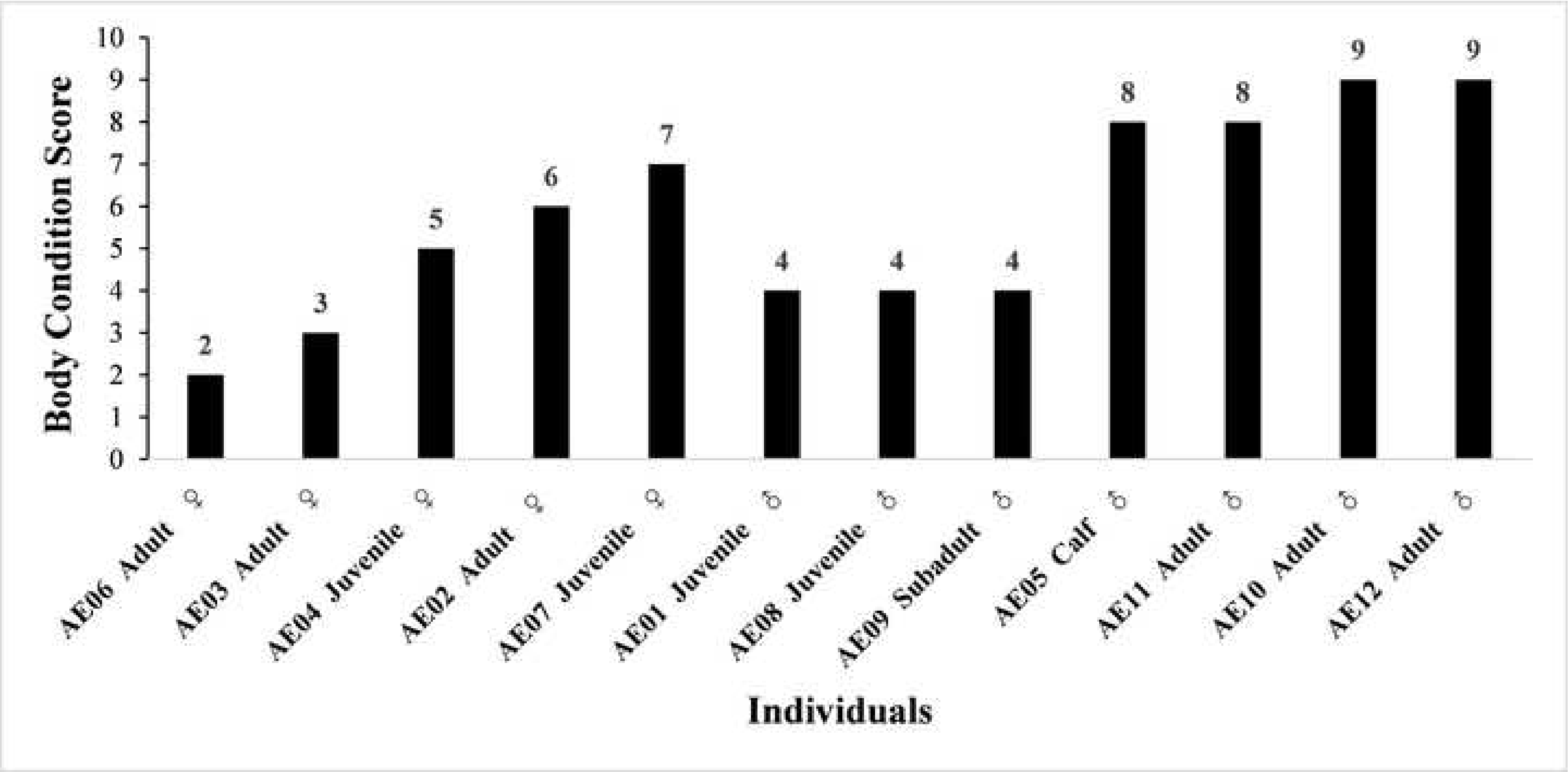

Figure 1

Click here to access/download;Figure;Fig1.tif

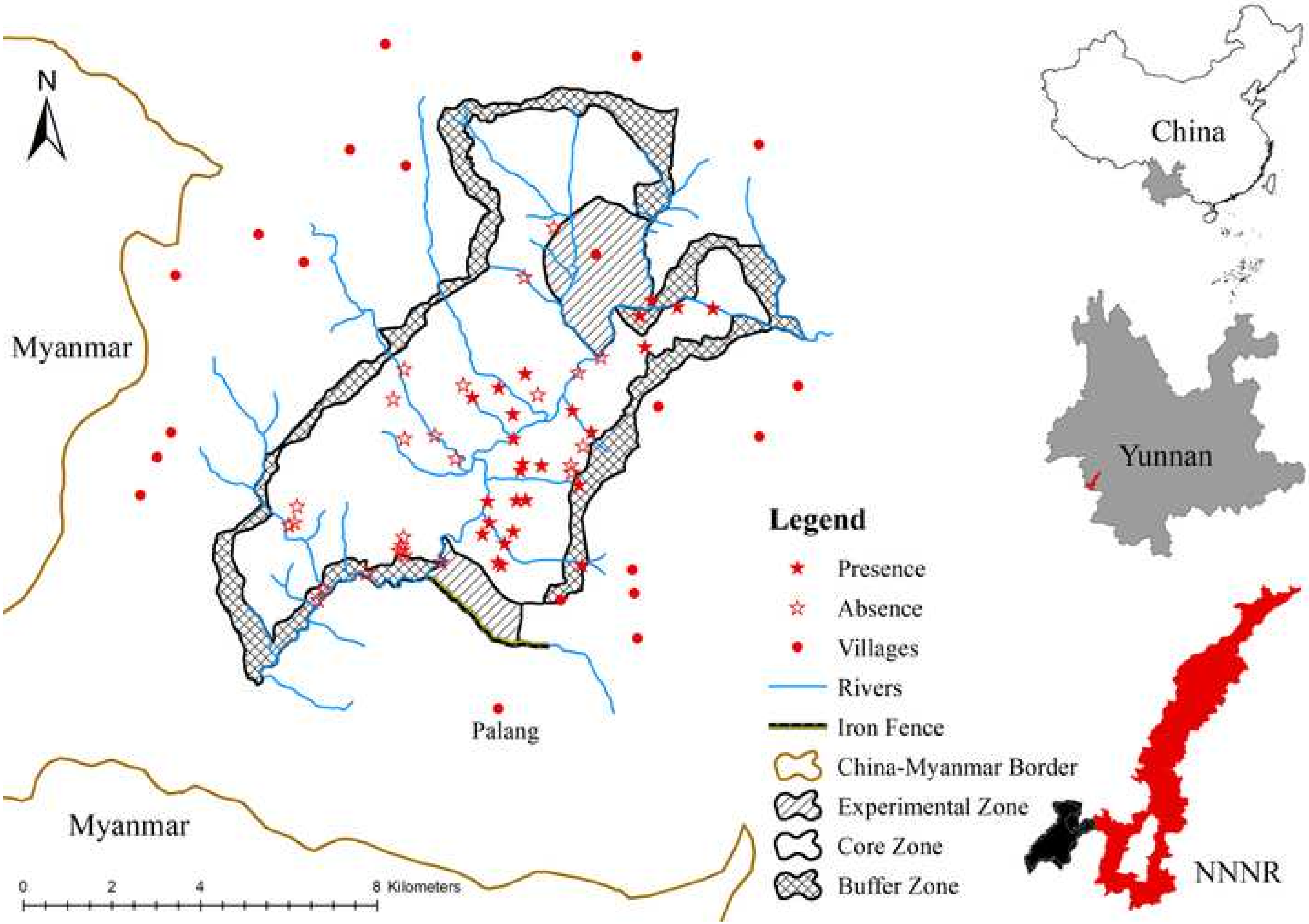

S1 Table

Click here to access/download
Supporting Information
S1 Table.docx

S1 Dataset

Click here to access/download
Supporting Information
S1 Dataset.pdf

Population structure and body condition assessment to inform conservation strategies for a small isolated Asian elephant population in southwest China

Yakuan Sun[1¶], Ying Chen[2¶], Juan José Díaz-Sacco[1¶], Kun Shi [1,3¶*]

[1]School of Ecology and Nature Conservation, Beijing Forestry University, Beijing- 100083, China

[2]School of Biological Science, The University of Hong Kong, Hong Kong, China

[3]Eco-Bridge Continental, Beijing 100085, China

[*]Corresponding author

E-mail: kunshi@bjfu.edu.cn (KS)

¶These authors contributed equally to this work.

Abstract

The Asian elephant (*Elephas maximus*) population in Nangunhe National Nature Reserve represents a unique evolutionary branch in China, which has been isolated for more than twenty years from other neighboring populations in Myanmar. The scarcity of information on population structure, sex ratio and body condition make it difficult to develop effective conservation measures for this elephant population. Twelve individuals were identified from 3,860 valid elephant ~~photographs~~ images ~~at 52 camera sites that were~~ obtained from February to June of 2018 (5,942 sampling effort ~~days~~nights) at 52 camera sites. Three adult females, three adult males, one subadult male, two juvenile females, two juvenile males and one calf male ~~calf~~ were identified. The ratio of adult females to adult males was 1:1~~.4~~, and the ratio of reproductive ability was 1:~~1.5~~0.67, indicating the scarcity of reproductive females as an important limiting factor to population growth. A p~~P~~opulation density of 5.32 ± 1.56 elephants ~~5~~/100 km^2 ~~that~~ was estimated by using Spatially Explicit Capture Recapture (SECR) models ~~was comparatively lower than the crude density estimate of 16.9/100 km^2~~. The health~~A body~~ condition of this elephant population was assessed by using an ~~scoring method that relies on six physical characteristics and ranks the elephant´s body condition according to a~~ 11-points scale of Body Condition Scoring (BCS) method which showed that this ~~was used. Overall, the elephant~~ population had a medium body condition ~~was found to be in normal health,~~ with an average ~~median BCS~~ of 5.75 (n=12, range 2-9). This study finds that this ~~However, as an~~ isolated population ~~with~~ has an extremely small size and ~~as well as~~ an inverted pyramid age structure~~,~~ and therefore the population is at high risk

of extinction~~, ~~. ~~and therefore ~~As such, this population should be of top conservation priority among the few remnant populations in China~~should be of top conservation priority in China~~. The database established in this study can provide researchers with valuable information to set up efficient conservation schemes.

Introduction

The Asian elephant (*Elephas maximus*), as the largest terrestrial mammal in Asia, is considered ~~to be ~~an ecosystem engineer because it can modify its habitat and aid in seed dispersal [1, 2]~~,~~. ~~as well as~~as the conservation of this ~~an ~~umbrella species ~~since its conservation can ~~contributes to the preservation of most of the biodiversity ~~found ~~in the ~~vast ~~ecosystems it uses, especially in tropical moist and in tropical dry broad-leaf forests of Southeast and South Asia [3-5]. It is also considered ~~to be ~~a flagship species that can attract interest for the protection of biodiversity ~~hotspot areas ~~[6]. It is a Class I Key Protected Species in China and ~~is also ~~listed as ~~an ~~Endangered ~~Species ~~under the IUCN Red List [7]~~, showing decreasing population trends~~ [8]. China sustains a population of c. 300 wild Asian elephants which are divided into seven geographic populations [9, 10]. Most Asian elephant populations are located in Mengyang, Mengla and Shangyong areas of Xishuangbanna National Nature Reserve, while there are also a few populations in Simao, Jiangcheng and Lancang counties of Pu'er city and ~~only ~~one small isolated population in the Nangunhe National Nature Reserve (NNNR) of Lincang city [9, 11].

The conventional methods used for tracking Asian elephants in the dense forest are difficult to accomplish and usually fail to provide ~~obtain ~~accurate population

numbers and age structure [12, 13]. Although it is relatively easy to detect elephant signs, such as dungs or footprints, by using sign surveys [14], it can be difficult to identify individual Asian elephants since signs are ~~using only signs is considered to be~~ highly inaccurate and unreliable [15]. ~~By using DNA methods, i~~Individuals can be identified using non-invasive DNA methods, but it requires relatively fresh samples and the lab costs can be quite high; moreover, the population size can only be determined with genetic capture-mark-recapture methods, which can be time and labor intensive [16, 17]. Furthermore, since ~~calf~~ elephant~~s~~ calves have lower defecation rates, ~~these~~ this method~~s~~ can ~~likely~~ underestimate the number of young individuals in a population [18].

Infrared trigger camera technology can make up for the above-mentioned disadvantages [19, 20]. ~~It has many advantages such as~~Infrared cameras ~~causing~~ cause minimal disturbance to the study animal and its habitat, ~~it~~ can be used for long working hours, can withstand ~~and endure~~ harsh weather conditions, and ~~is~~ are relatively ~~of low cost~~cheap [19, 21, 22]. ~~It is~~Infrared cameras are widely used to obtain ~~in~~ ecological baseline surveys of protected species [23, 24], to monitor endangered wildlife [25], to study habitat utilization [26], to observe animal behavior [27], and to study ~~as well as~~ the mechanisms of coexistence in sympatric species [28, 29]. Moreover, ~~the~~ infrared camera can also be used to estimate population size and density by using Spatially Explicit Capture-Recapture models (SECR) [30-33]. This technology has been used extensively ~~used~~ to estimate the population density of multiple species~~felines~~, especially those which possess easily observed markings~~the species that possess natural~~

~~spots or stripes~~, such as the snow leopard (*Panthera uncia*), the leopard cat (*Prionailurus bengalensis*)~~cougar (*Puma concolor*)~~, the Sumatran tiger (*Panthera tigris* ssp. Sumatrae), and the serval (*Leptailurus serval*) [33-36]. For species without natural markings, identification can also be conducted according to morphology, such as with the Asian elephant, the giant panda (*Ailuropoda melanoleuca*), and the fox squirrel （*Sciurus niger*) [37-39].

Population structure and density estimation are essential features that can be used to study the ecology of wildlife populations [40, 41]. The Body Condition Scoring (BCS) is an index of an animal's health that reflects the quality of the environment in which the animal lives [42]. It can be used in conjunction with population structure and density estimation to obtain a more accurate picture of the health of a population. Furthermore, it can be used for both longitudinal and cross-sectional studies, is comparatively inexpensive, and does not require a high technical capacity [43]. While BCS is individual-based, it is most meaningful when applied to a population, as an early indicator of the impact of management actions on the average health condition of a population [43, 44]. For Asian elephants, the BCS obtained through photographs can be as accurate as ultrasonic subcutaneous fat measurement, thus providing a safer and non-invasive method to evaluate the health condition of wild populations [43, 45].

Throughout the Asian elephant range, there have been a number of studies that have identified individual elephants by directly photographing them or by obtaining images from infrared cameras [17, 37, 46, 47]. In China, ~~these~~ population studies have mainly been concentrated in Xishuangbanna National Nature Reserve [17, 46], with a

few conducted in NNNR, focusing on population size estimation, from individual identification, but not on the density or the health of its population. assessment of its populations.

Studies conducted on the elephant population in NNNR concluded that it was a separate evolutionary unit– isolated from other geographic populations [13, 48], thus lacking genetic connectivity. Although the closestother elephant populations can be found in nearby Myanmar, the forest degradation in the China-Myanmar border area has led to the isolation of this elephant population since 1997 [9, 49, 50]. Additionally, this population is facing threats from habitat loss, fragmentation and illegal hunting [49, 51, 52]. Although conservation interventions have been proposed to rescue this population from extinction, the lack of scientific data and information on population structure, relatedness, activity pattern, and health status prevent the development of efficient conservation strategies.

Body condition is an index of an animal's health, since it can reflect the quality of the environment in which it lives . Body condition scoring (BCS) of Asian elephants based on photographs is considered to be as accurate as ultrasonic subcutaneous fat measurement, thus making it easier to evaluate the health condition of wild populations since individuals do not need to be captured . BCS is individual-based, but it is most meaningful when applied to a population, especially small isolated populations . It can be used for both longitudinal and cross-sectional studies, is comparatively inexpensive and does not require a high technical capacity . However, there have been no studies focusing on assessing the health of any of the Asian elephant populations in China.

Isolated populations are vulnerable to changes in the external environment and thus, their conservation should be a top priority [53, 54]. However, previous ~~research~~ studies on Asian elephant in China were only limited to studying the population size and did not ~~analyze~~ assess the health condition of the population [13, 52]. An ~~Accurate~~ accurate understanding of the current situation of the local elephant population in NNNR, especially its structure ~~sex ratio~~ and health condition~~breeding information.~~ , is key to establishing a baseline for the development of ~~a successful project focusing on the introduction of individual elephants from other populations and develop~~ more effective protection and management schemes.

~~Therefore, it is essential to study the population structure and health condition of the Asian elephant in NNNR.~~ In this study, our aim was to ~~use camera trapping to~~ determine the ~~number~~size, density, demographic structure and healthy condition of the ~~this~~ NNNR Asian elephant population by using infrared camera trapping.~~,~~ We sought~~and~~ to ~~analyze~~ understand which ~~the~~ factors ~~that~~ might have affected its population size in recent years in order to develop targeted and effective protection strategies. Furthermore, our approaches can be applied to other elephant populations ~~in China~~ with further implications for global Asian elephant conservation.

Material and Methods

Ethics Statement

Both National Forestry and Grassland Administration of China and Yunnan Forestry and Grassland Administration reviewed all sampling procedures and approved permits for the work conducted in NNNR. Non-invasive methods were applied and

approval from an Institutional Animal Care and Use Committee or equivalent animal ethics committee was therefore not required.

Study area

The study area is located in the western part of the Nangunhe National Nature Reserve, Yunnan, where elephants are found [49, 51] (Fig 1). It occupies 70.82 km^2 at latitude 23°13'- 23°19'N~~23° 13′ - 23° 19′~~, longitude 98°54'- 99°05'E~~98° 54′ - 99° 05′~~, and the altitude ranges from 510 m to 1747 m. Rainfall is highly seasonal, with a dry season between November ~~to~~ and April, and a rainy season between May ~~to~~ and October. Mean ~~Annual~~ annual precipitation is 351.3 mm in the dry season and 1983.3 mm in the rainy season, while~~and~~ temperature varies at different altitudes. The average monthly temperature range is hottest in July, varying between 12.3-25.5 ℃ and it is coldest in January with values between 2.8-14.3 ℃~~are 40.1 mm - 1371.8 mm and 15.8-24.5 ℃, respectively~~ [55]. Vegetation and climate vary across elevation zones. ~~Natural t~~Tropical forest and tropical monsoon forest are located at altitudes below 600 m,~~.~~ m~~M~~iddle elevation forests are dominated by subtropical monsoon evergreen broad-leaf forests,~~.~~ e~~E~~vergreen broadleaf ~~vegetation~~ forests are~~is~~ found above 1700 m [51]. Bamboo forest, shrub and grasslands are scattered throughout this area. Deforestation along the China and Myanmar border has disrupted the connectivity between elephant populations that are found in both ~~of these~~ countries [49]. ~~The~~ Furthermore, an iron fence in the southern part of reserve is used to protect the surrounding villages from wildlife (Fig 1).

Fig 1. Map of study area in NNNR showing ~~all~~ the camera sites with elephants (solid

pentacle~~presence points~~) or without elephants (hollow pentacle~~absence points~~).

Camera trap~~s~~ set-up

We carried out the survey for four months between February and May 2018 using automatically triggered camera traps (Ltl Acorn 6210). There was a total of 52 cameras stations which successfully recorded 43-129 camera trap nights in the field (Fig 1). The total camera trapping effort was 5,942 trap nights (114.27±19.83). All the stations were located in evergreen broadleaf forest, broadleaf and bamboo forest, bamboo forest, pine forest and nearby the brush forest, which represented the preferred habitat of elephants in NNNR. We systematically deployed camera traps along the animal trails ~~with~~ and forest gaps where there were signs of elephants~~, —~~, including lying sites, foraging sites, footprints, dung~~s, foraging sites as well as other activity sites~~ and urine marks. Cameras had attempted to be deployed throughout the reserve in a 1 km^2 grid formation. We increased the number of cameras in the grids with many elephant signs, but always with a minimum of 200 m between any two cameras. ~~However, w~~We avoided the northwestern part of the reserve as the topography is tough, with ~~where there were~~ steep cliffs~~slopes at higher altitudes~~ of up to 1747 meters, and historically, there have not been any records of elephants in this area [56, 57].

Cameras were strapped onto trees at approximately 1.5 m above the ground with the sensor parallel to the ground to maximize the extension of the detection zone. The positions of all the camera sites were recorded by GPS. To avoid blank images caused by sunlight, the cameras were never placed ~~away from the~~facing east~~ direction~~. Camera traps were ~~deployed as~~ hidden as much as possible to reduce the disturbance to wildlife

and the probability of been being stolen. Camera traps were set to operate 24 hours per day and programmed to take sequential three photographs and one 12 video as a sequential without delay, registering date and time for each exposure. At each site, a camera traps were was operated continuously for a maximum of four months. SD cards and batteries were replaced every month. We recorded the time of installation and retrieval of each camera and calculated the total duration of sampling.

Individual identification

The images obtained from the camera traps were categorized to independent events, which were defined as a “sequence”. Each sequence is defined by a set of images adjacent in time. For an elephant unit (defined as all or the maximum number of individuals of a group), the sequence includes the images from the first one individual entering the field to the last one exiting it, making it possible to identify different individuals based on distinct features. For solitary individuals, the sequence is a series of captured images of same individual within a continuous time periodThe photographs obtained from the camera traps were categorized to independent events, which were defined as sequences, meaning from the first individual entering the field to the last one exiting it in the same camera, with at least up to 5 hours between two connected sequences [58]. Only photographs images with sufficient quality, clarity and position of the elephant in the frame were used for individual identification [58]. Here, quality referred to how usable an picture image was for age and sex classification; clarity indicated whether the elephant was “captured” in good light, – or that the flash reached the individual, and the picture image was in focus and not over or under-

exposed; and position referred to the occurrence of the elephant within an ‘optimal distance and angle’ from the camera [47, 58]. The age of each animal was broadly classified into adult, subadult, juvenile and calf using approximate shoulder height as recommended by previous studies, and gender was mainly determined by secondary sexual characteristics [59-61] (Table 1). ~~Gender of an elephant was mainly determined by its secondary characteristics. Females were recognized by their apparent breasts and lack of tusks. Males were identified by their distinctive tusks, lack of breasts and whenever possible, by the presence of a penis.~~ We defined the age structure as the ratio of adults, subadults, juveniles and calves while the reproductive ability was the ratio of age structure between adult and juvenile females [47], due to the absence of subadult and calf females in this reserve. Male elephants living completely or mostly outside the herd were classified as solitary males [12]. Based on individual events, a capture history event database was established to record the individuals and their characteristics. Morphological characteristics including facial bones, ear, tusk, back and tail hair, old scars and injuries, ~~that did not change in a short period of time were clearly described~~as well as physical characteristics related to sex and age classes, ~~confirming~~ confirmed the reliability of identification results [17, 37, 46, 62] (Table 1). ~~According to the shooting time of Asian elephant photos, their daily activity patterns were also summarized.~~

Table 1. Morphological characteristics used for individual identification of elephants photographed in NNNR, between February and May 2018.

Characteristics	Categories	Description
Gender	Adult f~~F~~emale	Without obvious tusks but with prominent breasts, belly swelling or the presence of nursing

		offspring
	Adult m~~M~~ale	With obvious tusks or visible penis but without prominent breasts, the presence of penis for tuskless individual
	Subadult male	Without obvious tusks but with visible penis
	Juvenile female	Without obvious tusks
	Juvenile male	With obvious tusks
	Calf male	Only the testicles or penis were visible
Age	Adult (>15years)	With the highest shoulder height
	Subadult (5-15years)	Shoulder height reaches ~~the~~ an adult's eyes
	Juvenile (1-5years)	Shoulder height reaches half the height of an adult
	Calf (0-1year)	Below ~~the~~ an adult's knee
Facial bones	Wisdom tumor	Size
	Bones between two eyes	Extent of prominence
	Upper margin of eyes	Extent of depression~~Sunken prominence~~
Tusks	Length	Very short/short/long compared with trunk
	Thickness	Thick/normal/slender
	Shape	Straight/bend
	Crack	Yes/no
Ear	Upper edge	Fold/unfold
	Lobe	V-acute/U-rounded
	Lobe tear	Yes/no
	Ear hole	Yes/no
Tail brush	Standard	Present/both sides/both-continuous
	Nonstandard	Absent/single side/both-discontinuous

Density estimation

The elephants in NNNR belong to~~were identified as~~ a small isolated population without any migrants, thus conforming to the "close population" assumption of ~~Spatial Explicit Capture-Recapture models (~~SECR~~)~~ [63]. The model accounts for the movement in density estimation by combining a state model and an observation model, which describe how the observed data were generated [63]. The state model describes

statistically the mechanisms determining the distribution of the animals~~the movement pattern of individuals activity center~~ in study area, while the observation model ~~(spatial detection model)~~ ~~describes~~ quantifies the probability of detection or capture, given the searched region, their locations and characteristics at different sessions[64]~~detecting an individual at a particular detector (e.g. camera trap in this study) to the distance of the detector from a central point in each individual's activity center~~. Basically, SECR are generalized linear mixed models that assume that every individual *i* in the population has its own unobserved activity center S_i, and all the activity centers $S_i \cdots S_N$ are distributed across the study area [65, 66].

The encounter rate of an individual *i* with a given ~~detector~~ trap *j* located at X_j is a monotonically decreasing function of the distance from S_i to *j*. Y_{ij}, the number of times individual *i* is detected by trap *j* during a sampling occasion, is a random variable following a Poisson distribution and with mean λ_{ij} [67]:

$$Y_{ij} \sim \text{Poisson}(\lambda_{ij})$$

For an elephant *i* captured by a trap *j*, the model assumes a log-linear form [68]:

$$\text{Log}(\lambda_{ij}) = \text{Log}(\lambda_0) - \left(\frac{1}{2\sigma^2}\right) \|S_i - X_j\|$$

Here, λ_0 is the baseline encounter probability, ~~and~~ the function $\|S_i - X_j\|$ is the distance between the activity center and trapping station, and σ is the Gaussian scale parameter for the distance function between activity centers and detector locations~~,~~; assuming a bivariate normal model of space use, sigma can be used to calculate a 95% activity area radius~~home range radius~~.

~~Density is a derived parameter calculated by dividing N by the area of the state space S, which N is the number of activity center in S, includes the trapping grids.~~ SECR models estimate the density of animal activity centers in an area large enough so that animals residing beyond it have a negligible chance of being detected [63]. Considering the small size of the elephant distribution, with the east and west boundary being no wider than~~We used a 5~~ 10 km, we therefore defined our study area as extending 5 km beyond all cameras stations,~~ buffer from the outermost x and y coordinates of the camera sits which could cover the study area,~~ corresponding to an area ~~S~~ of 255.8 km^2[69].

SECR 3.1.5 package in R ~~Language~~ 3.6.1 was used for density estimation. ~~Therefore, d~~Data ~~for analysis in SECR was~~were prepared as an R object of class “capthist”. This object included both the capture data and the detector layout. The file named “capture histories” ~~containing~~ contained data on individual identification, the occasion (~~day~~each sample time, that is event here), the detector (camera ID). The detector file ~~containing~~ contained data on cameras ID, and x, y coordinates. Five different sessions, corresponding to each of the months and one corresponding to the whole survey period, were used~~We used monthly and whole data as five different sessions~~ to run the model to see the estimation accuracy under different detection efforts.

Body condition scoring

We established a database of each identified individual and the ~~photographs~~ images with the best angle of each individual elephant were selected to score the body condition. Compared with previous studies, our target population size was small, and

the sample size was limited so we used a simple method which can rapidly and more easily be applied to wild elephant populations. In this study, we applied a BCS method that focuses on six physical characteristics and ranks the elephant´s body condition according to an 11-points scale [42, 43]. Five of the clearest images for each physical characteristic were selected to assess the body condition. Thirty images were used to calculate the comprehensive scores for each individual. This scoring method is based on the extent of visibility of depressions around bone structures. Depressions around bones become visible as an animal loses its subcutaneous fat deposits and muscles in the region concerned, thereby making bones appear more prominent. Skull, pectoral girdles in shoulders, vertebral column, ribs and pelvic girdles are prominent bone structures. Thick rolls of skin that fold below the neck can accumulate fat, and thus, also represent a criteria of good body condition~~This method is based on a scale of 5 reference photographs of target scored elephants that focused on the ribs, shoulders, pelvic girdles, backbone and neck rolls~~ . We presented a~~The~~ scale of 5 reference scored elephants description ~~photographs were assigned scores~~ of 1, 3, 5, 7, 9, with numerically higher values corresponding to better body condition (Table 2). ~~But~~ However, the range of scores extended from 0 to 10 due to the possibility of greater variation. For example, i~~I~~f all the ribs of an elephant were visible, it meant that this individual presented a worse body condition than 1 and thus, it would be assigned 0. If an individual presented a fatter condition than 9 and had very thick rolls of skin fold below neck, it would be assigned a score of 10. Scores ranging from 0-3 were considered as poor, 4-7 were considered medium and 8-10 were considered as good

[44].~~ All statistical analyses were carried out using SPSS. C~~Pearson correlation coefficient (r) was used to study the relationship between BCS and ~~ratios of~~ various covariates such as sex, age, and whether an individual was solitary ~~present~~ or ~~not~~ in ~~the~~ a herd.

Table 2. Diagnostic character~~i~~istics pertaining to scores in photographic scale.

Score	Character~~i~~istics
1	Ribs from shoulder to pelvis are visible, some ribs prominent (spaces in between sunken in)
3	Some ribs visible (spaces in between not sunken in), shoulder and pelvic girdles prominent
5	Ribs not visible, shoulder and pelvic girdles visible
7	Backbone visible as a ridge, shoulder and pelvic girdles not visible
9	Backbone not visible or difficult to differentiate and pelvic bones not visible

Results

~~Individual identification~~Population size and density

~~We carried out camera trap surveys at 52 locations (Fig 1) for 5,942 trap days (114.27 ± 19.83 (SD); range: 73-129). All the camera traps were systematically deployed in broad-leaf forest, broad-leaf and bamboo forests, bamboo forest, pine forest and nearby the brush forest, which represented the dominant habitat types in NNNR.~~ A total of 70,902 ~~photos and videos~~images were obtained, among which 7.34% (5,207) were Asian elephant data. There was a total of 134 independent events, including 40 events of elephants in a herd ~~independent events~~ and 94 events of solitary elephants~~individual independent events~~. Excluding the image data that could not be used for individual identification, there were 3,860 valid Asian elephant ~~photos and videos~~images belonging to 85 independent events (26 events of individuals in a herd and 59 events of individuals). Generally, all herds and individuals had similar

frequencies of recapture histories during the study period, particularly herds. However, solitary elephants were image-captured with a minor difference: a minimum of 16 capture times of AE12 compared to a maximum of 25 capture times of AE10 (Table 3).~~A total of 12 individuals were identified among valid independent events (Fig 2). A simple database information is shown in S1 Table. The minimum population number of Asian elephants in each month was consistently 12. Photographic frequencies were different among individuals and capture histories: elephant number 12 (AE12) accounted for the least frequent (16) of the records whereas all other individuals were captured at least 20 times (Table 3).~~

~~**Fig 2. Several different characteristics used for individual identification of elephants.** All the pictures come from different capture events. A & B were juvenile males and treated as two individuals according to A's short straight tusks and B's long curve tusks; C & D were treated as the same individual by the V-acute of ear lobe; E was distinguished by its distinctive protuberance on the center of forehead; Both F & G had similar tail hair but were identified by others features.~~

Table 3. Number of capture events for a given individual per month.

Individual No.	Age	Gender	Tusk	Type	Capture events				
					Feb.	Mar.	Apr.	May	Total
AE01	Juvenile	Male	Yes	Herd	2	6	5	8	21
AE02	Adult	Female	**No**	Herd	3	5	6	6	20
AE03	Adult	Female	**No**	Herd	3	5	6	7	21
AE04	Juvenile	Female	**No**	Herd	2	6	5	7	20
AE05	Calf	Male	No	Herd	3	6	8	5	22
AE06	Adult	Female	**No**	Herd	4	6	8	5	23
AE07	Juvenile	Female	**No**	Herd	4	6	9	6	25
AE08	Juvenile	Male	Yes	Herd	4	7	6	7	24

AE09	Sub-adult	Male	No	Solitary	3	9	2	9	23
AE10	Adult	Male	Yes	Solitary	2	3	13	7	25
AE11	Adult	Male	No	Solitary	3	5	8	5	21
AE12	Adult	Male	No	Solitary	2	6	1	7	16
Total	-	-	-	-	35	70	77	79	261

A total of 12 individuals were identified among these valid independent events (Fig 2; S1 Dataset). The elephant population size in each session was consistent, except in February. Moreover, the population size estimated by SECR was 12.00 ± 0.71 (with a 95% confidence interval of 12.00-12.11; Table 4). The estimated density by SECR was 5.32 ± 1.56 elephants/100km^2 (with a 95% confidence interval of 3.02-9.36). In camera-trapping session at monthly intervals, April had the lowest density estimation at 6.07 ± 1.79 elephants/100km^2, while May had the highest density estimation at 24.43 ± 7.20 elephants/100km^2. The movement parameter σ was twice as large in April (6643 m ± 2356 m) than in May (3771 m ± 1471 m).

Different types of behaviors, such as foraging (including milk feeding of calves), drinking water, sand bathing, sleeping, communication and the use of branches were recorded. A total of six scenes of Asian elephants lying down and resting were recorded on video, including that of one calf, four juveniles and one adult female. The longest sleeping time recorded was 1 h 36 min for adult females. The herd of elephants clearly showed a higher daily activity frequency at 6:00-8:00 and 14:00-16:00, while the activity time of the solitary elephants lagged behind at 8:00-10:00 and 18:00-20:00 respectively.

Fig 2. Asian elephants individually identified in NNNR, China. Each individual's most distinguished characteristic was emphasized, except easily identified AE05 and

AE10, the only calf and adult male with tusks.

Table 4. Population density estimation by using SECR models under five sessions.

Sessions	Occasions	Density			λ_0		sigma		Population size		
		estimate	SE.estimate	95% CI	estimate	SE.estimate	estimate	SE.estimate	estimate	SE.estimate	95% CI
February	11	10.71	3.31	5.91-19.38	0.15	0.04	2983	664	14.00	2.19	12.35-23.43
March	26	6.57	1.94	3.73-11.58	0.07	0.02	4193	700	12.20	0.87	12.00-17.95
April	22	6.07	1.79	3.45-10.70	0.02	0.004	6643	2356	12.05	0.75	12.00-16.87
May	26	24.43	7.20	13.87-43.03	0.01	0.003	3711	1471	12.03	0.74	12.00-16.48
Total	85	5.32	1.56	3.02-9.36	0.02	0.003	6474	1065	12.00	0.71	12.00-12.11

Demographic structure ~~and relatedness~~

We identified an inverted pyramid age structure for this population: three adult females, three adult males, one subadult male, two juvenile females, two juvenile males and one male calf,~~.~~ with a sex ratio of 1:1.4 using females as a reference (Table 4). The three adult males comprised one tusker and two without tusks (Makhnas), giving an adult tusked to tuskless male ratio of 1:2. The subadult male was also a makhna, increasing the tusked to tuskless male ratio to 1:3. The ratio of reproductive ability was 1:0.67.

~~For the sex, the male to female ratio was 1.4:1, for adult as well as juvenile individuals, the ratio was 1:1. The ratio of reproductive ability was 1:1.5. Judging from camera trap data, there were two containment relationship family units.~~ The social

networking judging from camera trap data is AE01-AE08 formed the joint big family unit, while individuals calf AE05, adult female AE06 and juvenile female AE07 as a mother-calf formed the small unit which occasionally joined and departed from the large big family unit. Adult female AE06 and male calf AE05 were definitely in a mother-child relationship since there were photographs images of the calf sucking milknursing from the female., and juvenile AE07 might have a kindred relationship with AE05. Adult female AE03 and juvenile female AE04 were most likely in a mother-child relationship since lots of image data showed AE04 actively moving close to AE03. The solitary subadult male AE09 and adult male AE10 were observed to occasionally join the family unit.

Population density

The estimated density was 5.32±1.56 individuals per 100 km^2 (with a 95% confidence interval of 3.02-9.36) and the population size was 12.00±0.71 (with a 95% confidence interval of 12.00-12.11) (Table 4). April had the lowest density estimation at 6.07±1.79 individuals per 100 km^2, while in May the density reached 24.43±7.20 individuals per 100 km^2. The movement parameter σ was twice as large for April (6643m±2356m) than for May (3771m±1471m). Each session had a similar population size, excluding the standard error estimate in February which was higher than in the remaining sessions. The 95% confidence interval reached 12.35-23.43.

Table 4. Population density estimation by using SECR models under five sessions.

Sessions	Occasions	Density			λ_0		sigma		Population size		
		estimate	SE.estimate	95% CI	estimate	SE.estimate	estimate	SE.estimate	estimate	SE.estimate	95% CI
February	11	10.71	3.31	5.91-19.38	0.15	0.04	2983	664	14.00	2.19	12.35-23.43
March	26	6.57	1.94	3.73-11.58	0.07	0.02	4193	700	12.20	0.87	12.00-17.95
April	22	6.07	1.79	3.45-10.70	0.02	0.004	6643	2356	12.05	0.75	12.00-16.87
May	26	24.43	7.20	13.87-43.03	0.01	0.003	3711	1471	12.03	0.74	12.00-16.48
Total	85	5.32	1.56	3.02-9.36	0.02	0.003	6474	1065	12.00	0.71	12.00-12.11

Body condition scoring

The distribution of BCS for the population had an average median BCS of 5.75 (n=12, range 2-9) (Fig 3). Body condition evaluation revealed that 33.3% of the elephants were in a good health condition, while 50% and 16.7% were in medium, and poor health conditions respectively (Fig 3). Collectively, only one individual had a minimum BCS of 2 and two had a maximum BCS of 9, respectively. Half of the individuals reached above the median scores. The frequency of BCSs by sex is shown in Fig.3. BCS ranged 2-7 in females and 4-9 in males. The reproductive adult female AE02 had a BCS just above the median score while the other reproductive female AE03 had a lower BCS of 3. The calf's BCS was 8, juveniles' BCS ranged from 4-7, subadult was 4, adults' BCS ranged from 2-9. Age group was not significantly correlated to the BCS (r= 0.03). Solitary or not and sex played more important roles in the BCS (r = 0.53 and 0.42 respectively). Age was not significantly correlated to BCS (r=0.03), but sex was (r=0.42), as adult males had better body conditions than adult females. The body condition of the only three fertile females in this population was not good. The adult

female AE02 had a BCS just above the average score, while the other females, AE03 and AE06 were in poor health conditions with a low BCS of 3. Being solitary or in the herd played more important roles in the BCS (r=0.53) than sex. Individuals that were part of the herd had a lower BCS than solitary individuals.

Fig 3. Body condition score of the 12 individuals identified in NNNR.

Discussion

~~Asian elephant p~~Population size and density ~~number in NNNR~~

Accurate assessment of population size is essential for species conservation and management [70]. I~~ndividual i~~dentification from captured ~~photos and videos~~images was robust to identify individuals ~~.~~[71], ~~Unlike~~ unlike dung~~s~~ and footprints ~~that~~ which are easily influenced by slope, soil quality and climatic factors ~~,~~[72]. ~~this~~ This study ~~installed~~ used cameras installed in the elephant~~'s~~ preferred habitats that covered most of the western part of the reserve, which provided a higher chance of obtaining the data reflecting the real population, leading to a more reliable population assessment as in the study that used drone surveys [52]. Historical overview of the research on this population estimated by several different methods may generate inevitably a bias in direct comparison, the NNNR population showed a population size fluctuating around 20 individuals, and a tendency towards a slight population decrease [52, 72-74]. Nowadays, the distribution for this population still remains in the western part of the NNNR Cangyuan county [52, 73, 75] (Fig 1).~~Overall, the population size identified in this study was consistent with the survey results of 40 years ago , that determined that~~

~~it presented a small population size status fluctuating around 20 individuals . Compared with other Asian elephant populations in China as well as in other Asian countries, the NNNR population is definitely an extremely small population .~~ Therefore, we assumed that there is space available in NNNR to allow for this small population to reach its highest ever recorded population size of 24-25 individuals [56, 73]. ~~Unlike other small populations in China, such as the Xishuangbanna Menghai-Pu'er Lancang population with 19 individuals distributed outside the reserve that can move freely around farmlands and natural forest , the~~ The NNNR population is~~–~~ an extremely small and isolated population, compared with other elephant populations found around the world [3, 9, 10, 12]~~completely locked into this small area~~. Historical ~~Illegal~~ illegal hunting ~~could have~~ has had a direct impact on this population with a total of ~~16~~ 15 unnatural deaths since 1987 (Table 5). Additionally, the high mortality of immature elephants (75%) among these unnatural deaths since 2003, as well as a long adolescence and long inter-birth intervals [12, 76] have had potentially negative effects on this populations growth. ~~Moreover, inconsistent survey methods that have been adopted throughout the years might explain slight differences in population size results (see more details in S2 Table). All these results indicate that the small population size in NNNR is in urgent need of conservation interventions.~~

Table 5. Unnatural deaths of Asian elephant since the establishment of ~~the~~ NNNR.

Year	Number of individuals	~~Reason~~Cause of death	Data resources

1987	Two: One female and one male	Illegal killing	[55, 77]
1988	Four: Two adult males, one adult female and one calf	Poaching, electrocuting and unknown reason	[49]
1995	One male	Unknown	[52]
1996	One male	Vengefully hunted by local people	[51]
1997	Three: One female, one male and a calf	Unknown for the calf, the female and male were killed	[55]
2003	A Subadult male (about 10 years old) ~~and one adult male~~	Unknown ~~for the subadult, the adult male died for its ivory~~	[55]
2008	~~A~~ One ~~Subadult~~ subadult male	Unknown	Communication with local residents and rangers
2011	One juvenile	Unknown	[55]
2016	One ~~Adult~~ adult male	~~The cause of death was u~~Unknown, but the tusks show visible saw marks	Communication with local residents and rangers

SECR modelling has become a powerful tool to estimate the small population sizes like the one in NNNR [32]. The model defines the activity center according to the range of the species activities [78], so it is easy to overestimate the population density in a short survey period, which was confirmed by monthly estimates in this study (Table 4).

We first used the single monthly session to run the SECR model, with which we obtained a significant difference in density estimation. The SECR model simulated a larger movement buffer (sigma=6643 m) in April, which resulted in the smallest population density. May presented the highest density because there were 8 cameras that captured elephants, and the distance between each one was less than 800 m, which is smaller than its daily movement distance [79, 80]. The instability of single month session results by SECR, prompted us to use the four months data as the fifth session to run the model. We thus obtained the smallest standard deviation, which indicated that the result was closer to the actual value [47].

The population density results of the NNNR elephant population, as an unique evolutionary clade in China, can be used to compare with results from other populations in order to improve our understanding of elephant ecology and develop more detailed management and conservation plans [81]. However, with no other SECR analyses conducted on other populations in China, we used the crude density estimation (estimated population number/study area) to draw comparison between the NNNR population and others in Yunnan [47]. The crude density estimation of Asian elephants in NNNR was the highest at 16.9 elephants/100 km^2 due to its smaller area of distribution, compared with that in Shangyong, Mengla, and Menyang with a density of 10.68, 4.26, and 1.85 elephants/100 km^2 respectively [13, 17]. This is concerning as a high population density could lead to more intensive competition among individuals for limited natural resources [82], and even greater human-elephant conflict as elephants resort to crop raiding [83].

Population structure in NNNR

~~Our surveys showed differences in age structure and sex composition of the NNNR population compared with another recent study . We identified one more adult male elephant than in this study, a more detailed age ranking structure and sex composition among young elephants, and even identified the male calf by observing the penis sheath from high quality images. There are more adult and subadult individuals in the NNNR than juveniles and calves, thus indicating a declining population trend in the future.~~ Although the j~~J~~uveniles in this population accounted for 33% of the population, ~~although studies have shown that it is suitable for juveniles to account for more~~higher than the minimum of 20% of the population considered to be needed for ~~the population~~it to be self-sustaining in a short period of time ~~.~~ [18], inbreeding is inevitable in the near future due to the small population size.

We attempted to identify the relationship between individuals in the family unit through images captured by camera trap, since the allomaternal care or mother-like behaviour directed by females are prevalent in the Asian elephant social structure [84, 85]. We could not match adult females with their offspring in the groups relying on the observed behaviours, even though we captured intimate interaction between AE02 and AE08, as well as between AE03 and AE04. In recent years, the death of adult females has not been recorded in the reserve (Table 5). Therefore, we assumed that all the current juveniles came from these three adult females. ~~Small populations are likely to suffer from inbreeding depression . Two mother-calf relationships were identified from camera trap photos, and the NNNR population was likely to be a big family unit, which~~

may not be conducive to the long-term survival of the population. Genetic studies are urgently needed to better understand the lineage and degree of inbreeding depression in the NNNR population.

There were two tuskless juveniles that had similar body heights as the juvenile males identified by their apparent tusks and these were identified as females. However, it should be noted that mMales in some populations tend to be tuskless for survival due to the intensive historical poaching pressure [12, 86].elephants are commonly the first choice of poachers since illegal ivory-based poaching leads to a serious gender imbalance, with females outnumbering males . For example, the ratio of female to male in Shangyong reserve in China is 10.5:1, and as high as 100:1 in Periyar Tiger reserve in India . The NNNR population had an even sex ratio for adults which was consistent with a recent population survey by aerial drones . Large number of females could have a long-term negative effect on the population as shown by the decrease in the number of effective elephants, the decrease in the birth rate, and the low genetic viability caused by inbreeding, while a balanced sex ratio can avoid negative effects driven by skewed sex ratio .

The high proportion of tuskless adult malesmakhnas in NNNR identified from this investigation could indicate that these juveniles that were identified as females might in fact be males. We judged these juveniles as females under the condition that they have similar body height as juvenile males who had apparent tusks. Males in some populations tend to be tuskless for survival due to the intensive historical poaching pressure . The number of unnatural deaths in the past 30 years was skewed towards

~~males (Table 5) and at least 116 individual deaths in the past century might account for tuskless males .~~Since tuskless males are common in this population, it would be helpful to conduct genetic studies on this population that can confirm the sex of these two juveniles. Unbalanced sex ratio is not beneficial for population growth [87]. ~~track the historical trend of the proportion of males with or without tusks in order to obtain scientific evidence-based explanations for the current sex ratio and proportion of tuskless males . Moreover, genetic studies on elephant specimens and living individuals could give us more insights on the sex ratio trend in the last few decades as well as in the proportion of tuskless males. If further molecular studies confirm that all current juveniles are males, this could lead to a relatively high skewed sex ratio.~~ Currently, only AE02, AE03 and AE06 are ~~in~~ of reproductive age, but the latter was ~~nurturing~~ nursing a calf, leaving only two sexually active females in the reserve. The reproductive ratio of this small population was relatively high compared to the findings in Ruhuna National Park, Sri Lanka [83], and Salakpra Wildlife Sanctuary, Thailand [47], ~~Since~~ since females do not enter the breeding period until they are 10-14 years old and ~~afterwards they have a long birth interval (4-5 years between calves),~~as such these two ~~female~~ juvenile females in the NNNR population~~s~~ still need 3-7 years to become mature [9, 12, 88]. In either case, the scarcity of fertile females would be the primary constraint on population growth due to their long birth intervals.

~~Population density~~

~~Accurate assessment of population size is essential for species conservation and management . Before this study, only crude density estimation (estimated population~~

number/study area) was available to compare the density of Asian elephants in different regions . The crude density estimate of Asian elephants in NNNR was the highest at 16.9/100 km^2 comparing with that in Shangyong, Mengla, Menyang with a density of 10.68, 4.26, 1.85 per 100 km^2 respectively . Although elephants are known to swim across the river, a short iron fence was built on the opposite side of the river in the southern boundary of this part of the reserve thus restricting their range (Fig 1), none signs were found in this area and no records of elephant invasion from the settlements. Therefore, the fence and the high elevations found on the northwestern side of the reserve are obstacles that reduce the activity zones for the NNNR population. High population density could lead to more intensive competition among individuals for various natural resources .

At present, SECR models have become a powerful tool to estimate small population sizes like the one in NNNR , with the results being more robust than by using crude density estimation. Compared other population density in India by using SECR, the NNNR population density was quite low . The model defines the activity center according to the range of the species activities , so it is easy to overestimate the population density in a short monitoring time period, which was confirmed in this study. May presented the highest density because there were 8 cameras that captured elephants, and the distance between each one was less than 800 m. Thus, after four months of monitoring, more accurate elephant movement data was obtained, and we got the smallest standard deviation of density value and number, which indicated that the results of the total data were closer to that of the real value . A minimum number of 20

~~elephants were estimated in NNNR under the assumption of a population density of 0.1-0.3 elephant km^{2}, which is commonly seen in tropical forests across elephant range countries . Thus, our estimate of 12 elephants is likely to be below the area´s carrying capacity. Therefore, we assumed that there is enough space in NNNR to allow for this elephant population to reach its highest ever recorded population size of 24-25 individuals .~~

Health condition

~~Due to the small sample size, this study could not adopt more detailed assessment methods and only focused on dry season, but the results can still reflect the physical condition of NNNR population as well as the individual differences in range, foraging and behavioral activities . Sex was a significant factor affecting BCS. Females, however, usually have higher BCS than males as documented in other Asian and African elephant populations as well as in dogs and horses . However, a trend towards higher body fat in males compared to females in NNNR population was found.~~ We chose a simple but accurate body condition method to assess the health of this small population. Our results showed a higher body condition of adult males than adult females, which~~This situation~~ was also observed in another elephant population in Southern India [44]. This may be because adult ~~Solitary~~ males are known to have larger home ~~areas~~ ranges than adult females and immature males [80, 89], and are more tolerant to human disturbance [90]. ~~family units, and adopt a high-risk high-gain foraging strategy by venturing into agricultural areas and feeding on nutritious crops . The camera sites that only captured adult males also recorded highly~~ Which means they explore areas near the reserve

boundary frequently, where intensive anthropogenic activities ~~which included~~like hunting, gathering, fishing and collecting have also been recorded by cameras. Solitary elephants could have also had a higher BCS than the individuals in the family units because at least one of the herds could have had an even more reduced area of movement due to the presence of a calf [89]. Furthermore, additional nutritional stress from lactation for AE06 could have resulted in an even poorer body condition [44]. Additionally, the other two adult females both presented a medium health condition, which would not be conducive for reproduction [42].~~Besides, nutritional stress in lactating females (AE06) could have resulted in their poor body condition . The BCS of juveniles ranged 4-7, which is slightly lower than median scores were reasonable. Difference in patterns of fat deposition may exist between sexes, age-groups and sub-species. Previous studies reported that depressions and bony appearance were more visible in adult elephants than in subadults and juveniles .~~

Most of the individuals (66.7%) had medium or poor body conditions during the survey period. Since ~~The~~ the quantity and quality of food and availability of water are the primary factors determining body condition of free-ranging elephants ~~in the wild~~ [42], both female and male individuals show an obvious thinner body condition during the dry season compared to the rainy season [91].~~while there is also less availability of nutritional food during the dry season.~~ We did not have the opportunity to conduct camera trap surveys during the rainy season ~~Since~~ since ~~non-waterproof~~many cameras failed to work in ~~the rainy~~this season~~, we did not have the opportunity to conduct camera trap surveys during this period of time~~. Additionally, the rainy season coincides

with the harvest period. However,In the past, especially in recent years, NNNR elephant damage compensation data has shown thatbased on the data recorded by the bureau to compensate villagers for damages caused by elephants in NNNR, the elephants have been recorded foraging forage for corn and rice during the rainy season from the month of July to October (data from the compensation documentary of the reserve bureau). Therefore, we predict that this population is foraging off of crops to supplement its diet. Thus, future studies should focus on determining if crops could improve the body condition of elephants during the rainy season. Establishing the food base in an accessible area for the family unit during the whole year could improve their health condition in dry season. Therefore, the body condition of the elephant is not only a reflection of its health but also an indicator of other factors that affect body condition, such as energy reserve, fitness for reproduction and breeding behaviour that can help managers elaborate future conservation schemes .

Moreover, the body condition assessment in this study couldcan have implications for the research and conservation of other elephant populations in China. Since the vast majority of Asian elephant populations, especially in China, are found outside of reserves and near cropland, the health of these elephants are likely heavily influenced by access to cropscan have access to plenty of food crop resources [92].; Massive amounts of data have been collected by camera traps and drones in these areas [93, 94], but none have been used to determine the health of the populations. We have proven that using images for BCS to be a simple and fast method for determing the health of free-ranging elephant populations.thus causing serious conflicts with local inhabitants .

Therefore, future studies should consider analyzing the body condition of different regional Asian elephant populations from these data sets to~~can~~ be used as an early indicator of the health of individuals and quality of food resources in their habitats~~impact of management actions .~~[43]. ~~The data can directly reflect the obesity level of individuals among the population, and indirectly reflect the status of food resources in areas of distribution, which can~~This in turn can be used to guide conservationists and policymakers to evaluate the necessity for interventions and define regions for suitable habitats restoration~~restore suitable habitats as well as improve crop management schemes~~ [42].

Conservation recommendations

To maintain this extremely small isolated elephant population, the long-term placement of cameras in NNNR is needed to get valuable data for the study of demography, fecundity and health status [47, 58, 59, 91]. Molecular biological approaches are also needed in further studies to determine the kinship and genetic health of this population [41].

The results of this study have reconfirmed that the enlargement of suitable habitat is urgently needed to ensure an adequate supply of food is available for this isolated population during the dry season [13, 49, 51]. Attention should be paid to the low body condition in the dry season and a folow-up survey carried out in the rainy season as soon as posible to verify the health status of this elephant population throughout the year. Therefore, the short-term objective is should be to supplement the food shortages the NNNR population during the dry season by establishing or improving the vegetation

quality of the food-sources [95]. Fence dismantlement, as the mid-term goal, should be prioritized because it has partially blocked the movement of elephants and hindered their ability to find new food resources (Fig 1). Allowing the animals greater movement could provide more opportunities for the elephants to explore new suitable areas with enough food, eventually improving the health condition regardless of the season. Ecological corridors should be considered as a long-term objective to connect this isolated elephant population to other suitable habitats. Since the western part of this reserve (Fig 1. Red part of NNNR) has some suitable habitats [51], an ecological corridor could be created by planting bamboo forest or bamboo-broadleaf forests, allowing this population to migrate between the eastern and western part of the reserve. We also suggest that research on the evaluation of the environmental capacity and availability of food resources should be conducted to set up a scientific baseline. Due to the very small population size and the scarcity of reproductive females, all suggestions should be urgently accomplished before the juveniles reach reproductive age to avoid potential inbreeding.

~~By using camera trap photos, NNNR was found to have a small elephant population with an uneven age structure as well as few females in reproductive period with relatively poor body conditions. Since the long-term placement of cameras in the wild can provide valuable data for the study of demography, reproductive and health status of Asian elephants , systematic camera surveys and continuous long-term monitoring of NNNR population could aid in eliminating errors in the population size and density estimated in this study, by incorporating data on survival and reproductive~~

rates as well as movement patterns. Small population size, inbreeding and restricted gene flow all contribute to the reduction in the genetic diversity , which has been shown to affect population persistence and individual fitness as well as adaptation, thus likely increasing the risk of extinction . Population viability analyses for Asian elephant show that such a small population may have a 70% probability of extinction from chance alone if the deterministic population growth rate is zero . The presence of both breeding females and males, and the availability of areas with suitable habitat and resources, however, offer some hope for partial recovery. External conditions like strict protection measures, the promotion of the “Elephant Worshipping Festival” and successful strategies to mitigate human-elephant conflicts can all have a positive effect on its population recovery . Molecular techniques should be conducted in the near future to obtain information related to kinship, reproductive matters as well as physical health. The results obtained in this study have reconfirmed that the reintroduction of individual Asian elephants should be put on top of the conservation agenda of China and that the enlargement of suitable habitat is urgently needed to ensure the richness of food resources for this isolated population . Preferred elephant habitats that were confirmed from previous studies, such as mixed forests and thickets below 1000 m , should be prioritized for habitat restoration. The main activity regions have now been partially blocked by a thick iron fence to separate settlements from elephants. This approach can partly relieve human-elephant conflict . However, it has also completely isolated this elephant population from other populations, and is considered to be a money-wasting and time-consuming approach. Fence dismantlement could provide more opportunities

~~for elephants to explore new suitable activity places and areas with enough food resources. Due to the very small population size and the scarcity of reproductive females, the translocation of Asian elephants must be urgently accomplished before the juveniles reach reproductive age to avoid inbreeding . We also suggest that research on the evaluation of the environmental capacity and availability of food resources should be conducted to set up a scientific baseline before a detailed plan on the reintroduction of new individuals can be made.~~

Acknowledgments

This study was implemented by Beijing Forestry University and Beijing Eco-Bridge Continental. We thank ~~for~~ the ~~support received from the~~ Second National Survey of Terrestrial Wildlife in China~~,~~ and National Forestry and Grassland Administration of China for their support of this study. Furthermore, t~~T~~he field work was strongly supported by the administrators of Nangunhe National Nature Reserve, for which we thank them. We also thank all the field staff, including Wang Jiahui, Liu Zhen, Liu Ying and all local rangers, especially Li Yongxiang, Wang Zhisheng, Li Chunlian and Li Zhimin, for their hard and invaluable work.

References

1. McConkey KR, Nathalang A, Brockelman WY, Saralamba C, Santon J, Matmoon U, et al. Different megafauna vary in their seed dispersal effectiveness of the megafaunal fruit Platymitra macrocarpa (Annonaceae). Plos One. 2018;13(7):e0198960. https://doi.org/10.1371/journal.pone.0198960. PMID: 30020929.

2. Campos-Arceiz A, Blake S. Megagardeners of the forest – the role of elephants in seed dispersal. Acta Oecologica. 2011;37(6):542-553. https://doi.org/10.1016/j.actao.2011.01.014.

3. Riddle HS, Schulte BA, Desai AA, Meer Lvd. Elephants - A conservation overview. Journal of Threatened Taxa. 2010;2(1):653-661.

4. Aijun T, Lingjiang Y. Distribution and Protection of Asian elephant in China. Protection Forest Science and Technology. 2015;(5):89-91. https://doi.org/10.13601/j.issn.1005-5215.2015.05.035.

5. Platt SG, Bickford DP, Win MM, Rainwater TR. Water-filled Asian elephant tracks serve as breeding sites for anurans in Myanmar. Mammalia. 2019;83(3):287-289. 10.1515/mammalia-2017-0174

6. Bowen-Jones E, Entwistle A. Identifying appropriate flagship species: the importance of culture and local contexts. Oryx. 2002;36(2):189-195. https://doi.org/10.1017/S0030605302000261.

7. Jiang Z, Jiang J, Wang Y, Zhang E, Zhang Y, Li L, et al. Red List of China's Vertebrates. Biodiversity Science. 2016;24(5):500-551. http://www.biodiversity-science.net/EN/10.17520/biods.2016076.

8. Choudhury A, Lahiri Choudhury DK, Desai A, Duckworth JW, Easa PS, Johnsingh AJT, et al. Elephas maximus. The IUCN Red List of Threatened Species. 2008:e.T7140A12828813. https://dx.doi.org/10.2305/IUCN.UK.2008.RLTS.T7140A12828813.en.

9. Li Z. Research on Asian elephant Conservation in China: Science Press; 2018.

10. Yu Z, Kun J. Distribution, Population, Habitat Status and Population Management of Asian Elephant. World Forestry Research. 2018;31(2):25-30. https://doi.org/10.13348/j.cnki.sjlyyj.2018.0002.y.

11. Mingyong C. Asian elephant research of China: Science Press; 2006.

12. Sukumar R. A brief review of the status, distribution and biology of wild Asian elephants Elephas

maximus. International Zoo Yearbook. 2006;40(1):1-8. https://doi.org/10.1111/j.1748-1090.2006.00001.x.

13. Zhang L, Dong L, Lin L, Feng LM, Yan F, Wang LX, et al. Asian Elephants in China: Estimating Population Size and Evaluating Habitat Suitability. Plos One. 2015;10(5):e0124834. https://doi.org/10.1371/journal.pone.0124834. PMID: 25992617.

14. Tian Y, Feng Y, Zhang C, Yu B, Tang X, Hu H. Effectiveness of line transects during wild animal surveys in mountain forests of South China. Biodiversity Science. 2015;23(1):109-115. http://www.biodiversity-science.net/EN/10.17520/biods.2014128.

15. Barnes RFW. The problem of precision and trend detection posed by small elephant populations in West Africa. African Journal of Ecology. 2002;40:179-185. https://doi.org/10.1046/j.1365-2028.2002.00376.x.

16. Mills LS, Citta JJ, Lair KP, Schwartz MK, Tallmon DA. Estimating Animal Abundance Using Noninvasive DNA Sampling: Promise and Pitfalls. Ecological Applications. 2000;10(1):283-294. https://doi.org/10.2307/2641002.

17. Zicheng Y, Ying C, Junsong L, Lifan W, You P, Zhiyong S, et al. Individual identification and population size assessment for Asian elephant based on camera-trapping techniques. Acta Theriologica Sinica. 2018;38(1):18-27. https://doi.org/10.16829/j.slxb.150109

18. L.S.EGGERT, J.A.EGGERT, D.S.WOODRUFF. Estimating population sizes for elusive animals: the forest elephants of Kakum National Park, Ghana. Molecular Ecology. 2003;12(6):1389-1402. https://doi.org/10.1046/j.1365-294X.2003.01822.x. PMID: 12755869.

19. Liu X, Wu P, He X, Zhao X. Application and data mining of infra-red camera in the monitoring of species. Biodiversity Science. 2018;26(08):850-861. https://doi.org/10.17520/biods.2018053.

20. Xiao W, Hu L, Huang X, Xiao Z. Using capture-recapture models in wildlife camera-trapping monitoring and the study case. Biodiversity Science. 2019;27(3):257-265. https://doi.org/10.17520/biods.2018194

21. Zhang L, Cui S, Huang Y, Chen D, Qiao H, Li C, et al. Infrared camera traps in wildlife research and monitoring in China: issues and insights. Biodiversity Science. 2014;22(6):696-703. https://doi.org/10.3724/SP.J.1003.2014.14225.

22. Wearn OR, Glover-Kapfer P. Snap happy: camera traps are an effective sampling tool when compared with alternative methods. Royal Society Open Science. 2019;6(3):181748. https://doi.org/10.1098/rsos.181748. PMID: 31032031.

23. Jorge A A, Carlos E F S, Krisna G, Chris H, Johanna H, Emanuel M, et al. Community structure and diversity of tropical forest mammals: data from a global camera trap network. Philosophical Transactions of the Royal Society of London. 2011;366(1578):2703-2711. https://doi.org/10.1098/rstb.2011.0115. PMID: 21844049.

24. Chengcheng Z, Jun W, ALEXANDER JS, Zhigang D, Liji W, Wantao D, et al. Biodiversity Assessment of Mammal and Bird Species from Camera Trap Data in Yanchiwan National Nature Reserve, Gansu Province, China. Journal of Resources and Ecology. 2018;9(5):566-574. https://doi.org/10.5814/j.issn.1674-764x.2018.05.014.

25. Broto B, Mortelliti A. The status of research on the mammals of Sulawesi, Indonesia. Mammal Review. 2019;49(1):78-93. https://doi.org/10.1111/mam.12141.

26. Montalvo VH, Sáenz-Bola˜nos C, Luis D. Alfaro1 JCC, Guimarães-Rodrigues FH, Carrillo E, Sutherland C, et al. Seasonal use of waterholes and pathways by macrofauna in the dry forest of Costa Rica. Journal of Tropical Ecology. 2019;35(2):68-73.

https://doi.org/10.1017/S0266467418000457.

27. Matsukawa A, Ahmad AH, Kohshima S. Social structure and reproduction of long-tailed porcupine (*Trichys fasciculata*). Mammalia. 2019;83(1):23-33. https://doi.org/10.1515/mammalia-2017-0104.

28. Sunarto S, Kelly MJ, Parakkasi K, Hutajulu MB. Cat coexistence in central Sumatra: ecological characteristics, spatial and temporal overlap, and implications for management. Journal of Zoology. 2015;296(2):104-115. https://doi.org/10.1111/jzo.12218.

29. Rich LN, Miller DAW, Muñoz DJ, Robinson HS, McNutt JW, Kelly MJ. Sampling design and analytical advances allow for simultaneous density estimation of seven sympatric carnivore species from camera trap data. Biological Conservation. 2019;233:12-20. https://doi.org/10.1016/j.biocon.2019.02.018.

30. BLANC L, MARBOUTIN E, GATTI S, GIMENEZ O. Abundance of rare and elusive species: Empirical investigation of closed versus spatially explicit capture-recapture models with lynx as a case study. The Journal of Wildlife Management. 2013;77(2):372-378. https://doi.org/10.1002/jwmg.453.

31. Efford MG, Fewster RM. Estimating population size by spatially explicit capture-recapture. Oikos. 2013;122(6):918-928. https://doi.org/10.1111/j.1600-0706.2012.20440.x.

32. Bashir T, Bhattacharya T, Poudyal K, Sathyakumar S, Qureshi Q. Estimating leopard cat Prionailurus bengalensis densities using photographic captures and recaptures. Wildlife Biology. 2013;19(4):462-472. https://doi.org/10.2981/12-098.

33. Alexander JS. Advancing research and conservation of snow leopards in the Qilianshan mountains of China: management and methodological implications [Doctoral dissertation]: Beijing Forestry University; 2015.

34. O'Brien TG, Kinnaird MF, Wibisono HT. Crouching tigers, hidden prey: Sumatran tiger and prey populations in a tropical forest landscape. Animal Conservation. 2003;6(2):131-139. https://doi.org/10.1017/S1367943003003172

35. Bohm T, Hofer H. Population numbers, density and activity patterns of servals in savannah patches of Odzala-Kokoua National Park, Republic of Congo. African Journal of Ecology. 2018;56(4):841-849. https://doi.org/10.1111/aje.12520.

36. Park H, Lim A, Choi T-Y, Baek S-Y, Song E-G, Park YC. Where to spot: individual identification of leopard cats (*Prionailurus bengalensis* euptilurus) in South Korea. Journal of Ecology and Environment. 2019;43. https://doi.org/10.1186/s41610-019-0138-z.

37. Goswami VR, Madhusudan MD, Karanth KU. Application of photographic capture-recapture modelling to estimate demographic parameters for male Asian elephants. Animal Conservation. 2007;10(3):391-399. https://doi.org/10.1111/j.1469-1795.2007.00124.x.

38. Tye CA, Greene DU, Giuliano WM, Mccleery RA. Using camera-trap photographs to identify individual fox squirrels (*Sciurus niger*) in the Southeastern United States. Wildlife Society Bulletin. 2015;39(3):645-650. https://doi.org/10.1002/wsb.573.

39. Zheng X, Owen MA, Nie Y, Hu Y, Swaisgood RR, L.Yan, et al. Individual identification of wild giant pandas from camera trap photos - a systematic and hierarchical approach. Journal of Zoology. 2016;300(4):247-256. https://doi.org/10.1111/jzo.12377.

40. Gupta M, Joshi A, Vidya TNC. Effects of social organization, trap arrangement and density, sampling scale, and population density on bias in population size estimation using some common mark-recapture estimators. Plos One. 2017;12(3):e0173609. https://doi.org/10.1371/journal.pone.0173609. PMID: 28306735.

41. Hedges S, Johnson A, Ahlering M, Tyson M, Eggert LS. Accuracy, precision, and cost-effectiveness of conventional dung density and fecal DNA based survey methods to estimate Asian elephant (*Elephas maximus*) population size and structure. Biological Conservation. 2013;159:101-108. https://doi.org/10.1016/j.biocon.2012.12.010.

42. Wijeyamohan S, Treiber K, Schmitt D, Santiapillai C. A Visual System for Scoring Body Condition of Asian Elephants (*Elephas maximus*). Zoo Biology. 2015;34(1):53-59. https://doi.org/10.1002/zoo.21181. PMID: 25323789.

43. Fernando P, Janaka HK, Ekanayaka SKK, Nishantha HG, Pastorini J. A simple method for assessing elephant body condition. Gajah. 2009;31:29-31. https://doi.org/10.5167/uzh-32262.

44. Ramesh T, Sankar K, Qureshi Q, Kalle R. Assessment of wild Asiatic elephant (*Elephas maximus* indicus) body condition by simple scoring method in a tropical deciduous forest of Western Ghats, Southern India. Wildlife Biology in Practice. 2011;7(2):47-54. 10.2461/wbp.2011.7.17.

45. Morfeld KA, Lehnhardt J, Alligood C, Bolling J, Brown JL. Development of a body condition scoring index for female African elephants validated by ultrasound measurements of subcutaneous fat. Plos One. 2014;9(4):e93802. https://doi.org/10.1371/journal.pone.0093802. PMID: 24718304.

46. Zhiqiang Y, Li Z. Population and activity characteristic of wild Asian elephant in the Wild Elephant Valley, Xishuangbanna National Nature Reserve, Yunnan, China. Acta Theriologica Sinica. 2006;26(4):359-367. https://doi.org/10.3969/j.issn.1000-1050.2006.04.007.

47. Chaiyarat R, Youngpoy N, Prempree P. Wild Asian elephant Elephas maximus population in Salakpra Wildlife Sanctuary, Thailand. Endangered Species Research. 2015;29(2):95-102. https://doi.org/10.3354/esr00706.

48. Fan Y, Li Z. Population genetic structure and population genetic diversity analysis based on

mitochondrial DNA of Asian elephant Elephas maximus in Chhina. Acta Theriologica Sinica. 2012;32(2):90-100. https://doi.org/10.16829/j.slxb.2012.02.002.

49. Limin F, Zhisheng W, Liu L, Shaobing Y, Bin Z, Chunhua L, et al. Habitat selection in dry season of Asian elephant (*Elephas maximus*) and conservation strategies in Nangunhe National Nature Reserve, Yunnan, China. Acta Theriologica Sinica. 2010;30(1):1-10. https://doi.org/10.16829/j.slxb.2010.01.001.

50. Reddy CS, Unnikrishnan A, Asra M, Manikandan TM, R.Jaishanker. Spatial Conservation Prioritisation of Threatened Forest Ecosystems in Myanmar. Journal of the Indian Society of Remote Sensing. 2019;47(10):1737-1749. https://doi.org/10.1007/s12524-019-01025-2.

51. Peng L, Hui W, Liu L, Jing L, Li Z. Habitat evaluation for Asian elephants (*Elephas maximus*) in Lincang: Conservation planning for an extremely small population of elephants in China. Biological Conservation. 2016;198:113-121. https://doi.org/10.1016/j.biocon.2016.04.005.

52. Yong-jing T, Zhi-sheng W, Gui-lian J, Xuan Z, Yuan H, Zheng-ling L, et al. Population dynamics analysis of Asian elephant in Nangunhe river basin. Forestry Construction. 2019;6:101-106.

53. Barrett SCH, Kohn JR, Falk DA, Holsinger KE. Genetic and evolutionary consequences of small population size in plants: implications for conservation. Genetics & Conservation of Rare Plants. 1991.

54. Ruping W, Lin L. The extinction vortex of small population. Journal of Biology. 2008;25(6):14-16. https://doi.org/10.3969/j.issn.2095-1736.2008.06.004.

55. Tang F, Du F, Sun G. Investigation report on background resources of Nangunhe National Nature Reserve, Yunnan. China Forestry Press. 2015.

56. Guo B, Lan D. Status of wildlife resources in Nangunhe reserve. Chinese Journal of Wildlife.

1999;(4):46-47.

57. BOHNETT, Philip, RIORDAN, Kun S. Initial Assessment on Large and Medium Sized Terrestrial Mammal Assemblage Using Camera Trapping in Nangunhe Nature Reserve in Yunnan, China. Journal of Resources and Ecology. 2015;6(5):331-344. https://doi.org/10.5814/j.issn.1674-764x.2015.05.007.

58. Varma S, Pittet A, Jamadagni HS. Experimenting usage of camera-traps-for population dynamics study of the Asian elephant Elephas maximus in southern India. Current Science. 2007;6(1):81-95. https://doi.org/10.12723/mjs.10.7.

59. Silva Sd, Ranjeewa A, Weerakoon D. Demography of Asian elephants (*Elephas maximus*) at Uda Walawe National Park, Sri Lanka based on identified individuals. Biological Conservation. 2011;144(5):1742-1752. https://doi.org/10.1016/j.biocon.2011.03.011.

60. Vidya TNC, Prasad D, Ghosh A. Individual Identification in Asian Elephants. Gajah. 2014;40:3-17.

61. Sukumar R, Joshi nv, Krishnamurthy V. Growth in the Asian elephant. Proceedings: Animal Sciences. 1988;97(6):561-571. https://doi.org/10.1007/BF03179558.

62. Goswami VR, Lauretta MV, Madhusudan MD, Karanth KU. Optimizing individual identification and survey effort for photographic capture-recapture sampling of species with temporally variable morphological traits. Animal Conservation. 2012;15(2):174-183. https://doi.org/10.1111/j.1469-1795.2011.00501.x.

63. Borchers DL, Efford MG. Spatially explicit maximum likelihood methods for capture-recapture studies. Biometrics. 2008;64(2):377-385. 10.1111/j.1541-0420.2007.00927.x. PMID: WOS:000255959500007.

64. Borchers DL, Buckland ST, Zucchini W. Estimating Animal Abundance: Closed Populations.

Springer，London. 2002. https://doi.org/10.1007/978-1-4471-3708-5.

65. Gardner B, Reppucci J, Lucherini M, Royle JA. Spatially explicit inference for open populations: estimating demographic parameters from camera - trap studies. Ecology. 2010;91(11):3376-3383. 10.2307/20788170.

66. López-Bao JV, Godinho R, Pacheco C, Lema FJ, García E, Llaneza L, et al. Toward reliable population estimates of wolves by combining spatial capture-recapture models and non-invasive DNA monitoring. Scientific Reports. 2018;8(1):2177. https://doi.org/10.1038/s41598-018-20675-9 PMID: 29391588.

67. Royle JA, Chandler RB, Sollman R, Gardner B. Spatial Capture-Recapture: Academic Press; 2014.

68. Royle JA, Gardner B. Hierarchical spatial capture-recapture models for estimating density from trapping arrays. In: O'Connell A.F., Nichols J.D., Karanth K.U. (eds) Camera Traps in Animal Ecology. : Springer, Tokyo; 2011.

69. Efford MG. Estimation of population density by spatially explicit capture-recapture analysis of data from area searches. Ecology. 2011;92(12):2202-2207. https://doi.org/10.1890/11-0332.1. PMID: 22352159.

70. SILVE[illegible]A, Leandro, JACOMO, Anah TA, DINIZFILHO, José AF. Camera trap, line transect census and track surveys: a comparative evaluation. Biological Conservation. 2003;114(3):351-355.

71. Li Z, Kang A, Lang J, Xue Y, Ren Y, Zhu Z, et al. On the assessment of big cats and their prey populations based on camera trap data. Biodiversity Science. 2014;22(6):725-732. https://doi.org/10.3724/SP.J.1003.2014.14079

72. Ling Q. Habitat Selection by Asian Elephant (*Elephas maximus*) in Nangunhe Nature Reserve, Yunnan, China [Masters]: Northwest University; 2007.

73. Yuming Y, Genmin W, Zhisheng Z, Zhongli W. Wild elephant population in Nangunhe reserve. Forest Inventory & Planning. 1982.

74. Tang J, Wang J, Huang X. Summary of Nangunhe nature reserve. Forest Inventory & Planning. 1986.

75. Yuming Y, Fan D. Nangunhe National Nature Reserve in China. Yunnan Science Publication. 2004.

76. Vidya TNC, Sukumar R. Social and reproductive behaviour in elephants. Current Science. 2005;89.

77. Guo B. Investigation and protection advice of wild elephant in Nangunhe reserve. YuUnnan Forestry. 1989.

78. Royle JA, Nichols JD, Karanth KU, Gopalaswamy AM. A hierarchical model for estimating density in camera-trap studies. Journal of Applied Ecology. 2010;46(1):118-127. https://doi.org/10.1111/j.1365-2664.2008.01578.x.

79. Alfred R, Ahmad A, Payne J, Williams C, Ambu L, Phua M-H, et al. Home Range and Ranging Behaviour of Bornean Elephant (*Elephas maximus* borneensis) Females. Plos One. 2012;7(2):e31400. https://doi.org/10.1371/journal.pone.0031400. PMID: 22347469.

80. Leggett KEA. Home range and seasonal movement of elephants in the Kunene Region, northwestern Namibia. African Zoology. 2006;41(1):17-36. https://doi.org/10.3377/1562-7020(2006)41[17:Hrasmo]2.0.Co;2.

81. Kumara H, Rathnakumar S, Mavatur A, Singh M. Estimating Asian elephant, Elephas maximus, density through distance sampling in the tropical forests of Biligiri Rangaswamy Temple Tiger Reserve, India. Tropical Conservation Science. 2012;5(52):163-172. https://doi.org/10.1177/194008291200500206.

82. Baskaran N. RAN[illegible]G AND RESOURCE UTILIZATION BY ASIAN ELEPHANT (Elephas

maximus Linnaeus) IN NILGIRI BIOSPHERE RESERVE, SOUTHERN INDIA. 1998.

83. Gubbi S, Swaminath M, Poornesha H, Bhat R, Raghunath R. An elephantine challenge: Human-elephant conflict distribution in the largest Asian elephant population, southern India. Biodiversity and Conservation. 2014;23:633-647. https://doi.org/10.1007/s10531-014-0621-x.

84. Lisa R, Jay H. Some Observations Regarding Allomaternal Caretaking among Captive Asian Elephants (*Elephas maximus*). Journal of Mammalogy. 1987;68(2):438-442. https://doi.org/10.2307/1381494.

85. Lahdenperä M, Mar KU, Lummaa V. Nearby grandmother enhances calf survival and reproduction in Asian elephants. Scientific Reports. 2016;6:27213. https://doi.org/10.1038/srep27213. PMID: 27282468.

86. Kurt F, Hartl G, Tiedemann R. Tuskless bulls in Asian elephant Elephas maximus . History and population genetics of a man-made phenomenon. Acta Theriologica. 1995;40:125-143. https://doi.org/10.4098/AT.arch.95-51.

87. Paulraj S, Subramanian C. Skewed sex ratio in elephant population - How it may affect the demography and population growth? A case study in Mudumalai Wildlife Sanctuary and National Park, Tamil Nadu, Southern India. Indian Forester. 2000;126:1046-1053.

88. Silva Sd, Webber CE, Weerathunga US, Pushpakumara TV, Weerakoon DK, Wittemyer G. Demographic Variables for Wild Asian Elephants Using Longitudinal Observations. Plos One. 2013;8(12):e82788. https://doi.org/10.1371/journal.pone.0082788. PMID: 24376581.

89. Fernando P, Wikramanayake ED, Janaka HK, Jayasinghe LKA, Gunawardena M, Kotagama SW, et al. Ranging behavior of the Asian elephant in Sri Lanka. Mammalian Biology. 2008;73(1):2-13. https://doi.org/10.1016/j.mambio.2007.07.007.

90. Srinivasaiah N, Kumar V, Vaidyanathan S, Sukumar R, Sinha A. All-Male Groups in Asian Elephants: A Novel, Adaptive Social Strategy in Increasingly Anthropogenic Landscapes of Southern India. Scientific Reports. 2019;9:8678. https://doi.org/10.1038/s41598-019-45130-1. PMID: 31273235.

91. Pokharel SS, Seshagiri PB, Sukumar R. Assessment of season-dependent body condition scores in relation to faecal glucocorticoid metabolites in free-ranging Asian elephants. Conservation Physiology. 2017;5(1):cox039. https://doi.org/10.1093/conphys/cox039. PMID: 28721215.

92. Guozheng S, Guangyuan Z, Zicheng Y, Mengjun W. Research progress and direction of human elephant conflict mitigation projects. Forestry Construction. 2019;6:5-9.

93. Sheng-dong Y, Yun D, Ai-dong L, Mang-cai W, Bin W, De Z, et al. A preliminary study of wireless infrared camera technology in alleviating the human-elephant conflict. Forestry Construction. 2019;(6):61-66.

94. Zhi-hong W, Heng-zhen Z, Wen-lan Z, Xuan Z, Wen-bian Y, Zheng-ling L, et al. An analysis of population dynamic on the Xishuangbanna Menghai-Pu'er Lancang Asian elephant population. Forestry Construction. 2019;(6):79-84.

95. xian-ming G, lan-xin W. Thoughts on the construction of Asian elephant food source base in Xishuangbanna. Forestry Construction. 2019;(6):30-33.

Supporting information

S1 ~~Table~~Dataset. A simplified database of Asian elephant in NNNR

~~S2~~ S1 Table. Historical elephant population in NNNR estimated by different methods

- Dear reviewers and editor,

First, we would like to thank the reviewers and the editors for your time, comments and expertise. Your exhaustive and positive comments and suggestions makes our paper more rigorous and logistic. The following letter gives our responses to the reviewer's comments as requested in your letter 22 June 2020.

General Comments of Reviewer #1:

Response: Thank you for your comments and suggestions. Methods has been re-written as to make the Body Condition Scoring (BCS) section clearer and more comprehensible. This paper has been checked by professional editors, both native speakers of English.

Specific Comments of Reviewer #1:

Comment 1: On line 220, the buffer zone is said to be 5km around the outermost camera sites. There was no citation here, so what was the justification for using 5km and not a different value?

Response: Considering the small size of the elephant distribution, with the east and west boundary being no wider than 10 km, we therefore defined our study area as extending 5 km beyond all camera stations, corresponding to an area of 255.8 km^2. We cited one reference. Please go to P12, L226-228.

Comment 2: Lines 245-246 Please list all the model covariates and provide some description e.g. was age a categorical factor in the models? Was BCS considered as a continuous variable for the purposes of getting the correlation coefficients?

Response: The covariates include sex, age and whether the elephant was solitary or in the herd. BCS was not a continuous variable. Please go to P13, L260-262.

Comment 3: It is unclear how many photographs were used to calculate the scores for each elephant. If multiple, were they all from around the same time and consistent? If the score is an average or not needs to be stated.

Response: Five of the clearest images for each physical characteristic were selected to assess the body condition. Thirty images were used to calculate the comprehensive scores for each individual. Please go to P13, L244-246.

Comment 4: Lines 285-287 Asian elephants may allolactate (Rapaport, L. & Haight, J. 1987 Some observations regarding allomaternal caretaking among captive Asian elephants (Elephas maximus). J. Mammal. 68, 438–442), and there may be other forms of allomothering too (Lahdenperä M., Mar, K.U., Lummaa V. 2016 Nearby grandmother enhances calf survival and reproduction in Asian elephants. Scientific Reports 6: 27213; Lynch E. C., Lummaa, V., Htut W., Lahdenperä M. 2019 Evolutionary significance of maternal kinship in a long-lived mammal. Phil. Trans. R. Soc B. 374: 20180067) – I'm not sure that any relationship can be considered 'definite'.

Response: Yes, we lost sight of that. Only the DNA methods can tell us the true relationship among individuals in this population. The word "definitely" has been deleted. We discussed this situation in the Discussion section. P16, L297-301; P20, L370-379.

General Comments of Reviewer #2:

Response: Thank you for your exhaustive and positive comments and suggestions. Some missing information of Methods has been re-written as to make this section clearer and more comprehensible. We apologize for the previous manuscript as it was very unclear in several sections and there was overspeculation in the Discussion and Conservation Recommendations sections. A major revision of this manuscript has been accomplished and many unnecessary parts have been deleted. In the Result and Discussion sections, we combined the population size and density part to make the paper more coherent and logical. And in the Conservation Recommendations section, we totally replaced the previous version by a simpler and more summarized one. This paper has been checked by professional editors, both native speakers of English.

Specific Comments of Reviewer #2:

Comment 1: L. 34: Insert BCS here for the first time.

Response: We have inserted BCS. This sentence has been changed to "The health condition of this elephant population was assessed by using an 11-points scale of Body Condition Scoring (BCS) method which showed that this population had a medium body condition with an average of 5.75 (n=12, range 2-9)." Please go to P2, L33-35.

Comment 2: The fact that individuals had such low BCS should be highlighted in the abstract (if space allows).

Response: We have rewritten this sentence. Please see the response to comment 1 or P2, L33-35.

Comment 3: L. 44: End the sentence at "dispersal" and start a new sentence about elephants as umbrella species.

Response: This sentence has been changed as you recommended. Please go to P3, L42-46.

Comment 4: L.49: When you mention that the species is listed as Endangered in IUCN Red List, and then that its population is decreasing, you make a tautological argument. Any endangered population is decreasing. You should rather change the sentence and say that however important it is for conservation (as an umbrella species and an ecosystem engineer), there is a problem with the species, which population is decreasing, hence justifying its endangered status.

Response: Yes, we believe that this is more logical. This sentence has been changed to make it simpler. "It is a Class I Key Protected Species in China and listed as an Endangered under the IUCN Red List.". Please go to P3, L48-49.

Comment 5: L.77: Puma is not a good example, as they do not have spots or stripes. I would also keep this list more general, citing genera that have spots or stripes, such as tigers, leopards, giraffes, etc.

Response: Puma has been replaced by leopard cats since they have stripes on their bodies. Please go to P4, L76.

Comment 6: L.88: There is a jump between the two sentences (no logical sequence). Maybe you could close the previous argument by saying that for NNNR there are not

such data available. Then you could start a new paragraph on this isolated population, its genetic status, etc.

Response: A new paragraph has been created. Please go to P5, L99.

Comment 7: L.99-108: This new paragraph also makes a jump. You should place it in the series of paragraphs describing how trap cameras (and photographs more broadly) are another way that tool can be useful in conservation and management.

Response: We moved the BCS paragraph forward placing it behind the paragraph that introduces individual identification by camera traps. Please go to P4-5, L81-92.

Comment 8: L.104-105: It is unclear why BCS is most meaningful "especially [in] small isolated populations". Explain it or delete it.

Response: We deleted the word "small". Now the sentence looks like "While BCS is individual-based, it is most meaningful when applied to a population, as an early indicator of the impact of management actions on the average health condition of a population.". Please go to P5, L87-90.

Comment 9: L. 107-108. Delete this sentence. It is of very little relevance for a journal with a broad, worldwide audience.

Response: This sentence has been deleted. Please go to P5, L92.

Comment 10: L.109-116: There is one more reason to move the previous paragraph upward: this paragraph is connected to the one preceding the previous one (i.e., after the paragraph l. 82-98).

Response: That is correct. After moving the previous paragraph upward, this paragraph is now well connected to the previous and following paragraphs. Please go to P6, L109-115.

Comment 11: L. 114-115: "for the development of a successful project focusing on the introduction of individual elephants from other populations". That is the kind of sentence that does not serve you well. You talk about introducing individuals, but

you have provided no evidence that the conditions to ensure the survival of introduced animals are met in the area. In many areas, habitat (quantity or quality) or negative relationships with humans do not ensure survival, making the prospect of reintroduction a loosing strategy.

Response: We did not obtain enough evidence to support the introduction of individuals as a successful plan for the survival of this small population. Thus, we deleted any mention about the introduction of elephants throughout the manuscript. Please go to P6, L112-115 and Conservation Recommendation section.

Comment 12: L. 120: I would change "to analyze the factors" to reflect that it is indeed a discussion of some factors that might have affected the population that you will present.

Response: This sentence has been changed to "We sought to understand which factors might have affected this populations size in recent years in order to develop targeted and effective protection strategies.". Please go to P6, L118-119.

Comment 13: L. 122: Why in China only and not in other parts of the species distribution?

Response: We have deleted "in China". The new sentence is "Furthermore our approaches can be applied to other elephant populations with further implications for global Asian elephant conservation." Please go to P6, 120-121.

Comment 14: L. 134: Give properly the geographical coordinates (including N to the latitude, and W to the longitude).

Response: The sentence had been changed to "It occupies 70.82 km^2 at latitude 23°13'- 23°19'N, longitude 98°54'- 99°05'E, and the altitude ranges from 510 m to 1747 m.". P7, L131-133.

Comment 15: L. 137: Please give a proper annual precipitation and temperature (a single value), or explain the range you provide.

Response: Mean annual precipitation is 351.3 mm in the dry season and 1983.3 mm in the rainy season, while temperature varies at different altitudes. The average monthly

temperature range is hottest in July, varying between 12.3-25.5°C and it is coldest in January with values between 2.8-14.3 ℃. (Reference: NGH scientific report. No single value for annual temperature, just range value.) Please go to P7, L134-137.

Comment 16: L. 138: Why natural in “natural tropical forest”?

Response: It should be a tropical forest. We made a little mistake when we read the corresponding reference. Please go to P7, L138.

Comment 17: L.140: By evergreen vegetation do you mean evergreen broadleaf species, in opposition to coniferous species?

Response: Yes, it is evergreen broadleaf species. We have added “broadleaf” in the manuscript. Please go to P7, L141.

Comment 18: L. 148: Use a better title, such as survey design or camera trap set-up.

Response: The title has been changed to “Camera trap set-up”. Please go to P7, L148.

Comment 19: L. 151: “along the animal trails with forest gaps where there were signs of elephants”. Unclear. Did you set up the cameras in forest gaps that were crossed by elephant trails? If so replace “with” by “in”. In any case, review this.

Response: Actually, we set up the cameras in trails and forest gaps, but the gaps were often located in trails. So, here we replace “with” by “and” to make it clearer. Please go to P8, L155-157.

Comment 20: L. 152: “as well as other activities”. You cannot remain vague here; mention them all.

Response: The elephant signs included lying sites, foraging sites, footprints, dungs and urine marks. Please go to P8, L155-157.

Comment 21: L. 152: Explain why you avoided certain areas. Was it because elephants do not use them, or because you had concerns for the safety of people in the field?

Response: We avoided the northwestern part of the reserve as the topography is tough, with steep cliffs of up to 1747 meters, and historically, there have not been any records of elephants in this area. Please go to P8, L159-162.

Comment 22: L. 149-164: In this section, there is information missing. As far as I understand you had one camera per site, but one sentence mentions that "at each site, camera traps were operated...", which suggests there were several cameras at each site. Also, there is information missing regarding the number of sites or cameras, the minimum distance that could separate two sites (because there could be spatial autocorrelation in your data). Finally, you should mention the camera model(s) you used in your study. Elephants are not small nor super fast, so trigger time should not be an issue, but still, getting information on the material used is necessary for replication purposes.

Response: A total of 52 cameras at 52 stations or sites (P8, L150-151). We set one camera trap at each camera site, but the number of camera sites varied in each grid. More than one camera was installed in the grids that contained lots of elephant signs (P8, L170-171), and the minimum distance between two cameras was 200 meters (P8, L158-159). The camera model was Ltl Acorn 6210 (P8, L150) and we programmed the camera to take sequential images consisting of three photographs plus one 12 second video (P8, L168-170).

Comment 23: L. 166-169: The whole sentence needs to be rewritten for better clarity. After reading it, I am left with doubt about what constituted an independent event. I think I understood that an independent event could include several photographs of various individuals, with at least 5 h between such photographic sequences to make it temporally independent, but it is far from clear.

Response: We have provided a more detailed explanation about independent events than in the previous manuscript version. Please see P9, L175-181.

Comment 24: L. 177: Did you determine the sex in juveniles and calves? If so, how did you do that?

Response: Yes, we made it clearer in Table 1. Please see P10, L199.

Comment 25: L. 185-187: How do you know they did not change? Ear can be teared at any moment; a tusk could be broken digging up... maybe it is the way it is written that makes it not very convincing. If you have an elephant with a particular tear in the ear, it is easy to "recapture" it, but if there was no marking, I am not sure how this could be reliable. I think a better explanation would help.

Response: Yes, that is a better explanation that would be more convincing. Morphological characters including facial bones, ear, tusk, back and tail hair, old scars and injuries, as well as physical characteristics related to sex and age classes, confirmed the reliability of identification results. Please see P10, L196-198.

Comment 26: Table 1: 1) Regarding sexing, as mentioned earlier I think male and female should be labeled properly adult female and adult male, as the diagnostic characteristics are found in adult (and possibly subadult) individuals only. 2) “Sunken prominence”. How can a prominence be sunken? 3) Your classes for tusk length are ok, but I am not ok with the fact that you do not explain how you classified them in each of these categories. I am sure you had a criteria to decide (such as tusk length relative to the trunk).

Response: 1): We had rearranged sex categories as adult female, adult male, subadult male, juvenile female, juvenile male and calf male. 2): It was a description mistake. The sentence has been changed to “Extent of depression”. 3): Yes, we have a criterion that uses trunk as a reference to classify the tusk length. For more details please go to Table 1 on P10, L199.

Comment 27: L. 197: “The movement pattern of individuals activity center in study area”. This is unclear. Do you mean “The movement pattern of individual activity centers in the study area”, i.e., the movement pattern of the activity centers of a given individual evaluated at different times (sessions)? Sorry, but the devil is in the details!

Response: Yes, the model can evaluate the movement pattern according to the capture history. We missed the time limitation. The sentence has been changed to “The state model describes statistically the mechanisms determining the distribution of the animals in the study area, while the observation model quantifies the probabilities of detection or capture, given the searched region, their locations and characteristics at different sessions”. Please go to P11, L205-209.

Comment 28: L. 198-200: "the probability of detecting an individual at a particular detector (…) to the distance of the detector" makes no sense. Please rewrite.

Response: The sentence has been rewritten combining it with the forward sentence. Please see the response of comment 27 or onP11, L205-209.

Comment 29: L. 206 and elsewhere in this section: Use trap or detector, or explain that detectors are traps, but remain consistent throughout your explanations.

Response: All the words corresponding to “detectors” were replaced by “traps”. Please go to P11, L212; L214; L217.

Comment 30: L. 217: Again, there is a problem with the phrasing and it is unclear what you mean.

Response: This sentence has been deleted. Please go to L12, L224.

Comment 31: L. 219-221: The whole sentence needs to be rephrased so that the reader can understand exactly what you did. Also “from the outermost x and y coordinates of the camera sites” is very unclear. A camera site is not a discrete point in space???

Response: The sentence has been changed to “Considering the small size of the elephant distribution, with the east and west boundary being no wider than 10 km, we therefore defined our study area as extending 5 km beyond all camera stations, corresponding to an area of 255.8 km^2.”. Please go to P12, L226-228.

Comment 32: L. 224: Again, this is unclear. Is the file named "Capture histories"? Please check the sentence.

Response: Yes, the file is named “capture histories”. The sentence has been changed. Please go to P12, L231.

Comment 33: L. 225: Is it "occasion" (you haven't defined it previously) or "event"? Also, earlier you defined individual events as being separated by at least 5 hours. This means that several individual events could occur on a same day. How can your data base account for that if your time unit is "day"?

Response: “Occasion” was defined as each event, as well as the “sequence”. The cameras captured several events, accounting for different occasions. Please go to P12, L232.

Comment 34: L. 227: “whole data as five different sessions”. Again, it is unclear. Do you mean you divided your 4-month sampling period in 5 (i.e. each session would last 24 days)?

Response: Five different sessions, corresponding to each of the months and one corresponding to the whole survey period, were used to run the model to see the estimation accuracy under different detection efforts. P12, L233-236.

Comment 35: L. 236-244: The first of these five sentences is very unclear. Further, if you followed the method developed by 49 or 50, just cite them, and mention that the part on which the scoring focuses are the ribs, shoulders, etc. and say why. Usually it is because these parts can accumulate fat.

Response: We added more information to describe the reasons we choose ribs, shoulders and other body parts as scoring criterion. Please go to P13, L246-252.

Comment 36: L. 244: Before mentioning which software you used to perform your analyses, say what kind of analysis you performed.

Response: This sentence has been deleted. Please go to P13, L260.

Comment 37: L. 244-245: Which type of correlation coefficient?

Response: It is Pearson Correlation Coefficient. P13, L260.

Comment 38: L. 245: “ratios of various covariates”: why ratios? Age is not a ratio, nor is sex or the solitary vs group status.

Response: The word “ratio” has been deleted. Please go to P13, L260-262.

Comment 39: L. 246: “whether an individual was present or not in the herd”. Here you lost me... do you mean you checked the correlation between BCS and whether an individual was solitary or in herd? (note that this is not what you have written; you have written “the herd”)

Response: Yes, we wanted to divide individual elephants into those that were solitary and those that were part of a herd to see if this factor could affect the BCS. Please go to P13, L260-262.

Comment 40: Table 2: 1) Revise the title of this table. 2) Characters is incorrect here and in several places in the manuscript. The correct word here is characteristics.

Response: 1): The title has been changed to “Table 2. Characteristics used to assess the body condition in Asian elephant, NNNR”. 2): All the words “characters” have been changed to “characteristics”. Please go to P13, L263 to see Table 2.

Comment 41: L. 250-253: This is much better suited in Methods. I kept wondering how you set up your cameras, how many, and here is the answer. Yours results should start with what you obtained, not what you did.

Response: Yes, these sentences presented in the Methods section were more logical. These sentences have been moved to their current location. P7-8, L149-152.

Comment 42: L. 254: You never spoke of video before! Fix that in Methods or delete here if they were not used.

Response: We apologize for this mistake. We programmed the camera to take three pictures and a 12 second video. We replaced the words “picture” and “video” by “image” in the Methods section. Please go to P8, L168-170.

Comment 43: L. 261-264: You should revise that sentence. As it is, there is not much difference between the individual that was seen the least and the others. Rather give the mean and the range.

Response: We strongly agree with your suggestions. Currently, there is a brief comment about the capture histories of individual and herds. Please go to P14, L271-274.

Comment 44: Fig. 2: This title does not correspond to what you describe in the text (i.e., the 12 identified individuals). I think it should say something like "Asian elephants individually identified in NNNR park, China". Also, note that the way you cited the figure, one would expect to see the 12 individuals identified. Here you show only half of them.

Response: We strongly agree with your suggestions and the title of "Fig 2" was changed as you recommended. The figure was re-edited to combine all 12 individuals in one picture, and we used black squares to emphasize each individual's prominent characteristic so as to make it clearer for readers. Please go to P15, L285-287 and the file named "Fig2".

Comment 45: Table 3: 1) Put "No" in the column for Tusk for each female. This is all the more important that you later discuss the fact that you might have misclassified some individuals. 2) Put "Herd" in each file for individuals AE01 to AE08.

Response: The changes you proposed have been achieved in Table 3 of the new manuscript. Please go to P14, L275.

Comment 46: L. 272-279: This is interesting, but falls out of the scope of the section (Individual identification).

Response: Yes, we strongly agree with you and thus, this paragraph has been deleted after careful consideration.

Comment 47: L. 283-284: This sentence is not built correctly, and it is unclear what the 1:1 ratio stands for (is it a sex ratio too?). Also, as I mentioned previously, the way you mentioned the criteria for sexing seemed to refer to sexually mature

individuals only, so I wonder how you determined sex in juvenile individuals, if the 1:1 ratio is indeed a sex ratio. This needs to be clarified in the methods too!

Response: We wrote this sentence incorrectly. The 1:1 ratio stands for juvenile individuals as we identified two juvenile males and two juvenile females. But we failed to make it clear in the Methods section. Please go to P16, L290-296.

Comment 48: L. 285: What are "containment relationship family units"?

Response: Since we wrote this sentence using the wrong words, it has been deleted. Please go to P16, L297.

Comment 49: Table 4: In the title you mention five sessions, but in the table there are only four sessions, one for each sampled month.

Response: We apologize for the way that it was written. First, we used single month as a single session, and then we used the whole months data as the fifth session. Please see more details in comment 34 or go to P12, L233-236.

Comment 50: L. 306-307: This is the definition of the median!!!!! Please, take this out, or give the mean and here you could assess how skewed the distribution is.

Response: This sentence has been deleted. Please go to P16, L304.

Comment 51: L. 311-312: Say which sex had the highest BCI. Rephrase "solitary or not" (being in a herd or solitary), and explain which status was associated with having a lower or higher BCS.

Response: This information has been combined. Please go to P16-17, L307-313.

Comment 52: Figure 3: The figure does not help much, and a table would probably be more efficient here.

Response: We think a figure would be clearer and more visible than a table. We have improved the figure by changing the order of individuals by placing individuals of the same sex together and ranking them by scores. Please go to the file named "Fig3".

Comment 53: Table 5: For the adult individual that died in 2003, how can you know "it died for its ivory"? Was it killed (with bullets inside the body)? You could find an individual with the tusks removed post-mortem.

Response: We read these two references again and deleted one reference which provided us with incomplete information about an adult male that had died for its ivory without any citation nor detailed explanation. So, the description of "the adult male died for its ivory" has been deleted. Please go to Table 5 on P18, L338.

Comment 54: L. 337-341: This is definitely not of interest for the readership of PLoS One.

Response: These sentences have been deleted. Please go to P19, L364.

Comment 55: L. 343: Use a reference to justify the link you make between the population structure and its future.

Response: Four references have been added. Please go to P17, L323-327.

Comment 56: L. 343-345: Rephrase that sentence (self contradiction the way it is written), and note that this sentence contradicts the previous one (speaks of a possibly stable population, when earlier you say that it indicates a decline).

Response: This sentence has been rephrased. Please go to P20, L366-369.

Comment 57: L. 345-346: This sentence comes out of the blue. Link it to an argument. You can actually use it to finish the following sentence with this argument of inbreeding depression in small populations.

Response: This sentence has been combined. Please go to comment 56 or P20, L366-369.

Comment 58: L. 351-352: Again, a sentence out of the blue, which has no direct link with your results. What argument do you want to discuss in this paragraph? Start with that, and then develop the argument.

Response: We strongly agree with your comments. This sentence has been changed to "There were two tuskless juveniles that had similar body height as the juvenile males identified by their apparent tusks and these were identified as females. However, it should be noted

that males in some populations tend to be tuskless for survival due to the intensive historical poaching pressure. The high proportion of makhnas in NNNR identified from this investigation could indicate that these juveniles that were identified as females might in fact be males." Please go to P20, L380-385.

Comment 59: L. 355: Why do you say even if it was 1:1.5?

Response: This mistake has been corrected. Please go to P16, L290-296.

Comment 60: L. 359: Ref. 72 is not about balance in sex ratio in Asian elephant, it is about a sexing method. Make sure it is appropriate to cite it.

Response: These sentences and reference 72 have all been deleted. Please go to P17, L323.

Comment 61: L. 360-361: In this sentence you refer to tuskless males, but who identified these tuskless males? If it is another study, cite it.

Response: We identified these tuskless males. Approaches were displayed in Table 1 section. Please go to Table 1.

Comment 62: L. 366: "116 individual deaths (…) might account for tuskless males". It is unclear here what you mean.

Response: High poaching pressure could lead to the predominance of tuskless males. Since currently there is a high proportion of tuskless males in this population, we had assumed that the reason for this situation was historical poaching. But since we don't have data to support this assumption, this sentence has been deleted. Please go to P20, L383-385.

Comment 63: L. 360-370: I find all this difficult to follow because of some problems of clarity, but above all, you discuss a point when you recognize that you do not know for sure if the tuskless individuals you identified were males or females!

Response: Yes. We judged these juveniles as females under the condition that they have similar body height as juvenile males who had apparent tusks. And at the same time, we discussed that since tuskless males are common in this population, it would be helpful

to conduct genetic studies on this population that can confirm the sex of these two juveniles. Please go to P20, L380-387.

Comment 64: L. 379: Add at the end of that sentence that this is especially true due to birth intervals.

Response: The sentence has been changed to "In either case, the scarcity of fertile females would be the primary constraint on population growth due to their long birth intervals." P21, L394-395.

Comment 65: L. 398-399: OK, but the way you present it by comparing to India leaves the reader with the idea that these authors (ref 79) did the study over a short period of time. Was this the case, if not, why discuss this and in that way? This is very troubling.

Response: It has been deleted. Please go to P19, L344.

Comment 66: L. 401: This value (800 m distance) makes sense only if we have an idea of elephant activity areas and movements.

Response: Here, we cited two references that pointed out that this value was smaller than elephant daily movement distance. Please go to P19, L346-348.

Comment 67: L. 404: Not real, but actual. It is still based on what you captured on photographs.

Response: Yes, the result should be actual value. The changes have been made in the new manuscript. Please go to P19, L350-351.

Comment 68: L. 405: estimated by whom? Because your sentences are not well linked, it is hard to follow.

Response: We estimated it. We found the reference by Sukumar (2003) who gave an assumption that a population density of 0.1-0.3 elephant/km^2 was normal. So, we used this assumption to estimate there should be 20 individuals in NNNR (71 km^2). In the new manuscript, we deleted this hypothesis. Please go to P17, L329-331.

Comment 69: L. 408: About carrying capacity: well, you said before that 40 years ago the estimate was of 20 individuals, so at 12 it is obvious that you are below (even if we don't know if 20 was at carrying capacity or not).

Response: The use of the word "carrying capacity" made the sentence less rigorous. This word has been deleted. Please go to P17, L327-331.

Comment 70: L. 394-410: All this paragraph is convoluted. Why make weird assumptions (two sentences above) if you know that the population has already been that size.

Response: The whole paragraph has been rewritten. Thus, we combined the density section with population size section to make it simpler. Please go to the Population size and density part of the Discussion section. P18, L323-337.

Comment 71: L. 415-418: Rewrite these two sentences, and put what is a result in the corresponding section.

Response: These two sentences have been placed in the corresponding section. Please go to P16, L307-308.

Comment 72: L. 422-424: So what? Here it is not the males venturing into human areas, but rather humans venturing into elephant territories. It means that males are more tolerant to humans, and has nothing to do with the quest for better food as when they venture in crop areas. You need to thread your arguments better.

Response: Thank you for your recommendation. This part has been rewritten. Please see P21, L400-401.

Comment 73: L. 424-425: What you mention is well known, but you said that the other females were not lactating, so how does your argument hold?

Response: The only three adult females in this population were all considerd to be potentially productive, and the body health condition of all of them was considered to be average or even in poor health condition. Our data showed that the individual AE06 who was nursing an offspring had the lowest BCS in this population. Therefore, we mentioned

additional nutritional stress from lactation for AE06 could have resulted in an even poorer body condition. Please go to P21, L404-409.

Comment 74: L. 426: What does "reasonable" mean here? Also, you cannot consider the median or the mean as a standard. You may have a very healthy or very unhealthy population and the mean (or the median) can vary quite a lot.

Response: This sentence has been deleted. We had classified the 11-point scale with three ranks: poor, medium and good. Please go to P13, L259-260.

Comment 75: L. 427-429: Yes, this is true, but this would skew evaluation towards lower BCS for adults, compared to juveniles.... and you found the opposite.

Response: Yes, that is correct. Maybe the adults have lower BCS than juveniles assuming that we had a large population like previous studies. The truth is the population in NNNR was extremely small, and the fact is that we found that the adults had higher BCS than the juveniles. These sentences have been deleted since we do not want to confuse readers. Please go to P21, L406.

Comment 76: L. 431-438: All this is very speculative, and not very convincing. If the animals raid cultures during the rain season it might be for different reasons, and if they don't during the dry season maybe is it because they find enough food within the reserve. You have to be very careful: you do not have enough information to really discuss all that without speculating.

Response: We strongly agree with your comments. Please go to P22, L410-422.

Comment 77: L. 438-441: Yes, but you have nothing on that: your data is from one season, in one particular year... not much to inform conservation managers.

Response: This sentence has been deleted. Please go to P22, L419-422.

Comment 78: L. 445-446: Take out the conflict part. It is not part of this argument and just distract the reader.

Response: This conflict part has been deleted. Please go to P22, L423-427.

Comment 79: L. 448: “obesity level”. Surely you want to say something else! In nature animals are not obese (or very rarely), only in zoos.

Response: We wanted to say “health condition”. Please go to P23, L432.

Comment 80: L. 442-451: All this is well beyond what your data can tell.

Response: We aimed to extend our research to other elephant populations. Although more and more studies have been conducted in China, the study of the BCS has been neglected. Thus, we wanted to draw attention on this kind research for Chinese researchers and conservationists. Please go to P22-23, L423-435.

Comment 81: L. 454: You keep mentioning the question of evenness in the sex ratio, but there is no foundation in your manuscript to justify why an even structure would be better.

Response: We have deleted this part. Please go to P23, L436.

Comment 82: L. 472-474: About reintroduction. Your study does not confirm that reintroduction is the solution for this population!!! In no way. See my previous comment on this in the introduction.

Response: All the information related to reintroduction has been deleted. Please go to comment 11 and P6, L112-115 or Conservation Recommendation section.

Comment 83: L. 478: Why mention restoration here? You have never mentioned that the habitat was either destroyed or degraded within the reserve. If it is the case, you should have better described your study area.

Response: The word “restoration” has been deleted. Please go to Conservation Recommendation section on P436.

Comment 84: L. 485-486: About reintroduction to reduce inbreeding. You have no data on that, do not speculate, especially when recommending such drastic approach as translocation, which comes with lots of cons.

Response: All the information related to reintroduction has been deleted. Please go to comment 11 and P6, L112-115 or Conservation Recommendation section.

Comment 85: L. 489: Please restrain from mentioning reintroduction. Just managing the population to ensure its viability (if it is possible), would be enough! As I mentioned in the comments to the introduction, reintroduction is rarely a good solution, as the problem is usually with the habitat (quality or quantity) or with the negative interactions with humans.

Response: All the information related to reintroduction has been deleted. Please go to comment 11 and P6, L112-115 or Conservation Recommendation section.

Comment 86: Figure 3: 1) Eliminate “points” (after Presence, Absence, and Village). 2) The border with Myanmar and the limits of the reserve are similar (same width, same colour). 3) It is har to say where the experimental zone is on the map. It would be better to use the light shade to shade the area.

Response: All the suggestions that you proposed have been taken into consideration. See more details in file named “Fig 1”.
