# Supplementary material for: Assessing population structure and body condition to inform conservation strategies for a small isolated Asian elephant (Elephas maximus) population in southwest China
Source: PLoS One. 2021 Mar 9;16(3):e0248210. doi: 10.1371/journal.pone.0248210 (PMC7942997; doi:10.1371/journal.pone.0248210)
Supplement: Attachment — Submitted filename: PONE-D-20-11590_R2_reviewer revSC.pdf [file pone.0248210.s007.pdf]

PLOS ONE

Population structure and body condition assessment to inform conservation strategies for a small isolated Asian elephant population in southwest China

--Manuscript Draft--

**Manuscript Number:**	PONE-D-20-11590R2
**Article Type:**	Research Article
**Full Title:**	Population structure and body condition assessment to inform conservation strategies for a small isolated Asian elephant population in southwest China
**Short Title:**	Population structure and body condition of Asian elephant in southwest China
**Corresponding Author:**	Kun Shi<br>Beijing Forestry University<br>Beijing, CHINA
**Keywords:**	Asian elephant; Population size; population density; population structure; camera traps; body condition scoring
**Abstract:**	The Asian elephant (Elephas maximus) population in Nangunhe National Nature Reserve represents a unique evolutionary branch ~~in China, which~~ has been isolated for more than twenty years from other neighboring populations in Myanmar. The scarcity of information on population structure, sex ratio and body condition makes it difficult to develop effective conservation measures for this elephant population. Twelve individuals were identified from 3,860 valid elephant images obtained from February to June ~~of~~ 2018 (5,942 sampling effort nights) at 52 camera sites. Three adult females, three adult males, one subadult male, two juvenile females, two juvenile males and one calf male were identified. The ratio of adult females to adult males was 1:1, and the ratio of reproductive ability was 1:0.67, indicating the scarcity of reproductive females as an important limiting factor to population growth. A population density of 5.32 ± 1.56 elephants/100 km ^{2} was estimated using Spatially Explicit Capture Recapture (SECR) models. The health condition of this elephant population was assessed by using an 11-points scale of Body Condition Scoring (BCS), which showed that this population had a medium body condition with an average of 5.75 (n=12, range 2-9). This study finds that this isolated population has an extremely small size and an inver[illegible]pyramid age structure and therefore the population is at high risk of extinction. As such, this population should be of top conservation priority among the few remnant populations in China. The database established in this study can provide researchers with valuable information to set up e[illegible]nt conservation schemes.
**Order of Authors:**	Kun Shi
	Yakuan Sun
	Ying Chen
	Juan José Díaz-Sacco
**Response to Reviewers:**	General Comments of Reviewer #1:<br>Response: Thanks for your recognition of our new version manuscript. Here were responses of your two small suggestions.<br>Comment 1: Line 323, 'as in' should be 'than in'<br>Response: The word has been replaced by the one your recommendation. Please go to P16, L313.<br>Comment 2: line 401-402 - You may want to include a short explanation for why larger home ranges and tolerance of disturbance could be linked to higher BCS. Additionally, the sentence starting 'Which means' should instead start 'Tolerance means'<br>Response: Large home ranges and tolerance of disturbance means the male elephants obtain more food resources, which contribute a good body condition in turn. The new sentence has been changed like “This may be because adult males are known to have larger home ranges than adult females, and are more tolerant to human disturbance: that means they can explore more frequently areas near the reserve boundary to obtain more food resources, where intensive anthropogenic activities like hunting, grazing, fishing and collecting have also been recorded by cameras.”<br>General Comments of Reviewer #2:

Powered by Editorial Manager® and ProduXion Manager® from Aries Systems Corporation

	Response: Thank you for your exhaustive and positive comments and suggestions. We made huge changes on Discussion to make it clearer and logistical. In Conservation Recommendations part, we only used two sentences to describe the importance of long-term monitoring of infrared cameras. Then focus on the suggestions that improving food resources to enhance the elephant body condition. This paper has been checked by professional editors, both native speakers of English.<br>Comment 1: On line 93-95, This sentence should go Line 80, to finish the paragraph on photo ID.<br>Response: This sentence had moved to Line 80 in the new version of manuscript. Please go to P4, L80-82.<br>Comment 2: On the line 99-115, These two paragraphs should go right after the first one, when you describe elephant populations in China. Otherwise, these paragraphs do not connect well with the previous ones.<br>Response: We deleted some sentences in these two paragraphs and have rewritten remaining sentences. We thought these could be more logical and precise than before. Please go to P5, L95-105.<br>Comment 3：On the line 200, Maybe the way you present the table is not optimal, because when you describe the categories in SEX, you include already both sex and age. So maybe you should start describing AGE, and then SEX.<br>Response: We adapted your opinion by describing AGE first and then describing SEX which was simply categorized to female and male. Please go to P9-10, see Table 1.<br>Comment 4: On Table 1: Ok, so it is obvious that adult and subadult males that are tuskless may be impossible to distinguish; maybe add "and no penis", to distinguish them from their male counterparts; Same, you should add the testicles/penis part, but here, how do you distinguish juvenile from subadult males<br>Response: We have simplified the category of SEX by just describing female as “Without obvious tusks but with prominent breasts, belly swelling or the presence of nursing offspring” and male as “With visible testicles or penis, and/or obvious tusks”. See Table 1.<br>Comment 5: On the line 210, It should be replaced by "each", even when it is clear that you mean "each and every"<br>Response: It should be “each” in this situation. Please go to P11, L222.<br>Comment 6：On the line 232, Not too clear to me. You mean to say "i.e."?<br>Response: This sentence has been changed into “The file named “capture histories” contained data on individual identified each sampling occasion from the specific detector (camera).” Please go to P11, L221-222.<br>Comment 7: On the line 323-327, This sentence is unclear. What do you want to convey here? Do you want to say that different methods lead to different results inveitably, or do you just want to point out that historically the size of the population was estimated at about 20 individuals?I think it is the second case, but in any case by straightforward.<br>Response: This sentence has been changed to make it simpler. “Previous studies have estimated this population to be around 20 individuals. Although population estimation using different methods could generate bias in direct comparison, research has shown that this elephant population has remained small during the last few decades.” Please go to P16-17, L313-317.<br>Comment 8: On the line 327-330, The logics here remains unclear; with the information provided it is not obvious why being in the same part of the park means that there should be more elephants. Maybe habitat quality is not that good anymore (in fact, the fact that females were not in good body condition might indicate something like that)... or it might be that the highest ever recorded population in the park was an artefact due to loss of habitat in the surrounding (and then a packing effect).<br>Response: We did not do any analysis in habitat quality estimation, so it is surprising to readers see the point suddenly. This sentence has been deleted.<br>Comment 9: On the line 335, You have four elephants dead since 2003... don't calculate statistics over that! Just say that 3 of the 4 elephants found dead since 2003 have been immature individuals.<br>Response: This sentence has been changed to “Additionally, 3 of 4 elephants found dead since 2003 have been immature individuals (Table5).”Please go to P17, L320-321.<br>Comment 10: On the line 335-336, This is part of the life history of the species, not somethinf particular to this population. The way it is written, it is misleading.<br>Response: This sentence has been rewritten to “Moreover, the fact that elephants have a long adolescence and long inter-birth intervals means it takes time for NGH

elephants to recover and they are more vulnerable to local population extinction, especially if young individuals are being poached or dying from other unnatural causes. All these facts result in the current small population size that could lead to the extinction of this local population.” Please go to P17, L321-326.
Comment 11: On the line 362, Yes, which makes your suggestion that the population could be bigger even more bizarre.
Response: This sentence has been changed to “The relatively high population density might lead to more intense competition among individuals for limited natural resources, and even greater human-elephant conflict as elephants resort to crop-raiding for food, which could be further studied in the future.” Please go to P19, L350-353.
Comment 12: On the line 368-369, Why in the near future? Isn't it happening already?
Response: This sentence has been changed to “Previous study already confirmed these individuals were related to each other [82]. This phenomenon of inbreeding will be more serious in the near future due to the small population size.”Please go to P19, L357-359.
Comment 13: On the line 387, Why are you talking about unbalanced sex ratio here. it comes from nowhere. I think it should be a new paragraph, and for your reader to follow you, remind them of the sex ratio you found (assuming these individuals were indeed males or not).
Response: We agreed your comments on this paragraph which makes it more logical and coherence. So, we started a new paragraph to talk about sex ratio and have rewritten some sentences. Please go to P20, L378-387.
Comment 14: On the line 390-394, This sentence makes no sense. There are two informations here, but how they are related is a mystery.
Response: This sentence has been deleted.
Comment 15: On the line 399, Ok, it was observed in another population, but what about another one? You cannot cherrypick information as if were supporting yours. Is there a pattern among small population in crowded places? or in areas with low habitat quality? or anything that could explain why females exhibit this low body condition?
Response: The reference we cited before had similar situation on population density, but we had smaller study area. So, this sentence has been deleted.
Comment 16: On the line 401, The comparison is male vs female in adult individuals: leave the immature out.
Response: The “immature males” has been deleted in this sentence. Please go to P21, L392.
Comment 17: On the line 403, You surely mean collecting, which you already use.
Response: Actually, it should be grazing, we used the wrong word. Please go to P21, L394.
Comment 18: On the line 419, Use a proper way to cite this. If these are unpublished documents, cite the staff who provided the information as personal communication. Also, a documentary is not documentation.
Response: This citation way has been changed to “Li Zhimin, personal communication”. Please go to P21, L411.
Comment 19: On the line 437, Sorry, but how documenting this population will ensure its survival? It seems rather a leap of faith!
Response: Sorry the way we wrote the sentence confused you. This sentence has been changed to “The long-term placement of cameras in NNNR is an efficient mean of monitoring to obtain valuable data on demography, fecundity and body condition variation across seasons and years.” Please go to P22, L429-431.
Comment 20: On the line 442, Where was it confirmed first? I don't remember reading anything on that earlier in the manuscript
Response: Sorry for that we used the wrong word. This sentence has been changed to “The results of this study match those observed in earlier studies that providing more suitable habitat is urgently needed to ensure an adequate supply of food for this isolated population during the dry season.” Please go to P22, L433-435.
Comment 21: On the line 447, of what?
Response: This sentence has been changed to “The short-term objective to increase the survival quality of this small population should be to compensate for the food shortages the NNNR population during the dry season by establishing more food sources and improving the quality of existing one to make sure all the individuals have a good body condition.” Please go to P23, L438-441.
Comment 22: On the line 448, by establishing what?
Response: Please see the response of comment 21.
Comment 23: On the line 459-460, Do you mean carrying capacity? I don't think it

Powered by Editorial Manager® and ProduXion Manager® from Aries Systems Corporation

	makes sense, unless you start with that and use it to demosntrate that the population, under the current conditions, is not viable.<br>Response: We agreed your opinion after deeply considering, so, we deleted the "environmental capacity".<br>Comment 24: On the line 461-463, That recommendation makes no sense. First, there is probably inbreeding since quite long: you do not provide evidence that the connection with another population has been lost recently. Second, in the current state of affairs, it seems that ensuring a better body condition is more important than ensuring reproduction.<br>Response: We agreed that you mentioned this population facing the health problems. So, this sentence has been changed to "We emphasize the urgency of implementing these recommendations especially in ensuring a better body condition, which may improve the success rate of reproductivity in the long run." Please go to P23, L452-454.
**Additional Information:**	
**Question**	**Response**
**Financial Disclosure**<br><br>Enter a financial disclosure statement that describes the sources of funding for the work included in this submission. Review the submission guidelines for detailed requirements. View published research articles from *PLOS ONE* for specific examples.<br><br>This statement is required for submission and **will appear in the published article** if the submission is accepted. Please make sure it is accurate.<br><br>**Unfunded studies**<br>Enter: *The author(s) received no specific funding for this work.*<br><br>**Funded studies**<br>Enter a statement with the following details:<br>• Initials of the authors who received each award<br>• Grant numbers awarded to each author<br>• The full name of each funder<br>• URL of each funder website<br>• Did the sponsors or funders play any role in the study design, data collection and analysis, decision to publish, or preparation of the manuscript?<br>• **NO** - Include this sentence at the end of your statement: *The funders had no role in study design, data collection and analysis, decision to publish, or preparation of the manuscript.*<br>• **YES** - Specify the role(s) played.<br><br>* typeset	Funding for this work was provided by National Forest and Grassland Administration, http://http://www.forestry.gov.cn, China under the Project of Asian Elephant Habitat Assessment and Habitat Maintenance Pilot, with effective support by the Yunnan Forest and Grassland Administration. The funders had no role in study design, data collection and analysis, decision to publish, or preparation of the manuscript.

Powered by Editorial Manager® and ProduXion Manager® from Aries Systems Corporation

**Competing Interests**<br><br>Use the instructions below to enter a competing interest statement for this submission. On behalf of all authors, disclose any competing interests that could be perceived to bias this work—acknowledging all financial support and any other relevant financial or non-financial competing interests.<br><br>This statement **will appear in the published article** if the submission is accepted. Please make sure it is accurate. View published research articles from *PLOS ONE* for specific examples.<br><br>**NO authors have competing interests**<br><br>Enter: *The authors have declared that no competing interests exist.*<br><br>**Authors with competing interests**<br><br>Enter competing interest details beginning with this statement:<br><br>*I have read the journal's policy and the authors of this manuscript have the following competing interests: [insert competing interests here]*<br><br>* typeset	The authors have declared that no competing interests exist.
**Ethics Statement**<br><br>Enter an ethics statement for this submission. This statement is required if the study involved:<br><br>• Human participants<br>• Human specimens or tissue<br>• Vertebrate animals or cephalopods<br>• Vertebrate embryos or tissues<br>• Field research<br><br>Write "N/A" if the submission does not require an ethics statement.<br><br>General guidance is provided below.	Both National Forestry and Grassland Administration of China and Yunnan Forestry and Grassland Administration reviewed all sampling procedures and approved permits for the work conducted in NNNR. Non-invasive methods were applied and approval from an Institutional Animal Care and Use Committee or equivalent animal ethics committee was therefore not required.

Powered by Editorial Manager® and ProduXion Manager® from Aries Systems Corporation

Consult the submission guidelines for detailed instructions. **Make sure that all information entered here is included in the Methods section of the manuscript.**<br><br>**Format for specific study types**<br><br>**Human Subject Research (involving human participants and/or tissue)**<br>• Give the name of the institutional review board or ethics committee that approved the study<br>• Include the approval number and/or a statement indicating approval of this research<br>• Indicate the form of consent obtained (written/oral) or the reason that consent was not obtained (e.g. the data were analyzed anonymously)<br><br>**Animal Research (involving vertebrate animals, embryos or tissues)**<br>• Provide the name of the Institutional Animal Care and Use Committee (IACUC) or other relevant ethics board that reviewed the study protocol, and indicate whether they approved this research or granted a formal waiver of ethical approval<br>• Include an approval number if one was obtained<br>• If the study involved *non-human primates*, add *additional details* about animal welfare and steps taken to ameliorate suffering<br>• If anesthesia, euthanasia, or any kind of animal sacrifice is part of the study, include briefly which substances and/or methods were applied<br><br>**Field Research**<br><br>Include the following details if this study involves the collection of plant, animal, or other materials from a natural setting:<br>• Field permit number<br>• Name of the institution or relevant body that granted permission	
**Data Availability**<br><br>Authors are required to make all data underlying the findings described fully available, without restriction, and from the	Yes - all data are fully available without restriction

time of publication. PLOS allows rare exceptions to address legal and ethical concerns. See the PLOS Data Policy and FAQ for detailed information.<br><br>A Data Availability Statement describing where the data can be found is required at submission. Your answers to this question constitute the Data Availability Statement and **will be published in the article**, if accepted.<br><br>**Important:** Stating 'data available on request from the author' is not sufficient. If your data are only available upon request, select 'No' for the first question and explain your exceptional situation in the text box.<br><br>Do the authors confirm that all data underlying the findings described in their manuscript are fully available without restriction?	
**Describe where the data may be found in full sentences. If you are copying our sample text, replace any instances of XXX with the appropriate details.**<br><br>• If the data are **held or will be held in a public repository**, include URLs, accession numbers or DOIs. If this information will only be available after acceptance, indicate this by ticking the box below. For example: *All XXX files are available from the XXX database (accession number(s) XXX, XXX.)*.<br>• If the data are all contained **within the manuscript and/or Supporting Information files**, enter the following: *All relevant data are within the manuscript and its Supporting Information files.*<br>• If neither of these applies but you are able to provide **details of access elsewhere**, with or without limitations, please do so. For example:<br><br>*Data cannot be shared publicly because of [XXX]. Data are available from the XXX Institutional Data Access / Ethics Committee (contact via XXX) for*	All relevant data are within the manuscript and its Supporting Information files.

researchers who meet the criteria for access to confidential data.<br><br>*The data underlying the results presented in the study are available from (include the name of the third party and contact information or URL).*<br>• This text is appropriate if the data are owned by a third party and authors do not have permission to share the data.<br><br>* typeset	
Additional data availability information:	

Powered by Editorial Manager® and ProduXion Manager® from Aries Systems Corporation

Manuscript Click here to access/download;Manuscript;Manuscript.docx

Population structure and body condition assessment to inform conservation strategies for a small isolated Asian elephant population in southwest China

Yakuan Sun[1¶], Ying Chen[2¶], Juan José Díaz-Sacco[1¶], Kun Shi [1,3¶*]

[1]School of Ecology and Nature Conservation, Beijing Forestry University, Beijing, China

[2]School of Biological Science, The University of Hong Kong, Hong Kong, China

[3]Eco-Bridge Continental, Beijing, China

[*]Corresponding author

E-mail: kunshi@bjfu.edu.cn (KS)

[¶]These authors contributed equally to this work.

Abstract

The Asian elephant (*Elephas maximus*) population in Nangunhe National Nature Reserve represents a unique evolutionary branch in China, which has been isolated for more than twenty years from other neighboring populations in Myanmar. The scarcity of information on population structure, sex ratio and body condition makes it difficult to develop effective conservation measures for this elephant population. Twelve individuals were identified from 3,860 valid elephant images obtained from February to June of 2018 (5,942 sampling effort nights) at 52 camera sites. Three adult females, three adult males, one subadult male, two juvenile females, two juvenile males and one calf male were identified. The ratio of adult females to adult males was 1:1, and the ratio of reproductive ability was 1:0.67, indicating the scarcity of reproductive females as an important limiting factor to population growth. A population density of 5.32 ± 1.56 elephants/100 km^2 was estimated using Spatially Explicit Capture Recapture (SECR) models. The health condition of this elephant population was assessed by using an 11-points scale of Body Condition Scoring (BCS), which showed that this population had a medium body condition with an average of 5.75 (n=12, range 2-9). This study finds that this isolated population has an extremely small size and an inverted pyramid age structure and therefore the population is at high risk of extinction. As such, this population should be of top conservation priority among the few remnant populations in China. The database established in this study can provide researchers with valuable information to set up efficient conservation schemes.

Introduction

The Asian elephant (*Elephas maximus*), as the largest terrestrial mammal in Asia, is considered an ecosystem engineer because it can modify its habitat and aid in seed dispersal [1, 2]. The conservation of this umbrella species contributes to the preservation of biodiversity in the ecosystems it uses, especially in tropical moist and in tropical dry broadleaf forests of Southeast and South Asia [3-5]. It is also considered a flagship species that can attract interest for the protection of biodiversity [6]. It is a Class I Key Protected Species in China and listed as Endangered under the IUCN Red List [7, 8]. China sustains a population of c. 300 wild Asian elephants which are divided into seven geographic populations [9, 10]. Most Asian elephant populations are located in Mengyang, Mengla and Shangyong areas of Xishuangbanna National Nature Reserve, while there are also a few populations in Simao, Jiangcheng and Lancang counties of Pu'er city and one small isolated population in the Nangunhe National Nature Reserve (NNNR) of Lincang city [9, 11].

The conventional methods used for tracking Asian elephants in dense forest are difficult to accomplish and usually fail to provide accurate population numbers and age structure [12, 13]. Although it is relatively easy to detect elephant signs, such as dungs or footprints, by using sign surveys [14], it can be difficult to identify individual Asian elephants since signs are highly inaccurate and unreliable [15]. Individuals can be identified using non-invasive DNA methods, but it requires relatively fresh samples and the lab costs can be quite high; moreover, ~~the~~ population size can only be determined with genetic capture-mark-recapture methods, which can be time and labor intensive [16, 17]. Furthermore, since elephant calves have lower defecation rates, this method

can underestimate the number of young individuals in a population [18].

Infrared trigger camera technology can make up for the above-mentioned disadvantages [19, 20]. Infrared cameras cause minimal disturbance to the study animal and its habitat, can be used for long working hours, can withstand harsh weather conditions, and are relatively cheap [19, 21, 22]. Infrared cameras are widely used to obtain ecological baseline surveys of protected species [23, 24], to monitor endangered wildlife [25], to study habitat utilization [26], to observe animal behavior [27], and to study the mechanisms of coexistence in sympatric species [28, 29]. Moreover, infrared cameras can also be used to estimate population size and density by using Spatially Explicit Capture-Recapture models (SECR) [30-33]. This technology has been used extensively to estimate the population density of multiple species, especially those possessing easily observed markings, such as the snow leopard (*Panthera uncia*), the leopard cat (*Prionailurus bengalensis*), the Sumatran tiger (*Panthera tigris* ssp. Sumatrae), and the serval (*Leptailurus serval*) [33-36]. For species without natural markings, identification can also be conducted according to morphology, such as with the Asian elephant, the giant panda (*Ailuropoda melanoleuca*), and the fox squirrel （*Sciurus niger*) [37-39]. Throughout the Asian elephant range, there have been a number of studies that have identified individual elephants by directly photographing them or by obtaining images from infrared cameras [17, 37, 40, 41].

Population structure and density estimation are essential features that can be used to study the ecology of wildlife populations [42, 43]. The Body Condition Scoring (BCS) is an index of an animal’s health that reflects the quality of the environment in which

the animal lives [44]. It can be used in conjunction with population structure and density estimation to obtain a more accurate picture of the health of a population. Furthermore, it can be used for both longitudinal and cross-sectional studies, is comparatively inexpensive, and does not require a high technical capacity [45]. While BCS is individual-based, it is most meaningful when applied to a population, as an early indicator of the impact of management actions on the average health condition of a population [45, 46]. For Asian elephants, the BCS obtained through photographs can be as accurate as ultrasonic subcutaneous fat measurement, thus providing a safer and non-invasive method to evaluate the health condition of wild populations [45, 47].

In China, population studies have mainly been concentrated in Xishuangbanna National Nature Reserve [17, 40], and previous studies were limited to studying population sizes and none have assessed the health condition of the population[13, 48]. Although it is a unique evolutionary unit in China, the isolated elephant population in NGH has been poorly studied and the lack of scientific data and information on population structure, relatedness, and health status prevent the development of efficient conservation strategies. Additionally, this population is facing threats from habitat loss, fragmentation and illegal hunting [51, 53, 54]. An accurate understanding of the current situation of the local elephant population in NNNR, especially its structure and health condition, is key to establishing a baseline for the development of more effective protection and management schemes.

In this study, our aim was to determine the size, density, demographic structure and health condition of the NNNR Asian elephant population by using infrared camera

trapping. We sought to understand which factors might have affected its population size in recent years in order to develop targeted and effective protection strategies. Furthermore, our approaches can be applied to other elephant populations with further implications for global Asian elephant conservation.

Material and Methods

Ethics Statement

Both National Forestry and Grassland Administration of China and Yunnan Forestry and Grassland Administration reviewed all sampling procedures and approved permits for the work conducted in NNNR. Non-invasive methods were applied and approval from an Institutional Animal Care and Use Committee or equivalent animal ethics committee was therefore not required.

Study area

The study area is located in the western part of the Nangunhe National Nature Reserve, Yunnan, where elephants are found [51, 53] (Fig 1). It occupies 70.82 km^2 at latitude 23°13'- 23°19'N, longitude 98°54'- 99°05'E, and the altitude ranges from 510 m to 1747 m. Rainfall is highly seasonal, with a dry season between November and April, and a rainy season between May and October. Mean annual precipitation is 351.3 mm in the dry season and 1983.3 mm in the rainy season, while temperature varies at different altitudes. The average monthly temperature range is hottest in July, varying between 12.3-25.5 ℃ and it is coldest in January with values between 2.8-14.3 ℃ [55]. Vegetation and climate vary across elevation zones. Tropical forest and tropical

monsoon forest are located at altitudes below 600 m, middle elevation forests are dominated by subtropical monsoon evergreen broad-leaf forests, and evergreen broadleaf forests are found above 1700 m [53]. Bamboo forest, shrub and grasslands are scattered throughout this area. Deforestation along the China and Myanmar border has disrupted the connectivity between elephant populations that are found in both countries [51]. Furthermore, an iron fence in the southern part of reserve is used to protect the surrounding villages from wildlife (Fig 1).

Fig 1. Map of study area in NNNR showing the camera sites with or without elephants.

Camera trap set-up

We carried out the survey for four months between February and May 2018 using automatically triggered camera traps (Ltl Acorn 6210). There was a total of 52 cameras stations which successfully recorded 43-129 camera trap nights in the field (Fig 1). The total camera trapping effort was 5,942 trap nights (114.27±19.83). All the stations were located in evergreen broadleaf forest, broadleaf and bamboo forest, bamboo forest, pine forest and nearby brush forest, which represents the preferred habitat of elephants in NNNR. We systematically deployed camera traps along the animal trails and forest gaps where there were signs of elephants, including lying sites, foraging sites, footprints, dungs and urine marks. We attempted to deploy cameras throughout the reserve following a 1 km^2 grid. We increased the number of cameras in the grids with many elephant signs, always with a minimum of 200 m between any two cameras. We avoided the northwestern part of the reserve as the topography is tough, with steep cliffs of up to 1747 meters, and historically there have not been any records of elephants in this

area [56, 57].

Cameras were strapped onto trees at approximately 1.5 m above the ground with the sensor parallel to the ground to maximize the extension of the detection zone. The positions of all the camera sites were recorded by GPS. To avoid blank images caused by sunlight, the cameras were never placed facing east. Camera traps were hidden as much as possible to reduce the disturbance to wildlife and the probability of being stolen. Camera traps were set to operate 24 hours per day and programmed to take three photographs and one 12-second video as a sequential without delay, registering the date and time for each exposure. At each site, a camera trap was operated continuously for a maximum of four months. SD cards and batteries were replaced every month. We recorded the time of installation and retrieval of each camera and calculated the total duration of sampling.

Individual identification

The images obtained from the camera traps were categorized to independent events, which were defined as “sequences”. Each sequence was defined by a set of images adjacent in time. For an elephant unit (defined as all or the maximum number of individuals in a group), the sequence included the images from the first individual entering the field to the last one exiting it, making it possible to identify different individuals based on distinct features. For solitary individuals, a sequence was a series of captured images of same individual within a continuous time period [58]. Only images with sufficient quality, clarity and position of the elephant in the frame were used for individual identification [58]. Here, quality referred to how usable an image

was for age and sex classification; clarity indicated whether the elephant was "captured" in good light or that the flash reached the individual, and the image was in focus and not over or under-exposed; and position referred to the occurrence of the elephant within an 'optimal distance and angle' from the camera [41, 58]. The age of each animal was broadly classified into adult, subadult, juvenile and calf using approximate shoulder height as recommended by previous studies, and gender was mainly determined by secondary sexual characteristics [59-61] (Table 1). We defined the age structure as the ratio of adults, subadults, juveniles and calves while the reproductive ability was the ratio of age structure between adult and juvenile females [41], due to the absence of subadult and calf females in this reserve. Male elephants living completely or mostly outside the herd were classified as solitary males [12]. Based on individual events, a capture-history event database was established to record the individuals and their characteristics. Morphological characteristics including facial bones, ear, tusk, back and tail hair, old scars and injuries, as well as physical characteristics related to sex and age classes, confirmed the reliability of identification results [17, 37, 40, 62] (Table 1).

Table 1. Morphological characteristics used for individual identification of elephants photographed in NNNR, between February and May 2018.

Characteristics	Categories	Description
Age	Adult (>15years)	With the highest shoulder height
	Subadult (5-15years)	Shoulder height reaches an adult's eyes
	Juvenile (1-5years)	Shoulder height reaches half the height of an adult

	Calf (0-1year)	Below an adult’s knee
Sex	Female	Without obvious tusks but with prominent breasts, belly swelling or the presence of nursing offspring
	Male	With visible testicles or penis, and/or obvious tusks
Facial bones	Wisdom tumor	Size
	Bones between the eyes	Extent of prominence
	Upper margin of eyes	Extent of depression
Tusks	Length	Very short/short/long compared with trunk
	Thickness	Thick/normal/slender
	Shape	Straight/bent
	Crack	Yes/no
Ear	Upper edge	Fold/unfold
	Lobe	V-acute/U-rounded
	Lobe tear	Yes/no
	Ear hole	Yes/no
Tail brush	Standard	Present/both sides/both-continuous
	Nonstandard	Absent/single side/both-discontinuous

Density estimation

The elephants in NNNR belong to a small isolated population without any migrants, thus conforming to the “close population” assumption of SECR [63]. The model accounts for ~~the~~ movement in density estimation by combining a state model and an observation model, which describe how the observed data were generated [63]. The state model describes statistically the mechanisms determining the distribution of the animals in the study area, while the observation model quantifies the probabilities of detection or capture, given the searched region, their locations and characteristics at different sessions [64]. Basically, SECR are generalized linear mixed models that assume that each individual *i* in the population has its own unobserved activity center S_i, and all the activity centers $S_i \cdots S_N$ are distributed across the study area [65, 66].

The encounter rate of an individual *i* with a given trap *j* located at X_j is a monotonically decreasing function of the distance from S_i to *j*. Y_{ij}, the number of times individual *i* is detected by trap *j* during a sampling occasion, is a random variable following a Poisson distribution and with mean λ_{ij} [67]:

$$Y_{ij} \sim \text{Poisson}(\lambda_{ij})$$

For an elephant *i* captured by a trap *j*, the model assumes a log-linear form [68]:

$$\text{Log}\left(\lambda_{ij}\right) = \text{Log}(\lambda_0) - \left(\frac{1}{2\sigma^2}\right) \left\|S_i - X_j\right\|$$

Here, λ_0 is the baseline encounter probability, the function $\|S_i - X_j\|$ is the distance between the activity center and the trapping station, and σ is the Gaussian scale parameter for the distance function between activity centers and trap locations; assuming a bivariate normal model of space use, sigma can be used to calculate a 95% activity area radius.

SECR models estimate the density of animal activity centers in an area large enough so that animals residing beyond it have a negligible chance of being detected [63]. Considering the small size of the elephant distribution, with the east and west boundary being no wider than 10 km, we therefore defined our study area as extending 5 km beyond all camera stations, corresponding to an area of 255.8 km^2 [69].

SECR 3.1.5 package in R 3.6.1 was used for density estimation. Data were prepared as an R object of class “capthist”. This object included both the capture data and the detector layout. The file named “capture histories” contained data on individual identified at each sampling occasion from the specific detector (camera). The detector

file contained data on cameras ID, and x, y coordinates. Five different sessions, corresponding to each of the months and one corresponding to the whole survey period, were used to run the model to see the estimation accuracy under different detection efforts.

Body condition scoring

We established a database of each identified individual and the images with the best angle of each individual elephant were selected to score the body condition. Compared with previous studies, our target population size was small, and the sample size was limited so we used a simple method which can rapidly and more easily be applied to wild elephant populations. In this study, we applied a BCS method that focuses on six physical characteristics and ranks the elephant´s body condition according to an 11-points scale [44, 45]. Five of the clearest images for each physical characteristic were selected to assess the body condition. Thirty images were used to calculate the comprehensive scores for each individual. This scoring method is based on the extent of visibility of depressions around bone structures. Depressions around bones become visible as an animal loses its subcutaneous fat deposits and muscles in the region concerned, thereby making bones appear more prominent. Skull, pectoral girdles in shoulders, vertebral column, ribs and pelvic girdles are prominent bone structures. Thick rolls of skin that fold below the neck can accumulate fat, and thus, also represent a criteria of good body condition. We presented a scale of 5 reference scored elephants description of 1, 3, 5, 7, 9, with numerically higher values corresponding to better body condition (Table 2). However, the range of scores

extended from 0 to 10 due to the possibility of greater variation. For example, if all the ribs of an elephant were visible, it meant that this individual presented a worse body condition than 1 and thus, it would be assigned 0. If an individual presented a fatter condition than 9 and had very thick rolls of skin fold below neck, it would be assigned a score of 10. Scores ranging from 0-3 were considered as poor, 4-7 were considered medium and 8-10 were considered as good [46]. Pearson correlation coefficient (r) was used to study the relationship between BCS and various covariates such as sex, age, and whether an individual was solitary or in a herd.

Table 2. Diagnostic characteristics pertaining to scores in photographic scale.

Score	Characteristics
1	Ribs from shoulder to pelvis are visible, some ribs prominent (spaces in between sunken in)
3	Some ribs visible (spaces in between not sunken in), shoulder and pelvic girdles prominent
5	Ribs not visible, shoulder and pelvic girdles visible
7	Backbone visible as a ridge, shoulder and pelvic girdles not visible
9	Backbone not visible or difficult to differentiate and pelvic bones not visible

Results

Population size and density

A total of 70,902 images were obtained, among which 7.34% (5,207) were Asian elephant data. There was a total of 134 independent events, including 40 events of elephants in a herd and 94 events of solitary elephants. Excluding the image data that could not be used for individual identification, there were 3,860 valid Asian elephant images belonging to 85 independent events (26 events of individuals in a herd and 59 events of individuals). Generally, all herds and individuals had similar frequencies of recapture histories during the study period, particularly herds. However, solitary

elephants were image-captured with a minor difference: a minimum of 16 capture times for AE12 compared to a maximum of 25 capture times for AE10 (Table 3).

Table 3. Number of capture events per individual per month.

Individual No.	Age	Gender	Tusk	Type	Number of Capture events				
					**Feb.**	**Mar.**	**Apr.**	**May**	**Total**
AE01	Juvenile	Male	Yes	Herd	2	6	5	8	21
AE02	Adult	Female	No	Herd	3	5	6	6	20
AE03	Adult	Female	No	Herd	3	5	6	7	21
AE04	Juvenile	Female	No	Herd	2	6	5	7	20
AE05	Calf	Male	No	Herd	3	6	8	5	22
AE06	Adult	Female	No	Herd	4	6	8	5	23
AE07	Juvenile	Female	No	Herd	4	6	9	6	25
AE08	Juvenile	Male	Yes	Herd	4	7	6	7	24
AE09	Sub-adult	Male	No	Solitary	3	9	2	9	23
AE10	Adult	Male	Yes	Solitary	2	3	13	7	25
AE11	Adult	Male	No	Solitary	3	5	8	5	21
AE12	Adult	Male	No	Solitary	2	6	1	7	16
Total	-	-	-	-	35	70	77	79	261

A total of 12 individuals were identified among these valid independent events (Fig 2; S1 Dataset). The elephant population size in each session was consistent, except in February. Moreover, the population size estimated by SECR was 12.00 ± 0.71 (with a 95% confidence interval of 12.00-12.11; Table 4). The estimated density by SECR was 5.32 ± 1.56 elephants/100km^2 (with a 95% confidence interval of 3.02-9.36). In camera-trapping session at monthly intervals, April had the lowest density estimation at 6.07 ± 1.79 elephants/100km^2, while May had the highest density estimation at 24.43 ± 7.20 elephants/100km^2. The movement parameter σ was twice as large in April (6643 m ± 2356 m) than in May (3771 m ± 1471 m).

Fig 2. Asian elephants individually identified in NNNR, China. Each individual's

most distinguished characteristic was emphasized, except easily identified AE05 and AE10, the only calf and adult male with tusks.

Table 4. Population density estimation by using SECR models under five sessions.

Sessions	Occasions	Density			λ_0		sigma		Population size		
		estimate	SE.estimate	95% CI	estimate	SE.estimate	estimate	SE.estimate	estimate	SE.estimate	95% CI
February	11	10.71	3.31	5.91-19.38	0.15	0.04	2983	664	14.00	2.19	12.35-23.43
March	26	6.57	1.94	3.73-11.58	0.07	0.02	4193	700	12.20	0.87	12.00-17.95
April	22	6.07	1.79	3.45-10.70	0.02	0.004	6643	2356	12.05	0.75	12.00-16.87
May	26	24.43	7.20	13.87-43.03	0.01	0.003	3711	1471	12.03	0.74	12.00-16.48
Total	85	5.32	1.56	3.02-9.36	0.02	0.003	6474	1065	12.00	0.71	12.00-12.11

Demographic structure

We identified an inverted pyramid age structure for this population: three adult females, three adult males, one subadult male, two juvenile females, two juvenile males and one male calf, with a sex ratio of 1:1.4 using females as a reference (Table 4). The three adult males comprised one tusker and two without tusks (Makhnas), giving an adult tusked to tuskless male ratio of 1:2. The subadult male was also a makhna, increasing the tusked to tuskless male ratio to 1:3. The ratio of reproductive ability was 1:0.67.

The social network was comprised by AE01-AE08 that formed joint-family unit, with calf AE05, adult female AE06 and juvenile female AE07 forming a mother-calf unit which occasionally departed from the large family unit. AE06 and AE05 were in a mother-child relationship since there were images of the calf nursing from the female.

The solitary subadult male AE09 and adult male AE10 were observed to occasionally join the family unit.

Body condition scoring

The distribution of BCS for the population had an average of 5.75 (n=12, range 2-9; Fig 3). Body condition evaluation revealed that 33.3% of the elephants were in a good body condition, while 50% and 16.7% were in medium and poor body conditions, respectively (Fig 3). Age was not significantly correlated to BCS (r=0.03), but sex was (r=0.42), as adult males had better body conditions than adult females. The body condition of the only three fertile females in this population was not good. The adult female AE02 had a BCS just above the average score, while the other females, AE03 and AE06 were in poor body conditions with a low BCS of 3. Being solitary or in the herd played more important roles in the BCS (r=0.53) than sex. Individuals that were part of the herd had a lower BCS than solitary individuals.

Fig 3. Body condition score of the 12 individuals identified in NNNR.

Discussion

Population size and density

Accurate assessment of population size is essential for species conservation and management [70]. Identification from captured images was robust enough to identify individuals [71], unlike dung and footprints which are easily influenced by slope, soil quality and climatic factors [72]. This study used cameras installed in the elephant preferred habitats that covered most of the western part of the reserve, which provided

a higher chance of obtaining the data reflecting the real population, leading to a more reliable population assessment than in the study that used drone surveys [54]. Previous studies have estimated this population to be around 20 individuals. Although population estimation using different methods could generate bias in direct comparison, research has shown that this elephant population has remained small during the last few decades[54, 72-74] (S1 Table). The NNNR population is an extremely small and isolated population, compared with other elephant populations found around the world [3, 9, 10, 12]. Historical illegal hunting has had a direct impact on this population with a total of 15 unnatural deaths since 1987 (Table 5). Additionally, 3 of 4 elephants found dead since 2003 have been immature individuals (Table 5). Moreover, the fact that elephants have a long adolescence and long inter-birth intervals means it takes time for ~~NGH~~ elephants to recover and they are more vulnerable to local population extinction [12, 76], especially if young individuals are being poached or dying from other unnatural causes. All these facts result in the current small population size that could lead to the extinction of this local population.

Table 5. Unnatural deaths of Asian elephant since the establishment of NNNR.

Year	Number of individuals	Cause of death	Data resources
1987	Two: One female and one male	Illegal killing	[55, 77]
1988	Four: Two adult males, one adult female and one calf	Poaching, electrocuting and unknown reason	[51]
1995	One male	Unknown	[54]
1996	One male	Vengefully hunted by local people	[53]
1997	Three: One female, one male and a calf	Unknown for the calf, the female and male were killed	[55]

2003	A Subadult male (about 10 years old)	Unknown	[55]
2008	One subadult male	Unknown	Communication with local residents and rangers
2011	One juvenile	Unknown	[55]
2016	One adult male	Unknown, but the tusks show visible saw marks	Communication with local residents and rangers

SECR modelling has become a powerful tool to estimate the small population ~~sizes~~ like the one in NNNR [32]. The model defines the activity center according to the range of the species activities [78], so it is easy to overestimate the population density in a short survey period, which was confirmed by monthly estimates in this study (Table 4). We first used ~~the~~ single monthly session to run the SECR model, with which we obtained a significant difference in density estimation. The SECR model simulated a larger movement buffer (sigma=6643 m) in April, which resulted in the smallest population density. May presented the highest density because there were 8 cameras that captured elephants, and the distance between each one was less than 800 m, which is smaller than their daily movement ~~distance~~ [79, 80]. The ~~instability~~ of single month session results by SECR prompted us to use the four months data as the fifth session to run the model. We thus obtained the smallest standard deviation, which indicated that the result was closer to the actual value [41].

The population density results of the NNNR elephant population, as an unique evolutionary clade in China, can be used to compare with results from other populations in order to improve our understanding of elephant ecology and develop more detailed management and conservation plans [81]. However, with no other SECR analyses conducted on other populations in China, we used the crude density estimation

(estimated population number/study area) to draw comparison between the NNNR population and others in Yunnan [41]. The crude density estimation of Asian elephants in NNNR was the highest at 16.9 elephants/100 km^2 due to its smaller area of distribution, compared with that in Shangyong, Mengla, and Menyang with a density of 10.68, 4.26, and 1.85 elephants/100 km^2, respectively [13, 17]. The relatively high population density might lead to more intensive competition among individuals for limited natural resources, and even greater human-elephant conflict as elephants resort to crop raiding for food, which could be further studied in the future.

Population structure in NNNR

Although the juveniles in this population accounted for 33% of the population, higher than the minimum of 20% of the population considered to be needed for it to be self-sustaining in a short period of time [18]. Previous study already confirmed these individuals were related to each other[82]. This phenomenon of inbreeding will be more serious in the near future due to the small population size.

We attempted to identify the relationship between individuals in the family unit through images captured by camera trap, since the allomaternal care or mother-like behavior directed by females are prevalent in the Asian elephant social structure [83, 84]. We could not match adult females with their offspring in the groups relying on the observed behaviors, even though we captured intimate interaction between AE02 and AE08, as well as between AE03 and AE04. In recent years, the death of adult females has not been recorded in the reserve (Table 5). Therefore, we assumed that all the current juveniles came from these three adult females. Genetic studies are urgently

needed to better understand the lineage and degree of inbreeding depression in the NNNR population.

There were two tuskless juveniles that had body heights similar to that of the juvenile males identified by their apparent tusks and that were identified as females. It should be noted that males in some populations tend to be tuskless for survival due to intensive historical poaching pressure [12, 85]. The high proportion of ~~makhnas~~ in NNNR identified in this research could indicate that these juveniles identified as females might in fact be males. Since tuskless males are common in this population, it would be helpful to conduct genetic studies on this population that can confirm the sex of these two juveniles.

The extremely unbalanced sex ratio ~~that~~ skewed to males is not beneficial for population growth [86]. Although more males in social mammals could maintain the effective population size and increase genetic diversity, elephants' ability to recognize kin and avoid inbreeding might mitigate the loss of genetic diversity which cause local e[illegible]ction in small population [87]. Females do not enter the breeding period until they are 10-14 years old and as such the~~se~~ two juvenile females in the NNNR population still need~~ed~~ 3-7 years to become mature [9, 12, 89]. Currently, only females AE02, AE03 and AE06 are of reproductive age, but the latter was nursing a calf, leaving only two sexually active females in the reserve. In either case, the scarcity of fertile females would be the primary constraint on population growth due to their long birth intervals.

Health condition

We chose a simple but accurate body condition method to assess the health of this

small population. Our results showed a higher body condition of adult males than adult females. This may be because adult males are known to have larger home ranges than adult females [80, 90], and are more tolerant to human disturbance [91]: that means they can explore more frequently areas near the reserve boundary to obtain more food resources~~,~~ where intensive anthropogenic activities like hunting, grazing, fishing and collecting have also been recorded by cameras. Additional nutritional stress from lactation, ~~like~~ AE06, was another reason that could have resulted in an even poorer body condition than males [46]. Furthermore, solitary elephants, which were males, could have also had a higher BCS than the individuals in the family units because at least one of the herds could have had an even more reduced area of movement due to the presence of a calf [90]. Additionally, the other two adult females both presented a medium health condition, which would not be conducive for reproduction [44].

Most of the individuals (66.7%) had medium or poor body conditions during the survey period, which corresponded to the dry season. Since the quantity and quality of food and availability of water are the primary factors determining the body condition of free-ranging elephants [44], both female and male individuals show an obvious thinner body condition during the dry season compared to the rainy season [92]. We did not have the opportunity to conduct camera trap surveys during the rainy season since many cameras failed to work in this season. Additionally, the rainy season coincides with the harvest period. In the past, especially in recent years, NNNR elephant-damage compensation data has shown that the elephants forage for corn and rice during the rainy season from July to October (Li Zhimin, personal communication). Therefore, we

assume that at least males in this population are foraging crops to supplement ~~its~~ diet. Future studies should focus on determining how crops contribute to improving the body condition of elephants during the rainy season.

~~Moreover, the~~ body condition assessment in this study could have implications for the research and conservation of other elephant populations. Since the vast majority of Asian elephant populations, especially in China, are found outside of reserves and near cropland, the health of these populations are likely heavily influenced by access to crops [93]. Massive amounts of data have been collected by camera traps and drones in these areas [94, 95], but none have been used to determine the health of elephant populations. We have shown that using images to assess BCS is a simple and fast method for determining the health of free-ranging elephant populations. Therefore, future studies should consider analyzing the body condition of regional Asian elephant populations from these data sets to obtain early indications of the health of individuals and quality of food resources in their habitats [45]. This in turn can be used to guide conservationists and policymakers to evaluate the necessity for interventions, including the restoration of suitable habitat [44].

Conservation recommendations

The long-term placement of cameras in NNNR could be an efficient approach of monitoring to obtain valuable data on demography, fecundity and body condition variation across seasons and years. Molecular biological approaches are also needed in further studies to determine the kinship and genetic health status of this population.

The results of this study match those observed in earlier studies that providing

more suitable habitat is urgently needed to ensure an adequate supply of food for this isolated population during the dry season [13, 51, 53]. Attention should be paid to the low body condition in the dry season, and a follow-up survey should be carried out in the rainy season to verify the health status of this elephant population throughout the year. Therefore, the short-term objective ~~to increase the survival quality f~~ this small population ~~should be to compensate for the food shortages the NNNR population during the dry season by~~ establishing more food sources and improving the quality of existing ~~one~~ to ~~make sure all the~~ individuals ~~have a good~~ body condition [96]. In the mid-term, fence dismantlement should be prioritized because it has partially blocked the movement of elephants and hindered their ability to find new food resources (Fig 1). Allowing the animals greater movement could provide more opportunities for the elephants to explore new suitable areas with enough food, eventually improving elephant ~~health~~ condition regardless of the season. Ecological corridors should be considered as a long-term objective to connect this isolated elephant population to other suitable habitats. Since the western part of this reserve (Fig 1. Red part of NNNR) has some suitable habitats [53], an ecological corridor could be created by planting bamboo forest or bamboo-broadleaf forests, allowing individuals to move between the eastern and western parts of the reserve. We also suggest conduct research on the evaluation of the availability of food resources at different seasons. We emphasize the urgency of implementing these recommendations especially ~~in ensuring~~ a better body condition, which may improve ~~the~~ success ~~rate of reproductivity~~ in the long run.

Acknowledgments

This study was implemented by Beijing Forestry University and Beijing Eco-Bridge Continental. We thank the Second National Survey of Terrestrial Wildlife in China and National Forestry and Grassland Administration of China for their support of this study. Furthermore, the field work was strongly supported by the administrators of Nangunhe National Nature Reserve, for which we thank them. We also thank all the field staff, including Wang Jiahui, Liu Zhen, Liu Ying and all local rangers, especially Chen Deming, Li Yongxiang, Yang Shaobing, Wang Zhisheng, Li Chunlian and Li Zhimin, for their hard and invaluable work.

References

1. Campos-Arceiz A, Blake S. Megagardeners of the forest – the role of elephants in seed dispersal. Acta Oecologica. 2011;37(6):542-553. https://doi.org/10.1016/j.actao.2011.01.014.

2. McConkey KR, Nathalang A, Brockelman WY, Saralamba C, Santon J, Matmoon U, et al. Different megafauna vary in their seed dispersal effectiveness of the megafaunal fruit Platymitra macrocarpa (Annonaceae). Plos One. 2018;13(7):e0198960. https://doi.org/10.1371/journal.pone.0198960. PMID: 30020929.

3. Riddle HS, Schulte BA, Desai AA, Meer Lvd. Elephants - A conservation overview. Journal of Threatened Taxa. 2010;2(1):653-661.

4. Tan A, Yu L. Distribution and Protection of Asian elephant in China. Protection Forest Science and Technology. 2015;(5):89-91. https://doi.org/10.13601/j.issn.1005-5215.2015.05.035.

5. Platt SG, Bickford DP, Win MM, Rainwater TR. Water-filled Asian elephant tracks serve as breeding sites for anurans in Myanmar. Mammalia. 2019;83(3):287-289. https://doi.org/10.1515/mammalia-2017-0174.

6. Bowen-Jones E, Entwistle A. Identifying appropriate flagship species: the importance of culture and local contexts. Oryx. 2002;36(2):189-195. https://doi.org/10.1017/S0030605302000261.

7. Jiang Z, Jiang J, Wang Y, Zhang E, Zhang Y, Li L, et al. Red List of China’s Vertebrates. Biodiversity Science. 2016;24(5):500-551. http://www.biodiversity-science.net/EN/10.17520/biods.2016076.

8. Choudhury A, Lahiri Choudhury DK, Desai A, Duckworth JW, Easa PS, Johnsingh AJT, et al. Elephas maximus. The IUCN Red List of Threatened Species. 2008:e.T7140A12828813. https://dx.doi.org/10.2305/IUCN.UK.2008.RLTS.T7140A12828813.en.

9. Li Z. Research on Asian elephan Conservation in China. Beijing: Science Press; 2018.

10. Yu Z, Kun J. Distribution, Population, Habitat Status and Population Management of Asian Elephant. World Forestry Research. 2018;31(2):25-30. https://doi.org/10.13348/j.cnki.sjlyyj.2018.0002.y.

11. Mingyong C. Asian elephant research in China: Science Press; 2006.

12. Sukumar R. A brief review of the status, distribution and biology of wild Asian elephants Elephas maximus. International Zoo Yearbook. 2006;40(1):1-8. https://doi.org/10.1111/j.1748-1090.2006.00001.x.

13. Zhang L, Dong L, Lin L, Feng LM, Yan F, Wang LX, et al. Asian Elephants in China: Estimating Population Size and Evaluating Habitat Suitability. Plos One. 2015;10(5):e0124834. https://doi.org/10.1371/journal.pone.0124834. PMID: 25992617.

14. Yuan T, Yongjun F, Chunlan Z, Baocheng Y, Xiaoping T, Huijian H. Effectiveness of line transects during wild animal surveys in mountain forest of South China. Biodiversity Science. 2015;23(1):109-115. http://www.biodiversity-science.net/EN/10.17520/biods.2014128.

15. Barnes RFW. The problem of precision and trend detection posed by small elephant populations in West Africa. African Journal of Ecology. 2002;40:179-185. https://doi.org/10.1046/j.1365-2028.2002.00376.x.

16. Mills LS, Citta JJ, Lair KP, Schwartz MK, Tallmon DA. Estimating Animal Abundance Using Noninvasive DNA Sampling: Promise and Pitfalls. Ecological Applications. 2000;10(1):283-294. https://doi.org/10.2307/2641002.

17. Zicheng Y, Ying C, Junsong L, Lifan W, You P, Zhiyong S, et al. Individual identification and population size assessment for Asian elephant based on camera-trapping techniques. Acta Theriologica Sinica. 2018;38(1):18-27. https://doi.org/10.16829/j.slxb.150109

18. L.S.Eggert, J.A.Eggert, D.S.Woodruff. Estimating population sizes for elusive animals: the forest elephants of Kakum National Park, Ghana. Molecular Ecology. 2003;12(6):1389-1402. https://doi.org/10.1046/j.1365-294X.2003.01822.x. PMID: 12755869.

19. Liu X, Wu P, He X, Zhao X. Application and data mining of infra-red camera in the monitoring of species. Biodiversity Science. 2018;26(08):850-861. https://doi.org/10.17520/biods.2018053.

20. Xiao W, Hu L, Huang X, Xiao Z. Using capture-recapture models in wildlife camera-trapping monitoring and the study case. Biodiversity Science. 2019;27(3):257-265. https://doi.org/10.17520/biods.2018194

21. Zhang L, Cui S, Huang Y, Chen D, Qiao H, Li C, et al. Infrared camera traps in wildlife research and monitoring in China: issues and insights. Biodiversity Science. 2014;22(6):696-703. https://doi.org/10.3724/SP.J.1003.2014.14225.

22. Wearn OR, Glover-Kapfer P. Snap happy: camera traps are an effective sampling tool when compared with alternative methods. Royal Society Open Science. 2019;6(3):181748.

https://doi.org/10.1098/rsos.181748. PMID: 31032031.

23. Jorge A A, Carlos E F S, Krisna G, Chris H, Johanna H, Emanuel M, et al. Community structure and diversity of tropical forest mammals: data from a global camera trap network. Philosophical Transactions of the Royal Society of London. 2011;366(1578):2703-2711. https://doi.org/10.1098/rstb.2011.0115. PMID: 21844049.

24. Chengcheng Z, Jun W, Alexander JS, Zhigang D, Liji W, Wantao D, et al. Biodiversity Assessment of Mammal and Bird Species from Camera Trap Data in Yanchiwan National Nature Reserve, Gansu Province, China. Journal of Resources and Ecology. 2018;9(5):566-574. https://doi.org/10.5814/j.issn.1674-764x.2018.05.014.

25. Broto B, Mortelliti A. The status of research on the mammals of Sulawesi, Indonesia. Mammal Review. 2019;49(1):78-93. https://doi.org/10.1111/mam.12141.

26. Montalvo VH, Sáenz-Bolaño C, Luis D. Alfaro1 JCC, Guimarães-Rodrigues FH, Carrillo E, Sutherland C, et al. Seasonal use of waterholes and pathways by macrofauna in the dry forest of Costa Rica. Journal of Tropical Ecology. 2019;35(2):68-73. https://doi.org/10.1017/S0266467418000457.

27. Matsukawa A, Ahmad AH, Kohshima S. Social structure and reproduction of long-tailed porcupine (*Trichys fasciculata*). Mammalia. 2019;83(1):23-33. https://doi.org/10.1515/mammalia-2017-0104.

28. Sunarto S, Kelly MJ, Parakkasi K, Hutajulu MB. Cat coexistence in central Sumatra: ecological characteristics, spatial and temporal overlap, and implications for management. Journal of Zoology. 2015;296(2):104-115. https://doi.org/10.1111/jzo.12218.

29. Rich LN, Miller DAW, Muñoz DJ, Robinson HS, McNutt JW, Kelly MJ. Sampling design and analytical advances allow for simultaneous density estimation of seven sympatric carnivore species from camera trap data. Biological Conservation. 2019;233:12-20.

https://doi.org/10.1016/j.biocon.2019.02.018.

30. Blanc L, Marboutin E, Gatti S, Gimenez O. Abundance of rare and elusive species: Empirical investigation of closed versus spatially explicit capture-recapture models with lynx as a case study. The Journal of Wildlife Management. 2013;77(2):372-378. https://doi.org/10.1002/jwmg.453.

31. Efford MG, Fewster RM. Estimating population size by spatially explicit capture-recapture. Oikos. 2013;122(6):918-928. https://doi.org/10.1111/j.1600-0706.2012.20440.x.

32. Bashir T, Bhattacharya T, Poudyal K, Sathyakumar S, Qureshi Q. Estimating leopard cat Prionailurus bengalensis densities using photographic captures and recaptures. Wildlife Biology. 2013;19(4):462-472. https://doi.org/10.2981/12-098.

33. Alexander JS. Advancing research and conservation of snow leopards in the Qilianshan mountains of China: management and methodological implications [Doctoral dissertation]: Beijing Forestry University; 2015.

34. O'Brien TG, Kinnaird MF, Wibisono HT. Crouching tigers, hidden prey: Sumatran tiger and prey populations in a tropical forest landscape. Animal Conservation. 2003;6(2):131-139. https://doi.org/10.1017/S1367943003003172

35. Bohm T, Hofer H. Population numbers, density and activity patterns of servals in savannah patches of Odzala-Kokoua National Park, Republic of Congo. African Journal of Ecology. 2018;56(4):841-849. https://doi.org/10.1111/aje.12520.

36. Park H, Lim A, Choi T-Y, Baek S-Y, Song E-G, Park YC. Where to spot: individual identification of leopard cats (*Prionailurus bengalensis* euptilurus) in South Korea. Journal of Ecology and Environment. 2019;43. https://doi.org/10.1186/s41610-019-0138-z.

37. Goswami VR, Madhusudan MD, Karanth KU. Application of photographic capture-recapture

modelling to estimate demographic parameters for male Asian elephants. Animal Conservation. 2007;10(3):391-399. https://doi.org/10.1111/j.1469-1795.2007.00124.x.

38. Tye CA, Greene DU, Giuliano WM, Mccleery RA. Using camera-trap photographs to identify individual fox squirrels (*Sciurus niger*) in the Southeastern United States. Wildlife Society Bulletin. 2015;39(3):645-650. https://doi.org/10.1002/wsb.573.

39. Zheng X, Owen MA, Nie Y, Hu Y, Swaisgood RR, L.Yan, et al. Individual identification of wild giant pandas from camera trap photos - a systematic and hierarchical approach. Journal of Zoology. 2016;300(4):247-256. https://doi.org/10.1111/jzo.12377.

40. Zhiqiang Y, Li Z. Population and acticity characteristic of wild Asian elephant in Wild Elephant Valley. Acta Theriologica Sinica. 2006;26(4):359-367. https://doi.org/10.3969/j.issn.1000-1050.2006.04.007.

41. Chaiyarat R, Youngpoy N, Prempree P. Wild Asian elephant Elephas maximus population in Salakpra Wildlife Sanctuary, Thailand. Endangered Species Research. 2015;29(2):95-102. https://doi.org/10.3354/esr00706.

42. Gupta M, Joshi A, Vidya TNC. Effects of social organization, trap arrangement and density, sampling scale, and population density on bias in population size estimation using some common mark-recapture estimators. Plos One. 2017;12(3):e0173609. https://doi.org/10.1371/journal.pone.0173609. PMID: 28306735.

43. Hedges S, Johnson A, Ahlering M, Tyson M, Eggert LS. Accuracy, precision, and cost-effectiveness of conventional dung density and fecal DNA based survey methods to estimate Asian elephant (*Elephas maximus*) population size and structure. Biological Conservation. 2013;159:101-108. https://doi.org/10.1016/j.biocon.2012.12.010.

44. Wijeyamohan S, Treiber K, Schmitt D, Santiapillai C. A Visual System for Scoring Body Condition of Asian Elephants (*Elephas maximus*). Zoo Biology. 2015;34(1):53-59. https://doi.org/10.1002/zoo.21181. PMID: 25323789.

45. Fernando P, Janaka HK, Ekanayaka SKK, Nishantha HG, Pastorini J. A simple method for assessing elephant body condition. Gajah. 2009;31:29-31. https://doi.org/10.5167/uzh-32262.

46. Ramesh T, Sankar K, Qureshi Q, Kalle R. Assessment of wild Asiatic elephant (*Elephas maximus* indicus) body condition by simple scoring method in a tropical deciduous forest of Western Ghats, Southern India. Wildlife Biology in Practice. 2011;7(2):47-54. 10.2461/wbp.2011.7.17.

47. Morfeld KA, Lehnhardt J, Alligood C, Bolling J, Brown JL. Development of a body condition scoring index for female African elephants validated by ultrasound measurements of subcutaneous fat. Plos One. 2014;9(4):e93802. https://doi.org/10.1371/journal.pone.0093802. PMID: 24718304.

48. Fan Y, Li Z. Population genetic structure and population genetic diversity analysis based on mitochondrial DNA of Asian elephant Elephas maximus in China. Acta Theriologica Sinica. 2012;32(2):90-100. https://doi.org/10.16829/j.slxb.2012.02.002.

49. Barrett SCH, Kohn JR, Falk DA, Holsinger KE. Genetic and evolutionary consequences of small population size in plants: implications for conservation. Genetics & Conservation of Rare Plants. 1991.

50. Ruping W, Lin L. The extinction vortex of small population. Journal of Biology. 2008;25(6):14-16. https://doi.org/10.3969/j.issn.2095-1736.2008.06.004.

51. Limin F, Zhisheng W, Liu L, Shaobing Y, Bin Z, Chunhua L, et al. Habitat selection in dry season of Asian elephant (*Elephas maximus*) and conservation strategies in Nangunhe National Nature Reserve, Yunnan, China. Acta Theriologica Sinica. 2010;30(1):1-10. https://doi.org/10.16829/j.slxb.2010.01.001.

52. Reddy CS, Unnikrishnan A, Asra M, Manikandan TM, R.Jaishanker. Spatial Conservation

Prioritisation of Threatened Forest Ecosystems in Myanmar. Journal of the Indian Society of Remote Sensing. 2019;47(10):1737-1749. https://doi.org/10.1007/s12524-019-01025-2.

53. Peng L, Hui W, Liu L, Jing L, Li Z. Habitat evaluation for Asian elephants (*Elephas maximus*) in Lincang: Conservation planning for an extremely small population of elephants in China. Biological Conservation. 2016;198:113-121. https://doi.org/10.1016/j.biocon.2016.04.005.

54. Yong-jing T, Zhi-sheng W, Gui-lian J, Xuan Z, Yuan H, Zheng-ling L, et al. Population dynamics analysis of Asian elephant in Nangunhe river basin. Forestry Construction. 2019;6:101-106.

55. Tang F, Du F, Sun G. Investigation report on background resources of Nangunhe National Nature Reserve, Yunnan. China Forestry Press. 2015.

56. Guo B, Lan D. Status of wildlife resources in Nangunhe reserve. Chinese Journal of Wildlife. 1999;(4):46-47.

57. Bohnett E, Riordan P, Kun S. Initial Assessment on Large and Medium Sized Terrestrial Mammal Assemblage Using Camera Trapping in Nangunhe Nature Reserve in Yunnan, China. Journal of Resources and Ecology. 2015;6(5):331-344. https://doi.org/10.5814/j.issn.1674-764x.2015.05.007.

58. Varma S, Pittet A, Jamadagni HS. Experimenting usage of camera-traps-for population dynamics study of the Asian elephant *Elephas maximus* in southern India. Current Science. 2007;6(1):81-95. https://doi.org/10.12723/mjs.10.7.

59. Silva Sd, Ranjeewa A, Weerakoon D. Demography of Asian elephants (*Elephas maximus*) at Uda Walawe National Park, Sri Lanka based on identified individuals. Biological Conservation. 2011;144(5):1742-1752. https://doi.org/10.1016/j.biocon.2011.03.011.

60. Vidya TNC, Prasad D, Ghosh A. Individual Identification in Asian Elephants. Gajah. 2014;40:3-17.

61. Sukumar R, Joshi nv, Krishnamurthy V. Growth in the Asian elephant. Proceedings: Animal

Sciences. 1988;97(6):561-571. https://doi.org/10.1007/BF03179558.

62. Goswami VR, Lauretta MV, Madhusudan MD, Karanth KU. Optimizing individual identification and survey effort for photographic capture-recapture sampling of species with temporally variable morphological traits. Animal Conservation. 2012;15(2):174-183. https://doi.org/10.1111/j.1469-1795.2011.00501.x.

63. Borchers DL, Efford MG. Spatially explicit maximum likelihood methods for capture-recapture studies. Biometrics. 2008;64(2):377-385. https://doi.org/10.1111/j.1541-0420.2007.00927.x.

64. Borchers DL, Buckland ST, Zucchini W. Estimating Animal Abundance: Closed Populations. Springer，London. 2002. https://doi.org/10.1007/978-1-4471-3708-5.

65. López-Bao JV, Godinho R, Pacheco C, Lema FJ, García E, Llaneza L, et al. Toward reliable population estimates of wolves by combining spatial capture-recapture models and non-invasive DNA monitoring. Scientific Reports. 2018;8(1):2177. https://doi.org/10.1038/s41598-018-20675-9 PMID: 29391588.

66. Gardner B, Reppucci J, Lucherini M, Royle JA. Spatially explicit inference for open populations: estimating demographic parameters from camera‐trap studies. Ecology. 2010;91(11):3376-3383. https://doi.org/10.1890/09-0804.1. PMID: 21141198.

67. Royle JA, Chandler RB, Sollman R, Gardner B. Spatial Capture-Recapture: Academic Press; 2014.

68. Royle JA, Gardner B. Hierarchical spatial capture-recapture models for estimating density from trapping arrays. In: O’Connell A.F., Nichols J.D., Karanth K.U. (eds) Camera Traps in Animal Ecology. : Springer, Tokyo; 2011.

69. Efford MG. Estimation of population density by spatially explicit capture-recapture analysis of data from area searches. Ecology. 2011;92(12):2202-2207. https://doi.org/10.1890/11-0332.1. PMID:

22352159.

70. Silveira L, Anah TAJ, Diniz-Filho JAF. Camera trap, line transect census and track surveys: a comparative evaluation. Biological Conservation. 2003;114(3):351-355. https://doi.org/10.1016/S0006-3207(03)00063-6

71. Li Z, Kang A, Lang J, Xue Y, Ren Y, Zhu Z, et al. On the assessment of big cats and their prey populations based on camera trap data. Biodiversity Science. 2014;22(6):725-732. https://doi.org/10.3724/SP.J.1003.2014.14079

72. Ling Q. Habitat Selection by Asian Elephant (*Elephas maximus*) in Nangunhe Nature Reserve, Yunnan, China. M. Sc. Thesis, Northwest University; 2007.

73. Yuming Y, Genmin W, Zhisheng Z, Zhongli W. Wild elephant population in Nangunhe reserve. Forest Inventory & Planning. 1982;02.

74. Jiasheng T, Jianhao W, Xiaolin H. Summary of Nangunhe nature reserve. Forest Inventory & Planning. 1986;01.

75. Yuming Y, Fan D. Nangunhe National Nature Reserve in China. Yunnan Science Publication. 2004.

76. Vidya TNC, Sukumar R. Social and reproductive behaviour in elephants. Current Science. 2005;89.

77. Guo B. Investigation and protection advice of wild elephant in Nangunhe reserve. YUnnan Forestry. 1989.

78. Royle JA, Nichols JD, Karanth KU, Gopalaswamy AM. A hierarchical model for estimating density in camera-trap studies. Journal of Applied Ecology. 2010;46(1):118-127. https://doi.org/10.1111/j.1365-2664.2008.01578.x.

79. Alfred R, Ahmad A, Payne J, Williams C, Ambu L, Phua M-H, et al. Home Range and Ranging Behaviour of Bornean Elephant (*Elephas maximus* borneensis) Females. Plos One. 2012;7(2):e31400.

https://doi.org/10.1371/journal.pone.0031400. PMID: 22347469.

80. Leggett KEA. Home range and seasonal movement of elephants in the Kunene Region, northwestern Namibia. African Zoology. 2006;41(1):17-36. https://doi.org/10.3377/1562-7020(2006)41[17:Hrasmo]2.0.Co;2.

81. Kumara H, Rathnakumar S, Mavatur A, Singh M. Estimating Asian elephant, Elephas maximus, density through distance sampling in the tropical forests of Biligiri Rangaswamy Temple Tiger Reserve, India. Tropical Conservation Science. 2012;5(52):163-172. https://doi.org/10.1177/194008291200500206.

82. Lichao M. Conservation genetic path to understanding the structure of Asian elephant (*Elephas maximus*) group. M. Sc. Thesis, Beijing Normal University; 2004.

83. Lisa R, Jay H. Some Observations Regarding Allomaternal Caretaking among Captive Asian Elephants (*Elephas maximus*). Journal of Mammalogy. 1987;68(2):438-442. https://doi.org/10.2307/1381494.

84. Lahdenperä M, Mar KU, Lummaa V. Nearby grandmother enhances calf survival and reproduction in Asian elephants. Scientific Reports. 2016;6:27213. https://doi.org/10.1038/srep27213. PMID: 27282468.

85. Kurt F, Hartl G, Tiedemann R. Tuskless bulls in Asian elephant Elephas maximus . History and population genetics of a man-made phenomenon. Acta Theriologica. 1995;40:125-143. https://doi.org/10.4098/AT.arch.95-51.

86. Paulraj S, Subramanian C. Skewed sex ratio in elephant population - How it may affect the demography and population growth? A case study in Mudumalai Wildlife Sanctuary and National Park, Tamil Nadu, Southern India. Indian Forester. 2000;126:1046-1053.

87. Archie E, Chiyo P. Elephant behaviour and conservation: Social relationships, the effects of poaching, and genetic tools for management. Molecular Ecology. 2011;21:765-778. 10.1111/j.1365-294X.2011.05237.x.

88. Gubbi S, Swaminath M, Poornesha H, Bhat R, Raghunath R. An elephantine challenge: Human-elephant conflict distribution in the largest Asian elephant population, southern India. Biodiversity and Conservation. 2014;23:633-647. https://doi.org/10.1007/s10531-014-0621-x.

89. Silva Sd, Webber CE, Weerathunga US, Pushpakumara TV, Weerakoon DK, Wittemyer G. Demographic Variables for Wild Asian Elephants Using Longitudinal Observations. Plos One. 2013;8(12):e82788. https://doi.org/10.1371/journal.pone.0082788. PMID: 24376581.

90. Fernando P, Wikramanayake ED, Janaka HK, Jayasinghe LKA, Gunawardena M, Kotagama SW, et al. Ranging behavior of the Asian elephant in Sri Lanka. Mammalian Biology. 2008;73(1):2-13. https://doi.org/10.1016/j.mambio.2007.07.007.

91. Srinivasaiah N, Kumar V, Vaidyanathan S, Sukumar R, Sinha A. All-Male Groups in Asian Elephants: A Novel, Adaptive Social Strategy in Increasingly Anthropogenic Landscapes of Southern India. Scientific Reports. 2019;9:8678. https://doi.org/10.1038/s41598-019-45130-1. PMID: 31273235.

92. Pokharel SS, Seshagiri PB, Sukumar R. Assessment of season-dependent body condition scores in relation to faecal glucocorticoid metabolites in free-ranging Asian elephants. Conservation Physiology. 2017;5(1):cox039. https://doi.org/10.1093/conphys/cox039. PMID: 28721215.

93. Guozheng S, Guangyuan Z, Zicheng Y, Mengjun W. Research progress and direction of human elephant conflict mitigation projects. Forestry Construction. 2019;6:5-9.

94. Sheng-dong Y, Yun D, Ai-dong L, Mang-cai W, Bin W, De Z, et al. A preliminary study of wireless infrared camera technology in alleviating the human-elephant conflict. Forestry Construction.

2019;(6):61-66.

95. Zhi-hong W, Heng-zhen Z, Wen-lan Z, Xuan Z, Wen-bian Y, Zheng-ling L, et al. An analysis of population dynamic on the Xishuangbanna Menghai-Pu'er Lancang Asian elephant population. Forestry Construction. 2019;(6):79-84.

96. Xian-ming G, Lan-xin W. Thoughts on the construction of Asian elephant food source base in Xishuangbanna. Forestry Construction. 2019;(6):30-33.

Supporting information

S1 Dataset. A simplified database of Asian elephant in NNNR.

S1 Table. Historical elephant population in NNNR estimated by different methods.

Figure 2 Click here to access/download;Figure;Fig2.tif

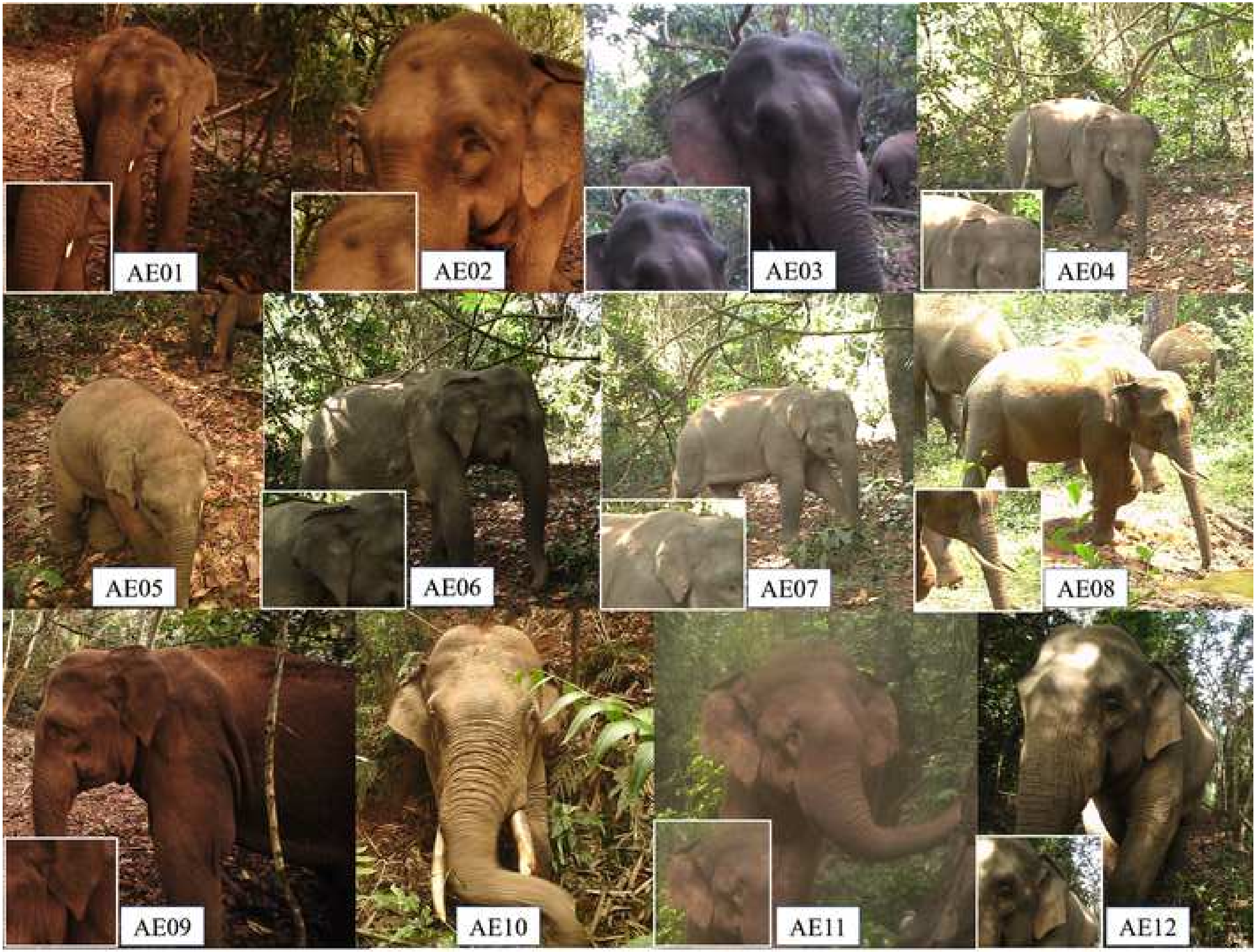

Figure 3

Click here to access/download;Figure;Fig3.tif

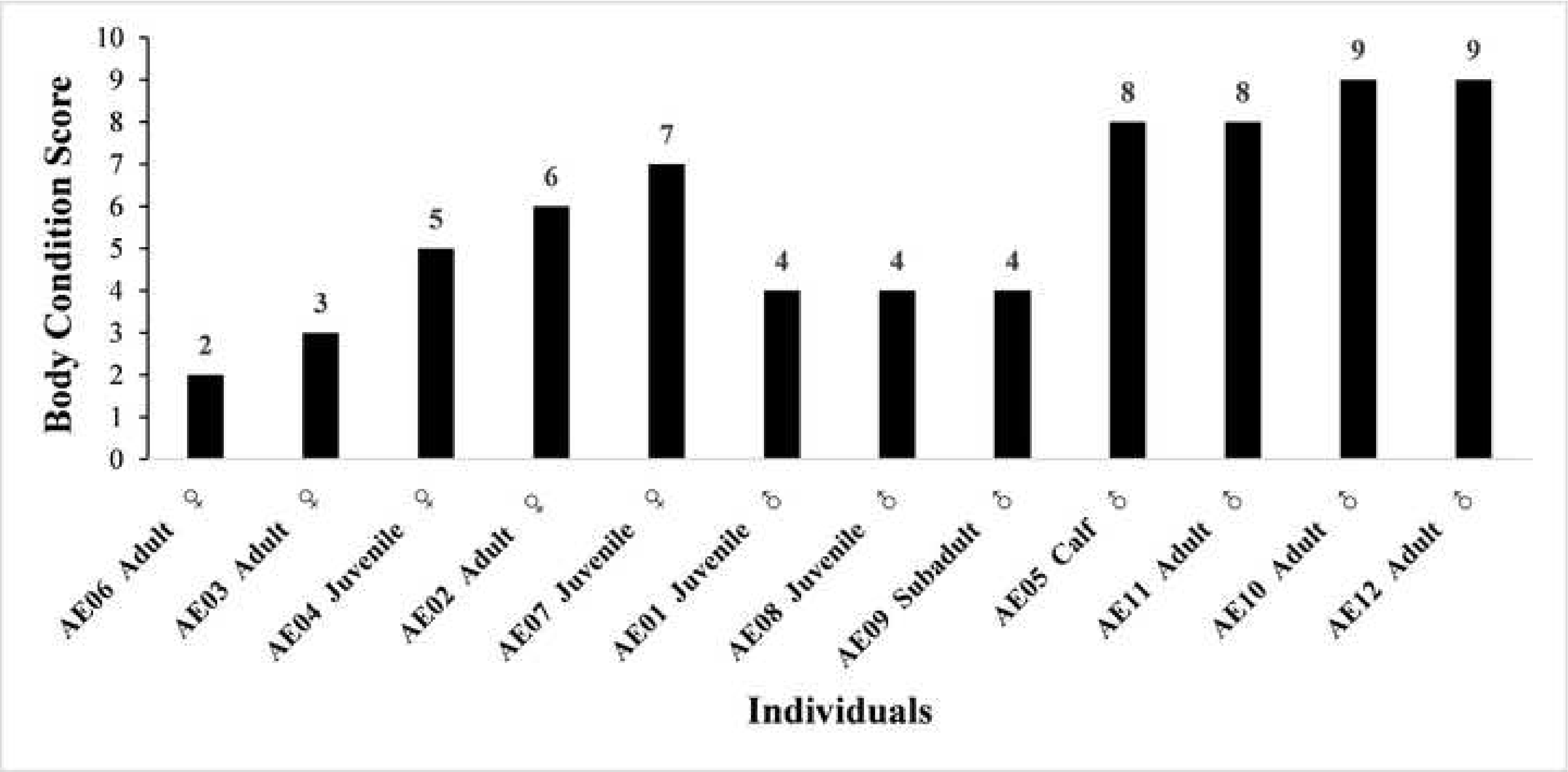

Figure 1

Click here to access/download;Figure;Fig1.tif

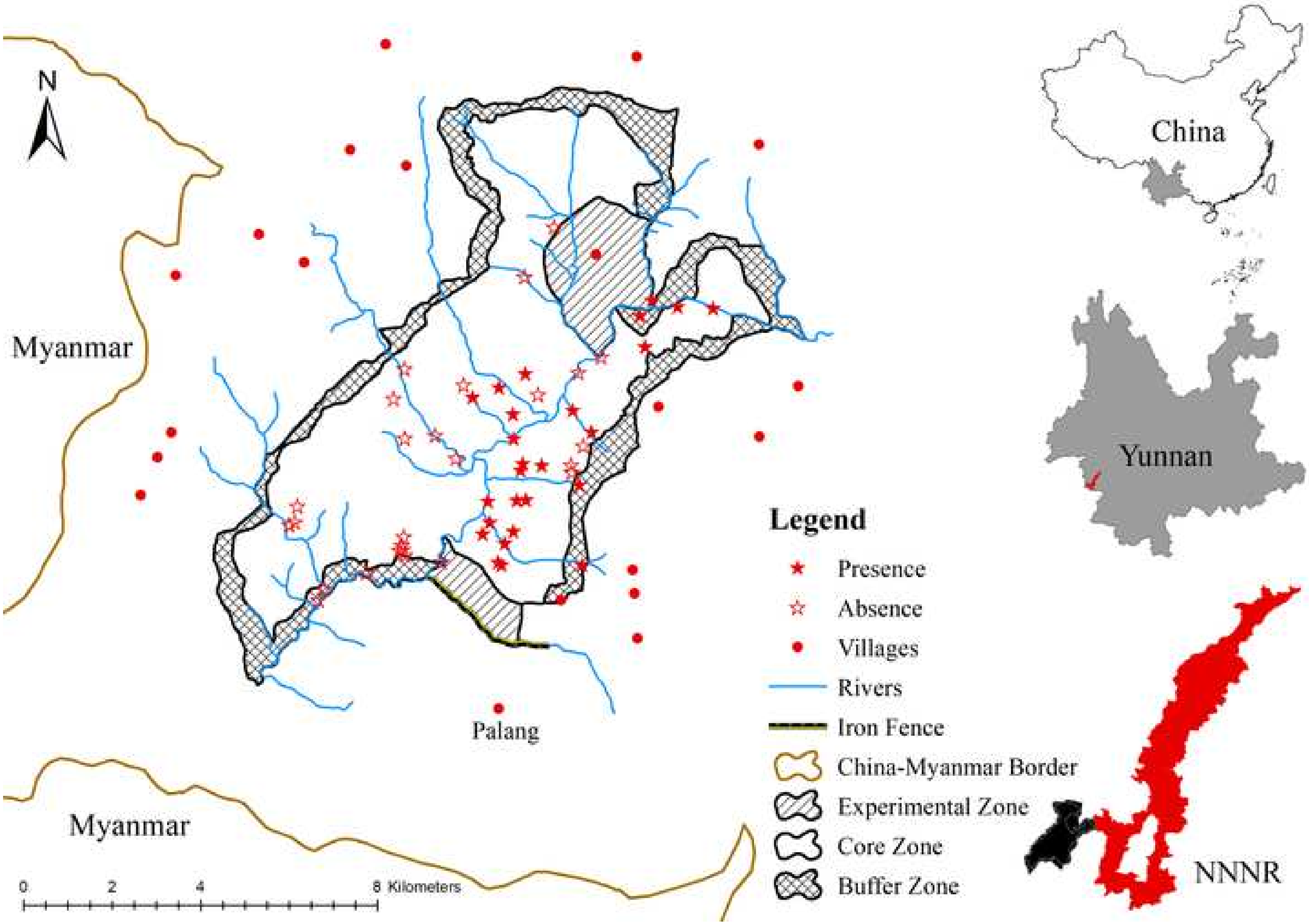

S1 Table

Click here to access/download
Supporting Information
S1 Table.docx

S1 Dataset

Click here to access/download
Supporting Information
S1 Dataset.pdf

Population structure and body condition assessment to inform conservation strategies for a small isolated Asian elephant population in southwest China

Yakuan Sun[1¶], Ying Chen[2¶], Juan José Díaz-Sacco[1¶], Kun Shi [1,3¶*]

[1]School of Ecology and Nature Conservation, Beijing Forestry University, Beijing, China

[2]School of Biological Science, The University of Hong Kong, Hong Kong, China

[3]Eco-Bridge Continental, Beijing, China

[*]Corresponding author

E-mail: kunshi@bjfu.edu.cn (KS)

[¶]These authors contributed equally to this work.

Abstract

The Asian elephant (*Elephas maximus*) population in Nangunhe National Nature Reserve represents a unique evolutionary branch in China, which has been isolated for more than twenty years from other neighboring populations in Myanmar. The scarcity of information on population structure, sex ratio and body condition makes it difficult to develop effective conservation measures for this elephant population. Twelve individuals were identified from 3,860 valid elephant images obtained from February to June of 2018 (5,942 sampling effort nights) at 52 camera sites. Three adult females, three adult males, one subadult male, two juvenile females, two juvenile males and one calf male were identified. The ratio of adult females to adult males was 1:1, and the ratio of reproductive ability was 1:0.67, indicating the scarcity of reproductive females as an important limiting factor to population growth. A population density of 5.32 ± 1.56 elephants/100 km^2 was estimated ~~by~~ using Spatially Explicit Capture Recapture (SECR) models. The health condition of this elephant population was assessed by using an 11-points scale of Body Condition Scoring (BCS), ~~method~~ which showed that this population had a medium body condition with an average of 5.75 (n=12, range 2-9). This study finds that this isolated population has an extremely small size and an inverted pyramid age structure and therefore the population is at high risk of extinction. As such, this population should be of top conservation priority among the few remnant populations in China. The database established in this study can provide researchers with valuable information to set up efficient conservation schemes.

Introduction

The Asian elephant (*Elephas maximus*), as the largest terrestrial mammal in Asia, is considered an ecosystem engineer because it can modify its habitat and aid in seed dispersal [1, 2]. The conservation of this umbrella species contributes to the preservation of ~~most of the~~ biodiversity in the ecosystems it uses, especially in tropical moist and in tropical dry broadleaf forests of Southeast and South Asia [3-5]. It is also considered a flagship species that can attract interest for the protection of biodiversity [6]. It is a Class I Key Protected Species in China and listed as Endangered under the IUCN Red List [7, 8]. China sustains a population of c. 300 wild Asian elephants which are divided into seven geographic populations [9, 10]. Most Asian elephant populations are located in Mengyang, Mengla and Shangyong areas of Xishuangbanna National Nature Reserve, while there are also a few populations in Simao, Jiangcheng and Lancang counties of Pu'er city and one small isolated population in the Nangunhe National Nature Reserve (NNNR) of Lincang city [9, 11].

The conventional methods used for tracking Asian elephants in ~~the~~ dense forest are difficult to accomplish and usually fail to provide accurate population numbers and age structure [12, 13]. Although it is relatively easy to detect elephant signs, such as dungs or footprints, by using sign surveys [14], it can be difficult to identify individual Asian elephants since signs are highly inaccurate and unreliable [15]. Individuals can be identified using non-invasive DNA methods, but it requires relatively fresh samples and the lab costs can be quite high; moreover, the population size can only be determined with genetic capture-mark-recapture methods, which can be time and labor intensive [16, 17]. Furthermore, since elephant calves have lower defecation rates, this

method can underestimate the number of young individuals in a population [18].

Infrared trigger camera technology can make up for the above-mentioned disadvantages [19, 20]. Infrared cameras cause minimal disturbance to the study animal and its habitat, can be used for long working hours, can withstand harsh weather conditions, and are relatively cheap [19, 21, 22]. Infrared cameras are widely used to obtain ecological baseline surveys of protected species [23, 24], to monitor endangered wildlife [25], to study habitat utilization [26], to observe animal behavior [27], and to study the mechanisms of coexistence in sympatric species [28, 29]. Moreover, infrared cameras can also be used to estimate population size and density by using Spatially Explicit Capture-Recapture models (SECR) [30-33]. This technology has been used extensively to estimate the population density of multiple species, especially those ~~which~~ possessing easily observed markings, such as the snow leopard (*Panthera uncia*), the leopard cat (*Prionailurus bengalensis*), the Sumatran tiger (*Panthera tigris* ssp. Sumatrae), and the serval (*Leptailurus serval*) [33-36]. For species without natural markings, identification can also be conducted according to morphology, such as with the Asian elephant, the giant panda (*Ailuropoda melanoleuca*), and the fox squirrel （*Sciurus niger*) [37-39]. Throughout the Asian elephant range, there have been a number of studies that have identified individual elephants by directly photographing them or by obtaining images from infrared cameras [17, 37, 40, 41].

Population structure and density estimation are essential features that can be used to study the ecology of wildlife populations [42, 43]. The Body Condition Scoring (BCS) is an index of ~~an~~ animal’s health that reflects the quality of the environment in which

the animal lives [44]. It can be used in conjunction with population structure and density estimation to obtain a more accurate picture of the health of a population. Furthermore, it can be used for both longitudinal and cross-sectional studies, is comparatively inexpensive, and does not require a high technical capacity [45]. While BCS is individual-based, it is most meaningful when applied to a population, as an early indicator of the impact of management actions on the average health condition of a population [45, 46]. For Asian elephants, the BCS obtained through photographs can be as accurate as ultrasonic subcutaneous fat measurement, thus providing a safer and non-invasive method to evaluate the health condition of wild populations [45, 47].

~~Throughout the Asian elephant range, there have been a number of studies that have identified individual elephants by directly photographing them or by obtaining images from infrared cameras~~ [17, 37, 40, 41]~~. In China, population studies have mainly been concentrated in Xishuangbanna National Nature Reserve~~ [17, 40]~~, with a few conducted in NNNR, focusing on population size estimation, but not on the density or the health of its populations.~~

In China, population studies have mainly been concentrated in Xishuangbanna National Nature Reserve [17, 40], and previous studies were limited to studying population sizes and none have assessed the health condition of the population[13, 48].

~~Isolated populations are vulnerable to changes in the external environment and thus, their conservation should be a top priority .~~ Although it is a unique evolutionary unit in China, the isolated elephant population in NGH has been poorly studied and the lack of scientific data and information on population structure, relatedness, and health

status prevent the development of efficient conservation strategies. Studies conducted on the elephant population in NNNR concluded that it was a separate evolutionary unit isolated from other geographic populations [13, 48], thus lacking genetic connectivity. Although other elephant populations can be found in nearby Myanmar, the forest degradation in the China-Myanmar border area has led to the isolation of this elephant population since 1997 [9, 51, 52]. Additionally, this population is facing threats from habitat loss, fragmentation and illegal hunting [51, 53, 54]. Although conservation interventions have been proposed to rescue this population from extinction, the lack of scientific data and information on population structure, relatedness, and health status prevent the development of efficient conservation strategies.

Isolated populations are vulnerable to changes in the external environment and thus, their conservation should be a top priority [49, 50]. However, previous studies on Asian elephants in China were limited to studying population sizes and did not assess the health condition of the population [13, 54]. An accurate understanding of the current situation of the local elephant population in NNNR, especially its structure and health condition, is key to establishing a baseline for the development of more effective protection and management schemes.

In this study, our aim was to determine the size, density, demographic structure and health condition of the NNNR Asian elephant population by using infrared camera trapping. We sought to understand which factors might have affected its population size in recent years in order to develop targeted and effective protection strategies. Furthermore, our approaches can be applied to other elephant populations with further

implications for global Asian elephant conservation.

Material and Methods

Ethics Statement

Both National Forestry and Grassland Administration of China and Yunnan Forestry and Grassland Administration reviewed all sampling procedures and approved permits for the work conducted in NNNR. Non-invasive methods were applied and approval from an Institutional Animal Care and Use Committee or equivalent animal ethics committee was therefore not required.

Study area

The study area is located in the western part of the Nangunhe National Nature Reserve, Yunnan, where elephants are found [51, 53] (Fig 1). It occupies 70.82 km^2 at latitude 23°13'- 23°19'N, longitude 98°54'- 99°05'E, and the altitude ranges from 510 m to 1747 m. Rainfall is highly seasonal, with a dry season between November and April, and a rainy season between May and October. Mean annual precipitation is 351.3 mm in the dry season and 1983.3 mm in the rainy season, while temperature varies at different altitudes. The average monthly temperature range is hottest in July, varying between 12.3-25.5 ℃ and it is coldest in January with values between 2.8-14.3 ℃ [55]. Vegetation and climate vary across elevation zones. Tropical forest and tropical monsoon forest are located at altitudes below 600 m, middle elevation forests are dominated by subtropical monsoon evergreen broad-leaf forests, and evergreen broadleaf forests are found above 1700 m [53]. Bamboo forest, shrub and grasslands

are scattered throughout this area. Deforestation along the China and Myanmar border has disrupted the connectivity between elephant populations that are found in both countries [51]. Furthermore, an iron fence in the southern part of reserve is used to protect the surrounding villages from wildlife (Fig 1).

Fig 1. Map of study area in NNNR showing the camera sites with ~~elephants~~ or without elephants.

Camera trap set-up

We carried out the survey for four months between February and May 2018 using automatically triggered camera traps (Ltl Acorn 6210). There was a total of 52 cameras stations which successfully recorded 43-129 camera trap nights in the field (Fig 1). The total camera trapping effort was 5,942 trap nights (114.27±19.83). All the stations were located in evergreen broadleaf forest, broadleaf and bamboo forest, bamboo forest, pine forest and nearby ~~the~~ brush forest, which ~~represented~~ represents the preferred habitat of elephants in NNNR. We systematically deployed camera traps along the animal trails and forest gaps where there were signs of elephants, including lying sites, foraging sites, footprints, dungs and urine marks. ~~Cameras had~~We attempted to ~~be~~ deploy~~ed~~ cameras throughout the reserve ~~in~~ following a 1 km^2 grid~~ formation~~. We increased the number of cameras in the grids with many elephant signs, ~~but~~ always with a minimum of 200 m between any two cameras. We avoided the northwestern part of the reserve as the topography is tough, with steep cliffs of up to 1747 meters, and historically~~,~~ there have not been any records of elephants in this area [56, 57].

Cameras were strapped onto trees at approximately 1.5 m above the ground with

the sensor parallel to the ground to maximize the extension of the detection zone. The positions of all the camera sites were recorded by GPS. To avoid blank images caused by sunlight, the cameras were never placed facing east. Camera traps were hidden as much as possible to reduce the disturbance to wildlife and the probability of being stolen. Camera traps were set to operate 24 hours per day and programmed to take three photographs and one ~~12~~ 12-second video as a sequential without delay, registering the date and time for each exposure. At each site, a camera trap was operated continuously for a maximum of four months. SD cards and batteries were replaced every month. We recorded the time of installation and retrieval of each camera and calculated the total duration of sampling.

Individual identification

The images obtained from the camera traps were categorized to independent events, which were defined as ~~a~~ “sequences”. Each sequence ~~is~~ was defined by a set of images adjacent in time. For an elephant unit (defined as all or the maximum number of individuals in a group), the sequence ~~includes~~ included the images from the first ~~one~~ individual entering the field to the last one exiting it, making it possible to identify different individuals based on distinct features. For solitary individuals, ~~the~~ a sequence ~~is~~ was a series of captured images of same individual within a continuous time period [58]. Only images with sufficient quality, clarity and position of the elephant in the frame were used for individual identification [58]. Here, quality referred to how usable an image was for age and sex classification; clarity indicated whether the elephant was “captured” in good light or that the flash reached the individual, and the image was in

focus and not over or under-exposed; and position referred to the occurrence of the elephant within an 'optimal distance and angle' from the camera [41, 58]. The age of each animal was broadly classified into adult, subadult, juvenile and calf using approximate shoulder height as recommended by previous studies, and gender was mainly determined by secondary sexual characteristics [59-61] (Table 1). We defined the age structure as the ratio of adults, subadults, juveniles and calves while the reproductive ability was the ratio of age structure between adult and juvenile females [41], due to the absence of subadult and calf females in this reserve. Male elephants living completely or mostly outside the herd were classified as solitary males [12]. Based on individual events, a capture-history event database was established to record the individuals and their characteristics. Morphological characteristics including facial bones, ear, tusk, back and tail hair, old scars and injuries, as well as physical characteristics related to sex and age classes, confirmed the reliability of identification results [17, 37, 40, 62] (Table 1).

Table 1. Morphological characteristics used for individual identification of elephants photographed in NNNR, between February and May 2018.

Characteristics	Categories	Description
Age	Adult (>15years)	With the highest shoulder height
	Subadult (5-15years)	Shoulder height reaches an adult's eyes
	Juvenile (1-5years)	Shoulder height reaches half the height of an adult
	Calf (0-1year)	Below an adult's knee
Sex	~~Adult f~~Female	Without obvious tusks but with prominent breasts, belly swelling or the presence of nursing offspring

	Adult mMale	With obvious tusks or visible testicles or penis, and/or obvious tusks but without prominent breasts, ; the presence of penis for tuskless individual
	Subadult male	Without obvious tusks but with visible penis
	Juvenile female	Without obvious tusks
	Juvenile male	With obvious tusks
	Calf male	Only theWith visible testicles or penis were visible
Age	Adult (>15years)	With the highest shoulder height
	Subadult (5-15years)	Shoulder height reaches an adult's eyes
	Juvenile (1-5years)	Shoulder height reaches half the height of an adult
	Calf (0-1year)	Below an adult's knee
Facial bones	Wisdom tumor	Size
	Bones between the eyes	Extent of prominence
	Upper margin of eyes	Extent of depression
Tusks	Length	Very short/short/long compared with trunk
	Thickness	Thick/normal/slender
	Shape	Straight/bent
	Crack	Yes/no
Ear	Upper edge	Fold/unfold
	Lobe	V-acute/U-rounded
	Lobe tear	Yes/no
	Ear hole	Yes/no
Tail brush	Standard	Present/both sides/both-continuous
	Nonstandard	Absent/single side/both-discontinuous

Density estimation

The elephants in NNNR belong to a small isolated population without any migrants, thus conforming to the "close population" assumption of SECR [63]. The model accounts for the movement in density estimation by combining a state model and an observation model, which describe how the observed data were generated [63]. The state model describes statistically the mechanisms determining the distribution of the

animals in the study area, while the observation model quantifies the probabilities of detection or capture, given the searched region, their locations and characteristics at different sessions [64]. Basically, SECR are generalized linear mixed models that assume that ~~every~~ each individual i in the population has its own unobserved activity center S_i, and all the activity centers $S_i \cdots S_N$ are distributed across the study area [65, 66].

The encounter rate of an individual i with a given trap j located at X_j is a monotonically decreasing function of the distance from S_i to j. Y_{ij}, the number of times individual i is detected by trap j during a sampling occasion, is a random variable following a Poisson distribution and with mean λ_{ij} [67]:

$$Y_{ij} \sim \text{Poisson}(\lambda_{ij})$$

For an elephant i captured by a trap j, the model assumes a log-linear form [68]:

$$\text{Log}(\lambda_{ij}) = \text{Log}(\lambda_0) - \left(\frac{1}{2\sigma^2}\right) \|S_i - X_j\|$$

Here, λ_0 is the baseline encounter probability, the function $\|S_i - X_j\|$ is the distance between the activity center and the trapping station, and σ is the Gaussian scale parameter for the distance function between activity centers and trap locations; assuming a bivariate normal model of space use, sigma can be used to calculate a 95% activity area radius.

SECR models estimate the density of animal activity centers in an area large enough so that animals residing beyond it have a negligible chance of being detected [63]. Considering the small size of the elephant distribution, with the east and west

boundary being no wider than 10 km, we therefore defined our study area as extending 5 km beyond all camera stations, corresponding to an area of 255.8 km^2 [69].

SECR 3.1.5 package in R 3.6.1 was used for density estimation. Data were prepared as an R object of class “capthist”. This object included both the capture data and the detector layout. The file named “capture histories” contained data on individual ~~identification~~identified at each sampling~~, the~~ occasion ~~(each sample time, that is event here)~~from~~,~~ the specific detector (camera~~ ID~~). The detector file contained data on cameras ID, and x, y coordinates. Five different sessions, corresponding to each of the months and one corresponding to the whole survey period, were used to run the model to see the estimation accuracy under different detection efforts.

Body condition scoring

We established a database of each identified individual and the images with the best angle of each individual elephant were selected to score the body condition. Compared with previous studies, our target population size was small, and the sample size was limited so we used a simple method which can rapidly and more easily be applied to wild elephant populations. In this study, we applied a BCS method that focuses on six physical characteristics and ranks the elephant´s body condition according to an 11-points scale [44, 45]. Five of the clearest images for each physical characteristic were selected to assess the body condition. Thirty images were used to calculate the comprehensive scores for each individual. This scoring method is based on the extent of visibility of depressions around bone structures. Depressions around bones become visible as an animal loses its subcutaneous fat deposits and muscles in

the region concerned, thereby making bones appear more prominent. Skull, pectoral girdles in shoulders, vertebral column, ribs and pelvic girdles are prominent bone structures. Thick rolls of skin that fold below the neck can accumulate fat, and thus, also represent a criteria of good body condition. We presented a scale of 5 reference scored elephants description of 1, 3, 5, 7, 9, with numerically higher values corresponding to better body condition (Table 2). However, the range of scores extended from 0 to 10 due to the possibility of greater variation. For example, if all the ribs of an elephant were visible, it meant that this individual presented a worse body condition than 1 and thus, it would be assigned 0. If an individual presented a fatter condition than 9 and had very thick rolls of skin fold below neck, it would be assigned a score of 10. Scores ranging from 0-3 were considered as poor, 4-7 were considered medium and 8-10 were considered as good [46]. Pearson correlation coefficient (r) was used to study the relationship between BCS and various covariates such as sex, age, and whether an individual was solitary or in a herd.

Table 2. Diagnostic characteristics pertaining to scores in photographic scale.

Score	Characteristics
1	Ribs from shoulder to pelvis are visible, some ribs prominent (spaces in between sunken in)
3	Some ribs visible (spaces in between not sunken in), shoulder and pelvic girdles prominent
5	Ribs not visible, shoulder and pelvic girdles visible
7	Backbone visible as a ridge, shoulder and pelvic girdles not visible
9	Backbone not visible or difficult to differentiate and pelvic bones not visible

Results

Population size and density

A total of 70,902 images were obtained, among which 7.34% (5,207) were Asian

elephant data. There was a total of 134 independent events, including 40 events of elephants in a herd and 94 events of solitary elephants. Excluding the image data that could not be used for individual identification, there were 3,860 valid Asian elephant images belonging to 85 independent events (26 events of individuals in a herd and 59 events of individuals). Generally, all herds and individuals had similar frequencies of recapture histories during the study period, particularly herds. However, solitary elephants were image-captured with a minor difference: a minimum of 16 capture times ~~of~~ for AE12 compared to a maximum of 25 capture times ~~of~~ forAE10 (Table 3).

Table 3. Number of capture events ~~for a given~~per individual per month.

Individual No.	Age	Gender	Tusk	Type	Number of Capture events				
					Feb.	Mar.	Apr.	May	Total
AE01	Juvenile	Male	Yes	Herd	2	6	5	8	21
AE02	Adult	Female	No	Herd	3	5	6	6	20
AE03	Adult	Female	No	Herd	3	5	6	7	21
AE04	Juvenile	Female	No	Herd	2	6	5	7	20
AE05	Calf	Male	No	Herd	3	6	8	5	22
AE06	Adult	Female	No	Herd	4	6	8	5	23
AE07	Juvenile	Female	No	Herd	4	6	9	6	25
AE08	Juvenile	Male	Yes	Herd	4	7	6	7	24
AE09	Sub-adult	Male	No	Solitary	3	9	2	9	23
AE10	Adult	Male	Yes	Solitary	2	3	13	7	25
AE11	Adult	Male	No	Solitary	3	5	8	5	21
AE12	Adult	Male	No	Solitary	2	6	1	7	16
Total	-	-	-	-	35	70	77	79	261

A total of 12 individuals were identified among these valid independent events (Fig 2; S1 Dataset). The elephant population size in each session was consistent, except in February. Moreover, the population size estimated by SECR was 12.00 ± 0.71 (with a 95% confidence interval of 12.00-12.11; Table 4). The estimated density by SECR was 5.32 ± 1.56 elephants/100km^2 (with a 95% confidence interval of 3.02-9.36). In

camera-trapping session at monthly intervals, April had the lowest density estimation at 6.07 ± 1.79 elephants/100km^2, while May had the highest density estimation at 24.43 ± 7.20 elephants/100km^2. The movement parameter σ was twice as large in April (6643 m ± 2356 m) than in May (3771 m ± 1471 m).

Fig 2. Asian elephants individually identified in NNNR, China. Each individual's most distinguished characteristic was emphasized, except easily identified AE05 and AE10, the only calf and adult male with tusks.

Table 4. Population density estimation by using SECR models under five sessions.

Sessions	Occasions	Density			λ_0		sigma		Population size		
		estimate	SE.estimate	95% CI	estimate	SE.estimate	estimate	SE.estimate	estimate	SE.estimate	95% CI
February	11	10.71	3.31	5.91-19.38	0.15	0.04	2983	664	14.00	2.19	12.35-23.43
March	26	6.57	1.94	3.73-11.58	0.07	0.02	4193	700	12.20	0.87	12.00-17.95
April	22	6.07	1.79	3.45-10.70	0.02	0.004	6643	2356	12.05	0.75	12.00-16.87
May	26	24.43	7.20	13.87-43.03	0.01	0.003	3711	1471	12.03	0.74	12.00-16.48
Total	85	5.32	1.56	3.02-9.36	0.02	0.003	6474	1065	12.00	0.71	12.00-12.11

Demographic structure

We identified an inverted pyramid age structure for this population: three adult females, three adult males, one subadult male, two juvenile females, two juvenile males and one male calf, with a sex ratio of 1:1.4 using females as a reference (Table 4). The three adult males comprised one tusker and two without tusks (Makhnas), giving an adult tusked to tuskless male ratio of 1:2. The subadult male was also a makhna,

increasing the tusked to tuskless male ratio to 1:3. The ratio of reproductive ability was 1:0.67.

The social network was comprised by judging from camera trap data is AE01-AE08 that formed the joint-family unit, while with individual calf AE05, adult female AE06 and juvenile female AE07 as forming a mother-calf unit which occasionally joined and departed from the large family unit. AE06 and AE05 were in a mother-child relationship since there were images of the calf nursing from the female. The solitary subadult male AE09 and adult male AE10 were observed to occasionally join the family unit.

Body condition scoring

The distribution of BCS for the population had an average of 5.75 (n=12, range 2-9; Fig 3). Body condition evaluation revealed that 33.3% of the elephants were in a good health body condition, while 50% and 16.7% were in medium, and poor health body conditions, respectively (Fig 3). Age was not significantly correlated to BCS (r=0.03), but sex was (r=0.42), as adult males had better body conditions than adult females. The body condition of the only three fertile females in this population was not good. The adult female AE02 had a BCS just above the average score, while the other females, AE03 and AE06 were in poor health body conditions with a low BCS of 3. Being solitary or in the herd played more important roles in the BCS (r=0.53) than sex. Individuals that were part of the herd had a lower BCS than solitary individuals.

Fig 3. Body condition score of the 12 individuals identified in NNNR.

Discussion

Population size and density

Accurate assessment of population size is essential for species conservation and management [70]. Identification from captured images was robust enough to identify individuals [71], unlike dung and footprints which are easily influenced by slope, soil quality and climatic factors [72]. This study used cameras installed in the elephant preferred habitats that covered most of the western part of the reserve, which provided a higher chance of obtaining the data reflecting the real population, leading to a more reliable population assessment ~~as~~ than in the study that used drone surveys [54]. ~~Historical overview of the research on this population estimated by several different methods may generate inevitably a bias in direct comparison, the NNNR population showed a population size fluctuating around 20 individuals, and a tendency towards a slight population decrease~~Previous studies have estimated this population to be around 20 individuals. Although population estimation using different methods could generate bias in direct comparison, research has shown that this elephant population has remained small during the last few decades~~-~~[54, 72-74] (S1 Table). ~~Nowadays, the distribution for this population still remains~~ distributed ~~in the western part of the NNNR Cangyuan county [54, 73, 75] (Fig 1). Therefore, we assumed that there is space available in NNNR to allow for this small population to reach its highest ever recorded population size of 24-25 individuals [56, 73].~~ The NNNR population is an extremely small and isolated population, compared with other elephant populations found around the world [3, 9, 10, 12]. Historical illegal hunting has had a direct impact on this population with a total of 15 unnatural deaths since 1987 (Table 5). Additionally, 3 of

4 elephants found dead ~~the high mortality of immature elephants (75%) among these unnatural deaths~~ since 2003 have been immature individuals(Table 5).~~, as well as~~ Moreover, the fact that elephants have a long adolescence and long inter-birth intervals means it takes time for NGH elephants to recover and they are more vulnerable to local population extinction [12, 76], especially if young individuals are being poached or dying from other unnatural causes. All these facts result in the current small population size that could lead to the extinction of this local population.~~have had potentially negative effects on this populations growth.~~

Table 5. Unnatural deaths of Asian elephant since the establishment of NNNR.

Year	Number of individuals	Cause of death	Data resources
1987	Two: One female and one male	Illegal killing	[55, 77]
1988	Four: Two adult males, one adult female and one calf	Poaching, electrocuting and unknown reason	[51]
1995	One male	Unknown	[54]
1996	One male	Vengefully hunted by local people	[53]
1997	Three: One female, one male and a calf	Unknown for the calf, the female and male were killed	[55]
2003	A Subadult male (about 10 years old)	Unknown	[55]
2008	One subadult male	Unknown	Communication with local residents and rangers
2011	One juvenile	Unknown	[55]
2016	One adult male	Unknown, but the tusks show visible saw marks	Communication with local residents and rangers

SECR modelling has become a powerful tool to estimate the small population sizes like the one in NNNR [32]. The model defines the activity center according to the range

of the species activities [78], so it is easy to overestimate the population density in a short survey period, which was confirmed by monthly estimates in this study (Table 4). We first used the single monthly session to run the SECR model, with which we obtained a significant difference in density estimation. The SECR model simulated a larger movement buffer (sigma=6643 m) in April, which resulted in the smallest population density. May presented the highest density because there were 8 cameras that captured elephants, and the distance between each one was less than 800 m, which is smaller than ~~its~~ their daily movement distance [79, 80]. The instability of single month session results by SECR~~,~~ prompted us to use the four months data as the fifth session to run the model. We thus obtained the smallest standard deviation, which indicated that the result was closer to the actual value [41].

The population density results of the NNNR elephant population, as an unique evolutionary clade in China, can be used to compare with results from other populations in order to improve our understanding of elephant ecology and develop more detailed management and conservation plans [81]. However, with no other SECR analyses conducted on other populations in China, we used the crude density estimation (estimated population number/study area) to draw comparison between the NNNR population and others in Yunnan [41]. The crude density estimation of Asian elephants in NNNR was the highest at 16.9 elephants/100 km^2 due to its smaller area of distribution, compared with that in Shangyong, Mengla, and Menyang with a density of 10.68, 4.26, and 1.85 elephants/100 km^2, respectively [13, 17]. The relatively high population density ~~This is concerning as a high population density could~~might ~~–~~lead

to more intensive competition among individuals for limited natural resources, and even greater human-elephant conflict as elephants resort to crop raiding for food, which could be further studied in the future .

Population structure in NNNR

Although the juveniles in this population accounted for 33% of the population, higher than the minimum of 20% of the population considered to be needed for it to be self-sustaining in a short period of time ~~[18],~~ [18]. Previous study already confirmed these individuals were related to each other[82]. This phenomenon of inbreeding will be more serious ~~is inevitable~~ in the near future due to the small population size.

We attempted to identify the relationship between individuals in the family unit through images captured by camera trap, since the allomaternal care or mother-like behavior directed by females are prevalent in the Asian elephant social structure [83, 84]. We could not match adult females with their offspring in the groups relying on the observed behaviors, even though we captured intimate interaction between AE02 and AE08, as well as between AE03 and AE04. In recent years, the death of adult females has not been recorded in the reserve (Table 5). Therefore, we assumed that all the current juveniles came from these three adult females. Genetic studies are urgently needed to better understand the lineage and degree of inbreeding depression in the NNNR population.

There were two tuskless juveniles that had ~~similar~~ body heights ~~as~~similar to that of the juvenile males identified by their apparent tusks and ~~these~~ that were identified as females. It ~~However, it~~ should be noted that males in some populations tend to be

tuskless for survival due to ~~the~~ intensive historical poaching pressure [12, 85]. The high proportion of makhnas in NNNR identified ~~from~~ in this ~~investigation~~ research could indicate that these juveniles ~~that were~~ identified as females might in fact be males. Since tuskless males are common in this population, it would be helpful to conduct genetic studies on this population that can confirm the sex of these two juveniles.

The extremely ~~Unbalanced~~ unbalanced sex ratio that skewed to males is not beneficial for population growth [86]. Although more males in social mammals could maintain the effective population size and increase genetic diversity, elephants' ability to recognize kin and avoid inbreeding might mitigate the loss of genetic diversity which cause local extinction in small population [87]. ~~Currently, only females AE02, AE03 and AE06 are of reproductive age, but the latter was nursing a calf, leaving only two sexually active females in the reserve. The reproductive ratio of this small population was relatively high compared to the findings in Ruhuna National Park, Sri Lanka~~ [88]~~, and Salakpra Wildlife Sanctuary, Thailand [41], since females~~ Females do not enter the breeding period until they are 10-14 years old and as such these two juvenile females in the NNNR population still needed 3-7 years to become mature [9, 12, 89]. Currently, only females AE02, AE03 and AE06 are of reproductive age, but the latter was nursing a calf, leaving only two sexually active females in the reserve. In either case, the scarcity of fertile females would be the primary constraint on population growth due to their long birth intervals.

Health condition

We chose a simple but accurate body condition method to assess the health of this

small population. Our results showed a higher body condition of adult males than adult females~~, which was also observed in another elephant population in Southern India [46]~~. This may be because adult males are known to have larger home ranges than adult females ~~and immature males~~ [80, 90], and are more tolerant to human disturbance [91]~~.~~: that ~~Which~~ means they can explore more frequently areas near the reserve boundary to obtain more food resources ~~frequently~~, where intensive anthropogenic activities like hunting, ~~gathering,~~ fishing and collecting have also been recorded by cameras. Additional nutritional stress from lactation, like AE06, was another reason that could have resulted in an even poorer body condition than males [46]. Furthermore, s~~S~~olitary elephants, which were males, could have also had a higher BCS than the individuals in the family units because at least one of the herds could have had an even more reduced area of movement due to the presence of a calf [90]. ~~Furthermore, additional nutritional stress from lactation for AE06 could have resulted in an even poorer body condition [46].~~ Additionally, the other two adult females both presented a medium health condition, which would not be conducive for reproduction [44].

Most of the individuals (66.7%) had medium or poor body conditions during the survey period, which corresponded to the dry season. Since the quantity and quality of food and availability of water are the primary factors determining the body condition of free-ranging elephants [44], both female and male individuals show an obvious thinner body condition during the dry season compared to the rainy season [92]. We did not have the opportunity to conduct camera trap surveys during the rainy season since many cameras failed to work in this season. Additionally, the rainy season coincides

with the harvest period. In the past, especially in recent years, NNNR elephant-damage compensation data has shown that the elephants forage for corn and rice during the rainy season from ~~the month of~~ July to October (Li Zhimin, personal communication~~data from the compensation documentary of the reserve bureau~~). Therefore, we ~~predict~~ assume that at least males in this population ~~is~~ are foraging ~~off of~~ crops to supplement its diet. ~~Thus, f~~Future studies should focus on determining ~~if~~ how crops ~~could~~ contribute to ~~improve~~ improving the body condition of elephants during the rainy season.

Moreover, the body condition assessment in this study could have implications for the research and conservation of other elephant populations. Since the vast majority of Asian elephant populations, especially in China, are found outside of reserves and near cropland, the health of these populations are likely heavily influenced by access to crops [93]. Massive amounts of data have been collected by camera traps and drones in these areas [94, 95], but none have been used to determine the health of ~~the~~ elephant populations. We have ~~proven~~ shown that using images ~~for~~ to assess BCS ~~to be~~is a simple and fast method for determing the health of free-ranging elephant populations. Therefore, future studies should consider analyzing the body condition of ~~different~~ regional Asian elephant populations from these data sets to ~~be used as an~~obtain early ~~indicator~~ indications of the health of individuals and quality of food resources in their habitats [45]. This in turn can be used to guide conservationists and policymakers to evaluate the necessity for interventions ~~and define regions for~~, including the restoration of suitable habitat ~~restoration~~ [44].

Conservation recommendations

To maintain this extremely small isolated elephant population, the long-term placement of cameras in NNNR could be an efficient approach of monitoring to obtain is needed to get valuable data for the study ofon demography, fecundity and health body condition variation across seasons and yearsstatus . Molecular biological approaches are also needed in further studies to determine the kinship and genetic health status of this population .

The results of this study match those observed in earlier studies have reconfirmed that the enlargement ofproviding more suitable habitat is urgently needed to ensure an adequate supply of food is available for this isolated population during the dry season [13, 51, 53]. Attention should be paid to the low body condition in the dry season, and a follow-up survey should be carried out in the rainy season as soon as posible to verify the health status of this elephant population throughout the year. Therefore, the short-term objective to increase the survival quality f this small population should be to supplement compensate for the food shortages the NNNR population during the dry season by establishing more food sources and or improving the vegetation quality of existing onethe food sources [96] to make sure all the individuals have a good body condition. In the mid-term, fFence dismantlement, as the mid-term goal, should be prioritized because it has partially blocked the movement of elephants and hindered their ability to find new food resources (Fig 1). Allowing the animals greater movement could provide more opportunities for the elephants to explore new suitable areas with enough food, eventually improving theelephant health condition regardless of the

season. —Ecological corridors should be considered as a long-term objective to connect this isolated elephant population to other suitable habitats. Since the western part of this reserve (Fig 1. Red part of NNNR) has some suitable habitats[53], an ecological corridor could be created by planting bamboo forest or bamboo-broadleaf forests, allowing individuals to move~~this population to migrate~~ between the eastern and western parts of the reserve. We also suggest conduct~~conduct~~ research on the evaluation of the ~~environmental capacity and~~ availability of food resources at different seasons. ~~Due to the very small population size and the scarcity of reproductive females, all suggestions should be urgently accomplished before the juveniles reach reproductive age to avoid potential inbreeding.~~ We emphasize the urgency of implementing these recommendations especially in ensuring a better body condition, which may improve the success rate of reproductivity in the long run.

Acknowledgments

This study was implemented by Beijing Forestry University and Beijing Eco-Bridge Continental. We thank the Second National Survey of Terrestrial Wildlife in China and National Forestry and Grassland Administration of China for their support of this study. Furthermore, the field work was strongly supported by the administrators of Nangunhe National Nature Reserve, for which we thank them. We also thank all the field staff, including Wang Jiahui, Liu Zhen, Liu Ying and all local rangers, especially Chen Deming, Li Yongxiang, Yang Shaobing, Wang Zhisheng, Li Chunlian and Li Zhimin, for their hard and invaluable work.

References

1. Campos-Arceiz A, Blake S. Megagardeners of the forest – the role of elephants in seed dispersal. Acta Oecologica. 2011;37(6):542-553. https://doi.org/10.1016/j.actao.2011.01.014.
2. McConkey KR, Nathalang A, Brockelman WY, Saralamba C, Santon J, Matmoon U, et al. Different megafauna vary in their seed dispersal effectiveness of the megafaunal fruit Platymitra macrocarpa (Annonaceae). Plos One. 2018;13(7):e0198960. https://doi.org/10.1371/journal.pone.0198960. PMID: 30020929.
3. Riddle HS, Schulte BA, Desai AA, Meer Lvd. Elephants - A conservation overview. Journal of Threatened Taxa. 2010;2(1):653-661.
4. Tan A, Yu L. Distribution and Protection of Asian elephant in China. Protection Forest Science and Technology. 2015;(5):89-91. https://doi.org/10.13601/j.issn.1005-5215.2015.05.035.
5. Platt SG, Bickford DP, Win MM, Rainwater TR. Water-filled Asian elephant tracks serve as breeding sites for anurans in Myanmar. Mammalia. 2019;83(3):287-289. https://doi.org/10.1515/mammalia-2017-0174.
6. Bowen-Jones E, Entwistle A. Identifying appropriate flagship species: the importance of culture and local contexts. Oryx. 2002;36(2):189-195. https://doi.org/10.1017/S0030605302000261.
7. Jiang Z, Jiang J, Wang Y, Zhang E, Zhang Y, Li L, et al. Red List of China's Vertebrates. Biodiversity Science. 2016;24(5):500-551. http://www.biodiversity-science.net/EN/10.17520/biods.2016076.
8. Choudhury A, Lahiri Choudhury DK, Desai A, Duckworth JW, Easa PS, Johnsingh AJT, et al. Elephas maximus. The IUCN Red List of Threatened Species. 2008:e.T7140A12828813. https://dx.doi.org/10.2305/IUCN.UK.2008.RLTS.T7140A12828813.en.

9. Li Z. Research on Asian elephan Conservation in China. Beijing: Science Press; 2018.

10. Yu Z, Kun J. Distribution, Population, Habitat Status and Population Management of Asian Elephant. World Forestry Research. 2018;31(2):25-30. https://doi.org/10.13348/j.cnki.sjlyyj.2018.0002.y.

11. Mingyong C. Asian elephant research in China: Science Press; 2006.

12. Sukumar R. A brief review of the status, distribution and biology of wild Asian elephants Elephas maximus. International Zoo Yearbook. 2006;40(1):1-8. https://doi.org/10.1111/j.1748-1090.2006.00001.x.

13. Zhang L, Dong L, Lin L, Feng LM, Yan F, Wang LX, et al. Asian Elephants in China: Estimating Population Size and Evaluating Habitat Suitability. Plos One. 2015;10(5):e0124834. https://doi.org/10.1371/journal.pone.0124834. PMID: 25992617.

14. Yuan T, Yongjun F, Chunlan Z, Baocheng Y, Xiaoping T, Huijian H. Effectiveness of line transects during wild animal surveys in mountain forest of South China. Biodiversity Science. 2015;23(1):109-115. http://www.biodiversity-science.net/EN/10.17520/biods.2014128.

15. Barnes RFW. The problem of precision and trend detection posed by small elephant populations in West Africa. African Journal of Ecology. 2002;40:179-185. https://doi.org/10.1046/j.1365-2028.2002.00376.x.

16. Mills LS, Citta JJ, Lair KP, Schwartz MK, Tallmon DA. Estimating Animal Abundance Using Noninvasive DNA Sampling: Promise and Pitfalls. Ecological Applications. 2000;10(1):283-294. https://doi.org/10.2307/2641002.

17. Zicheng Y, Ying C, Junsong L, Lifan W, You P, Zhiyong S, et al. Individual identification and population size assessment for Asian elephant based on camera-trapping techniques. Acta Theriologica

Sinica. 2018;38(1):18-27. https://doi.org/10.16829/j.slxb.150109

18. L.S.Eggert, J.A.Eggert, D.S.Woodruff. Estimating population sizes for elusive animals: the forest elephants of Kakum National Park, Ghana. Molecular Ecology. 2003;12(6):1389-1402. https://doi.org/10.1046/j.1365-294X.2003.01822.x. PMID: 12755869.

19. Liu X, Wu P, He X, Zhao X. Application and data mining of infra-red camera in the monitoring of species. Biodiversity Science. 2018;26(08):850-861. https://doi.org/10.17520/biods.2018053.

20. Xiao W, Hu L, Huang X, Xiao Z. Using capture-recapture models in wildlife camera-trapping monitoring and the study case. Biodiversity Science. 2019;27(3):257-265. https://doi.org/10.17520/biods.2018194

21. Zhang L, Cui S, Huang Y, Chen D, Qiao H, Li C, et al. Infrared camera traps in wildlife research and monitoring in China: issues and insights. Biodiversity Science. 2014;22(6):696-703. https://doi.org/10.3724/SP.J.1003.2014.14225.

22. Wearn OR, Glover-Kapfer P. Snap happy: camera traps are an effective sampling tool when compared with alternative methods. Royal Society Open Science. 2019;6(3):181748. https://doi.org/10.1098/rsos.181748. PMID: 31032031.

23. Jorge A A, Carlos E F S, Krisna G, Chris H, Johanna H, Emanuel M, et al. Community structure and diversity of tropical forest mammals: data from a global camera trap network. Philosophical Transactions of the Royal Society of London. 2011;366(1578):2703-2711. https://doi.org/10.1098/rstb.2011.0115. PMID: 21844049.

24. Chengcheng Z, Jun W, Alexander JS, Zhigang D, Liji W, Wantao D, et al. Biodiversity Assessment of Mammal and Bird Species from Camera Trap Data in Yanchiwan National Nature Reserve, Gansu Province, China. Journal of Resources and Ecology. 2018;9(5):566-574.

https://doi.org/10.5814/j.issn.1674-764x.2018.05.014.

25. Broto B, Mortelliti A. The status of research on the mammals of Sulawesi, Indonesia. Mammal Review. 2019;49(1):78-93. https://doi.org/10.1111/mam.12141.

26. Montalvo VH, Sáenz-Bolaño C, Luis D. Alfaro1 JCC, Guimarães-Rodrigues FH, Carrillo E, Sutherland C, et al. Seasonal use of waterholes and pathways by macrofauna in the dry forest of Costa Rica. Journal of Tropical Ecology. 2019;35(2):68-73. https://doi.org/10.1017/S0266467418000457.

27. Matsukawa A, Ahmad AH, Kohshima S. Social structure and reproduction of long-tailed porcupine (*Trichys fasciculata*). Mammalia. 2019;83(1):23-33. https://doi.org/10.1515/mammalia-2017-0104.

28. Sunarto S, Kelly MJ, Parakkasi K, Hutajulu MB. Cat coexistence in central Sumatra: ecological characteristics, spatial and temporal overlap, and implications for management. Journal of Zoology. 2015;296(2):104-115. https://doi.org/10.1111/jzo.12218.

29. Rich LN, Miller DAW, Muñoz DJ, Robinson HS, McNutt JW, Kelly MJ. Sampling design and analytical advances allow for simultaneous density estimation of seven sympatric carnivore species from camera trap data. Biological Conservation. 2019;233:12-20. https://doi.org/10.1016/j.biocon.2019.02.018.

30. Blanc L, Marboutin E, Gatti S, Gimenez O. Abundance of rare and elusive species: Empirical investigation of closed versus spatially explicit capture-recapture models with lynx as a case study. The Journal of Wildlife Management. 2013;77(2):372-378. https://doi.org/10.1002/jwmg.453.

31. Efford MG, Fewster RM. Estimating population size by spatially explicit capture-recapture. Oikos. 2013;122(6):918-928. https://doi.org/10.1111/j.1600-0706.2012.20440.x.

32. Bashir T, Bhattacharya T, Poudyal K, Sathyakumar S, Qureshi Q. Estimating leopard cat Prionailurus bengalensis densities using photographic captures and recaptures. Wildlife Biology.

2013;19(4):462-472. https://doi.org/10.2981/12-098.

33. Alexander JS. Advancing research and conservation of snow leopards in the Qilianshan mountains of China: management and methodological implications [Doctoral dissertation]: Beijing Forestry University; 2015.

34. O'Brien TG, Kinnaird MF, Wibisono HT. Crouching tigers, hidden prey: Sumatran tiger and prey populations in a tropical forest landscape. Animal Conservation. 2003;6(2):131-139. https://doi.org/10.1017/S1367943003003172

35. Bohm T, Hofer H. Population numbers, density and activity patterns of servals in savannah patches of Odzala-Kokoua National Park, Republic of Congo. African Journal of Ecology. 2018;56(4):841-849. https://doi.org/10.1111/aje.12520.

36. Park H, Lim A, Choi T-Y, Baek S-Y, Song E-G, Park YC. Where to spot: individual identification of leopard cats (*Prionailurus bengalensis* euptilurus) in South Korea. Journal of Ecology and Environment. 2019;43. https://doi.org/10.1186/s41610-019-0138-z.

37. Goswami VR, Madhusudan MD, Karanth KU. Application of photographic capture-recapture modelling to estimate demographic parameters for male Asian elephants. Animal Conservation. 2007;10(3):391-399. https://doi.org/10.1111/j.1469-1795.2007.00124.x.

38. Tye CA, Greene DU, Giuliano WM, Mccleery RA. Using camera-trap photographs to identify individual fox squirrels (*Sciurus niger*) in the Southeastern United States. Wildlife Society Bulletin. 2015;39(3):645-650. https://doi.org/10.1002/wsb.573.

39. Zheng X, Owen MA, Nie Y, Hu Y, Swaisgood RR, L.Yan, et al. Individual identification of wild giant pandas from camera trap photos - a systematic and hierarchical approach. Journal of Zoology. 2016;300(4):247-256. https://doi.org/10.1111/jzo.12377.

40. Zhiqiang Y, Li Z. Population and acticity characteristic of wild Asian elephant in Wild Elephant Valley. Acta Theriologica Sinica. 2006;26(4):359-367. https://doi.org/10.3969/j.issn.1000-1050.2006.04.007.

41. Chaiyarat R, Youngpoy N, Prempree P. Wild Asian elephant Elephas maximus population in Salakpra Wildlife Sanctuary, Thailand. Endangered Species Research. 2015;29(2):95-102. https://doi.org/10.3354/esr00706.

42. Gupta M, Joshi A, Vidya TNC. Effects of social organization, trap arrangement and density, sampling scale, and population density on bias in population size estimation using some common mark-recapture estimators. Plos One. 2017;12(3):e0173609. https://doi.org/10.1371/journal.pone.0173609. PMID: 28306735.

43. Hedges S, Johnson A, Ahlering M, Tyson M, Eggert LS. Accuracy, precision, and cost-effectiveness of conventional dung density and fecal DNA based survey methods to estimate Asian elephant (*Elephas maximus*) population size and structure. Biological Conservation. 2013;159:101-108. https://doi.org/10.1016/j.biocon.2012.12.010.

44. Wijeyamohan S, Treiber K, Schmitt D, Santiapillai C. A Visual System for Scoring Body Condition of Asian Elephants (*Elephas maximus*). Zoo Biology. 2015;34(1):53-59. https://doi.org/10.1002/zoo.21181. PMID: 25323789.

45. Fernando P, Janaka HK, Ekanayaka SKK, Nishantha HG, Pastorini J. A simple method for assessing elephant body condition. Gajah. 2009;31:29-31. https://doi.org/10.5167/uzh-32262.

46. Ramesh T, Sankar K, Qureshi Q, Kalle R. Assessment of wild Asiatic elephant (Elephas maximus indicus) body condition by simple scoring method in a tropical deciduous forest of Western Ghats, Southern India. Wildlife Biology in Practice. 2011;7(2):47-54. 10.2461/wbp.2011.7.17.

47. Morfeld KA, Lehnhardt J, Alligood C, Bolling J, Brown JL. Development of a body condition scoring index for female African elephants validated by ultrasound measurements of subcutaneous fat. Plos One. 2014;9(4):e93802. https://doi.org/10.1371/journal.pone.0093802. PMID: 24718304.

48. Fan Y, Li Z. Population genetic structure and population genetic diversity analysis based on mitochondrial DNA of Asian elephant Elephas maximus in China. Acta Theriologica Sinica. 2012;32(2):90-100. https://doi.org/10.16829/j.slxb.2012.02.002.

49. Barrett SCH, Kohn JR, Falk DA, Holsinger KE. Genetic and evolutionary consequences of small population size in plants: implications for conservation. Genetics & Conservation of Rare Plants. 1991.

50. Ruping W, Lin L. The extinction vortex of small population. Journal of Biology. 2008;25(6):14-16. https://doi.org/10.3969/j.issn.2095-1736.2008.06.004.

51. Limin F, Zhisheng W, Liu L, Shaobing Y, Bin Z, Chunhua L, et al. Habitat selection in dry season of Asian elephant (*Elephas maximus*) and conservation strategies in Nangunhe National Nature Reserve, Yunnan, China. Acta Theriologica Sinica. 2010;30(1):1-10. https://doi.org/10.16829/j.slxb.2010.01.001.

52. Reddy CS, Unnikrishnan A, Asra M, Manikandan TM, R.Jaishanker. Spatial Conservation Prioritisation of Threatened Forest Ecosystems in Myanmar. Journal of the Indian Society of Remote Sensing. 2019;47(10):1737-1749. https://doi.org/10.1007/s12524-019-01025-2.

53. Peng L, Hui W, Liu L, Jing L, Li Z. Habitat evaluation for Asian elephants (*Elephas maximus*) in Lincang: Conservation planning for an extremely small population of elephants in China. Biological Conservation. 2016;198:113-121. https://doi.org/10.1016/j.biocon.2016.04.005.

54. Yong-jing T, Zhi-sheng W, Gui-lian J, Xuan Z, Yuan H, Zheng-ling L, et al. Population dynamics analysis of Asian elephant in Nangunhe river basin. Forestry Construction. 2019;6:101-106.

55. Tang F, Du F, Sun G. Investigation report on background resources of Nangunhe National Nature

Reserve, Yunnan. China Forestry Press. 2015.

56. Guo B, Lan D. Status of wildlife resources in Nangunhe reserve. Chinese Journal of Wildlife. 1999;(4):46-47.

57. Bohnett E, Riordan P, Kun S. Initial Assessment on Large and Medium Sized Terrestrial Mammal Assemblage Using Camera Trapping in Nangunhe Nature Reserve in Yunnan, China. Journal of Resources and Ecology. 2015;6(5):331-344. https://doi.org/10.5814/j.issn.1674-764x.2015.05.007.

58. Varma S, Pittet A, Jamadagni HS. Experimenting usage of camera-traps-for population dynamics study of the Asian elephant *Elephas maximus* in southern India. Current Science. 2007;6(1):81-95. https://doi.org/10.12723/mjs.10.7.

59. Silva Sd, Ranjeewa A, Weerakoon D. Demography of Asian elephants (*Elephas maximus*) at Uda Walawe National Park, Sri Lanka based on identified individuals. Biological Conservation. 2011;144(5):1742-1752. https://doi.org/10.1016/j.biocon.2011.03.011.

60. Vidya TNC, Prasad D, Ghosh A. Individual Identification in Asian Elephants. Gajah. 2014;40:3-17.

61. Sukumar R, Joshi nv, Krishnamurthy V. Growth in the Asian elephant. Proceedings: Animal Sciences. 1988;97(6):561-571. https://doi.org/10.1007/BF03179558.

62. Goswami VR, Lauretta MV, Madhusudan MD, Karanth KU. Optimizing individual identification and survey effort for photographic capture-recapture sampling of species with temporally variable morphological traits. Animal Conservation. 2012;15(2):174-183. https://doi.org/10.1111/j.1469-1795.2011.00501.x.

63. Borchers DL, Efford MG. Spatially explicit maximum likelihood methods for capture-recapture studies. Biometrics. 2008;64(2):377-385. 10.1111/j.1541-0420.2007.00927.x. PMID: WOS:000255959500007.

64. Borchers DL, Buckland ST, Zucchini W. Estimating Animal Abundance: Closed Populations. Springer，London. 2002. https://doi.org/10.1007/978-1-4471-3708-5.

65. López-Bao JV, Godinho R, Pacheco C, Lema FJ, García E, Llaneza L, et al. Toward reliable population estimates of wolves by combining spatial capture-recapture models and non-invasive DNA monitoring. Scientific Reports. 2018;8(1):2177. https://doi.org/10.1038/s41598-018-20675-9 PMID: 29391588.

66. Gardner B, Reppucci J, Lucherini M, Royle JA. Spatially explicit inference for open populations: estimating demographic parameters from camera‐trap studies. Ecology. 2010;91(11):3376-3383. https://doi.org/10.1890/09-0804.1. PMID: 21141198.

67. Royle JA, Chandler RB, Sollman R, Gardner B. Spatial Capture-Recapture: Academic Press; 2014.

68. Royle JA, Gardner B. Hierarchical spatial capture-recapture models for estimating density from trapping arrays. In: O'Connell A.F., Nichols J.D., Karanth K.U. (eds) Camera Traps in Animal Ecology. : Springer, Tokyo; 2011.

69. Efford MG. Estimation of population density by spatially explicit capture-recapture analysis of data from area searches. Ecology. 2011;92(12):2202-2207. https://doi.org/10.1890/11-0332.1. PMID: 22352159.

70. Silveira L, Anah TAJ, Diniz-Filho JAF. Camera trap, line transect census and track surveys: a comparative evaluation. Biological Conservation. 2003;114(3):351-355. https://doi.org/10.1016/S0006-3207(03)00063-6

71. Li Z, Kang A, Lang J, Xue Y, Ren Y, Zhu Z, et al. On the assessment of big cats and their prey populations based on camera trap data. Biodiversity Science. 2014;22(6):725-732. https://doi.org/10.3724/SP.J.1003.2014.14079

72. Ling Q. Habitat Selection by Asian Elephant (*Elephas maximus*) in Nangunhe Nature Reserve, Yunnan, China: Northwest University; 2007.

73. Yuming Y, Genmin W, Zhisheng Z, Zhongli W. Wild elephant population in Nangunhe reserve. Forest Inventory & Planning. 1982;02.

74. Jiasheng T, Jianhao W, Xiaolin H. Summary of Nangunhe nature reserve. Forest Inventory & Planning. 1986;01.

75. Yuming Y, Fan D. Nangunhe National Nature Reserve in China. Yunnan Science Publication. 2004.

76. Vidya TNC, Sukumar R. Social and reproductive behaviour in elephants. Current Science. 2005;89.

77. Guo B. Investigation and protection advice of wild elephant in Nangunhe reserve. YUnnan Forestry. 1989.

78. Royle JA, Nichols JD, Karanth KU, Gopalaswamy AM. A hierarchical model for estimating density in camera-trap studies. Journal of Applied Ecology. 2010;46(1):118-127. https://doi.org/10.1111/j.1365-2664.2008.01578.x.

79. Alfred R, Ahmad A, Payne J, Williams C, Ambu L, Phua M-H, et al. Home Range and Ranging Behaviour of Bornean Elephant (*Elephas maximus* borneensis) Females. Plos One. 2012;7(2):e31400. https://doi.org/10.1371/journal.pone.0031400. PMID: 22347469.

80. Leggett KEA. Home range and seasonal movement of elephants in the Kunene Region, northwestern Namibia. African Zoology. 2006;41(1):17-36. https://doi.org/10.3377/1562-7020(2006)41[17:Hrasmo]2.0.Co;2.

81. Kumara H, Rathnakumar S, Mavatur A, Singh M. Estimating Asian elephant, Elephas maximus, density through distance sampling in the tropical forests of Biligiri Rangaswamy Temple Tiger Reserve, India. Tropical Conservation Science. 2012;5(52):163-172.

https://doi.org/10.1177/194008291200500206.

82. Lichao M. Conservation genetic path to understanding the structure of Asian elephant (*Elephas maximus*) group: Beijing Normal University; 2004.

83. Lisa R, Jay H. Some Observations Regarding Allomaternal Caretaking among Captive Asian Elephants (*Elephas maximus*). Journal of Mammalogy. 1987;68(2):438-442. https://doi.org/10.2307/1381494.

84. Lahdenperä M, Mar KU, Lummaa V. Nearby grandmother enhances calf survival and reproduction in Asian elephants. Scientific Reports. 2016;6:27213. https://doi.org/10.1038/srep27213. PMID: 27282468.

85. Kurt F, Hartl G, Tiedemann R. Tuskless bulls in Asian elephant Elephas maximus . History and population genetics of a man-made phenomenon. Acta Theriologica. 1995;40:125-143. https://doi.org/10.4098/AT.arch.95-51.

86. Paulraj S, Subramanian C. Skewed sex ratio in elephant population - How it may affect the demography and population growth? A case study in Mudumalai Wildlife Sanctuary and National Park, Tamil Nadu, Southern India. Indian Forester. 2000;126:1046-1053.

87. Archie E, Chiyo P. Elephant behaviour and conservation: Social relationships, the effects of poaching, and genetic tools for management. Molecular Ecology. 2011;21:765-778. 10.1111/j.1365-294X.2011.05237.x.

88. Gubbi S, Swaminath M, Poornesha H, Bhat R, Raghunath R. An elephantine challenge: Human-elephant conflict distribution in the largest Asian elephant population, southern India. Biodiversity and Conservation. 2014;23:633-647. https://doi.org/10.1007/s10531-014-0621-x.

89. Silva Sd, Webber CE, Weerathunga US, Pushpakumara TV, Weerakoon DK, Wittemyer G.

Demographic Variables for Wild Asian Elephants Using Longitudinal Observations. Plos One. 2013;8(12):e82788. https://doi.org/10.1371/journal.pone.0082788. PMID: 24376581.

90. Fernando P, Wikramanayake ED, Janaka HK, Jayasinghe LKA, Gunawardena M, Kotagama SW, et al. Ranging behavior of the Asian elephant in Sri Lanka. Mammalian Biology. 2008;73(1):2-13. https://doi.org/10.1016/j.mambio.2007.07.007.

91. Srinivasaiah N, Kumar V, Vaidyanathan S, Sukumar R, Sinha A. All-Male Groups in Asian Elephants: A Novel, Adaptive Social Strategy in Increasingly Anthropogenic Landscapes of Southern India. Scientific Reports. 2019;9:8678. https://doi.org/10.1038/s41598-019-45130-1. PMID: 31273235.

92. Pokharel SS, Seshagiri PB, Sukumar R. Assessment of season-dependent body condition scores in relation to faecal glucocorticoid metabolites in free-ranging Asian elephants. Conservation Physiology. 2017;5(1):cox039. https://doi.org/10.1093/conphys/cox039. PMID: 28721215.

93. Guozheng S, Guangyuan Z, Zicheng Y, Mengjun W. Research progress and direction of human elephant conflict mitigation projects. Forestry Construction. 2019;6:5-9.

94. Sheng-dong Y, Yun D, Ai-dong L, Mang-cai W, Bin W, De Z, et al. A preliminary study of wireless infrared camera technology in alleviating the human-elephant conflict. Forestry Construction. 2019;(6):61-66.

95. Zhi-hong W, Heng-zhen Z, Wen-lan Z, Xuan Z, Wen-bian Y, Zheng-ling L, et al. An analysis of population dynamic on the Xishuangbanna Menghai-Pu'er Lancang Asian elephant population. Forestry Construction. 2019;(6):79-84.

96. Xian-ming G, Lan-xin W. Thoughts on the construction of Asian elephant food source base in Xishuangbanna. Forestry Construction. 2019;(6):30-33.

Supporting information

S1 ~~Table~~Dataset. A simplified database of Asian elephant in NNNR.

~~S2~~ S1 Table. Historical elephant population in NNNR estimated by different methods.

Dear reviewers and editor,

First, we would like to thank the reviewers and editors for your time, comments and expertise. Your exhaustive and positive comments and suggestions again make our paper more rigorous and logistic. We were fully agreed that you made small changes in the main text and reference in manuscript, all the changes were accepted. The following letter gives our response to the reviewer's specific comments are requested in this Major Revision.

General Comments of Reviewer #1:

Response: Thanks for your recognition of our new version manuscript. Here were responses of your two small suggestions.

Comment 1: Line 323, 'as in' should be 'than in'

Response: The word has been replaced by the one your recommendation. Please go to P16, L313.

Comment 2: line 401-402 - You may want to include a short explanation for why larger home ranges and tolerance of disturbance could be linked to higher BCS. Additionally, the sentence starting 'Which means' should instead start 'Tolerance means'

Response: Large home ranges and tolerance of disturbance means the male elephants obtain more food resources, which contribute a good body condition in turn. The new sentence has been changed like "This may be because adult males are known to have larger home ranges than adult females, and are more tolerant to human disturbance: that means they can explore more frequently areas near the reserve boundary to obtain more food resources, where intensive anthropogenic activities like hunting, grazing, fishing and collecting have also been recorded by cameras."

General Comments of Reviewer #2:

Response: Thank you for your exhaustive and positive comments and suggestions. We made huge changes on Discussion to make it clearer and logistical. In Conservation Recommendations part, we only used two sentences to describe the importance of

long-term monitoring of infrared cameras. Then focus on the suggestions that improving food resources to enhance the elephant body condition. This paper has been checked by professional editors, both native speakers of English.

Comment 1: On line 93-95, This sentence should go Line 80, to finish the paragraph on photo ID.

Response: This sentence had moved to Line 80 in the new version of manuscript. Please go to P4, L80-82.

Comment 2: On the line 99-115, These two paragraphs should go right after the first one, when you describe elephant populations in China. Otherwise, these paragraphs do not connect well with the previous ones.

Response: We deleted some sentences in these two paragraphs and have rewritten remaining sentences. We thought these could be more logical and precise than before. Please go to P5, L95-105.

Comment 3：On the line 200, Maybe the way you present the table is not optimal, because when you describe the categories in SEX, you include already both sex and age. So maybe you should start describing AGE, and then SEX.

Response: We adapted your opinion by describing AGE first and then describing SEX which was simply categorized to female and male. Please go to P9-10, see Table 1.

Comment 4: On Table 1: Ok, so it is obvious that adult and subadult males that are tuskless may be impossible to distinguish; maybe add "and no penis", to distinguish them from their male counterparts; Same, you should add the testicles/penis part, but here, how do you distinguish juvenile from subadult males

Response: We have simplified the category of SEX by just describing female as “Without obvious tusks but with prominent breasts, belly swelling or the presence of nursing offspring” and male as “With visible testicles or penis, and/or obvious tusks”. See Table 1.

Comment 5: On the line 210, It should be replaced by "each", even when it is clear that you mean "each and every"

Response: It should be “each” in this situation. Please go to P11, L222.

Comment 6: On the line 232, Not too clear to me. You mean to say "i.e."?

Response: This sentence has been changed into “The file named “capture histories” contained data on individual identified each sampling occasion from the specific detector (camera).” Please go to P11, L221-222.

Comment 7: On the line 323-327, This sentence is unclear. What do you want to convey here? Do you want to say that different methods lead to different results inveitably, or do you just want to point out that historically the size of the population was estimated at about 20 individuals?I think it is the second case, but in any case by straightforward.

Response: This sentence has been changed to make it simpler. “Previous studies have estimated this population to be around 20 individuals. Although population estimation using different methods could generate bias in direct comparison, research has shown that this elephant population has remained small during the last few decades.” Please go to P16-17, L313-317.

Comment 8: On the line 327-330, The logics here remains unclear; with the information provided it is not obvious why being in the same part of the park means that there should be more elephants. Maybe habitat quality is not that good anymore (in fact, the fact that females were not in good body condition might indicate something like that)... or it might be that the highest ever recorded population in the park was an artefact due to loss of habitat in the surrounding (and then a packing effect).

Response: We did not do any analysis in habitat quality estimation, so it is surprising to readers see the point suddenly. This sentence has been deleted.

Comment 9: On the line 335, You have four elephants dead since 2003... don't calculate statistics over that! Just say that 3 of the 4 elephants found dead since 2003 have been immature individuals.

Response: This sentence has been changed to "Additionally, 3 of 4 elephants found dead since 2003 have been immature individuals (Table5)." Please go to P17, L320-321.

Comment 10: On the line 335-336, This is part of the life history of the species, not somethinf particular to this population. The way it is written, it is misleading.

Response: This sentence has been rewritten to "Moreover, the fact that elephants have a long adolescence and long inter-birth intervals means it takes time for NGH elephants to recover and they are more vulnerable to local population extinction, especially if young individuals are being poached or dying from other unnatural causes. All these facts result in the current small population size that could lead to the extinction of this local population." Please go to P17, L321-326.

Comment 11: On the line 362, Yes, which makes your suggestion that the population could be bigger even more bizarre.

Response: This sentence has been changed to "The relatively high population density might lead to more intense competition among individuals for limited natural resources, and even greater human-elephant conflict as elephants resort to crop-raiding for food, which could be further studied in the future." Please go to P19, L350-353.

Comment 12: On the line 368-369, Why in the near future? Isn't it happening already?

Response: This sentence has been changed to "Previous study already confirmed these individuals were related to each other [82]. This phenomenon of inbreeding will be more serious in the near future due to the small population size." Please go to P19, L357-359.

Comment 13: On the line 387, Why are you talking about unbalanced sex ratio here. it comes from nowhere. I think it should be a new paragraph, and for your reader to follow you, remind them of the sex ratio you found (assuming these individuals were indeed males or not).

Response: We agreed your comments on this paragraph which makes it more logical and coherence. So, we started a new paragraph to talk about sex ratio and have rewritten some sentences. Please go to P20, L378-387.

Comment 14: On the line 390-394, This sentence makes no sense. There are two informations here, but how they are related is a mystery.

Response: This sentence has been deleted.

Comment 15: On the line 399, Ok, it was observed in another population, but what about another one? You cannot cherrypick information as if were supporting yours. Is there a pattern among small population in crowded places? or in areas with low habitat quality? or anything that could explain why females exhibit this low body condition?

Response: The reference we cited before had similar situation on population density, but we had smaller study area. So, this sentence has been deleted.

Comment 16: On the line 401, The comparison is male vs female in adult individuals: leave the immature out.

Response: The "immature males" has been deleted in this sentence. Please go to P21, L392.

Comment 17: On the line 403, You surely mean collecting, which you already use.

Response: Actually, it should be grazing, we used the wrong word. Please go to P21, L394.

Comment 18: On the line 419, Use a proper way to cite this. If these are unpublished documents, cite the staff who provided the information as personal communication. Also, a documentary is not documentation.

Response: This citation way has been changed to "Li Zhimin, personal communication". Please go to P21, L411.

Comment 19: On the line 437, Sorry, but how documenting this population will ensure its survival? It seems rather a leap of faith!

Response: Sorry the way we wrote the sentence confused you. This sentence has been changed to "The long-term placement of cameras in NNNR is an efficient mean of monitoring to obtain valuable data on demography, fecundity and body condition variation across seasons and years." Please go to P22, L429-431.

Comment 20: On the line 442, Where was it confirmed first? I don't remember reading anything on that earlier in the manuscript

Response: Sorry for that we used the wrong word. This sentence has been changed to "The results of this study match those observed in earlier studies that providing more suitable habitat is urgently needed to ensure an adequate supply of food for this isolated population during the dry season." Please go to P22, L433-435.

Comment 21: On the line 447, of what?

Response: This sentence has been changed to "The short-term objective to increase the survival quality of this small population should be to compensate for the food shortages the NNNR population during the dry season by establishing more food sources and improving the quality of existing one to make sure all the individuals have a good body condition." Please go to P23, L438-441.

Comment 22: On the line 448, by establishing what?

Response: Please see the response of comment 21.

Comment 23: On the line 459-460, Do you mean carrying capacity? I don't think it makes sense, unless you start with that and use it to demosntrate that the population, under the current conditions, is not viable.

Response: We agreed your opinion after deeply considering, so, we deleted the "environmental capacity".

Comment 24: On the line 461-463, That recommendation makes no sense. First, there is probably inbreeding since quite long: you do not provide evidence that the connection with another population has been lost recently. Second, in the current state of affairs, it seems that ensuring a better body condition is more important than ensuring reproduction.

Response: We agreed that you mentioned this population facing the health problems. So, this sentence has been changed to "We emphasize the urgency of implementing these

recommendations especially in ensuring a better body condition, which may improve the success rate of reproductivity in the long run.” Please go to P23, L452-454.
