# Supplementary material for: Assessing population structure and body condition to inform conservation strategies for a small isolated Asian elephant (Elephas maximus) population in southwest China
Source: PLoS One. 2021 Mar 9;16(3):e0248210. doi: 10.1371/journal.pone.0248210 (PMC7942997; doi:10.1371/journal.pone.0248210)
Supplement: Attachment — Submitted filename: Response to Reviewers.docx [file pone.0248210.s004.docx]

·Dear reviewers and editor,

First, we would like to thank the reviewers and the editors for your time, comments and expertise. Your exhaustive and positive comments and suggestions makes our paper more rigorous and logistic. The following letter gives our responses to the reviewer’s comments as requested in your letter 22 June 2020.

**General Comments of Reviewer #1:**

**Response:** Thank you for your comments and suggestions. Methods has been re-written as to make the Body Condition Scoring (BCS) section clearer and more comprehensible. This paper has been checked by professional editors, both native speakers of English.

**Specific Comments of Reviewer #1:**

**Comment 1:** **On line 220, the buffer zone is said to be 5km around the outermost camera sites. There was no citation here, so what was the justification for using 5km and not a different value?**

Response: Considering the small size of the elephant distribution, with the east and west boundary being no wider than 10 km, we therefore defined our study area as extending 5 km beyond all camera stations, corresponding to an area of 255.8 km^2^. We cited one reference. Please go to P12, L226-228.

**Comment 2: Lines 245-246 Please list all the model covariates and provide some description e.g. was age a categorical factor in the models? Was BCS considered as a continuous variable for the purposes of getting the correlation coefficients?**

Response: The covariates include sex, age and whether the elephant was solitary or in the herd. BCS was not a continuous variable. Please go to P13, L260-262.

**Comment 3: It is unclear how many photographs were used to calculate the scores for each elephant. If multiple, were they all from around the same time and consistent? If the score is an average or not needs to be stated.**

Response: Five of the clearest images for each physical characteristic were selected to assess the body condition. Thirty images were used to calculate the comprehensive scores for each individual. Please go to P13, L244-246.

**Comment 4: Lines 285-287 Asian elephants may allolactate (Rapaport, L. & Haight, J. 1987 Some observations regarding allomaternal caretaking among captive Asian elephants (Elephas maximus). J. Mammal. 68, 438–442), and there may be other forms of allomothering too (Lahdenperä M., Mar, K.U., Lummaa V. 2016 Nearby grandmother enhances calf survival and reproduction in Asian elephants. Scientific Reports 6: 27213; Lynch E. C., Lummaa, V., Htut W., Lahdenperä M. 2019 Evolutionary significance of maternal kinship in a long-lived mammal. Phil. Trans. R. Soc B. 374: 20180067) – I’m not sure that any relationship can be considered ‘definite’.**

Response: Yes, we lost sight of that. Only the DNA methods can tell us the true relationship among individuals in this population. The word “definitely” has been deleted. We discussed this situation in the Discussion section. P16, L297-301; P20, L370-379.

**General Comments of Reviewer #2:**

**Response:** Thank you for your exhaustive and positive comments and suggestions. Some missing information of Methods has been re-written as to make this section clearer and more comprehensible. We apologize for the previous manuscript as it was very unclear in several sections and there was overspeculation in the Discussion and Conservation Recommendations sections. A major revision of this manuscript has been accomplished and many unnecessary parts have been deleted. In the Result and Discussion sections, we combined the population size and density part to make the paper more coherent and logical. And in the Conservation Recommendations section, we totally replaced the previous version by a simpler and more summarized one. This paper has been checked by professional editors, both native speakers of English.

**Specific Comments of Reviewer #2:**

**Comment 1:** **L. 34: Insert BCS here for the first time.**

Response: We have inserted BCS. This sentence has been changed to “The health condition of this elephant population was assessed by using an 11-points scale of Body Condition Scoring (BCS) method which showed that this population had a medium body condition with an average of 5.75 (n=12, range 2-9).” Please go to P2, L33-35.

**Comment 2:** **The fact that individuals had such low BCS should be highlighted in the abstract (if space allows).**

Response: We have rewritten this sentence. Please see the response to comment 1 or P2, L33-35.

**Comment 3:** **L. 44: End the sentence at “dispersal” and start a new sentence about elephants as umbrella species.**

Response: This sentence has been changed as you recommended. Please go to P3, L42-46.

**Comment 4:** **L.49: When you mention that the species is listed as Endangered in IUCN Red List, and then that its population is decreasing, you make a tautological argument. Any endangered population is decreasing. You should rather change the sentence and say that however important it is for conservation (as an umbrella species and an ecosystem engineer), there is a problem with the species, which population is decreasing, hence justifying its endangered status.**

Response: Yes, we believe that this is more logical. This sentence has been changed to make it simpler. “It is a Class I Key Protected Species in China and listed as an Endangered under the IUCN Red List.”. Please go to P3, L48-49.

**Comment 5:** **L.77: Puma is not a good example, as they do not have spots or stripes. I would also keep this list more general, citing genera that have spots or stripes, such as tigers, leopards, giraffes, etc.**

Response: Puma has been replaced by leopard cats since they have stripes on their bodies. Please go to P4, L76.

**Comment 6:** **L.88: There is a jump between the two sentences (no logical sequence). Maybe you could close the previous argument by saying that for NNNR there are not such data available. Then you could start a new paragraph on this isolated population, its genetic status, etc.**

Response: A new paragraph has been created. Please go to P5, L99.

**Comment 7:** **L.99-108: This new paragraph also makes a jump. You should place it in the series of paragraphs describing how trap cameras (and photographs more broadly) are another way that tool can be useful in conservation and management.**

Response: We moved the BCS paragraph forward placing it behind the paragraph that introduces individual identification by camera traps. Please go to P4-5, L81-92.

**Comment 8:** **L.104-105: It is unclear why BCS is most meaningful “especially [in] small isolated populations”. Explain it or delete it.**

Response: We deleted the word “small”. Now the sentence looks like “While BCS is individual-based, it is most meaningful when applied to a population, as an early indicator of the impact of management actions on the average health condition of a population.”. Please go to P5, L87-90.

**Comment 9:** **L. 107-108. Delete this sentence. It is of very little relevance for a journal with a broad, worldwide audience.**

Response: This sentence has been deleted. Please go to P5, L92.

**Comment 10:** **L.109-116: There is one more reason to move the previous paragraph upward: this paragraph is connected to the one preceding the previous one (i.e., after the paragraph l. 82-98).**

Response: That is correct. After moving the previous paragraph upward, this paragraph is now well connected to the previous and following paragraphs. Please go to P6, L109-115.

**Comment 11:** **L. 114-115: “for the development of a successful project focusing on the introduction of individual elephants from other populations”. That is the kind of sentence that does not serve you well. You talk about introducing individuals, but you have provided no evidence that the conditions to ensure the survival of introduced animals are met in the area. In many areas, habitat (quantity or quality) or negative relationships with humans do not ensure survival, making the prospect of reintroduction a loosing strategy.**

Response: We did not obtain enough evidence to support the introduction of individuals as a successful plan for the survival of this small population. Thus, we deleted any mention about the introduction of elephants throughout the manuscript. Please go to P6, L112-115 and Conservation Recommendation section.

**Comment 12:** **L. 120: I would change “to analyze the factors” to reflect that it is indeed a discussion of some factors that might have affected the population that you will present.**

Response: This sentence has been changed to “We sought to understand which factors might have affected this populations size in recent years in order to develop targeted and effective protection strategies.”. Please go to P6, L118-119.

**Comment 13:** **L. 122: Why in China only and not in other parts of the species distribution?**

Response: We have deleted “in China”. The new sentence is “Furthermore our approaches can be applied to other elephant populations with further implications for global Asian elephant conservation.” Please go to P6, 120-121.

**Comment 14:** **L. 134: Give properly the geographical coordinates (including N to the latitude, and W to the longitude).**

Response: The sentence had been changed to “It occupies 70.82 km^2^ at latitude 23°13'- 23°19'N, longitude 98°54'- 99°05'E, and the altitude ranges from 510 m to 1747 m.”. P7, L131-133.

**Comment 15:** **L. 137:** **Please give a proper annual precipitation and temperature (a single value), or explain the range you provide.**

Response: Mean annual precipitation is 351.3 mm in the dry season and 1983.3 mm in the rainy season, while temperature varies at different altitudes. The average monthly temperature range is hottest in July, varying between 12.3-25.5°C and it is coldest in January with values between 2.8-14.3 ℃. (Reference: NGH scientific report. No single value for annual temperature, just range value.) Please go to P7, L134-137.

**Comment 16:** **L. 138: Why natural in “natural tropical forest”?**

Response: It should be a tropical forest. We made a little mistake when we read the corresponding reference. Please go to P7, L138.

**Comment 17:** **L.140: By evergreen vegetation do you mean evergreen broadleaf species, in opposition to coniferous species?**

Response: Yes, it is evergreen broadleaf species. We have added “broadleaf” in the manuscript. Please go to P7, L141.

**Comment 18:** **L. 148: Use a better title, such as survey design or camera trap set-up.**

Response: The title has been changed to “Camera trap set-up”. Please go to P7, L148.

**Comment 19:** **L. 151: “along the animal trails with forest gaps where there were signs of elephants”. Unclear. Did you set up the cameras in forest gaps that were crossed by elephant trails? If so replace “with” by “in”. In any case, review this.**

Response: Actually, we set up the cameras in trails and forest gaps, but the gaps were often located in trails. So, here we replace “with” by “and” to make it clearer. Please go to P8, L155-157.

**Comment 20:** **L. 152: “as well as other activities”. You cannot remain vague here; mention them all.**

Response: The elephant signs included lying sites, foraging sites, footprints, dungs and urine marks. Please go to P8, L155-157.

**Comment 21:** **L. 152: Explain why you avoided certain areas. Was it because elephants do not use them, or because you had concerns for the safety of people in the field?**

Response: We avoided the northwestern part of the reserve as the topography is tough, with steep cliffs of up to 1747 meters, and historically, there have not been any records of elephants in this area. Please go to P8, L159-162.

**Comment 22:** **L. 149-164: In this section, there is information missing. As far as I understand you had one camera per site, but one sentence mentions that "at each site, camera traps were operated...", which suggests there were several cameras at each site. Also, there is information missing regarding the number of sites or cameras, the minimum distance that could separate two sites (because there could be spatial autocorrelation in your data). Finally, you should mention the camera model(s) you used in your study. Elephants are not small nor super fast, so trigger time should not be an issue, but still, getting information on the material used is necessary for replication purposes.**

Response: A total of 52 cameras at 52 stations or sites (P8, L150-151). We set one camera trap at each camera site, but the number of camera sites varied in each grid. More than one camera was installed in the grids that contained lots of elephant signs (P8, L170-171), and the minimum distance between two cameras was 200 meters (P8, L158-159). The camera model was Ltl Acorn 6210 (P8, L150) and we programmed the camera to take sequential images consisting of three photographs plus one 12 second video (P8, L168-170).

**Comment 23:** **L. 166-169: The whole sentence needs to be rewritten for better clarity. After reading it, I am left with doubt about what constituted an independent event. I think I understood that an independent event could include several photographs of various individuals, with at least 5 h between such photographic sequences to make it temporally independent, but it is far from clear.**

Response: We have provided a more detailed explanation about independent events than in the previous manuscript version. Please see P9, L175-181.

**Comment 24:** **L. 177: Did you determine the sex in juveniles and calves? If so, how did you do that?**

Response: Yes, we made it clearer in Table 1. Please see P10, L199.

**Comment 25:** **L. 185-187: How do you know they did not change? Ear can be teared at any moment; a tusk could be broken digging up... maybe it is the way it is written that makes it not very convincing. If you have an elephant with a particular tear in the ear, it is easy to "recapture" it, but if there was no marking, I am not sure how this could be reliable. I think a better explanation would help.**

Response: Yes, that is a better explanation that would be more convincing. Morphological characters including facial bones, ear, tusk, back and tail hair, old scars and injuries, as well as physical characteristics related to sex and age classes, confirmed the reliability of identification results. Please see P10, L196-198.

**Comment 26:** **Table 1: 1) Regarding sexing, as mentioned earlier I think male and female should be labeled properly adult female and adult male, as the diagnostic characteristics are found in adult (and possibly subadult) individuals only. 2) “Sunken prominence”. How can a prominence be sunken? 3) Your classes for tusk length are ok, but I am not ok with the fact that you do not explain how you classified them in each of these categories. I am sure you had a criteria to decide (such as tusk length relative to the trunk).**

Response: 1): We had rearranged sex categories as adult female, adult male, subadult male, juvenile female, juvenile male and calf male. 2): It was a description mistake. The sentence has been changed to “Extent of depression”. 3): Yes, we have a criterion that uses trunk as a reference to classify the tusk length. For more details please go to Table 1 on P10, L199.

**Comment 27:** **L. 197: “The movement pattern of individuals activity center in study area”. This is unclear. Do you mean “The movement pattern of individual activity centers in the study area”, i.e., the movement pattern of the activity centers of a given individual evaluated at different times (sessions)? Sorry, but the devil is in the details!**

Response: Yes, the model can evaluate the movement pattern according to the capture history. We missed the time limitation. The sentence has been changed to “The state model describes statistically the mechanisms determining the distribution of the animals in the study area, while the observation model quantifies the probabilities of detection or capture, given the searched region, their locations and characteristics at different sessions”. Please go to P11, L205-209.

**Comment 28:** **L. 198-200: "the probability of detecting an individual at a particular detector (…) to the distance of the detector" makes no sense. Please rewrite.**

Response: The sentence has been rewritten combining it with the forward sentence. Please see the response of comment 27 or onP11, L205-209.

**Comment 29:** **L. 206 and elsewhere in this section: Use trap or detector, or explain that detectors are traps, but remain consistent throughout your explanations.**

Response: All the words corresponding to “detectors” were replaced by “traps”. Please go to P11, L212; L214; L217.

**Comment 30:** **L. 217: Again, there is a problem with the phrasing and it is unclear what you mean.**

Response: This sentence has been deleted. Please go to L12, L224.

**Comment 31:** **L. 219-221: The whole sentence needs to be rephrased so that the reader can understand exactly what you did. Also “from the outermost x and y coordinates of the camera sites” is very unclear. A camera site is not a discrete point in space???**

Response: The sentence has been changed to “Considering the small size of the elephant distribution, with the east and west boundary being no wider than 10 km, we therefore defined our study area as extending 5 km beyond all camera stations, corresponding to an area of 255.8 km^2^.”. Please go to P12, L226-228.

**Comment 32:** **L. 224: Again, this is unclear. Is the file named "Capture histories"? Please check the sentence.**

Response: Yes, the file is named “capture histories”. The sentence has been changed. Please go to P12, L231.

**Comment 33:** **L. 225: Is it "occasion" (you haven't defined it previously) or "event"? Also, earlier you defined individual events as being separated by at least 5 hours. This means that several individual events could occur on a same day. How can your data base account for that if your time unit is "day"?**

Response: “Occasion” was defined as each event, as well as the “sequence”. The cameras captured several events, accounting for different occasions. Please go to P12, L232.

**Comment 34:** **L. 227: “whole data as five different sessions”. Again, it is unclear. Do you mean you divided your 4-month sampling period in 5 (i.e. each session would last 24 days)?**

Response: Five different sessions, corresponding to each of the months and one corresponding to the whole survey period, were used to run the model to see the estimation accuracy under different detection efforts. P12, L233-236.

**Comment 35:** **L. 236-244: The first of these five sentences is very unclear. Further, if you followed the method developed by 49 or 50, just cite them, and mention that the part on which the scoring focuses are the ribs, shoulders, etc. and say why. Usually it is because these parts can accumulate fat.**

Response: We added more information to describe the reasons we choose ribs, shoulders and other body parts as scoring criterion. Please go to P13, L246-252.

**Comment 36:** **L. 244: Before mentioning which software you used to perform your analyses, say what kind of analysis you performed.**

Response: This sentence has been deleted. Please go to P13, L260.

**Comment 37:** **L. 244-245: Which type of correlation coefficient?**

Response: It is Pearson Correlation Coefficient. P13, L260.

**Comment 38:** **L. 245: “ratios of various covariates”: why ratios? Age is not a ratio, nor is sex or the solitary vs group status.**

Response: The word “ratio” has been deleted. Please go to P13, L260-262.

**Comment 39:** **L. 246: “whether an individual was present or not in the herd”. Here you lost me... do you mean you checked the correlation between BCS and whether an individual was solitary or in herd? (note that this is not what you have written; you have written “the herd”)**

Response: Yes, we wanted to divide individual elephants into those that were solitary and those that were part of a herd to see if this factor could affect the BCS. Please go to P13, L260-262.

**Comment 40:** **Table 2: 1) Revise the title of this table. 2) Characters is incorrect here and in several places in the manuscript. The correct word here is characteristics.**

Response: 1): The title has been changed to “Table 2. Characteristics used to assess the body condition in Asian elephant, NNNR”. 2): All the words “characters” have been changed to “characteristics”. Please go to P13, L263 to see Table 2.

**Comment 41:** **L. 250-253: This is much better suited in Methods. I kept wondering how you set up your cameras, how many, and here is the answer. Yours results should start with what you obtained, not what you did.**

Response: Yes, these sentences presented in the Methods section were more logical. These sentences have been moved to their current location. P7-8, L149-152.

**Comment 42:** **L. 254: You never spoke of video before! Fix that in Methods or delete here if they were not used.**

Response: We apologize for this mistake. We programmed the camera to take three pictures and a 12 second video. We replaced the words “picture” and “video” by “image” in the Methods section. Please go to P8, L168-170.

**Comment 43:** **L. 261-264: You should revise that sentence. As it is, there is not much difference between the individual that was seen the least and the others. Rather give the mean and the range.**

Response: We strongly agree with your suggestions. Currently, there is a brief comment about the capture histories of individual and herds. Please go to P14, L271-274.

**Comment 44:** **Fig. 2: This title does not correspond to what you describe in the text (i.e., the 12 identified individuals). I think it should say something like "Asian elephants individually identified in NNNR park, China”. Also, note that the way you cited the figure, one would expect to see the 12 individuals identified. Here you show only half of them.**

Response: We strongly agree with your suggestions and the title of “Fig 2” was changed as you recommended. The figure was re-edited to combine all 12 individuals in one picture, and we used black squares to emphasize each individual’s prominent characteristic so as to make it clearer for readers. Please go to P15, L285-287 and the file named “Fig2”.

**Comment 45:** **Table 3: 1) Put “No” in the column for Tusk for each female. This is all the more important that you later discuss the fact that you might have misclassified some individuals. 2) Put “Herd” in each file for individuals AE01 to AE08.**

Response: The changes you proposed have been achieved in Table 3 of the new manuscript. Please go to P14, L275.

**Comment 46:** **L. 272-279: This is interesting, but falls out of the scope of the section (Individual identification).**

Response: Yes, we strongly agree with you and thus, this paragraph has been deleted after careful consideration.

**Comment 47:** **L. 283-284: This sentence is not built correctly, and it is unclear what the 1:1 ratio stands for (is it a sex ratio too?). Also, as I mentioned previously, the way you mentioned the criteria for sexing seemed to refer to sexually mature individuals only, so I wonder how you determined sex in juvenile individuals, if the 1:1 ratio is indeed a sex ratio. This needs to be clarified in the methods too!**

Response: We wrote this sentence incorrectly. The 1:1 ratio stands for juvenile individuals as we identified two juvenile males and two juvenile females. But we failed to make it clear in the Methods section. Please go to P16, L290-296.

**Comment 48:** **L. 285: What are “containment relationship family units”?**

Response: Since we wrote this sentence using the wrong words, it has been deleted. Please go to P16, L297.

**Comment 49:** **Table 4: In the title you mention five sessions, but in the table there are only four sessions, one for each sampled month.**

Response: We apologize for the way that it was written. First, we used single month as a single session, and then we used the whole months data as the fifth session. Please see more details in comment 34 or go to P12, L233-236.

**Comment 50:** **L. 306-307: This is the definition of the median!!!!! Please, take this out, or give the mean and here you could assess how skewed the distribution is.**

Response: This sentence has been deleted. Please go to P16, L304.

**Comment 51:** **L. 311-312: Say which sex had the highest BCI. Rephrase “solitary or not” (being in a herd or solitary), and explain which status was associated with having a lower or higher BCS.**

Response: This information has been combined. Please go to P16-17, L307-313.

**Comment 52:** **Figure 3: The figure does not help much, and a table would probably be more efficient here.**

Response: We think a figure would be clearer and more visible than a table. We have improved the figure by changing the order of individuals by placing individuals of the same sex together and ranking them by scores. Please go to the file named “Fig3”.

**Comment 53:** **Table 5: For the adult individual that died in 2003, how can you know “it died for its ivory”? Was it killed (with bullets inside the body)? You could find an individual with the tusks removed post-mortem.**

Response: We read these two references again and deleted one reference which provided us with incomplete information about an adult male that had died for its ivory without any citation nor detailed explanation. So, the description of “the adult male died for its ivory” has been deleted. Please go to Table 5 on P18, L338.

**Comment 54:** **L. 337-341: This is definitely not of interest for the readership of PLoS One.**

Response: These sentences have been deleted. Please go to P19, L364.

**Comment 55:** **L. 343: Use a reference to justify the link you make between the population structure and its future.**

Response: Four references have been added. Please go to P17, L323-327.

**Comment 56:** **L. 343-345: Rephrase that sentence (self contradiction the way it is written), and note that this sentence contradicts the previous one (speaks of a possibly stable population, when earlier you say that it indicates a decline).**

Response: This sentence has been rephrased. Please go to P20, L366-369.

**Comment 57:** **L. 345-346: This sentence comes out of the blue. Link it to an argument. You can actually use it to finish the following sentence with this argument of inbreeding depression in small populations.**

Response: This sentence has been combined. Please go to comment 56 or P20, L366-369.

**Comment 58:** **L. 351-352: Again, a sentence out of the blue, which has no direct link with your results. What argument do you want to discuss in this paragraph? Start with that, and then develop the argument.**

Response: We strongly agree with your comments. This sentence has been changed to “There were two tuskless juveniles that had similar body height as the juvenile males identified by their apparent tusks and these were identified as females. However, it should be noted that males in some populations tend to be tuskless for survival due to the intensive historical poaching pressure. The high proportion of makhnas in NNNR identified from this investigation could indicate that these juveniles that were identified as females might in fact be males.” Please go to P20, L380-385.

**Comment 59:** **L. 355: Why do you say even if it was 1:1.5?**

Response: This mistake has been corrected. Please go to P16, L290-296.

**Comment 60:** **L. 359: Ref. 72 is not about balance in sex ratio in Asian elephant, it is about a sexing method. Make sure it is appropriate to cite it.**

Response: These sentences and reference 72 have all been deleted. Please go to P17, L323.

**Comment 61:** **L. 360-361: In this sentence you refer to tuskless males, but who identified these tuskless males? If it is another study, cite it.**

Response: We identified these tuskless males. Approaches were displayed in Table 1 section. Please go to Table 1.

**Comment 62:** **L. 366: “116 individual deaths (…) might account for tuskless males”. It is unclear here what you mean.**

Response: High poaching pressure could lead to the predominance of tuskless males. Since currently there is a high proportion of tuskless males in this population, we had assumed that the reason for this situation was historical poaching. But since we don’t have data to support this assumption, this sentence has been deleted. Please go to P20, L383-385.

**Comment 63:** **L. 360-370: I find all this difficult to follow because of some problems of clarity, but above all, you discuss a point when you recognize that you do not know for sure if the tuskless individuals you identified were males or females!**

Response: Yes. We judged these juveniles as females under the condition that they have similar body height as juvenile males who had apparent tusks. And at the same time, we discussed that since tuskless males are common in this population, it would be helpful to conduct genetic studies on this population that can confirm the sex of these two juveniles. Please go to P20, L380-387.

**Comment 64:** **L. 379: Add at the end of that sentence that this is especially true due to birth intervals.**

Response: The sentence has been changed to “In either case, the scarcity of fertile females would be the primary constraint on population growth due to their long birth intervals.” P21, L394-395.

**Comment 65:** **L. 398-399: OK, but the way you present it by comparing to India leaves the reader with the idea that these authors (ref 79) did the study over a short period of time. Was this the case, if not, why discuss this and in that way? This is very troubling.**

Response: It has been deleted. Please go to P19, L344.

**Comment 66:** **L. 401: This value (800 m distance) makes sense only if we have an idea of elephant activity areas and movements.**

Response: Here, we cited two references that pointed out that this value was smaller than elephant daily movement distance. Please go to P19, L346-348.

**Comment 67:** **L. 404: Not real, but actual. It is still based on what you captured on photographs.**

Response: Yes, the result should be actual value. The changes have been made in the new manuscript. Please go to P19, L350-351.

**Comment 68:** **L. 405: estimated by whom? Because your sentences are not well linked, it is hard to follow.**

Response: We estimated it. We found the reference by Sukumar (2003) who gave an assumption that a population density of 0.1-0.3 elephant/km^2^ was normal. So, we used this assumption to estimate there should be 20 individuals in NNNR (71 km^2^). In the new manuscript, we deleted this hypothesis. Please go to P17, L329-331.

**Comment 69:** **L. 408: About carrying capacity: well, you said before that 40 years ago the estimate was of 20 individuals, so at 12 it is obvious that you are below (even if we don't know if 20 was at carrying capacity or not).**

Response: The use of the word “carrying capacity” made the sentence less rigorous. This word has been deleted. Please go to P17, L327-331.

**Comment 70:** **L. 394-410: All this paragraph is convoluted. Why make weird assumptions (two sentences above) if you know that the population has already been that size.**

Response: The whole paragraph has been rewritten. Thus, we combined the density section with population size section to make it simpler. Please go to the Population size and density part of the Discussion section. P18, L323-337.

**Comment 71:** **L. 415-418: Rewrite these two sentences, and put what is a result in the corresponding section.**

Response: These two sentences have been placed in the corresponding section. Please go to P16, L307-308.

**Comment 72:** **L. 422-424: So what? Here it is not the males venturing into human areas, but rather humans venturing into elephant territories. It means that males are more tolerant to humans, and has nothing to do with the quest for better food as when they venture in crop areas. You need to thread your arguments better.**

Response: Thank you for your recommendation. This part has been rewritten. Please see P21, L400-401.

**Comment 73:** **L. 424-425: What you mention is well known, but you said that the other females were not lactating, so how does your argument hold?**

Response: The only three adult females in this population were all considerd to be potentially productive, and the body health condition of all of them was considered to be average or even in poor health condition. Our data showed that the individual AE06 who was nursing an offspring had the lowest BCS in this population. Therefore, we mentioned additional nutritional stress from lactation for AE06 could have resulted in an even poorer body condition. Please go to P21, L404-409.

**Comment 74:** **L. 426: What does “reasonable” mean here? Also, you cannot consider the median or the mean as a standard. You may have a very healthy or very unhealthy population and the mean (or the median) can vary quite a lot.**

Response: This sentence has been deleted. We had classified the 11-point scale with three ranks: poor, medium and good. Please go to P13, L259-260.

**Comment 75:** **L. 427-429: Yes, this is true, but this would skew evaluation towards lower BCS for adults, compared to juveniles.... and you found the opposite.**

Response: Yes, that is correct. Maybe the adults have lower BCS than juveniles assuming that we had a large population like previous studies. The truth is the population in NNNR was extremely small, and the fact is that we found that the adults had higher BCS than the juveniles. These sentences have been deleted since we do not want to confuse readers. Please go to P21, L406.

**Comment 76:** **L. 431-438: All this is very speculative, and not very convincing. If the animals raid cultures during the rain season it might be for different reasons, and if they don't during the dry season maybe is it because they find enough food within the reserve. You have to be very careful: you do not have enough information to really discuss all that without speculating.**

Response: We strongly agree with your comments. Please go to P22, L410-422.

**Comment 77:** **L. 438-441: Yes, but you have nothing on that: your data is from one season, in one particular year... not much to inform conservation managers.**

Response: This sentence has been deleted. Please go to P22, L419-422.

**Comment 78:** **L. 445-446: Take out the conflict part. It is not part of this argument and just distract the reader.**

Response: This conflict part has been deleted. Please go to P22, L423-427.

**Comment 79:** **L. 448: “obesity level”. Surely you want to say something else! In nature animals are not obese (or very rarely), only in zoos.**

Response: We wanted to say “health condition”. Please go to P23, L432.

**Comment 80:** **L. 442-451: All this is well beyond what your data can tell.**

Response: We aimed to extend our research to other elephant populations. Although more and more studies have been conducted in China, the study of the BCS has been neglected. Thus, we wanted to draw attention on this kind research for Chinese researchers and conservationists. Please go to P22-23, L423-435.

**Comment 81:** **L. 454: You keep mentioning the question of evenness in the sex ratio, but there is no foundation in your manuscript to justify why an even structure would be better.**

Response: We have deleted this part. Please go to P23, L436.

**Comment 82:** **L. 472-474: About reintroduction. Your study does not confirm that reintroduction is the solution for this population!!! In no way. See my previous comment on this in the introduction.**

Response: All the information related to reintroduction has been deleted. Please go to comment 11 and P6, L112-115 or Conservation Recommendation section.

**Comment 83:** **L. 478: Why mention restoration here? You have never mentioned that the habitat was either destroyed or degraded within the reserve. If it is the case, you should have better described your study area.**

Response: The word “restoration” has been deleted. Please go to Conservation Recommendation section on P436.

**Comment 84:** **L. 485-486: About reintroduction to reduce inbreeding. You have no data on that, do not speculate, especially when recommending such drastic approach as translocation, which comes with lots of cons.**

Response: All the information related to reintroduction has been deleted. Please go to comment 11 and P6, L112-115 or Conservation Recommendation section.

**Comment 85:** **L. 489: Please restrain from mentioning reintroduction. Just managing the population to ensure its viability (if it is possible), would be enough! As I mentioned in the comments to the introduction, reintroduction is rarely a good solution, as the problem is usually with the habitat (quality or quantity) or with the negative interactions with humans.**

Response: All the information related to reintroduction has been deleted. Please go to comment 11 and P6, L112-115 or Conservation Recommendation section.

**Comment 86:** **Figure 3: 1) Eliminate “points” (after Presence, Absence, and Village). 2) The border with Myanmar and the limits of the reserve are similar (same width, same colour). 3) It is har to say where the experimental zone is on the map. It would be better to use the light shade to shade the area.**

Response: All the suggestions that you proposed have been taken into consideration. See more details in file named “Fig 1”.
