# Supplementary material for: Assessing population structure and body condition to inform conservation strategies for a small isolated Asian elephant (Elephas maximus) population in southwest China
Source: PLoS One. 2021 Mar 9;16(3):e0248210. doi: 10.1371/journal.pone.0248210 (PMC7942997; doi:10.1371/journal.pone.0248210)
Supplement: Attachment — Submitted filename: Response to Reviewers.docx [file pone.0248210.s006.docx]

Dear reviewers and editor,

First, we would like to thank the reviewers and editors for your time, comments and expertise. Your exhaustive and positive comments and suggestions again make our paper more rigorous and logistic. We were fully agreed that you made small changes in the main text and reference in manuscript, all the changes were accepted. The following letter gives our response to the reviewer’s specific comments are requested in this Major Revision.

**General Comments of Reviewer #1:**

Response: Thanks for your recognition of our new version manuscript. Here were responses of your two small suggestions.

**Comment 1: Line 323, 'as in' should be 'than in'**

Response: The word has been replaced by the one your recommendation. Please go to P16, L313.

**Comment 2: line 401-402 - You may want to include a short explanation for why larger home ranges and tolerance of disturbance could be linked to higher BCS. Additionally, the sentence starting 'Which means' should instead start 'Tolerance means'**

Response: Large home ranges and tolerance of disturbance means the male elephants obtain more food resources, which contribute a good body condition in turn. The new sentence has been changed like “This may be because adult males are known to have larger home ranges than adult females, and are more tolerant to human disturbance: that means they can explore more frequently areas near the reserve boundary to obtain more food resources, where intensive anthropogenic activities like hunting, grazing, fishing and collecting have also been recorded by cameras.”

**General Comments of Reviewer #2:**

Response: Thank you for your exhaustive and positive comments and suggestions. We made huge changes on Discussion to make it clearer and logistical. In Conservation Recommendations part, we only used two sentences to describe the importance of long-term monitoring of infrared cameras. Then focus on the suggestions that improving food resources to enhance the elephant body condition. This paper has been checked by professional editors, both native speakers of English.

**Comment 1: On line 93-95, This sentence should go Line 80, to finish the paragraph on photo ID.**

Response: This sentence had moved to Line 80 in the new version of manuscript. Please go to P4, L80-82.

**Comment 2: On the line 99-115, These two paragraphs should go right after the first one, when you describe elephant populations in China. Otherwise, these paragraphs do not connect well with the previous ones.**

Response: We deleted some sentences in these two paragraphs and have rewritten remaining sentences. We thought these could be more logical and precise than before. Please go to P5, L95-105.

**Comment 3：On the line 200, Maybe the way you present the table is not optimal, because when you describe the categories in SEX, you include already both sex and age. So maybe you should start describing AGE, and then SEX.**

Response: We adapted your opinion by describing AGE first and then describing SEX which was simply categorized to female and male. Please go to P9-10, see Table 1.

**Comment 4: On Table 1: Ok, so it is obvious that adult and subadult males that are tuskless may be impossible to distinguish; maybe add "and no penis", to distinguish them from their male counterparts; Same, you should add the testicles/penis part, but here, how do you distinguish juvenile from subadult males**

Response: We have simplified the category of SEX by just describing female as “Without obvious tusks but with prominent breasts, belly swelling or the presence of nursing offspring” and male as “With visible testicles or penis, and/or obvious tusks”. See Table 1.

**Comment 5: On the line 210, It should be replaced by "each", even when it is clear that you mean "each and every"**

Response: It should be “each” in this situation. Please go to P11, L222.

**Comment 6：On the line 232, Not too clear to me. You mean to say "i.e."?**

Response: This sentence has been changed into “The file named “capture histories” contained data on individual identified each sampling occasion from the specific detector (camera).” Please go to P11, L221-222.

**Comment 7: On the line 323-327, This sentence is unclear. What do you want to convey here? Do you want to say that different methods lead to different results inveitably, or do you just want to point out that historically the size of the population was estimated at about 20 individuals?I think it is the second case, but in any case by straightforward.**

Response: This sentence has been changed to make it simpler. “Previous studies have estimated this population to be around 20 individuals. Although population estimation using different methods could generate bias in direct comparison, research has shown that this elephant population has remained small during the last few decades.” Please go to P16-17, L313-317.

**Comment 8: On the line 327-330, The logics here remains unclear; with the information provided it is not obvious why being in the same part of the park means that there should be more elephants. Maybe habitat quality is not that good anymore (in fact, the fact that females were not in good body condition might indicate something like that)... or it might be that the highest ever recorded population in the park was an artefact due to loss of habitat in the surrounding (and then a packing effect).**

Response: We did not do any analysis in habitat quality estimation, so it is surprising to readers see the point suddenly. This sentence has been deleted.

**Comment 9: On the line 335, You have four elephants dead since 2003... don't calculate statistics over that! Just say that 3 of the 4 elephants found dead since 2003 have been immature individuals.**

Response: This sentence has been changed to “Additionally, 3 of 4 elephants found dead since 2003 have been immature individuals (Table5).”Please go to P17, L320-321.

**Comment 10: On the line 335-336, This is part of the life history of the species, not somethinf particular to this population. The way it is written, it is misleading.**

Response: This sentence has been rewritten to “Moreover, the fact that elephants have a long adolescence and long inter-birth intervals means it takes time for NGH elephants to recover and they are more vulnerable to local population extinction, especially if young individuals are being poached or dying from other unnatural causes. All these facts result in the current small population size that could lead to the extinction of this local population.” Please go to P17, L321-326.

**Comment 11: On the line 362, Yes, which makes your suggestion that the population could be bigger even more bizarre.**

Response: This sentence has been changed to “The relatively high population density might lead to more intense competition among individuals for limited natural resources, and even greater human-elephant conflict as elephants resort to crop-raiding for food, which could be further studied in the future.” Please go to P19, L350-353.

**Comment 12: On the line 368-369, Why in the near future? Isn't it happening already?**

Response: This sentence has been changed to “Previous study already confirmed these individuals were related to each other [82]. This phenomenon of inbreeding will be more serious in the near future due to the small population size.”Please go to P19, L357-359.

**Comment 13: On the line 387, Why are you talking about unbalanced sex ratio here. it comes from nowhere. I think it should be a new paragraph, and for your reader to follow you, remind them of the sex ratio you found (assuming these individuals were indeed males or not).**

Response: We agreed your comments on this paragraph which makes it more logical and coherence. So, we started a new paragraph to talk about sex ratio and have rewritten some sentences. Please go to P20, L378-387.

**Comment 14: On the line 390-394, This sentence makes no sense. There are two informations here, but how they are related is a mystery.**

Response: This sentence has been deleted.

**Comment 15: On the line 399, Ok, it was observed in another population, but what about another one? You cannot cherrypick information as if were supporting yours. Is there a pattern among small population in crowded places? or in areas with low habitat quality? or anything that could explain why females exhibit this low body condition?**

Response: The reference we cited before had similar situation on population density, but we had smaller study area. So, this sentence has been deleted.

**Comment 16: On the line 401, The comparison is male vs female in adult individuals: leave the immature out.**

Response: The “immature males” has been deleted in this sentence. Please go to P21, L392.

**Comment 17: On the line 403, You surely mean collecting, which you already use.**

Response: Actually, it should be grazing, we used the wrong word. Please go to P21, L394.

**Comment 18: On the line 419, Use a proper way to cite this. If these are unpublished documents, cite the staff who provided the information as personal communication. Also, a documentary is not documentation.**

Response: This citation way has been changed to “Li Zhimin, personal communication”. Please go to P21, L411.

**Comment 19: On the line 437, Sorry, but how documenting this population will ensure its survival? It seems rather a leap of faith!**

Response: Sorry the way we wrote the sentence confused you. This sentence has been changed to “The long-term placement of cameras in NNNR is an efficient mean of monitoring to obtain valuable data on demography, fecundity and body condition variation across seasons and years.” Please go to P22, L429-431.

**Comment 20: On the line 442, Where was it confirmed first? I don't remember reading anything on that earlier in the manuscript**

Response: Sorry for that we used the wrong word. This sentence has been changed to “The results of this study match those observed in earlier studies that providing more suitable habitat is urgently needed to ensure an adequate supply of food for this isolated population during the dry season.” Please go to P22, L433-435.

**Comment 21: On the line 447, of what?**

Response: This sentence has been changed to “The short-term objective to increase the survival quality of this small population should be to compensate for the food shortages the NNNR population during the dry season by establishing more food sources and improving the quality of existing one to make sure all the individuals have a good body condition.” Please go to P23, L438-441.

**Comment 22: On the line 448, by establishing what?**

Response: Please see the response of comment 21.

**Comment 23: On the line 459-460, Do you mean carrying capacity? I don't think it makes sense, unless you start with that and use it to demosntrate that the population, under the current conditions, is not viable.**

Response: We agreed your opinion after deeply considering, so, we deleted the “environmental capacity”.

**Comment 24: On the line 461-463, That recommendation makes no sense. First, there is probably inbreeding since quite long: you do not provide evidence that the connection with another population has been lost recently. Second, in the current state of affairs, it seems that ensuring a better body condition is more important than ensuring reproduction.**

Response: We agreed that you mentioned this population facing the health problems. So, this sentence has been changed to “We emphasize the urgency of implementing these recommendations especially in ensuring a better body condition, which may improve the success rate of reproductivity in the long run.” Please go to P23, L452-454.
