# Supplementary material for: Assessing population structure and body condition to inform conservation strategies for a small isolated Asian elephant (Elephas maximus) population in southwest China
Source: PLoS One. 2021 Mar 9;16(3):e0248210. doi: 10.1371/journal.pone.0248210 (PMC7942997; doi:10.1371/journal.pone.0248210)
Supplement: Attachment — Submitted filename: Response to Reviewers.docx [file pone.0248210.s008.docx]

Dear reviewers and editor,

Special thanks to reviewers and editors for providing exhaustive and positive guidance and suggestions for this paper for so long, which made this paper more and more perfect than the initial version. In this minor revision, we combined two native English speakers’ polished manuscript to make this paper suitable for English standard writing. We hope this version meet PLOS ONE’s publication criteria. The following letter gives our response to the reviewer’s specific comments are requested in this Minor Revision.

**General Comments of Reviewer #2:**

Response: Thank you very much for your recommendation in this minor revision. We have tried our best to revise the manuscript according to your kind and construction comments and suggestions. We have followed your advice and found native English speakers to polish this paper again. All the small problems in this version were fixed this time. We sincerely hope that this revised manuscript has addressed all your comments and you will be satisfied.

**Specific Comments of Reviewer #2:**

**Comment 1:** Abstract: “I do not know if you are already at the limit of words permitted for the abstract, but if you have some space allowed, you should mention the particularly dire body condition of reproductive females.”

Response: The sentence had been changed to “The average BCS was 5.75 (n=12, range 2-9), with adult females scoring lower than adult males.” Please go to Abstract.

**Comment 2:** Instead of writing a very general sentence, you should probably mention your 3 recommendations. Don't forget that most readers don't go further than the abstract.

Response: This general sentence had been deleted. New sentence had been added like “We propose three plans to improve the survival of this population: improving the quality and quantity of food resources, removing fencing and establishing corridors between the east and west parts of Nangunhe reserve.” Please go to Abstract.

**Comment 3:** I have made a number of comments in the abstract that you can find above. The abstract is your selling card!

Response: We totally agreed your opinion and made the Abstract showed more information.

**Comment 3:** On Line 83, Here it is better to make one sentence of the two first sentences, because the first one is not related to the paragraph, which is really about BCS. That's what I suggest with these corrections.

Response: The sentence had been changed to “Effective conservation strategies for small, threatened populations need detailed baseline information on both the population size and its habitat. Population structure and density estimation are other essential features that can be used to study the ecology of wildlife population while, the Body Condition Scoring (BCS) is an index of an animal’s health that reflects habitat quality.” Please go to P4, L81-85.

**Comment 3:** On Line 99, Repeat the complete name here.

Response: The sentence had been changed to “Although it is a unique evolutionary unit in China, the isolated Nangunhe National Nature Reserve population has been poorly studied.” Please go to P5, L 97-98.

**Comment 3:** On line 110, This is a sentence that seems more suited for the conclusion than here.

Response: This sentence had been moved to Conservation recommendations part. Please go to P21, L415-416.

**Comment 3:** On Line 124, I think I already mentioned that previously. If you provide the values for the dry seaon and the rain season, it is no annual mean. The mean annual precipitation is 351.3 + 1983.3 = 2334.6 mm.

Response: The word “annual” had been deleted. Please go to P6, L122.

**Comment 3:** On Line 355, If you use "although" there needs to be an argument to counterbalance. There no such argument in that sentence and it is in fact the following sentence. Because this first sentence is already log, I propose you make the changes I suggest in the text.

Response: The sentence had been changed to “The juveniles in this population accounted for 33% of the population, higher than the minimum of 20% needed for the population to be self-sustaining over a short period.” Please go to P18, L345-346.

**Comment 3:** On Line 379-382, loss of genetic diversity is far from being the most common cause of extinction in small populations!

Response: The sentence had been changed to “Male elephants can recognise kin and avoid inbreeding, and so male-biased populations usually indicate an increased genetic diversity and an ability to persist. However, without introductions from elsewhere, such a small population with only 7 males is likely to go extinct in the near future” Please go to P19, L363-366.

**Comment 3:** On line 438, The sentence was a mess, but also you have nothing to assert that there is some food shortage. You know that BCS tends to be lower, which might be indicative of that, but no proof. However, it makes perfect sense, in a management strategy, to try and provide more food.

Response: The sentence had been changed to “To improve elephant survival in the short term, we recommend establishing more food sources and improving the quality of existing sources to increase individuals’ body condition.” Please go to P22, L424-426.
